# What Makes an Image Interesting and How Can We Explain It

**DOI:** 10.3389/fpsyg.2021.668651

**Published:** 2021-09-01

**Authors:** Maham Gardezi, King Hei Fung, Usman Mirza Baig, Mariam Ismail, Oren Kadosh, Yoram S. Bonneh, Bhavin R. Sheth

**Affiliations:** ^1^University of Houston, Houston, TX, United States; ^2^Faculty of Life Sciences, School of Optometry and Vision Science, Bar-Ilan University, Ramat Gan, Israel; ^3^Department of Electrical and Computer Engineering, University of Houston, Houston, TX, United States; ^4^Center for Neuroengineering and Cognitive Science, University of Houston, Houston, TX, United States

**Keywords:** image complexity, eye scan patterns, eye movements, interestingness, natural scene processing, multiple regression

## Abstract

Here, we explore the question: What makes a photograph interesting? Answering this question deepens our understanding of human visual cognition and knowledge gained can be leveraged to reliably and widely disseminate information. Observers viewed images belonging to different categories, which covered a wide, representative spectrum of real-world scenes, in a self-paced manner and, at trial’s end, rated each image’s interestingness. Our studies revealed the following: landscapes were the most interesting of all categories tested, followed by scenes with people and cityscapes, followed still by aerial scenes, with indoor scenes of homes and offices being least interesting. Judgments of relative interestingness of pairs of images, setting a fixed viewing duration, or changing viewing history – all of the above manipulations failed to alter the hierarchy of image category interestingness, indicating that interestingness is an intrinsic property of an image unaffected by external manipulation or agent. Contrary to popular belief, low-level accounts based on computational image complexity, color, or viewing time failed to explain image interestingness: more interesting images were not viewed for longer and were not more complex or colorful. On the other hand, a single higher-order variable, namely image uprightness, significantly improved models of average interest. Observers’ eye movements partially predicted overall average interest: a regression model with number of fixations, mean fixation duration, and a custom measure of novel fixations explained >40% of variance. Our research revealed a clear category-based hierarchy of image interestingness, which appears to be a different dimension altogether from memorability or awe and is as yet unexplained by the dual appraisal hypothesis.

## Introduction

There has been a surge of interest in interest—as an emotion, what functions it serves, what makes something interesting and its link to happiness (Silvia, 2008). Interest is seen as a counterweight to feelings of uncertainty and anxiety (Kashdan, 2004), but is distinguished from the emotion of happiness in several ways (Turner and Silvia, 2006; Silvia, 2008). One of the issues within this domain that has attracted interest is the question of what kinds of natural, real-world scenes are interesting.

At the heart of this issue is the question of whether there is a hierarchy of preference for different domains. While it is known that scenes of nature are preferred over built scenes (Yang and Brown, 1992; Howley, 2011; Valtchanov and Ellard, 2015), the categories are too broad. Built scenes can consist of outdoor scenes, i.e., cityscapes, or indoor scenes, i.e., shots of the interiors of homes or offices. To date, no one has studied differences in interest between outdoor and indoor built scenes. Natural scenes can be further sub-divided into landscapes and aerial shots, which are scenes shot from near or distant perspectives respectively. It is likely that one’s level of interest in each differs, perhaps because scenes of nature shot at eye level are more in line with our own daily experience and therefore, in the language of Silvia, rates high in appraisals of their comprehensibility (Silvia, 2005, 2006, 2008); on the other hand, scenes shot from the sky are novel relative to our daily experience and also likely to be more complex from the viewpoint of fractal dimension or perceived complexity; therefore, in the language of Silvia, rates high in appraisals of their novelty-complexity (Silvia, 2005, 2006, 2008). New experimental evidence may help resolve this argument. One other category that has not been looked at extensively to date is scenes with people. Human interest in conspecifics is generally high and scenes that show people in isolation or interacting with one another seem interesting, but exactly how interesting remains an open question: interest in scenes with people has not been examined in relation to interest in other domains. In short, the relative interestingness of a wide diversity of visual scenes remains an open question, and a significant one, not only for its own sake but also because interest’s function is to motivate learning and exploration (Silvia, 2008); therefore, this newfound knowledge can be applied to practical problems of learning, education, marketing and others.

A second unanswered question is how we can measure interest. This is an important question as questioning a person as to their interest may not be feasible or practical in some cases, and when it is, the individual may not always be truthful; lack of communication is a painfully stark and real issue for certain sub-populations, e.g., infants, clinical populations such as autism, amyotrophic lateral sclerosis (ALS) and many others.

One way to measure interest is viewing time: intuitively, we expect that more interesting images are viewed longer. Intuition can be correct, though not always: interestingness as a single independent variable in isolation may control viewing time—one study explicitly instructed their participants to view more interesting images for longer (Van den Berg et al., 2016), possibly contaminating any relationship between the two—but the effect of interestingness may be diluted if other independent variables, such as total time spent on the experiment, are introduced into the mix. In any case, the issue of viewing time and interestingness needs to be investigated in broader contexts.

An alternative possibility is eye movements. Studies of eye movements and their relationship to interestingness have just begun. In one landmark study, eye movements were recorded during free viewing of photographs high (landscapes or natural scenes) versus low (cityscapes or urban scenes) on fascination (Berto et al., 2008). One might think that fascination is a proxy for interestingness. Although viewing time was the same on low and high fascination photographs, eye movements related to photographs low on fascination were characterized by greater exploration, i.e., longer scan paths, and a greater number of fixations compared to those rated high on fascination. Thus scenes high on fascination were viewed without really focusing on particular features, which was consistent with Kaplan’s highly influential “soft fascination” hypothesis for explaining preference for nature (Kaplan, 1995). In short, eye movements inform on the interestingness of a photograph, but it is not known to what extent they do, which features contribute and which ones do not.

Yet another possibility is that the interestingness of a scene is related, at least in part, to the scene’s perceived complexity or to computational measures like entropy or fractal dimension that can be computed. Perceived complexity and computational complexity are not interchangeable, although studies claim a strong positive relationship between them. In particular, Stamps has found strong direct positive correlations between entropy values corresponding to variation in aspects of building features and respondents’ estimates of diversity (Stamps, 2002, 2003). However, the tests have been limited to variations of the same scene. In point of fact, a scene is manipulated so as to change the number and type of objects (e.g., shrubs, bushes, branches trees, or simple geometric pattern buildings digitally replaced by more ornate ones) in the scene, and correlations are observed under such conditions (Kuper, 2017). In a related vein, studies have compared perceived and computational measures of complexity such as fractal dimension (Taylor et al., 2011) and Shannon entropy (Kuper, 2017) with visual preference, but the results have varied. On the one hand, studies have claimed that visual preference increases with increase in perceived complexity (Van den Berg et al., 2016). Other studies have claimed that natural environments as opposed to human-made environments tend to be characterized by intermediate levels of complexity, which appear to be just right for attracting attention in a moderate, pleasant way. By contrast, most human-made environments are either highly complex (evoking hard fascination) or virtually lacking in visual complexity and unable to capture attention at all (Wohlwill, 1983). Objective measures of complexity have not been vetted across scenes in most studies and not across domains, and when they have, the results have been disappointing; for example, in comparing natural and built scenes in close-up and distant views, visual aesthetics and a measure known as the restorative effect were found to be significantly *negatively* correlated with visual complexity (Kang and Kim, 2019). Studies have come to a similar conclusion about the limitations of perceived complexity, namely that measures of perceived complexity cannot account for differences between preferences for natural versus built scenes (Kaplan et al., 1972; Sparks and Wang, 2014). The two computational measures of complexity used thus far each have their own limitations: Shannon entropy is maximum for white noise and zero for homogeneous background, and both images are uninteresting; similarly, perceived preference or complexity does not vary monotonically with fractal dimension (in addition, the image has to be reduced to a two-tone image before calculating its fractal dimension. This approach throws out a lot of information, but see Nayak et al. (2015) for a work-around). Alternative new measures that overcome said limitations need to be tested. Even so, image complexity and image interestingness may not be one and the same in the end. This remains to be seen.

## Materials and Methods

### Experiment 1: Interestingness and Image Category

#### Observers

Observers were a set of 47 (23 female) healthy volunteers with normal or corrected-to-normal vision (20 ± 2 years of age). All were naïve as to the purpose of the study. The study was conducted with the understanding and written consent of each participant and under a protocol approved by the University of Houston Committee for the Protection of Human Subjects.

#### Stimuli and Procedure

**FIGURE 1:**
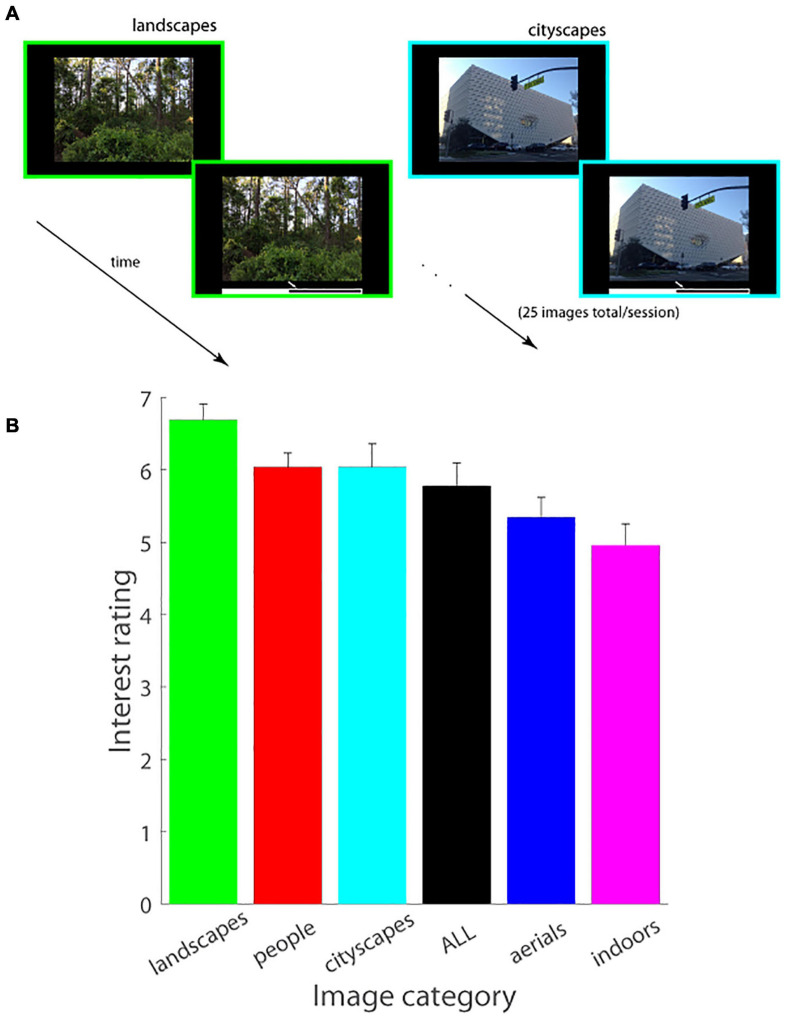
**(A)** Schematic of experimental paradigm (Experiment 1). An image was displayed on the screen in a self-paced manner, followed by a mouse cursor which was used to adjust the image’s interestingness on an analog scale displayed underneath the image. Images of a single domain (figure shows *landscapes* and *cityscapes* as examples) were serially shown in a single session (25 images/session). **(B)** Interest ratings (mean ± SEM.) as a function of image category (*landscapes*, *people*, *cityscapes*, *aerials*, *indoors*) are plotted (Experiment 1). ALL consists of an equal number of images from each of the five image categories.

All experiments were performed on a Windows desktop computer connected to a 21-inch ViewSonic Graphics Series G225f monitor with a refresh rate of 75 Hz and a resolution of 1,280 × 1,024 pixels. Observers sat comfortably in a chair in front of the computer monitor during all components of the experiment. Each image was 1,190 × 893 pixels, and was centered over the screen. Viewing distance, i.e., distance from the head and chin-rest (HeadSpot, UH College of Optometry) to the screen, was 91 cm. Observers (*n* = 47) serially viewed images on a screen in a self-paced manner. All images were chosen from the following two websites: www.pixabay.com and www.pexels.com. Upon viewing an image, the observer had to rate the level of interestingness of said image by sliding a mouse cursor on an analog scale displayed on the screen. Twenty-five images of the same category were presented in sequential order. There were five categories of images: *aerials*, *cityscapes*, *indoors*, *landscapes*, and *people* and a combined mix of all five (*all*). *Aerials* are aerial perspectives of the environment; *cityscapes* are outdoor scenes of urban environments, *indoors* are interior living and office spaces of buildings, *landscapes* are scenes of nature and rural environments, and *people* are scenes containing one or more humans. Observers viewed all 25 images of a particular category in a single session, and viewed different categories of images on separate days; the order of these within-category viewing sessions was randomized across observers (see Figure 1A for schematic of experimental procedure and kinds of stimuli used). The observer’s eyes were tracked using a head-mounted eye tracker (EyeLink II, SR Research, Inc.) and the experiment was designed using ExperimentBuilder software (SR Research, Inc.). A full 9-point calibration was done with the nine calibration points on a 3 × 3 grid covering the corners and edges of the computer screen, with the final point located at the center of the screen. This was conducted at the beginning of every experimental session (one for each image category). Prior to the onset of each image, a shorter single-point calibration was done to ensure that the observer was looking at the middle of the screen before the image came on. Once the eye- tracker verified that they were looking at the middle of the screen, the next image would automatically come on. Calibration was either labeled “good” or “poor” based on EyeLink given standards. The nine point calibration process was repeated until the eye tracker ensured calibration was conducted correctly. Each session typically lasted about 15–20 min, with the variation in time across observers and sessions occurring because all sessions were self-paced.

#### Analysis and Statistics

Interest rating per image was calculated and averaged for each observer and category. A 5-way repeated measured ANOVA [function *ranova()* in MATLAB] was performed comparing across image categories. *Post hoc* Tukey HSD tests were conducted to test for pairwise differences between categories. Viewing time spent per image was averaged for each observer and category as well. Eye tracking data were analyzed and statistical analysis was performed in a similar way as for interest ratings. We computed the Pearson correlation [function *corrcoef*() in MATLAB] between interest rating and other measures. In particular, we studied various known measures of eye movements, including the total number of fixations made while the observer views a given image, the average duration of each fixation, and the average duration of each saccade. In addition to these known eye tracking variables, we looked at custom-designed variables extracted from the data tracking the eye.

In particular, we derived a measure termed Z (zsi), which measured the degree to which fixations were made to previously unexplored locations on the image. A value of 1 of Z means all fixations were to the same location on the screen (or nearby locations within a certain pre-specified radius—see below); lower values of Z means progressively more exploration of the scene. In other words, the smaller the value of Z, less is the spatial overlap between multiple fixations. One may also think of Z as a measure of the spatial variance of fixation locations but with a thresholding component (distant or far locations beyond a certain radius are treated identically). At the beginning of each trial, the value of Z started at 1; the location of each new fixation was compared with the locations of all prior fixations made by the observer to the image, and the value of Z was then reduced by an amount weighted by the degree of spatial overlap between the current and all past fixations. A two-dimensional Gaussian window centered at the location of each previous fixation was used to quantify the degree of overlap; the Gaussian had a standard deviation equal to twice the size of the foveal avascular zone (FAZ) in degrees of visual angle (1.5°); the overlap was the Euclidean distance between the two fixations in units of standard deviation of the 2D Gaussian. Specifically, for each new, current fixation,

$Z=Z-\alpha \times e^{\frac{-{\left(x_{c\,u\,r\,r}-x_{p\,r\,e\,v}\right)}^{2}}{2\,\sigma^{2}}\,\frac{-{\left(y_{c\,u\,r\,r}-y_{p\,r\,e\,v}\right)}^{2}}{2\,\sigma^{2}}}$,

where α is a scaling factor, (*x*_*curr*_,*y*_*curr*_) is location of current fixation, (*x*_*prev*_,*y*_*prev*_) is location of previous fixation, and σ = 3.0° of visual angle. We wondered about the extent to which these eye tracking variables could predict the image’s level of interestingness. To this end, we ran a regression model [functions *fitlm()* and *regress()* in MATLAB] of average interest rating across all observers for each individual image using all the eye tracking variables as regressors, and, following further tests of multicollinearity of the various regressors, a second model with a smaller number of key regressors. We allowed for the possibility that different individuals may have different inherent biases while rating their interest in images and follow different ranges; we normalized the interest rating data for each individual and performed the same analysis for normalized interest rating data (Supplementary Materials provides detailed methods and results from the analysis on normalized data).

### Experiment 2a: Interestingness and Novelty-I

#### Observers

A new set of observers (*n* = 68; 45 female) with normal or corrected-to-normal vision (20 ± 3 years of age) participated.

#### Stimuli and Experimental Procedure

**FIGURE 2:**
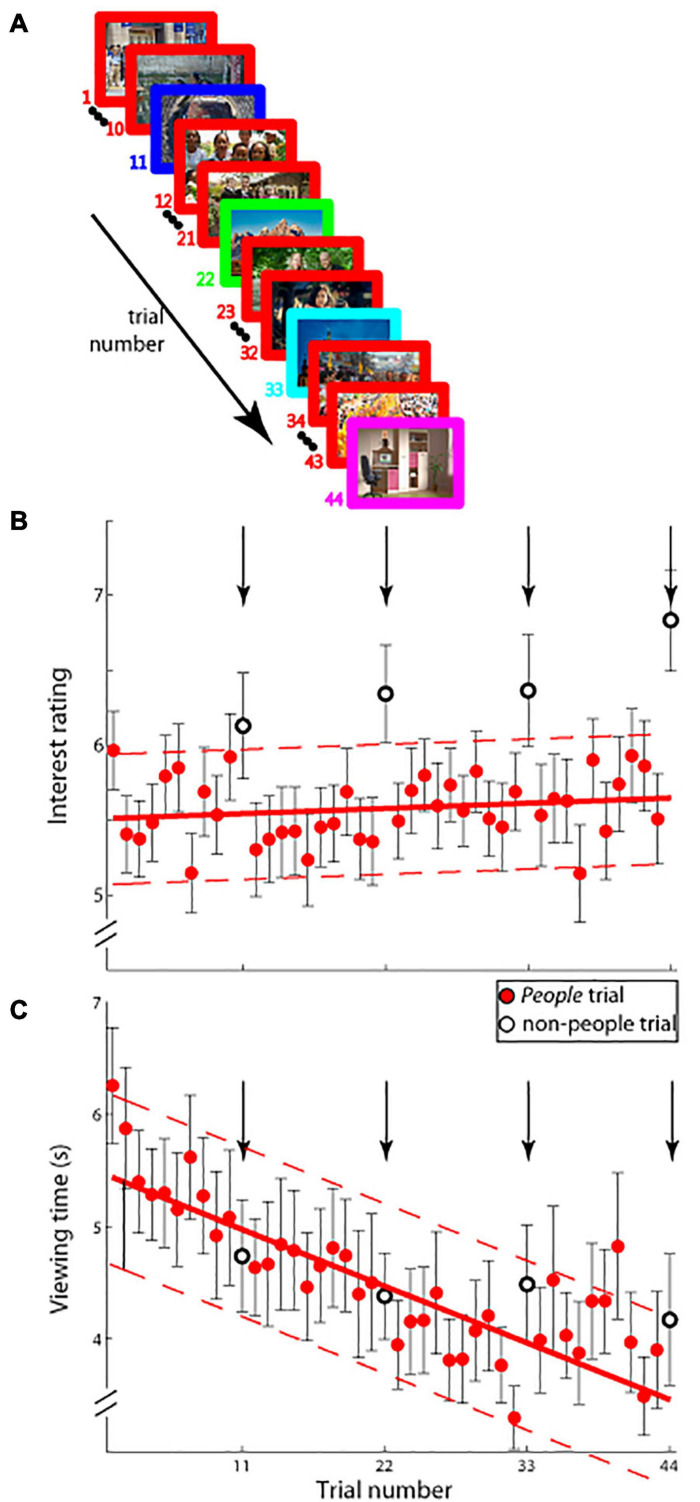
**(A)** Schematic of experimental paradigm (Experiment 2A). On each trial (44 trials total), an image was displayed on the screen and observers viewed it for as long as they desired. Interest ratings were obtained as in Experiment 1. Images from *people* were shown on all trials (red border) except on trial numbers 11, 22, 33, and 44 (borders not colored red), on which images from one of the remaining four categories was shown. **(B)** Interest ratings on a ten-point scale (mean ± SEM.; ordinate) and **(C)** viewing time in seconds; (mean ± SEM; ordinate) as a function of trial number (abscissa) are shown. Group *people* trial data (solid red circles) were fitted with a least-squares linear function (solid red lines) and its 95% confidence interval (dashed red lines). Trial numbers 11, 22, 33, and 44 are shown in open circles and arrows, and were not used in determining the fit.

Observers serially viewed a total of 44 images on a screen in a self-paced manner. Images were presented in the following order: 10 *people* images→1 image from one of the remaining four categories (*aerials/landscapes/indoors/cityscapes*)→10 *people* images→1 image from one of the remaining three categories→10 *people* images→1 image from one of the remaining two categories→10 *people* images→ 1 image from the last remaining category. Thus, observers viewed a total of 40 different images of people, and 4 images, one from each of the remaining four categories. The order of the four categories was counter-balanced across observers. As before, the observer had to rate their level of interest for each image viewed and their eye movements were monitored. Each session typically lasted about 25–30 min, depending on the pace determined by the observer. All other experimental and stimulus parameters were the same as in Experiment 1 (see Figure 2A for schematic of experimental procedure).

#### Analysis and Statistics

We fitted a least squares straight line to the mean interest rating on the 40 *people* trials as a function of trial number. The interest ratings on the four images from the non-people categories other than *people* were compared with the 95% confidence interval of the slope to see whether they stayed within the bounds for the fitted *people* trial value for the corresponding trial number. The same analysis was performed for viewing time spent per image. Eye tracking data were analyzed and statistical analysis was performed in a similar way as for interest ratings.

### Experiment 2b: Interestingness and Novelty-II

#### Observers

An altogether new set of observers (*n* = 68, 41 female, 20 ± 2 years of age) with normal or corrected-to-normal vision participated.

#### Stimuli and Experimental Procedure

**FIGURE 3:**
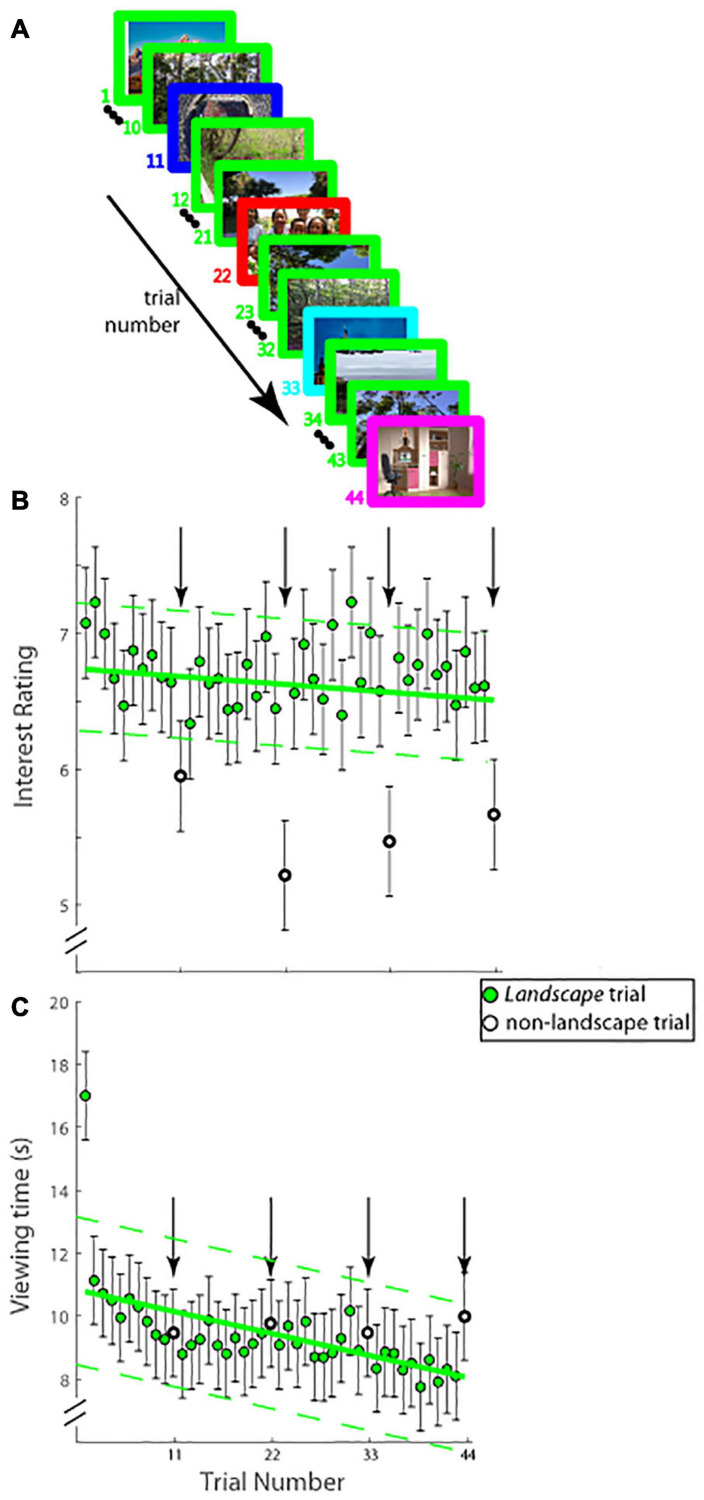
**(A)** Schematic of experimental paradigm (Experiment 2B). On each trial (44 trials total), an image was displayed on the screen and observers viewed it for as long as they desired. Interest ratings were obtained as in Experiments 1 and 2A. Images from *landscapes* were shown on all trials (green border) except on trial numbers 11, 22, 33, and 44 (borders not colored green), on which images from one of the remaining four categories was shown. **(B)** Interest ratings on a ten-point scale (mean ± SEM; ordinate) and **(C)** viewing time in seconds; (mean ± SEM; ordinate) as a function of trial number (abscissa) are shown. Group *landscape* trial data were fitted with a least-squares linear function (solid green lines) and its 95% confidence interval (dotted green lines). Trial numbers 11, 22, 33, and 44 are shown in open circles and arrows, and were not used in determining the fit.

As in Experiment 2a, observers serially viewed images on a screen in a self-paced manner and report on their level of interest in each image using a mouse cursor on an analog scale displayed at the end of each image display. Images were shown in the following sequence: 10 images of the more frequent category, followed by a single image from one of the remaining four categories; this sequence was repeated four times, for a total of 44 images, just as before. The critical difference from Experiment 2a was that here, the more frequent category shown (40/44 images) was landscapes, and one image each from the four remaining categories (*aerials/people/indoors/cityscapes*) was shown. All other experimental parameters and procedures were identical to Experiment 2a (see Figure 3A for schematic of experimental procedure and kinds of stimuli used).

#### Analysis and Statistics

All analysis and statistics were identical to Experiment 2a.

### Interestingness and Image Complexity

#### Jensen-Shannon Divergence (JSD)

We used a well-known measure called Jensen-Shannon divergence (JSD) to compute the low-level complexity of each image. The measure is based on information theory and image segmentation: Unlike in the case of entropy, JSDs of both a uniform gray image and a white noise image are zero, which is in line with human notions of complexity. Briefly, it is a framework based on considering the information channel that goes from the intensity histogram (in each individual color component R, G or B) to the regions of the partitioned image, where the partition of the image into two components chosen is one that maximizes the mutual information—an approach that takes into account the spatial distribution of pixels. Compositional complexity of the image is defined by the Jensen-Shannon divergence of the final partitioned image at which there is asymptotically no gain in mutual information. Details are provided in Rigau et al. (2005) and Sbert et al. (2007).

Code was written in MATLAB to compute the complexity of a given image based on algorithms in the literature (Rigau et al., 2005; Sbert et al., 2007) for computing the asymptotic JSD. JSD was calculated for each image and six successively decimated versions of said image; the decimation factor was 2, so the seven images were the original image and images with resolution reduced by factors of 2, 4, 8, 16, 32, and 64 with respect to the original. We calculated JSD in two different color models: RGB and HSV. For the RGB model, intensity values from the three color channels were combined for each individual pixel (Anderson et al., 1996) and the JSD of the resulting gray level image was then calculated in the manner described briefly above. For the HSV model, the image was converted from an RGB color model to an HSV color model [function *rgb2hsv()* in MATLAB] and the resulting hue (H) channel was used for computing JSD. For both models, a series of JSD values corresponding to the original image and its progressively lower resolution versions was obtained.

We regressed mean interest rating of each image on its JSD, namely the JSD of the original, non-decimated image. The data were fitted with linear (2 free parameters—slope and Y-intercept) and quadratic models (3 free parameters) using the MATLAB function *polyfit()*, and the fits of the two nested models were compared using three different methods: (i) Bayes Information Criterion (BIC) (Schwarz, 1978), (ii) Akaike Information Criterion (AIC)(Akaike, 1974), and (iii) F ratio test. We describe each approach briefly below.

(i) Bayes information criterion (BIC): For a statistical model of the data, with n the number of data points or sample size, *k* the number of estimated parameters of the model, L the maximum value of the likelihood function for the model, the BIC value for the model is given as follows:
  BIC=k⁢ln⁢(n)-2⁢l⁢n⁢(L)
  The model with the lower BIC value is the preferred model.
(ii) Akaike information criterion (AIC): The AIC value for the model is given as follows:
  AIC=2⁢k-2⁢ln⁢(L)
  Again, the model with the lower AIC value is the preferred model.
(iii) The F ratio quantifies the relationship between the relative increase in sum-of-squares (SS) and the relative increase in degrees of freedom (*df*) with the linear fit.
  $$F\,\left(d\,f_{l\,i\,n\,e\,a\,r}-d\,f_{q\,u\,a\,d\,r\,a\,t\,i\,c},d\,f_{q\,u\,a\,d\,r\,a\,t\,i\,c}\right)=\frac{\left(S\,S_{l\,i\,n\,e\,a\,r}-S\,S_{q\,u\,a\,d\,r\,a\,t\,i\,c}\right)/\left(d\,f_{l\,i\,n\,e\,a\,r}-d\,f_{q\,u\,a\,d\,r\,a\,t\,i\,c}\right)}{S\,S_{q\,u\,a\,d\,r\,a\,t\,i\,c}/d\,f_{q\,u\,a\,d\,r\,a\,t\,i\,c}}$$
  If the simpler linear regression model is correct, an F ratio near 1.0 is expected. If the ratio is > > 1.0, then either the quadratic model is correct, or the simpler linear model is still correct, but random scatter led the more complicated quadratic model to fit better. The *p*-value distinguishes between the two possibilities, with a low *p*-value indicating that the more complicated model is the better fit.

We further regressed [functions *fitlm()* and *regress()* in MATLAB] average interest rating across all observers for each individual image on the complete JSD series of values and a higher-order variable we labeled orientation. The variable was a binary-valued variable that indicated if the image was best viewed at a particular orientation, i.e., upright. By this logic, images belonging to the categories *landscapes*, *cityscapes*, *indoors*, and *people* all had a preferred upright orientation. On the other hand, *aerials* were photographs of scenes shot from the sky roughly parallel to the ground and, as such, had no preferred or default upright orientation. Therefore, the variable orientation was set to one for photographs belonging to all the categories except aerials, for which orientation was set to zero.

### Experiment 3 Relative Interestingness of Images Forming a Pair

#### Observers

A new set of observers (*n* = 50, 29 female; 20 ± 2 years of age) with normal or corrected-to-normal vision participated. Note there was no overlap between any of our observers on any of our experiments.

#### Stimuli and Experimental Procedure

**FIGURE 4:**
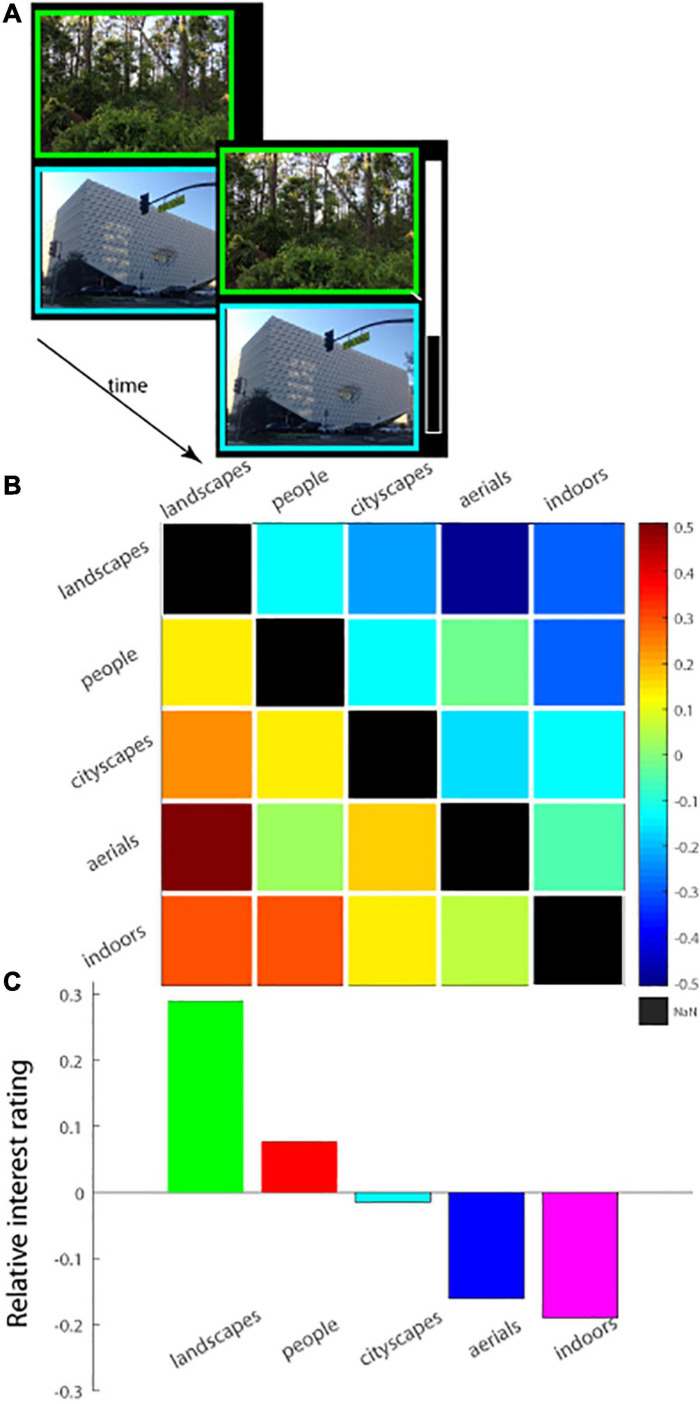
**(A)** Schematic of experimental paradigm (Experiment 3). A pair of images was displayed on the screen, one above the other, and observers had a fixed amount of time to view the images. The two images forming a pair were chosen from different image categories. At trial’s end, the observer rated the interestingness of the images of the pair relative to one another on a vertical analog scale. Top of the scale corresponds to the top image being 100% more interesting than the bottom, and mid-way corresponds to both images being equally interesting; the scale was then re-scaled to lie from 0.5 (top image) to −0.5) for subsequent analysis, as shown in **(B,C)**. **(B)** Relative interest ratings for each pair of image categories shown in a 5 × 5 matrix with each column representing the choice for a particular image category relative to another. Warm colors in a square of the matrix represent preference for one image category over another. **(C)** The interest ratings along each column in **(B)** were summed and averaged and then plotted, showing the overall relative interest rating for each image category integrated across all the remaining image categories.

Observers serially viewed a pair of images on a screen, one above the other. There were 40 pairs of images, with the images of a given pair belonging to two separate categories, chosen from the same five categories as before, namely *aerials*, *cityscapes*, *indoors*, *landscapes*, and *people*. Each pair of categories $\left(\left({}_{2}^{5}\right)=10\right)$ had a total of four image pairs; the display order (top/bottom) was counter-balanced between the two image categories for each category pair. Images were carefully paired so that their JSD values clearly and significantly differed: the more complex image of a given pair averaged 5.961 ± 0.121 bits, whereas the less complex image averaged 4.127 ± 0.158 bits [*t*(39) = 12.359, *p* < <0.00000001], and the difference in complexity within the pair was 50.7 ± 5.1%. The sign of the difference was counter-balanced between the two categories (i.e., on 2/4 (*landscape*, *aerial*) pairs, the *landscape* image JSD was greater, and on the remaining 2/4 the *aerial* image JSD was greater). Each pair was displayed for 8 s, after which the observer had to judge the relative interestingness of each image in the pair by sliding a cursor over a vertical, analog scale and clicking on the location along the scale. The midpoint of the scale represented equal interest in the two images of the pair (corresponding to a value of +0 on the normalized rating scale), and a click on either extreme (top/bottom) of the scale indicated all interest in the image at the corresponding location on the screen (corresponding to a value of +1.0 on the normalized rating scale for the preferred image of the pair) and none in the remaining image of the pair (−1.0 on the normalized rating scale for the non-preferred image). Observers’ eye movements were monitored. Each session typically lasted about 10–12 min, depending on the time it took to judge the relative interestingness for each pair. All other experimental and stimulus parameters were the same as in Experiment 1 (see Figure 4A for schematic of experimental procedure and display).

#### Analysis and Statistics

The relative interest rating (range: −1.0 to 1.0) was calculated for each image of the pair for each individual observer and averaged across all observers separately for each image pair. Ratings such obtained were then combined for each pair of image categories and statistics were conducted. The following analyses were performed (i) The mean interest ratings for each category were compared statistically in a 5-way ANOVA [function *anova1()* in MATLAB]. *Post hoc* Tukey HSD tests were conducted to test for pairwise differences between categories. (ii) The percentage of observers that preferred one image of a given pair over another was calculated and binned by image category. Viewing time spent per image was averaged for each observer and category as well. A 5-way ANOVA followed by Tukey HSD tests were conducted to test for pairwise differences between categories. (iii) The number of images of a particular category that were preferred by more than 50% of observers was calculated. Again, a 5-way ANOVA followed by Tukey HSD tests were conducted to test for pairwise differences between categories on this measure.

Because the images forming each pair were selected because of their difference in complexity, we asked if the more complex image was found to be more interesting. The following analyses were performed on the ratings obtained for each image pair and each observer. (iv) The percentage of observers that preferred the more complex image of a given pair was calculated. The percentages obtained for all 40 image pairs were then arcsine and square-root transformed before running a *t*-test to determine if the percentage was significantly different from chance (50%).

## Results

### Landscapes Are Most Interesting, Indoor Scenes Are Least Interesting

The same set of observers viewed images of different categories in different sessions and rated their level of interest in each individual image. Interestingness data, averaged across observers and images, is shown in Figure 1B, which illustrates that images of *landscapes* were the most interesting, and *indoor* scenes were the least interesting of all categories tested. A 6-way repeated measures ANOVA test (five categories plus the catch-all ALL session containing an equal number of images from each) demonstrated a highly significant statistical difference in the interestingness of different image categories tested [*F*(5, 120) = 133.89, *p* < < 0.000001; *MS*_*e*_ = 0.0769]. *Post hoc* Tukey HSD tests further showed that *landscapes* were judged significantly more interesting than all of the other image categories (*p* < 0.0001 on all pairwise comparisons with other image categories), interest in *cityscapes* versus *people* was statistically indistinguishable across our population sample (*p* = 1.00) and significantly higher than the interestingness of each of the remaining categories (*p* < 0.0001 on all pairwise comparisons), and *aerials* were significantly more interesting than *indoors* (*p* < 0.0001) with *indoors* being the least interesting statistically of all image categories considered (*p* < 0.0001 on all pairwise comparisons of indoors with all other image categories. Note that a qualitatively similar pattern of results, i.e., *landscapes* were the most interesting image category on average and significantly so as compared to *indoors* and *aerials* (see Supplementary Figure 1, Supplementary Table 1, and Supplementary Materials for details), was observed on the ALL session in which new images from all five categories were displayed and judged for interest level.

### Complexity and Distribution of Color (hue) of an Image Cannot Explain Its Interestingness

**FIGURE 5:**
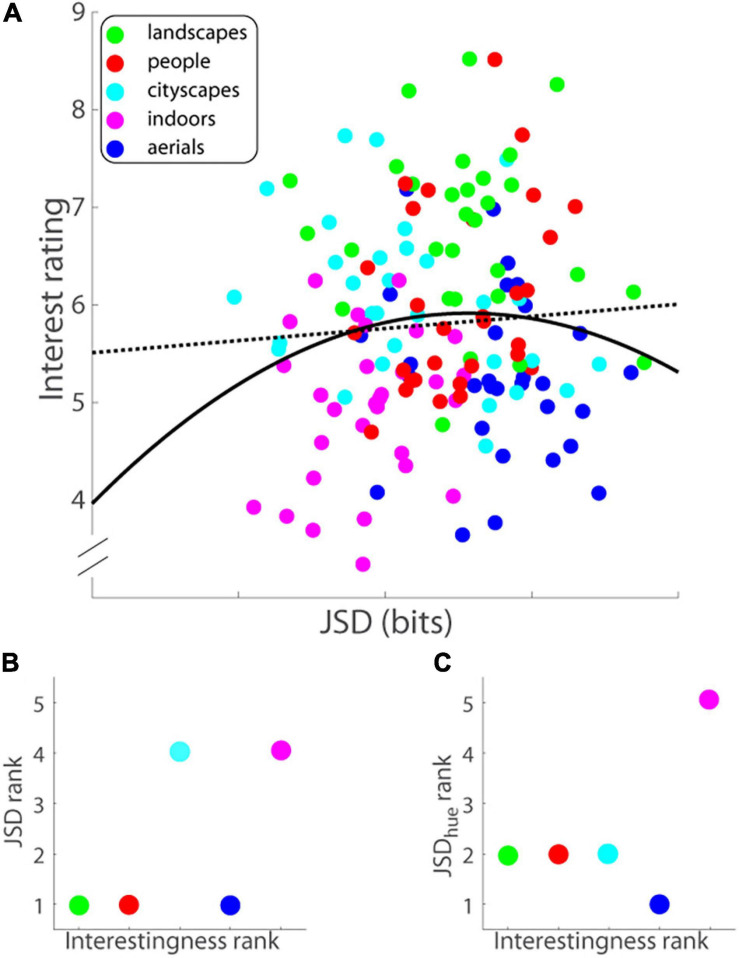
**(A)** Interest rating on a ten-point scale (ordinate) versus image complexity or Jensen-Shannon divergence in bits (abscissa) for each image (Experiment 1). Data were fitted with least-squares optimal quadratic (solid black line) and linear (dotted black line) functions. **(B)** JSD rank of image category (ordinate) versus rank of category interestingness (abscissa). JSDs were computed of RGB formatted images. **(C)** JSD_hue_ rank of image category (ordinate) versus rank of category interestingness (abscissa). RGB images were converted into HSV format and JSDs were then computed of the H (hue) component of the image.

One possible account for an image’s interestingness is its inherent image complexity, the argument being that the more complex an image, the more interesting it appears. Alternatively, both too simple images and too complex ones are not as interesting as an image that has intermediate level of complexity and that hits the “sweet spot” in between the two extremes. To answer this question, we computed the complexity of each image and its successively decimated (by increasing powers of 2) versions using a measure called Jensen-Shannon Divergence (JSD) (Rigau et al., 2005; Sbert et al., 2007). Briefly, in the past image complexity has been related to Shannon entropy of the image intensity histogram—the entropy of a white noise image would be maximum, which is not commensurate with human perception. In contrast, the new measure takes into account the spatial distribution of pixels, and recursively segments the image into binary partitions based on mutual information between them, which, in turn, quantifies the degree of structure or correlation in said image (Rigau et al., 2005) (notably, the JSD of a homogeneous image as well as that of a white noise image will be zero, in accord with human perception). JSD of an image represented in either RGB or HSV formats can be calculated. Here, we present the findings from calculating the JSD of an RGB image (Supplementary Materials presents the findings from calculating the JSD of an HSV image). Interest ratings as a function of JSD (RGB format) are illustrated in Figure 5A.

It has been suggested that an object’s aesthetic value is closely related to image complexity (Moles, 1971). Apropos, images that are simultaneously visually complex and easy to process should be the ones that have a higher aesthetic or interestingness value (Machado and Cardoso, 1998). JSD is a measure that is supposed to capture this sweet spot between image complexity and processing complexity. From this logic, one posits a U shaped function of interest rating as a function of image complexity as measured using JSD. We tested this hypothesis by comparing quadratic and linear fits to the data (Figure 5A; solid and dotted lines, respectively) using three different measures. First, Bayes information criterion (BIC) values for the quadratic and linear models of the data were 23.05 and 20.77, respectively, with the lower value of the linear model indicating that the linear function is the better model. Akaike information criterion (AIC) levies a lower penalty for the number of parameters as compared to BIC, and therefore is more biased toward the more complicated model—indeed, the AIC value of the quadratic model was 14.01 compared with 14.75 for the linear model, which, strictly speaking, suggests that the quadratic model was a slightly better fit but notably the difference in AIC values was negligibly small—less than 1. Finally, an *F*-test showed that the quadratic model was not a statistically better fit of the data [*F*(1, 147) = 2.030, *p* = 0.156]. In sum, the three measures put together show that there are no grounds to discard the more parsimonious linear model of interest rating versus JSD in favor of the more complicated quadratic model.

Although the linear model of JSD to interest rating was a more parsimonious fit than the quadratic model, the linear model still did not significantly predict its interestingness: The slope of the linear fit (−0.062; black dotted line in Figure 5A) was not significantly different from zero [*t*(148) = −0.830, *p* = 0.408]. We then regressed mean interest reported for each image on the series of seven JSD values corresponding to said image and its successively decimated versions (see section “Materials and Methods” above for details), but it too barely accounted for the variance in reported average interest level [*R*^2^ = 0.019, adjusted-*R*^2^ = −0.023, *F*(7, 142) = 0.383, *p* = 0.911]; none of the seven JSD predictor coefficient estimates was individually significant either (all *t*-statistics were less than 1.05, all *p*s ≥ 0.29). The findings were similar for the distribution of hue in the scene (see Supplementary Figure 2A and Supplementary Materials for details).

Next, we wondered if complexity and color combined may explain observers’ reports of interestingness of the images in the experiment. A mixed-effects model, with image (the same images were shown to observers) and image category as repeated measures and JSD and JSD_hue_ as covariates (image category, JSD, JSD_hue_, image category ^∗^ JSD and image category ^∗^ JSD_hue_ as fixed effects), was fit to the data on reported image interestingness. The model was implemented on SPSS. It yielded results that were in line with the assertion that low-level stimulus accounts were not sufficient to explain the data: a strong effect of image category on interestingness [*F*(4, 5938) = 36.512, *p* < 10^–34^] remained, in spite of significant but considerably smaller effects of JSD [*F*(1, 5938) = 6.253, *p* = 0.012], JSD_hue_ [*F*(1, 5938) = 13.877, *p* = 0.0002] and interactions of each with image category interestingness [category ^∗^ JSD: *F*(4, 5938) = 15.237, *p* = 2.120^∗^10^–12^; category ^∗^ JSD_hue_: *F*(4, 5938) = 2.866, *p* = 0.022]. Pairwise comparisons of the image categories (Bonferroni adjusted for multiple comparisons) yielded similar results as before: *Landscapes* was statistically the most interesting of all categories, *people* and *cityscapes* were statistically indistinguishable and each statistically more interesting than each of the two remaining categories—*aerials* and *indoors*, which were themselves not distinguishable statistically in reported interestingness (note that the term JSD ^∗^ JSD_hue_ was statistically not significant, and the third-order interaction term category ^∗^ JSD ^∗^ JSD_hue_ was uninterpretable and were therefore removed in model pruning conducted earlier). Note that only the last result was somewhat different from before (see Figure 1), suggesting that category-wide differences in JSD and JSD_hue_ between *aerials* and *indoors* could explain away differences in reported interestingness. Overall, however, the results of the mixed model analysis suggest that low-level differences in computational complexity and color were not able to explain away any of the other differences in interestingness. In short, interestingness is a higher-order phenomenon of visual cognition that, at least to a first approximation, low-level stimulus differences cannot adequately account for.

Could differences in complexity account for differences in interestingness at a coarser level, i.e., across image category? There were significant between-category differences in JSD [*F*(4, 120) = 16.016, *p* = 1.559^∗^10^–10^, MS_*e*_ = 0.975, see Supplementary Figure 2B]; *post hoc* Tukey tests revealed, among others, that *aerials* had significantly higher JSD than *cityscapes*, which runs counter to results on interestingness). More importantly, there was no correlation found between complexity and interestingness of image category (Spearman’s ρ = 0.577, *p* = 0.400; Figure 5B); for example, *aerials* as an image category had the highest complexity on average but were rated second to last in interestingness. In line with the last analysis, we asked if differences in the distribution of another low-level measure, namely color, account for the differences in interestingness at the image category level. Specifically, we asked if there were significant between-category differences in the distribution of color (hue) that paralleled overall ratings of category interestingness. Again, there were significant between-category differences in JSD_hue_ [*F*(4, 120) = 17.535, *p* = 2.347^∗^10^–11^, MS_*e*_ = 1.280, see Supplementary Figure 2C]; *post hoc* Tukey tests revealed *aerials* had significantly higher JSD_hue_ than *landscapes*, *people*, and *cityscapes*—all of the above run counter to results on interestingness), but more importantly, no correlation was found between JSD_hue_ and interestingness of image category (Spearman’s ρ = 0.105, *p* = 0.867; Figure 5C).

In counter to the failure of low-level measures to account for an image’s reported level of interestingness, the addition of a single, relatively simple higher-order variable, termed orientation, to the JSD predictors helped the new model account for a small but highly significant fraction of the variance in image interestingness across the image sample. The new model with the inclusion of the new variable was able to predict image interestingness better [*R*^2^ = 0.127, adjusted-*R*^2^ = 0.078, *F*(8, 141) = 2.57, *p* = 0.0119]; furthermore, the orientation/uprightness predictor coefficient estimate was individually significant [β_orientation/uprightness_ = 0.967 ± 0.231, *t*(141) = 4.195, *p* = 0.0000481]. None of the coefficient estimates corresponding to the JSD series of predictors was statistically significant (all *p*s > 0.05), and therefore offered negligible predictive value to the image’s interestingness.

### Observers’ Eye Scan Patterns Partially Explain an Image’s Interestingness

**FIGURE 6:**
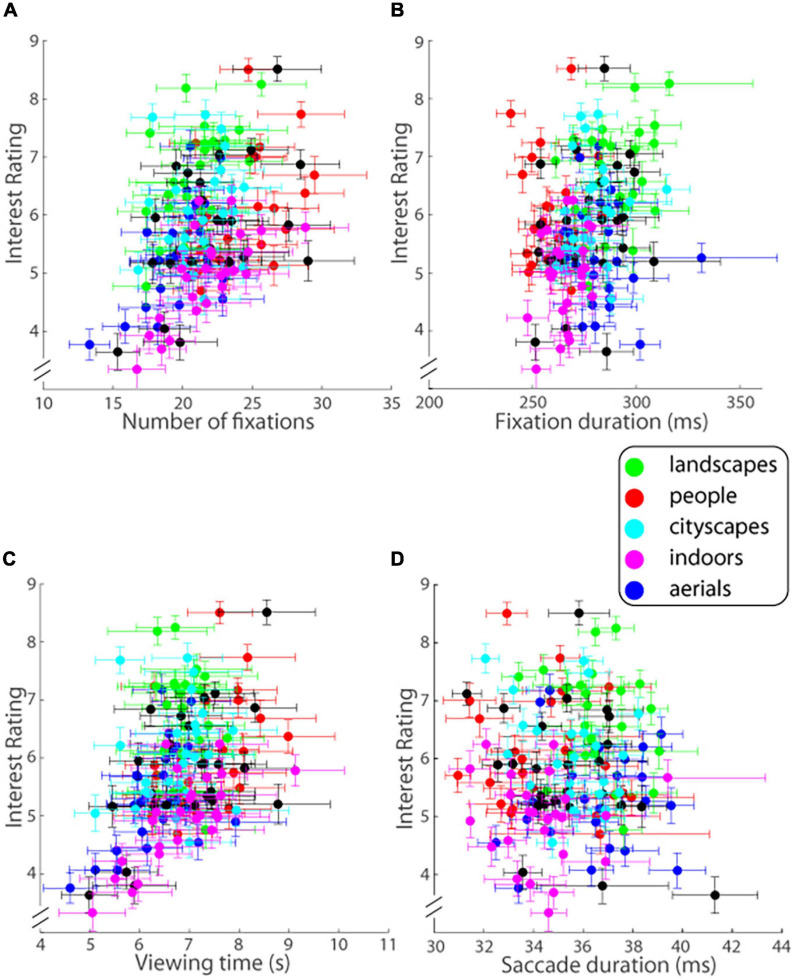
Interest ratings on a ten-point scale (mean ± SEM.; ordinate) as a function of **(A)** number of fixations, **(B)** fixation duration, **(C)** viewing time, and **(D)** saccade duration (abscissa) for each image of each of the five image categories as well as the ALL mix bag (Experiment 1). Error bars are one SEM.

In contrast, the eye scan pattern of observers did provide insights into the image’s interestingness. Key eye tracking measures were found to co-vary with image interestingness. In particular, the total number of fixations (F) made on a given trial and ensemble averaged across all observers was highly significantly correlated with the level of interest reported in the image (*r* = 0.397, 95% CI = [0.253, 0.524], *p* = 0.00000047764; Figure 6A). The duration of fixations (Fd), averaged for each image viewed and then ensemble averaged across all observers for the given image (Figure 6B), was correlated with observers’ ratings of image interestingness (Pearson’s *r* = 0.235, 95% CI = [0.078, 0.381], *p* = 0.0037) but not to the same extent as Fd. Viewing time (V) on a given trial again ensemble averaged across observers was significantly correlated with the level of interest reported in the image (r = 0.421, 95% CI = [0.279, 0.544], *p* = 0.0000000828; Figure 6C). In contrast to these variables, the average duration of saccades (Sd; note that saccade duration is tightly linked with saccade length), was not correlated at all with the level of interest reported (*r* = −0.028, *p* = 0.737; Figure 6D).

Combining the different standardized eye tracking variables above to predict the outcome of interest rating across the population in a multiple linear regression procedure, it was found that the standardized predictor variables accounted for a moderate but highly significant amount of the variance in reported interest level across all images shown [*R*^2^ = 0.378, adjusted-*R*^2^ = 0.331, *F*(10, 134) = 8.134, *p* = 0.000000000344; *MS*_e_ = 0.742]. The coefficient estimates (βs), t-statistics and *p*-values for each predictor variable are shown in Table 1: increases in either the average duration of each fixation (Fd) or number of fixations (F) or was significantly associated with increase in the level of interest reported whereas no association was seen between the average duration of saccades while viewing the image (Sd) or total viewing time (V) and level of interest. No significant interactions between the predictors were found except for a small but significant one between number of fixations (F) and viewing time [V; all other *p*s > 0.15 Table 1; therefore, a linear model with no interactions between the eye tracking variables also accounted for a highly significant amount of the variance in reported interest level: *R*^2^ = 0.328, adjusted-*R*^2^ = 0.308, *F*(4, 140) = 17.045, *p* = 0.0000000000207; *MS*_*e*_ = 0.767; Supplementary Table 2].

**TABLE 1:** Multiple regression model of interest rating (I) as a function of all eye tracking predictor variables I = β_0_+β_1_*F + β_2_*Fd + β_3_*V + β_4_*Sd + β_5_*F*Fd + β_6_*F*V + β_7_*F*Sd + β_8_*Fd*V+ β_9_*Fd*Sd+ β_10_*V*Sd.

Variable (s)	Coefficient estimate ± SEM	*t*-statistic	*p*-value
F	β_1_ = 0.813 ± 0.240	3.383	0.00094052
Fd	β_2_ = 0.448 ± 0.105	4.260	0.000038284
V	β_3_ = −0.197 ± 0.219	–0.900	0.3703 (ns)
Sd	β_4_ = 0.092 ± 0.080	1.156	0.250(ns)
F*Fd	β_5_ = −0.173 ± 0.127	–1.371	0.173 (ns)
F*V	β_6_ = −0.153 ± 0.055	–2.766	0.0064848
F*Sd	β_7_ = 0.235 ± 0.246	0.955	0.342 (ns)
Fd*V	β_8_ = 0.101 ± 0.153	0.659	0.512 (ns)
Fd* Sd	β_9_ = 0.058 ± 0.123	0.476	0.635 (ns)
V*Sd	β_10_ = −0.223 ± 0.225	–0.992	0.323 (ns)
Intercept	β_0_ = 5.879 ± 0.092	63.862	<<0.0000001

The number of fixations made (F) while viewing an image is expected to increase the longer one views the image. Our results bear this out—a high correlation between number of fixations (F) and viewing time (V; Supplementary Figure 3) was seen (*r* = 0.872, 95% CI = [0.827, 0.905], *p* < < 0.00000001). This was reflected in the high variance inflation factor values (i.e., ≥5) for the two variables. In addition, it was observed that there was a strong, negative correlation between F and Fd, which accounts for the highly significant conditional effect of Fd on the model above (i.e., the high correlation between Fd and the residuals obtained when regressing F onto interest rating). Because F was a better predictor of level of interest reported than V, it was included in the final reduced model, which contained two variables, namely F and Fd, with an interaction term between them. The leaner, two predictor variable model, shown in Table 2 still managed to account for a moderate but highly significant amount of the variance in reported interest level [*R*^2^ = 0.328, adjusted-*R*^2^ = 0.315, *F*(3, 146) = 23.783, *p* = 0.00000000000137; *MS_*e*_* = 0.768]; the conditional predictions of both variables of image interestingness were highly significant.

**TABLE 2:** Reduced regression model of interest rating (I) as a function of number of fixations (F) and fixation duration (Fd) I = β_0_ + β_1_*F + β_2_*Fd + β_3_*F*Fd.

Variable (s)	Coefficient estimate ± SEM	*t*-statistic	*p*-value
F	β_1_ = 0.599 ± 0.080	7.533	0.00000000000477
Fd	β_2_ = 0.471 ± 0.078	6.084	0.00000000981
F*Fd	β_5_ = 0.012 ± 0.064	0.189	0.850 (ns)
Intercept	β_0_ = 5.810 ± 0.075	77.121	<<0.000000000001

Next, we studied the pattern of fixations. Specifically, we quantified the tendency of the viewer to make fixations to the same region(s) of the image previously fixated on earlier in the trial with a new, custom-designed parameter Z (see section “Materials and Methods” for details): a value of Z = 1 meant that the viewer always fixated on new, previously unexplored regions of the image, whereas a value of Z = 0 meant the opposite, i.e., they re-fixated on the same region of the image throughout their viewing. We expected that more interesting images would have several interesting regions that the viewer would visit, meaning that fixations will be to new, previously unexplored image regions. As predicted, the parameter Z associated positively and significantly with image interestingness (*r* = 0.178, 95% CI = [0.019, 0.329], *p* = 0.0290): the more interesting the image, the less likely the viewer was to revisit previously fixated regions. Incorporating Z into the previous regression model (with no interaction terms) helped improve substantially the model fit to image interestingness [*R*^2^ = 0.350, adjusted-*R*^2^ = 0.326, *F*(5, 139) = 14.943, *p* = 0.00000000000999; *MS*_*e*_ = 0.748—compare with the previous high *R*^2^ = 0.328 of a model that did not include Z]—the coefficient corresponding to Z was significant in the new model (Table 3; also see Supplementary Table 3 for the full interaction model, which, with the addition of Z, also shows a gain in R^2^ from 0.378 to 0.409). A minimalist regression model with F, Fd, and Z as predictors outperformed other models of similar size [*R*^2^ = 0.366, adjusted-*R*^2^ = 0.340, *F*(6, 143) = 13.766, *p* = 0.00000000000261; *MS*_*e*_ = 0.740; Table 4].

**TABLE 3:** Regression model of interest rating (I) as a function of eye tracking predictor variables and Z I = β_0_+β_1_*F + β_2_*Fd + β_3_*V + β_4_*Sd + β_5_*Z.

Variable (s)	Coefficient estimate ± SEM	*t*-statistic	*p*-value
F	β_1_ = 0.677 ± 0.182	3.725	0.000284
Fd	β_2_ = 0.440 ± 0.092	4.806	0.00000395
V	β_3_ = −0.059 ± 0.171	–0.347	0.729 (ns)
Sd	β_4_ = 0.061 ± 0.079	0.778	0.438 (ns)
Z	β_5_ = 0.168 ± 0.078	2.173	0.0315
Intercept	β_0_ = 5.802 ± 0.072	80.800	<<0.0000001

**TABLE 4:** Regression model of interest rating (I) as a function of F, Fd, and Z I = β_0_+ β_1_*F + β_2_*Fd + β_3_*Z + β_4_*F*Fd + β_5_*F*Z + β_6_*Fd*Z.

Variable (s)	Coefficient estimate ± SEM	*t*-statistic	*p*-value
F	β_1_ = 0.619 ± 0.078	7.897	0.000000000000686
Fd	β_2_ = 0.444 ± 0.078	5.700	0.0000000661
Z	β_3_ = 0.178 ± 0.074	2.409	0.017
F*Fd	β_4_ = 0.008 ± 0.065	0.122	0.903 (ns)
F*Z	β_5_ = 0.011 ± 0.077	0.142	0.888 (ns)
Fd*Z	β_6_ = −0.087 ± 0.079	–1.092	0.277 (ns)
Intercept	β_0_ = 5.830 ± 0.075	77.256	<<0.0000000000001

Finally, we address the issue of differences in individual bias. It is possible that different individuals in our sample bring inherently different subjective biases while rating how interesting they find the same images and observers’ ratings may also vary across different ranges of values. However, the results were robust to such bias: Normalizing ratings in two different ways—a within-observer Z-scored interest rating, and a within-observer minmax normalized interest rating (see Supplementary Materials: Methods for further details on methodology)—yielded similar results to those obtained on the raw data (Supplementary Tables 4–7), with the same two predictor variables—number of fixations (F) and the duration of fixations (Fd)—being the only two variables in a multiple regression model showing a significant correlation with reported level of interest (Supplementary Tables 4–7).

### Longer Viewing Times Do Not Necessarily Imply More Interesting Images

One of the surprising findings from the above analysis is that viewing time does not have a positive association with interest, as indicated by a lack of a significant effect of viewing time as a predictor of interest rating in the multiple regression models tabled above.

We further investigated this issue in a new experiment (Experiment 2A) with an altogether new set of observers. Here, we showed a sequence of 44 images, with 40/44 images being of the same category and every 11th image shown being of one of the remaining categories. Figure 2B shows pooled interest ratings as a function of trial number: note that on all but trials 11, 22, 33, and 44 different images from the *people* category were displayed, whereas trials 11, 22, 33, and 44 showed images from each of the four remaining categories—*landscapes*, *cityscapes*, *aerials*, and *indoors* (in randomized, counter-balanced order across observers). The data were fitted with a least-squares linear fit (solid line in Figure 2B) and the 95% confidence interval estimates were calculated and plotted (dotted lines in Figure 2B). As Figure 2B shows, interest ratings on the critical trials displaying novel categories fell outside the 95% confidence interval estimates, indicating that interest in images selected from non-people categories was higher than expected based on interest in images shown from the predominantly displayed *people* category (40/44 images shown were *people*): The average reported level of interest in the images from the non-people categories was outside the 95% confidence intervals of the linear fits on each of the four non-people trials—trial 11 (mean interest rating: 6.130 vs. 95% CI: [5.102, 5.986]), trial 22 (6.344 vs. 95% CI: [5.137, 6.013]), trial 33 (6.366 vs. 95% CI: [5.164, 6.049]), and trial 44 (6.835 vs. 95% CI: [5.184, 6.092]). This result was in stark contrast with the data on viewing time: as Figure 2C illustrates, and statistical analysis confirms (trial 11—mean viewing time: 4,741 ms vs. 95% CI: [4,235 ms, 5,771 ms]), trial 22–4,384 ms vs. 95% CI: [3,781 ms, 5,303 ms], trial 33–4,491 ms vs. 95% CI: [3,313 ms, 4,849 ms]), and trial 44–4,174 ms vs. 95% CI: [2,831 ms, 4,409 ms]), the average viewing time on the infrequent non-people category trials was within the 95% confidence intervals of the linear fit of the data, indicating that viewing time and interest were not aligned. The dissociation was also observed when comparing interest versus viewing time data on the more frequent *people* trials: the linear fits in Figures 2B,C had significantly different slopes [m_Interest_ = 0.00284 vs. m_ViewingTime_ = −41.911; *t*(76) = 8.907, *p* < 0.000000001], and the correlation between mean interest ratings and mean viewing times was not significant (Pearson’s *r* = −0.048, 95% CI = [−0.354, 0.268], *p* = 0.769). In brief, the findings illustrated in Figure 2 demonstrate that, on average, more interesting images were not necessarily always viewed for longer periods.

### Novel Image Category ≠ More Interesting Image

One interpretation of the above result is that interest spikes while images from novel categories were displayed rarely and intermittently while images of a single category were shown far more frequently, indicating that novelty of category may relate to the image’s interestingness. We tested this idea in a new experiment that was a variation of the previous one on an altogether new set of observers. The design of Experiment 2B was identical to the previous one’s with the exception being that *landscapes* constituted the frequent category (40/44 images), while images from the other four remaining categories (*people*, *cityscapes, aerials*, and *indoors*) were each shown once.

The results, illustrated in Figure 3B, are similar to those of Figure 2B—but with one key difference. As before, the reported level of interest in images from the rarely shown categories was outside the 95% confidence intervals—trial 11 (mean interest rating: 5.933 vs. 95% CI: [6.249, 7.198]), trial 22 (5.187 vs. 95% CI: [6.232, 7.172]), trial 33 (5.458 vs. 95% CI: [6.206, 7.155]), and trial 44 (5.675 vs. 95% CI: [6.172, 7.146]); however, here, unlike in the previous experiment, the level of interest was significantly *lower* than that for the frequently shown category of images (*landscapes*; Figure 3B). Viewing time, illustrated in Figure 3C shows that the average viewing time on the rare non-landscape category trials was not different statistically from the linear fits of the frequent *landscape* category trial data (trial 11—mean viewing time: 9,423 ms vs. 95% CI: [7,858 ms, 12,422 ms]), trial 22–9,733 ms vs. 95% CI: [7,099 ms, 11,621 ms], trial 33–9,480 ms vs. 95% CI: [6,298 ms, 10,862 ms]), and trial 44–10,041 ms vs. 95% CI: [5,456 ms, 10,144 ms]), meaning novelty of image category did not affect appreciably average viewing times. A similar dissociation as in the previous experiment between viewing time and interest was observed on the frequent *landscape* trials: the slopes of the linear fits in Figures 3B,C were significantly different [m_Interest_ = −0.002 vs. m_ViewingTime_ = −70.910; *t*(76) = 5.071, *p* = 0.00000271], while the correlation between the mean interest ratings and mean viewing times was not significant (Pearson’s *r* = 0.130, 95% CI = [−0.174, 0.411], *p* = 0.401). These results confirm the findings from the previous experiment, namely that time devoted to viewing the image was dissociated from interest in said image. Furthermore, because level of interest reported on the infrequent novel category trials was significantly lower than that on the frequent *landscape* trials, novelty of image category was not a proxy for level of interest.

### Pairwise Category Comparisons: New Paradigm, Similar Results

One question that remained is if the findings, i.e., landscapes were the most interesting and image complexity was not predictive of observers’ level of interest, were limited to experimental paradigm, in which images from the same category were displayed in a single session and images from different categories were presented in separate sessions. To this end, in the new Experiment 3, we serially presented pairs of images—from two separate categories—and had observers indicate their relative level of interest in them (40 pairs, 4 each per category combination). Images were carefully chosen so that one image was decidedly more complex than the other in terms of JSD value (details are provided in Methods: Experiment 4). All combinations were tested and counter-balanced (so there were an equal number of trials in which the *landscape* image was more or less complex than the one belonging to the *indoor* category, for example; see section “Materials and Methods” for details). The results of the experiment confirmed key findings. First, landscapes were the most interesting, and indoor scenes were the least interesting of the image categories tested: The mean relative normalized interest rating (Figure 4) statistically differed among the five image categories [one-way ANOVA, *F*(4, 15) = 4.4254, *p* = 0.0146; *MS*_*e*_ = 0.342]; *post hoc* Tukey HSD tests further showed that *landscapes* were judged significantly more interesting than *aerials* as well as *indoors* (Figure 4B, left column, bottom two squares and Figure 4C)—results in line with those of Experiment 1 described above. Similarly, the proportion of observers (*n* = 50; Supplementary Figure 4) that preferred images from certain categories over others also differed statistically [one-way ANOVA, *F*(4, 15) = 4.5293, *p* = 0.0134; *MS*_*e*_ = 0.01487]; *post hoc* Tukey HSD tests again showed that a significantly larger proportion of observers judged *landscapes* more interesting than *aerials* or *indoors* (Supplementary Figure 4B, left column, bottom two squares and Supplementary Figure 4C). Finally, we calculated the proportion of trials for which more than half of all observers preferred a particular category; here again, certain categories were more preferred significantly over others [one-way ANOVA, *F*(4, 15) = 3.9244, *p* = 0.0225; *MS*_*e*_ = 0.0896; Supplementary Figure 5]; *post hoc* Tukey HSD tests showed that *landscapes* were more frequently chosen than *indoors* (Supplementary Figure 5B, left column, bottom square and Supplementary Figure 5C). Overall, the category-based hierarchy of interestingness that was observed in Experiment 1 was maintained, by and large, in Experiment 3 in all the analyses.

Second, image complexity did little to account for the level of interest reported for the image. The more complex image of the pair was judged more interesting by an average of 50.2 ± 0.04 (SEM.) % of observers, which was not significantly different from chance [*t*(39) = 0.123, *p* = 0.903]; moreover, the more complex image was judged more interesting by more than half of observers on a mere 21/40 = 52.5% of image pairs (Supplementary Figure 6), which was not statistically distinguishable from chance either (*p* = 0.636, binomial test). It bears mention that this result is not surprising in light of the previous one showing how image category governed observer preferences; because image complexity was counter-balanced for image category thus eliminating any inherent correlation between image complexity and category, an effect of complexity on interestingness was not likely to have been found anyway.

Third, relative viewing time was associated with the relative interestingness of the image in the pair. There was a positive and statistically significant correlation between the relative interestingness of an image averaged across observers and the percentage of total display time spent viewing it (Pearson’s *r* = 0.368, 95% CI = [0.162, 0.544], *p* = 0.0008; Supplementary Figure 7A). The finding remained unchanged when the median values of the two variables—interestingness and viewing time—were considered instead (Pearson’s *r* = 0.368, 95% CI = [0.161, 0.544], *p* = 0.0008). Recall that in this experiment, each pair of images was displayed for a fixed 8 s and the observer had no control over the time that the pair of images stayed on. Thus, observers were forced to look at one of the images of the pair being displayed in this paradigm. The correlation between the mean relative interestingness of the image in the pair and the mean number of total fixations to the pair was positive and statistically significant (Pearson’s *r* = 0.261, 95% CI = [0.244, 0.455], *p* = 0.019; Supplementary Figure 7B) as well (the relation between the medians of the two variables was similar and significant).

## Discussion

Here, we investigated the question of interestingness; in particular, what makes a photograph interesting to an individual. We explored the contributions of novelty, low-level explanations based on image complexity, exposure or viewing time, and image category to reported level of interest; we further explored the extent to which eye scan patterns that individuals make while viewing a scene provides a window into their level of interest in the scene.

At least three commonly held intuitions (held by the authors) about interestingness were refuted, or at least strongly altered, by the emerging experimental evidence. First, we expected that more interesting images would be viewed for longer times, and that image interestingness would drive viewing time in self-paced paradigms. There was a positive, direct correlation between image interestingness and viewing time when it was considered in isolation (Experiment 1), and also when the duration over which images were displayed was fixed and not under observer control (Experiment 3); however, in a multiple regression model that included other variables including number of fixations, *decrease*, not increase, in viewing time was significantly associated with increase in level of interest reported. Furthermore, in another set of experiments (2a and 2b), no correlation between interest levels and viewing times was found—interest levels changed significantly—spiked or dipped in different experiments—for images from novel categories, but viewing time did not change correspondingly at all. Thus, while viewing time for a given image was correlated with reported level of interest, and especially in experiments in which display time was not under observer control, viewing times were driven by several other factors, including trial number in self-paced settings. In short, image interestingness turned out to be but one factor that could affect viewing time. In this light, our current, working hypothesis is that the relative percentage of time spent viewing an image of a pair is associated with its interestingness relative to that of the other image, under the condition that the time period that each pair is displayed for remains fixed; this implies that the observer’s gaze must be monitored in order to figure out how much time is spent viewing each image of a pair displayed on the screen; therefore, viewing time is, at best, not even useful unless other variables are measured.

Second, we expected novelty to trigger interest, specifically, for images from a novel category to attract a higher level of interest, but our findings showed otherwise. Unseen images from a less interesting category were not reported to be more interesting just because they belonged to a novel category, indicating that the interestingness of an image is not affected by short-term manipulations but is rather a stable, sustained phenomenon.

Third, we expected that more complex images would be more interesting, either up to a point (before becoming less interesting with still higher levels of complexity), or monotonically throughout. However, the information theoretic complexity of the image (and of its scaled versions) had hardly any relationship—linear or quadratic—with its level of interestingness.

The last result dovetails with the idea that high-level accounts based on semantic content rather than low-level accounts are key to unlocking what makes an image interesting, and in particular with our finding that image category significantly influences observers’ preferences. Across experimental paradigms and different sets of observers, one of the most consistent findings was that landscapes were the most interesting category of images viewed, and indoor scenes and aerials were the least interesting of all. The finding begs the question: Why? There are several candidate underlying causes. One possibility is sampling bias, namely that the landscapes shown here were somehow fortuitously more interesting and indoor and aerial scenes shown here somehow were fortuitously least interesting. This possibility can never be fully eliminated, but it is not likely: different paradigms had different sets of images for each of the categories including landscapes, and the results were consistent across paradigm. Moreover, images of all categories were professionally shot and there was no built-in bias for landscapes or against aerials—which are somewhat paradoxically scenes of the natural environment generally but shot from an aerial perspective—and indoors that we could discern. A second possibility, coined “no two landscapes are alike” is that for landscapes, more so than for any other category tested, each image is new and uniquely different from others of the same category, and therefore more interesting. In the first experiment, images from a single category were all shown in the same session one by one, and landscapes were found most interesting. Therefore, the possibility is at least consistent with the data. However, in another experiment in which we showed two images from two different categories, landscapes were still found to be preferred the most when the pairwise preferences were integrated across all category combinations. Therefore, “no two landscapes are alike” cannot by itself explain all of the present data.

A third possibility is related to the second but is different: Of all the categories of images tested, perhaps landscapes hit the “sweet spot” between familiarity and novelty, and the sweet spot is what imparts landscapes their interestingness. In the case of landscapes, the objects are familiar (trees, water, sky etc.), but the way in which they are arranged could be novel, which hits the “sweet spot”; in the case of aerials on the other hand, objects are shot from unreal perspectives, rendering them unfamiliar at first glance; in the case of scenes of people, the typical viewer is unlikely to be familiar with any of the people in the scenes in the study; in the case of cityscapes and indoor scenes, the configurations of familiar objects may be too rigid (i.e., chairs, desks, sofas are arranged in familiar patterns in an indoor scene as are skyscrapers and roads and cars in cityscapes) to be able to hit the sweet spot. The last possibility is the most intriguing of all and we have plans to put these speculations to the test. Past studies of the roles of familiarity and novelty in viewing preference, which was not the same as mere exposure duration (Shimojo et al., 2003), just as in our study—reveal intriguing parallels to the present findings: Image category does affect whether familiarity or novelty preference emerges from repeated stimulus exposure (Park et al., 2010). Faces of people elicit familiarity preference, but natural scenes, or landscapes, elicited novelty preference (Park et al., 2010), especially when rather than passive viewing, active preference judgments were made (Liao et al., 2011)—just as in our study.

We argue further that the novelty familiarity axis is related to Silvia’s influential dual appraisal hypothesis (Silvia, 2008), which states that interest stems from evaluations of an event or scene in terms of both its novelty-complexity and its comprehensibility, which involves considering whether the person has the skills, resources and knowledge to deal with it. One would expect that an individual would have the resources and knowledge to deal with familiar items. Taking this argument further, the sweet spot account squishes the two-dimensional novelty-comprehensibility space into a single dimension with novelty and familiarity at its two ends. While this connection is speculative and perhaps interesting, we caution that the dual appraisal account—or, for that matter, the “sweet spot” account—is not a magic wand. For example, the dual appraisal account is consistent with the argument that people should be the most interesting: our observers had never met the people shown in our photos, so *people* should rate high in novelty; at the same time, a lifetime of dealing with fellow humans means that *people* should also rate high in comprehensibility. However, *people* were not the most interesting image category (see the Introduction for why the dual appraisal account could be construed to argue for why *aerials* should be the most or least interesting; one could construct an argument based on novelty/comprehensibility that *landscapes* should be least interesting). While these counter-arguments do not invalidate either of the accounts, it does show that more details (perhaps based on differences between items and context or other differences) need to be fleshed out to explain the hierarchy of interestingness discovered here.

An issue addressed here was if one’s eye scan pattern while viewing an image foretells its interestingness. The answer was yes—to an extent. A regression model of mean interest rating consisting of eye scan pattern regressors accounted for a modest (*R*^2^ = 0.409) but highly significant amount of variance in the interest rating data: a higher number of fixations, longer fixation durations, and fewer fixations to previously explored regions of the image, all combined, predicted greater interest in the image. Upon hindsight, the findings are not entirely unexpected—more interesting images presumably have more interesting regions; each interesting region should theoretically grab the viewer’s focus, and for a longer duration; once attended, interest in said region ought to decline to levels below those for other interesting regions of said image, which, in turn, will then move up to the front of the queue for future fixations. However, one should not over-emphasize the predictive power of eye movements on image interestingness. A large amount of variance in the interestingness data remained unaccounted for. Moreover, the model was based on group or aggregate data. That is to say, it predicted the image’s interestingness to an average or canonical viewer from the average of the eye scan parameters across the sample. Predicting how interesting an individual viewer will find an image based on their single-trial eye scan pattern remains an unachieved holy grail.

There are several questions raised by our study. Is an image’s interestingness an intrinsic property, or one that is specific to experiment condition and can be affected by extrinsic factors? The ability to experimentally manipulate an image’s interestingness to an observer can have important applications. In the absence of a comprehensive battery of tests as yet, we do not have a definitive answer to this question. Experiment 1 tests whether image interestingness is intrinsic and provides an affirmative answer. Experiment 2 examined if the extrinsic factor of viewing history has an effect on interestingness and it did not. Experiment 3 examined if the presence of a second image affects it and it did not appear, to a first approximation, to be the case: note the remarkable similarity in the hierarchy of interestingness between Experiments 1 and 3. Thus, so far, it appears that interestingness is a somewhat “sticky” intrinsic property of the image that is relatively unaffected by experimental condition or by extrinsic factors in general.

A second crucial question raised is how the interestingness of an image is related to its memorability: are more interesting images more memorable? The best way to answer this question is to directly investigate the memorability of our images on a different set of observers. We are in the process of so doing. In the meantime, studies of memorability offer tantalizing hints. To quote from the seminal study of image memorability (Isola et al., 2011), “blue and green outdoor landscapes [are] remembered less frequently than more warmly colored human faces and indoor scenes.” In other words, the more interesting scenes of landscapes are less memorable than the less interesting indoor scenes and scenes of people. Only experimental evidence on our images can verify this assertion, but we can tentatively claim for now that an image’s interestingness is likely to be a different measure altogether than its memorability.

A third crucial question raised is whether the emotion of awe underlies the proposition and more importantly, the findings here. Awe is an emotion that “lies at the upper reaches of pleasure and on the boundary of fear” (Keltner and Haidt, 2003), and arises from a perception of vastness and a need to accommodate the perception into existing mental schemas (Chirico and Yaden, 2018). Awe is considered to be a response to nature, one believed to have originated from human search for the right place to seek shelter (Appleton, 1996)—conditions that are most often fulfilled by elevated locations with a sweeping view of the surrounding area and this sweeping view of natural scenery happens to be the stereotypical and most prevalent elicitor of awe in contemporary settings (Chirico and Yaden, 2018). Supporting this assertion, a preference in children for paintings of sweeping scenery viewed from an elevated position has been reported (Fischer and Shrout, 2006). Does awe explain our findings? We do not think so: First, we report a hierarchy of interestingness—landscapes are more interesting than cityscapes and scenes of people, which are more interesting than aerial scenes, which are, in turn, more interesting than indoor scenes—that cannot be entirely explained by awe. Second, it appears from studies of awe that aerial scenes, and not landscapes taken from ground view, would be most awe-inspiring; aerials were not the most interesting category in our study. Nevertheless, it is possible that interestingness and awe intersect internally and teasing the two apart is a direction for future study.

**FIGURE 7:**
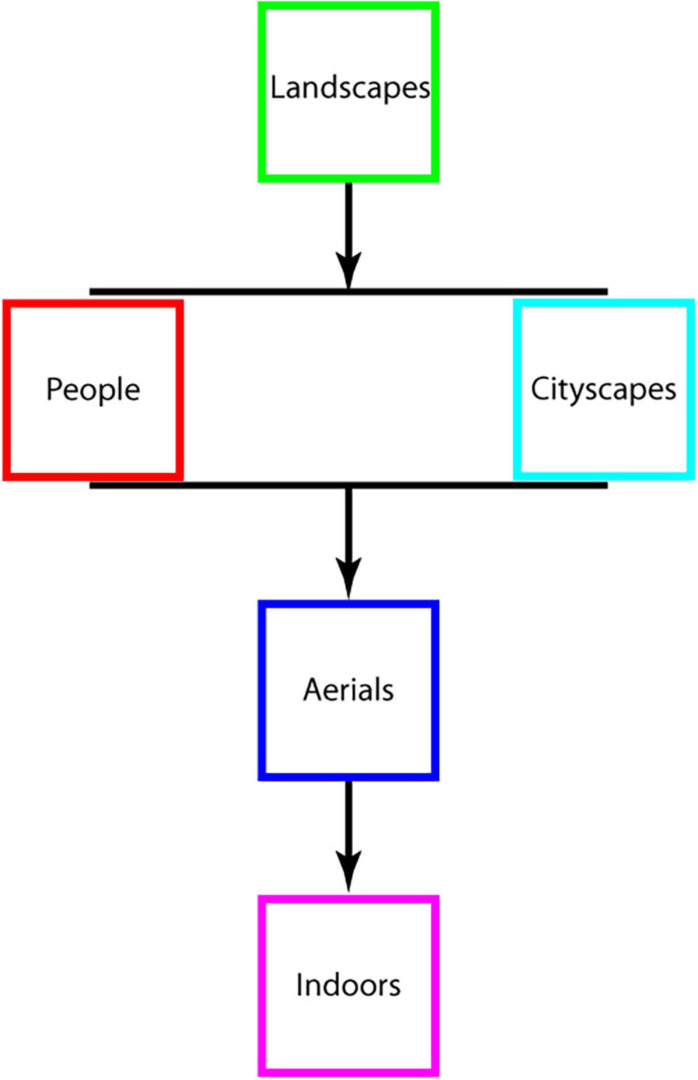
Schematic illustrating the hierarchy of image interestingness revealed by our study.

We end with a summary of our study’s unique contributions. Our study focused on two broad classes of questions: (1) What makes a scene interesting? (2) How can we measure interest in a scene? Regarding question 1, while it has been shown that scenes of nature are preferred over built scenes, scenes come in a wide range of categories. Built scenes can be indoor or outdoor scenes; natural scenes can be scenes taken from ground level of vast, sweeping views from high up and people may or may not be present. Our study is the first to investigate the relative interestingness of a diverse, representative spectrum of image categories and to demonstrate a clear hierarchy (Figure 7). We further asked if interestingness is an intrinsic property of an image or specific to experimental condition, i.e., can it be influenced by extrinsic factors? We found evidence that interestingness is intrinsic, unaffected by viewing history or by changes in experimental paradigm; therefore, image interestingness appears hard to manipulate by an external agent. A strongly held claim in the literature is that an image’s complexity is intimately connected with its interestingness. However, prior studies had tested this out on variations of the same image (e.g., digitally varying number and kind of items). We put this claim to a more rigorous test: across image and across image category, but did not find a relationship. More generally, we showed that low-level measures like complexity and hue do not, to a first approximation, offer significant predictive power over an image’s interestingness but high-level measures (uprightness/orientation of scene) do. Another commonly held belief is that more interesting images are viewed for longer. We systematically examined this and found little support: other variables (total time on test) influence viewing time more strongly. Finally, is the question of whether a viewer’s eye movements can betray their interest in the image. The first report on this issue found correlations between certain features of eye movements and fascination. We delved deeper into this claim: we statistically compared the relative contributions of several features of eye movements over a diverse set of image categories and found that the number of fixations made was, by far, the most significant predictor (more interesting images entail more fixations). Overall, eye movements could explain but a modest amount (∼40% of the variance) of interestingness across our stimuli. New, heretofore studied, variables may improve upon the modest predictive power of eye movements: perhaps, mid-level accounts based on the idea that the more interesting images are those that blend familiarity and novelty may be able to explain the different levels of interest reported for the different image categories. Knowledge of what kinds of images are interesting and what makes images interesting is likely to have application in a gamut of areas such as marketing, business, public service announcements: in general, any area in which information is to be disseminated to a wide and diverse audience with a limited attention span.

## Data Availability Statement

The raw data supporting the conclusions of this article will be made available by the authors, without undue reservation. Photos used in Experiments 1 and 3 are found in Supplementary Material.

## Ethics Statement

The studies involving human participants were reviewed and approved by Committee for the Protection of Human Subjects, University of Houston, Houston, TX 77204, United States. The patients/participants provided their written informed consent to participate in this study.

## Author Contributions

BRS conceived of the project and helped write software to run experiments to acquire and analyze data and wrote the manuscript. MG, KF, UB, and MI screened and ran participants and helped analyze the data and create graphs. MG analyzed the eye tracking data and ran statistics. YB and OK wrote the software to analyze eye movements and microsaccades and helped in the use of their software to analyze the data in the manuscript. YB edited and revised the manuscript. All authors contributed to the article and approved the submitted version.

## Conflict of Interest

The authors declare that the research was conducted in the absence of any commercial or financial relationships that could be construed as a potential conflict of interest.

## Publisher’s Note

All claims expressed in this article are solely those of the authors and do not necessarily represent those of their affiliated organizations, or those of the publisher, the editors and the reviewers. Any product that may be evaluated in this article, or claim that may be made by its manufacturer, is not guaranteed or endorsed by the publisher.
