# Supplementary material for: What Makes an Image Interesting and How Can We Explain It
Source: Front Psychol. 2021 Sep 1;12:668651. doi: 10.3389/fpsyg.2021.668651 (PMC8440840; doi:10.3389/fpsyg.2021.668651)
Supplement: Supplementary file 1 [file Table_1.DOCX]

**Methods**

Z-score: For each observer, their interest ratings provided for all the images in the experiment were normalized (function *normalize()* in MATLAB): $z= \frac{x-\mu}{\sigma}$ , where *x* is the raw interest rating provided for a given image and observer, and $\mu$* *and $\sigma$ are the mean and standard deviation respectively of interest ratings across all 25 images for the same observer, and $z$ is the normalized interest rating (Z-scored interest ratings have zero mean and unit variance). The same calculations as above were performed for eye tracking variables in the Z scored analysis.

Minmax: For each observer, their interest ratings provided for all the images in the experiment were minmax normalized:$m= \frac{x-min}{\max- min}$, where *x* is the raw interest rating provided for a given image and observer, and *min* and *max* are the minimum and maximum interest ratings across all 25 images for the same observer, and m is the normalized interest rating.

Regression models (using functions *fitlm()* and *regress()* in MATLAB) were run on normalized interest ratings and results are provided in Supplementary tables (Supplementary tables 3, 4 for Z-score normalized and Supplementary tables 6, 7 for minmax normalized values) below.

**Results**

**Suppl. Table 1:** Interest ratings of image categories on the *all* session

| Image  category | Interest rating  (mean±s.e.m.) |
| --- | --- |
| *Landscapes* | 6.96±0.43 |
| *People* | 6.08±0.39 |
| *Cityscapes* | 6.07±0.23 |
| *Aerials* | 4.91±0.32 |
| *Indoors* | 4.85±0.40 |

*F*(4,20) = 6.103, *p* = 0.002, *MS_e_* = 0.653. Post-hoc Tukey tests showed that the interest reported on *landscapes* was significantly higher than the interest reported on *aerials* and *indoors* (see Suppl. Fig. 1).

**JSD_hue_** **vs. interest rating**

The original image in RGB format was converted to an HSV format (*rgb2hsv()* function in MATLAB) The H (hue) component of the HSV formatted image was used to calculate the JSD.

We plotted JSD_hue_ of the original image versus reported mean interest rating (Suppl. Fig. 2A) and compared quadratic and linear fits to the data (Suppl. Fig. 2A; solid and dotted lines, respectively). The results for JSD_hue_ did not follow the results on JSD (RGB) described in the main text: BIC value for the quadratic model of the JSD_hue_ vs. interest rating data was lower than that for the linear model – 21.13 and 27.03, respectively; AIC revealed a similar result: AIC value of the quadratic model (12.10) was lower than the AIC of the linear model (21.01) – both BIC and AIC side-by-side comparisons of the two models showed that the quadratic model of the data was a better fit; an *F*-test confirmed that as well (*F*(1,147) = 7.267, *p* = 0.008). The quadratic model of interest rating versus JSD_hue_ was thus better than the simpler linear model.

Based on the above result, it was not unsurprising that the JSD_hue_ of an image failed to linearly predict its interestingness. The slope of the linear fit (-0.011; black dotted line in Suppl. Fig. 2A) was not significant (*t*(148) = -0.173, *p* = 0.863). Further, a model of interest rating regressed on the entire series of JSD_hue_ values failed (*R*^2^ = 0.084, adjusted-*R*^2^ = 0.039, *F*(7,142) = 1.86, *p* = 0.081) as well, and only one of seven individual JSD predictor coefficient estimates (JSD_hue_ of 🡫16 image) was individually significant (*p* = 0.006). Thus, linear differences in the distribution of hue across our stimuli failed to explain observers' reports of their interestingness.

The JSD_hue_ model with the inclusion of the orientation/uprightness predictor predicted image interestingness to a modest degree (*R*^2^ = 0.135, adjusted-*R*^2^ = 0.086, *F*(8,141) = 2.76, *p* = 0.00735) just as was the case for the image in the RGB format; furthermore, the orientation/uprightness predictor coefficient estimate was individually significant (β_orientation/uprightness_ = 0.685±0.237, *t*(141) = 2.894, *p* = 0.0044). The coefficient estimate corresponding to the JSD_hue_ for the 🡫16 image was significant as well (β_JSDhue16)_ = 2.979±1.139, *t*(141) = 2.616, *p* = 0.0099). None of the other coefficient estimates corresponding to the remaining JSD_hue_ predictors was significant (all other *p*s > 0.12). In summary, the findings were remarkably similar for the JSD of the image in HSV and RGB formats.

**Suppl. Table 2:** Regression model of interest rating (I) as a function of eye tracking predictor variables

I = β_0_+ β_1_*F + β_2_*Fd + β_3_*V + β_4_*Sd

| Variable(s) | Coefficient estimate  ±s.e.m. | *t*-statistic | *p*-value |
| --- | --- | --- | --- |
| F | β_1_ = 0.720 ± 0.183 | 3.937 | 0.00012951 |
| Fd | β_2_ = 0.485 ± 0.095 | 5.358 | 0.000000337 |
| V | β_3_ = -0.111 ± 0.171 | -0.651 | 0.5161 (ns) |
| Sd | β_4_ = 0.097 ± 0.078 | 1.234 | 0.2193 (ns) |
| Intercept | β_0_ = 5.802 ± 0.073 | 79.738 | <<0.0000001 |

R^2^ = 0.328, adjusted-*R*^2^ = 0.308, *F*(4,140) = 17.045, *p* = 0.0000000000207; *MS_e_* = 0.767, *RMS_e_* = 0.876

**Suppl. Table 3:** Multiple regression model of interest rating (I) as a function of all eye tracking predictor variables + 

I__ = β_0_+ β_1_*F + β_2_*Fd + β_3_*V + β_4_*Sd + β_5_* + β_6_*F*Fd + β_7_*F*V + β_8_*F*Sd + β_9_*F* β_10_*Fd*V+ β_11_*Fd*Sd + β_12_*Fd*Z + β_13_*V*Sd + β_14_*V*Z + β_15_*Sd*

| Variable(s) | Coefficient estimate  ±s.e.m. | *t*-statistic | *p*-value |
| --- | --- | --- | --- |
| F | β_1_ = 0.749 ± 0.243 | 2.875 | 0.0025 |
| Fd | β_2_ = 0.410 ± 0.109 | 3.776 | 0.0002423 |
| V | β_3_ = -0.133 ± 0.222 | -1.505 | 0.548 (ns) |
| Sd | β_4_ = 0.065 ± 0.085 | -0.883 | 0.448(ns) |
|  | β_5_ = 0.169 ± 0.079 | 3.039 | 0.00420 |
| F*Fd | β_6_ = -0.152 ± 0.127 | -0.172 | 0.2305 (ns) |
| F*V | β_7_ = -0.146 ± 0.055 | -0.883 | 0.0093 |
| F*Sd | β_8_ = 0.178± 0.269 | -0.906 | 0.509 (ns) |
| F* | β_9_ = 0.097± 0.218 | 0.736 | 0.658 (ns) |
| Fd*V | β_10_ = 0.080 ± 0.158 | 0.256 | 0.612 (ns) |
| Fd* Sd | β_11_ = 0.037 ± 0.113 | -1.119 | 0.788 (ns) |
| Fd*  | β_12_ - 0.056 ± 0.124 | 0.269 | 0.624 (ns) |
| V*Sd | β_13_ = - 0.193± 0.235 | -0.491 | 0.422 (ns) |
| V*  | β_14_ = -0.102± 0.354 | -0.806 | 0.600 (ns) |
| Sd*  | β_15_ = 0.057 ± 0.086 | -0.526 | 0.506(ns) |
| Intercept | β_0_ = 5.985± 0.093 | 64.097 | <<0.000000001 |

R^2^ = 0.409, adjusted-*R*^2^ = 0.340, *F*(15,129) = 5.948, *p* = 0.00000000290; *MS_e_* = 0.732

**Suppl. Table 4:** Multiple regression model of within-observer normalized (Z-scored) interest rating as a function of all eye tracking predictor variables

I_z_ = β_0_+ β_1_*F + β_2_*Fd + β_3_*V + β_4_*Sd + β_5_*F*Fd + β_6_*F*V + β_7_*F*Sd + β_8_*Fd*V+ β_9_*Fd*Sd+ β_10_*V*Sd

| Variable(s) | Coefficient estimate  ±s.e.m. | *t*-statistic | *p*-value |
| --- | --- | --- | --- |
| F | β_1_ = 0.371 ± 0.074 | 5.048 | 0.0000013734 |
| Fd | β_2_ = 0.151 ± 0.042 | 3.545 | 0.00053562 |
| V | β_3_ = -0.069± 0.070 | -0.991 | 0.3524(ns) |
| Sd | β_4_ = 0.022± 0.036 | 0.607 | 0.545(ns) |
| F*Fd | β_5_ = 0.009± 0.055 | 0.158 | 0.874(ns) |
| F*V | β_6_ = -0.038±0.024 | -1.596 | 0.112 (ns) |
| F*Sd | β_7_ = 0.174± 0.109 | 1.590 | 0.114 (ns) |
| Fd*V | β_8_ = -0.018± 0.060 | -0.314 | 0.753 (ns) |
| Fd* Sd | β_9_ = 0.022±0.048 | 0.467 | 0.641 (ns) |
| V*Sd | β_10_ = -0.186±0.105 | -1.765 | 0.080 (ns) |
| Intercept | β_0_ = 0.026± 0.043 | 0.612 | 0.542 (ns) |

R^2^ = 0.377, adjusted-*R*^2^ = 0.332, *F*(10,139) = 8.399, *p* = 0.000000000134; *MS_e_* = 0.164

**Suppl. Table 5:** Reduced regression model of within-observer normalized (Z-scored) interest rating (I_z_) as a function of number of fixations (F) and fixation duration (Fd)

I_z_ = β_1_*F + β_2_*Fd + β_3_*F*Fd

| Variable(s) | Coefficient estimate  ±s.e.m. | *t*-statistic | *p*-value |
| --- | --- | --- | --- |
| F | β_1_ = 0.297 ±0.035 | 8.370 | 0.0000000000000430 |
| Fd | β_2_ = 0.132 ± 0.035 | 3.777 | 0.000231 |
| F*Fd | β_3_ = -0.000 ± 0.030 | -0.004 | 0.996(ns) |
| intercept | β_0_ = -0.000 ± 0.034 | -0.001 | 0.999 (ns) |

R^2^ = 0.338, adjusted-*R*^2^ = 0.324, *F*(3,146) = 24.858, *p* = 0.000000000000473; *MS_e_* = 0.166

**Suppl. Table 6:** Multiple regression model of within-observer minmax normalized interest rating (I_mm_) as a function of all eye tracking predictor variables

I_mm_ = β_0_+ β_1_*F + β_2_*Fd + β_3_*V + β_4_*Sd + β_5_*F*Fd + β_6_*F*V + β_7_*F*Sd + β_8_*Fd*V+ β_9_*Fd*Sd+ β_10_*V*Sd

| Variable(s) | Coefficient estimate  ±s.e.m. | *t*-statistic | *p*-value |
| --- | --- | --- | --- |
| F | β_1_ = 0.087 ± 0.021 | 4.247 | 0.00003947 |
| Fd | β_2_ = 0.035 ± 0.012 | 2.959 | 0.036 |
| V | β_3_ = -0.005 ± 0.020 | -0.258 | 0.797(ns) |
| Sd | β_4_ = 0.004 ± 0.010 | 0.414 | 0.679 (ns) |
| F*Fd | β_5_ = -0.007 ± 0.015 | -0.442 | 0.659 (ns) |
| F*V | β_6_ = -0.016 ± 0.007 | -2.339 | 0.021 |
| F*Sd | β_7_ = 0.050 ± 0.029 | 1.711 | 0.089 (ns) |
| Fd*V | β_8_ = -0.006 ± 0.017 | -0.349 | 0.728(ns) |
| Fd* Sd | β_9_ 0.008 ± 0.014 | 0.554 | 0.581 (ns) |
| V*Sd | β_10_ =-0.057 ± 0.028 | -2.020 | 0.045 |
| Intercept | β_0_ = 0.527 ±0.012 | 42.891 | <<0.000000001 |

R^2^ = 0.378, adjusted-*R*^2^ = 0.334, *F*(10,139) = 8.462, *p* = 0.000000000112 *MS_e_* = 0.013

**Suppl. Table 7:** Reduced regression model of within-observer minmax normalized interest rating (I_mm_) as a function of number of fixations (F) and fixation duration (Fd)

I_mm_ = β_1_*F + β_2_*Fd + β_3_*F*Fd (intercept is at the bottom, fixation at the top)

| Variable(s) | Coefficient estimate  ±s.e.m. | *t*-statistic | *p*-value |
| --- | --- | --- | --- |
| F | β_1_ = 0.081± 0.010 | 7.957 | 0.000000000000448 |
| Fd | β_2_ = 0.036± 0.010 | 3.578 | 0.000470 |
| F*Fd | β_3_ = -0.007± 0.009 | -0.762 | 0.447 (ns) |
| intercept | β_0_ = 0.516 ±0.010 | 51.988 | <<0.000000001 |

R^2^ = 0.324, adjusted-*R*^2^ = 0.310, *F*(3,146) = 23.332, *p* = 0.00000000000214; *MS_e_* = 0.014

In a preliminary analysis, we examined if other features related to small eye movements, i.e. microsaccade rate, drift rate, and pupil size could help account for image interestingness. Epochs for each image were extracted beginning from 0.5 seconds before the onset of the image until when the observer moved on to the next image on the screen in the self-paced Experiment 1. The epoch period varied across trials and individuals and time in which each participant viewed the image varied drastically; therefore, we sliced the epochs into fixed time periods for ease of analysis: -0.5s-1s, -0.5s-5s, -0.5s-10s. The sampling frequency was 400 Hz. Custom code was written in the new toolbox. We used this toolbox to measure pupil diameter (camera pixels), micro saccades (1/second) and drift speed (deg/sec). The results thus far have not yielded significant differences amongst any of the different image categories. However, further analysis is ongoing with studies on hippus, i.e. spectral analysis of change in pupil diameter.
