# Supplementary figures and images for: What Makes an Image Interesting and How Can We Explain It (part 2 of 3)
Source: Front Psychol. 2021 Sep 1;12:668651. doi: 10.3389/fpsyg.2021.668651 (PMC8440840; doi:10.3389/fpsyg.2021.668651)

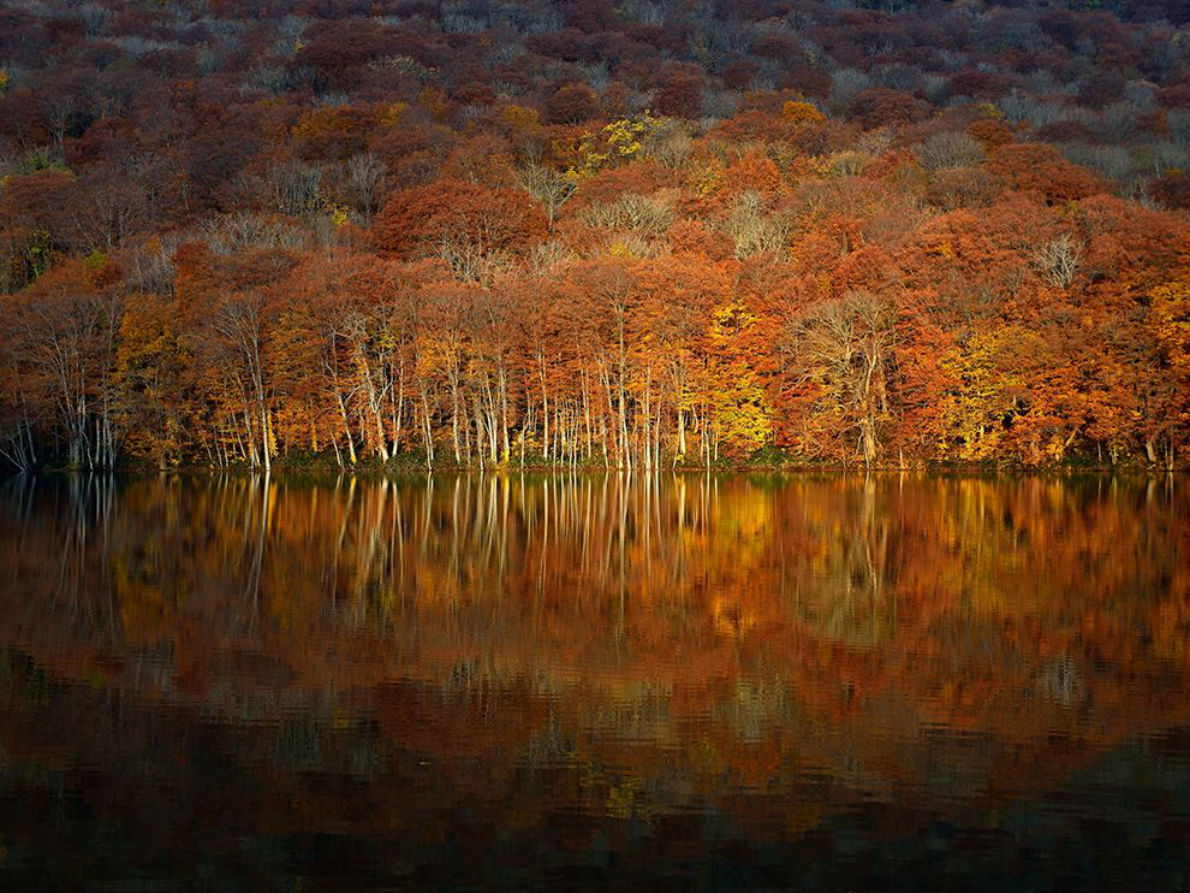

Supplement: Supplementary file 2 [file Data_Sheet_1.zip › Raw Images for Experiment 1/Landscapes/ls1.jpg]

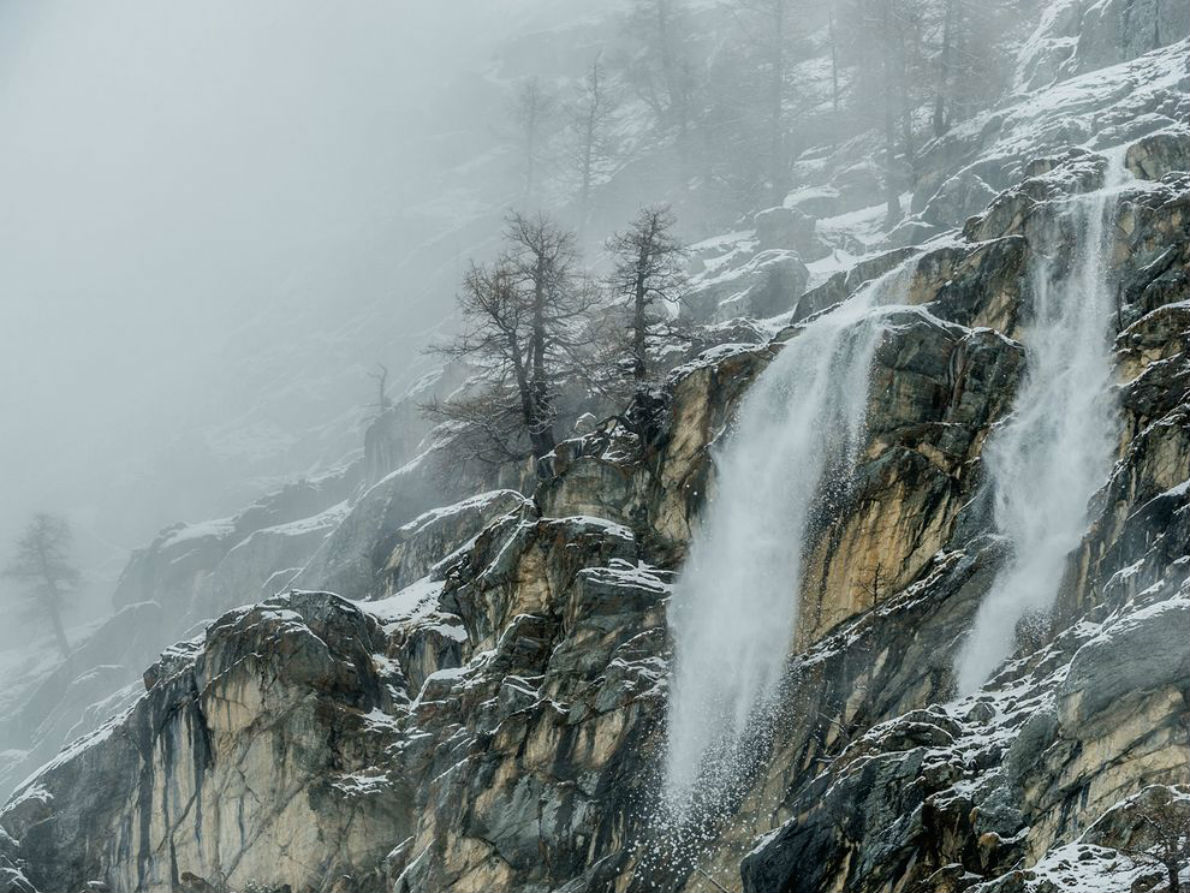

Supplement: Supplementary file 2 [file Data_Sheet_1.zip › Raw Images for Experiment 1/Landscapes/ls10.jpg]

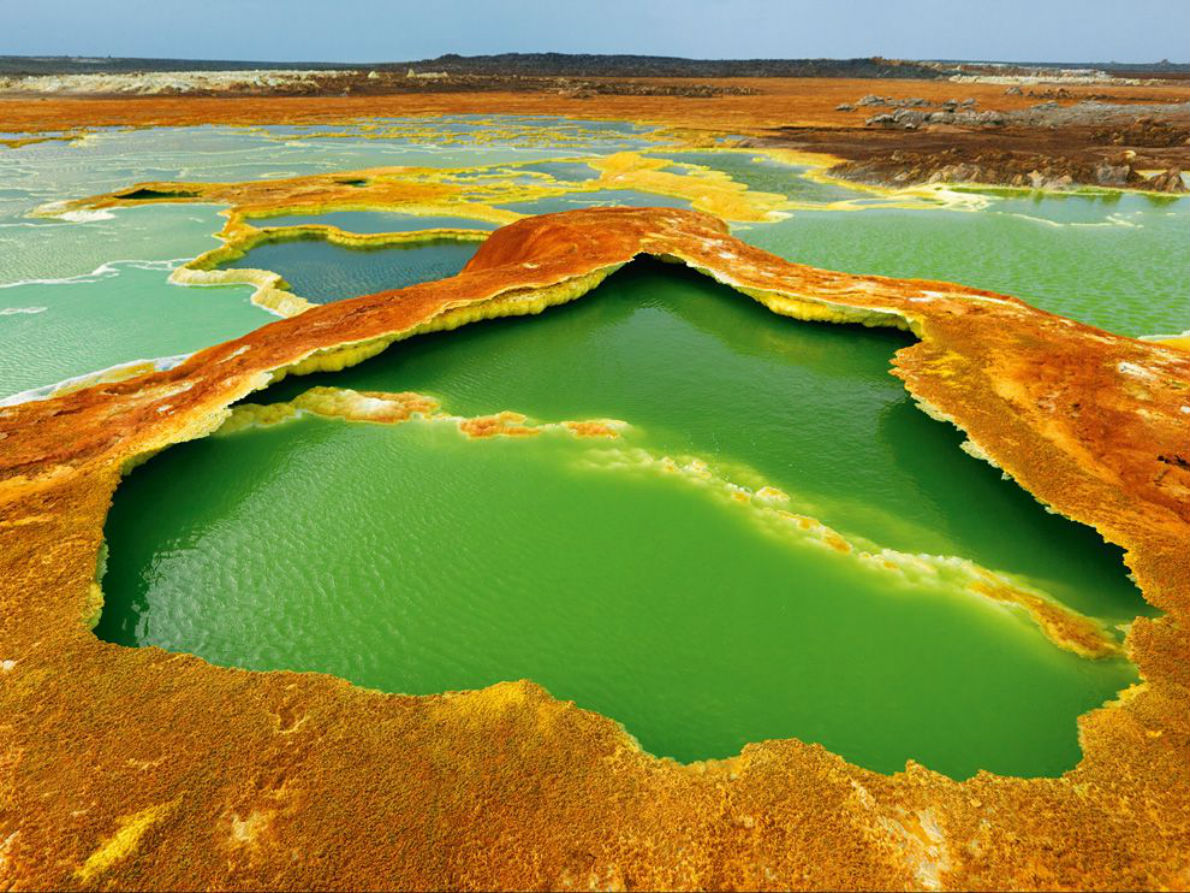

Supplement: Supplementary file 2 [file Data_Sheet_1.zip › Raw Images for Experiment 1/Landscapes/ls11.jpg]

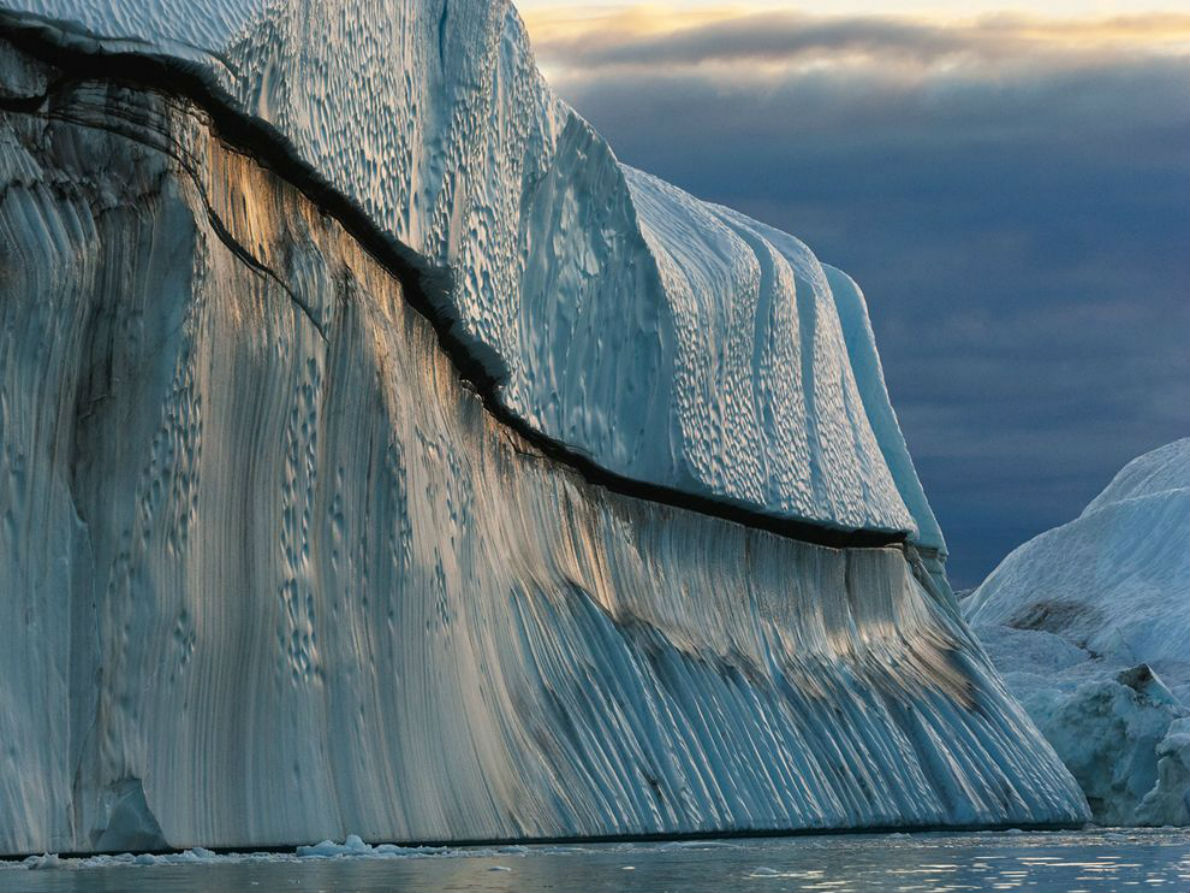

Supplement: Supplementary file 2 [file Data_Sheet_1.zip › Raw Images for Experiment 1/Landscapes/ls12.jpg]

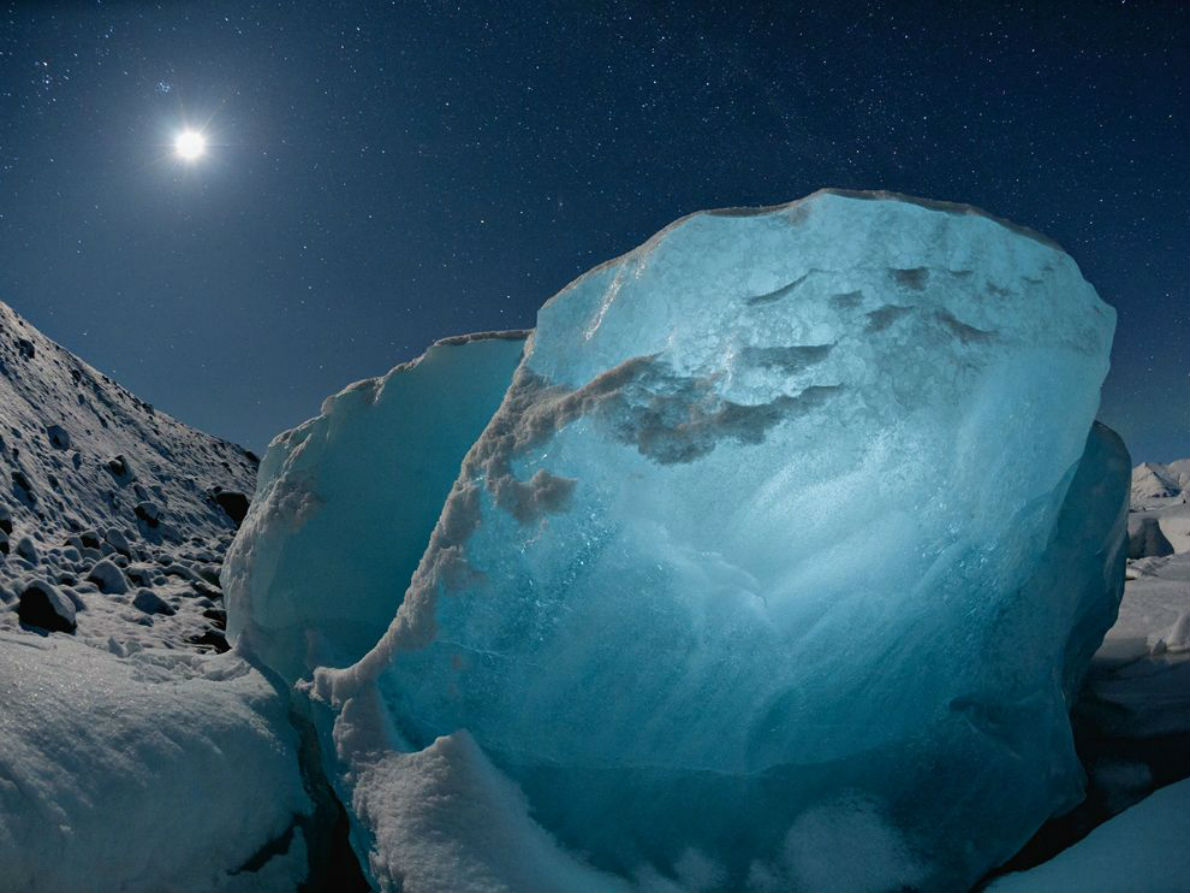

Supplement: Supplementary file 2 [file Data_Sheet_1.zip › Raw Images for Experiment 1/Landscapes/ls13.jpg]

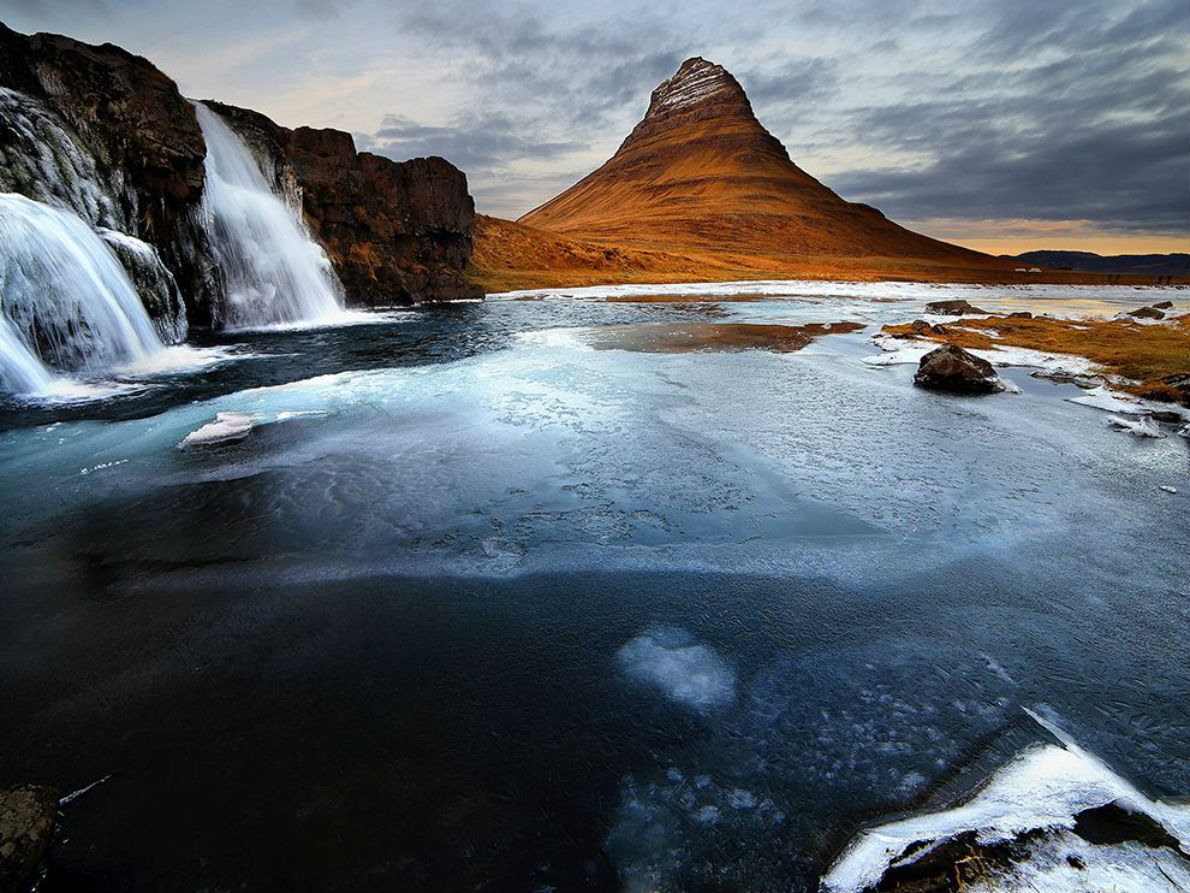

Supplement: Supplementary file 2 [file Data_Sheet_1.zip › Raw Images for Experiment 1/Landscapes/ls14.jpg]

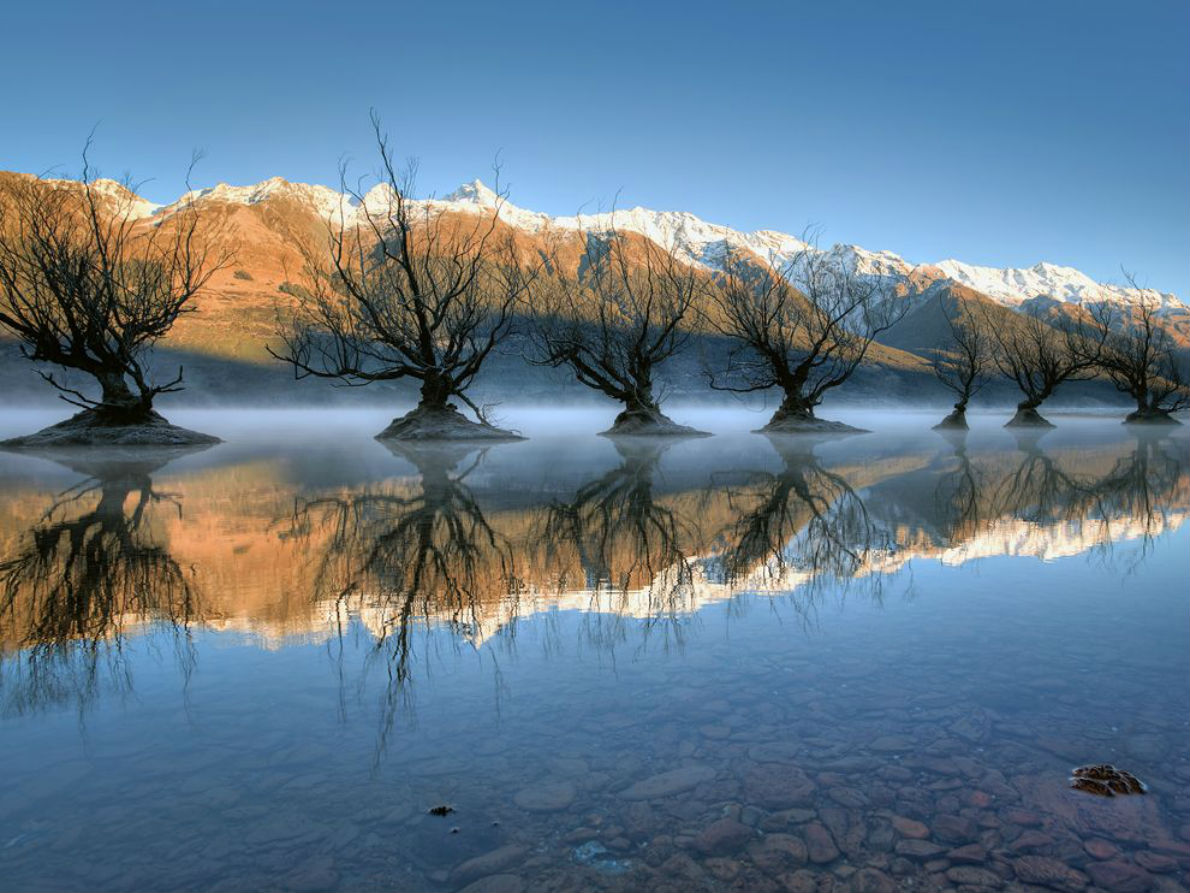

Supplement: Supplementary file 2 [file Data_Sheet_1.zip › Raw Images for Experiment 1/Landscapes/ls15.jpg]

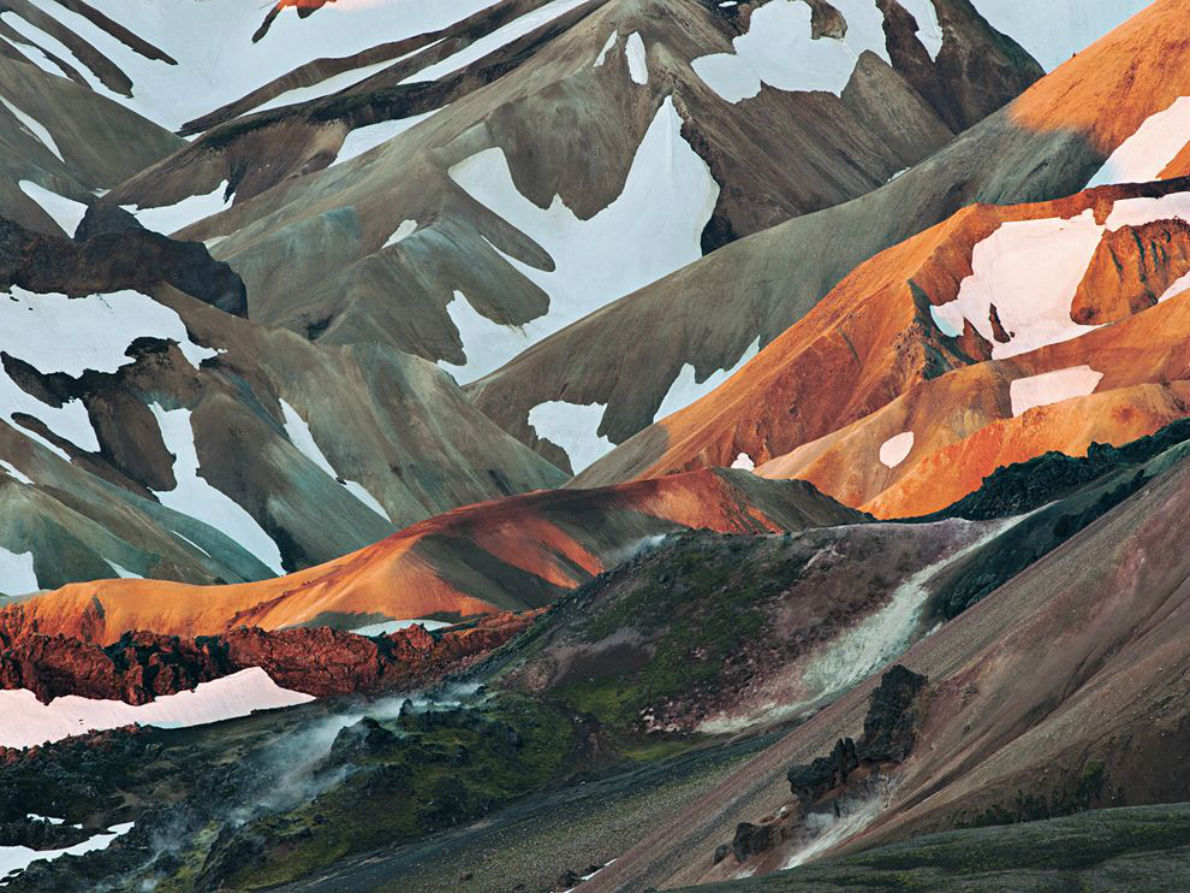

Supplement: Supplementary file 2 [file Data_Sheet_1.zip › Raw Images for Experiment 1/Landscapes/ls16.jpg]

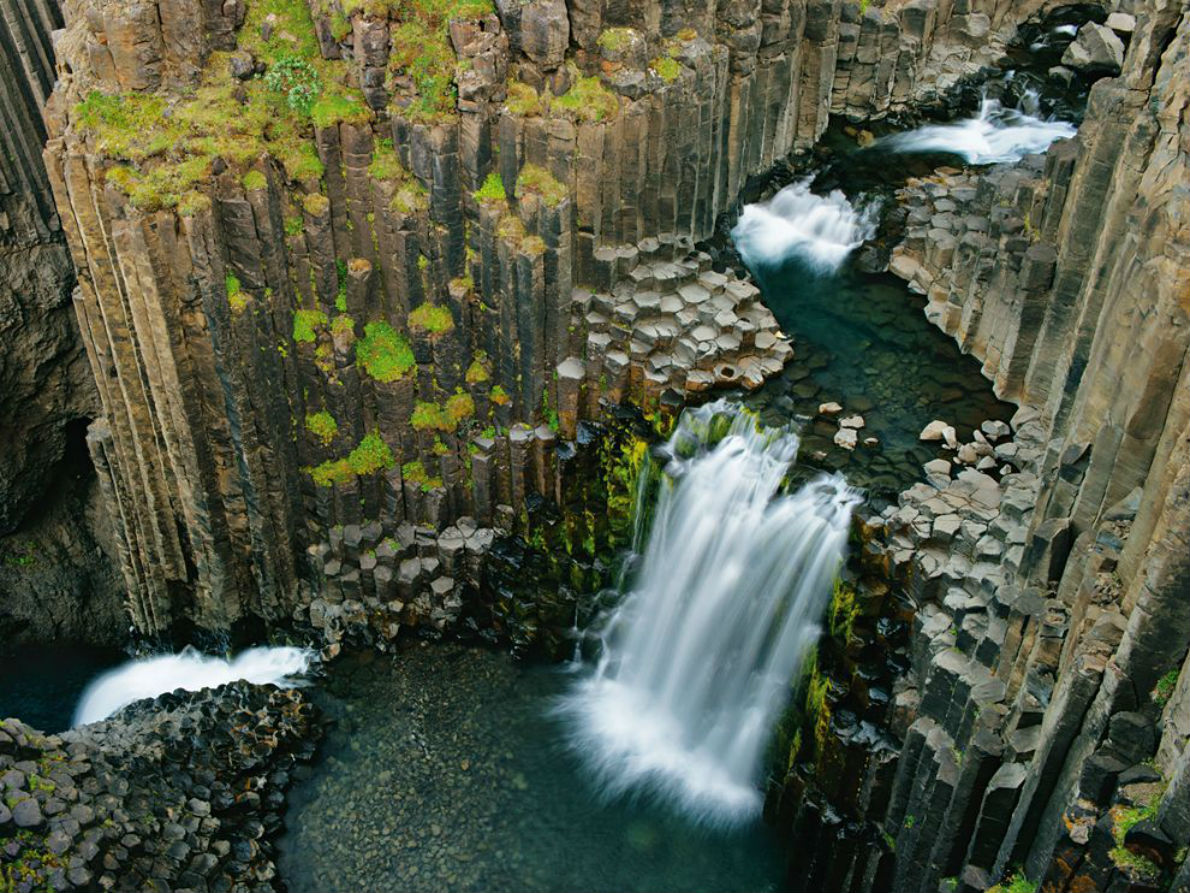

Supplement: Supplementary file 2 [file Data_Sheet_1.zip › Raw Images for Experiment 1/Landscapes/ls17.jpg]

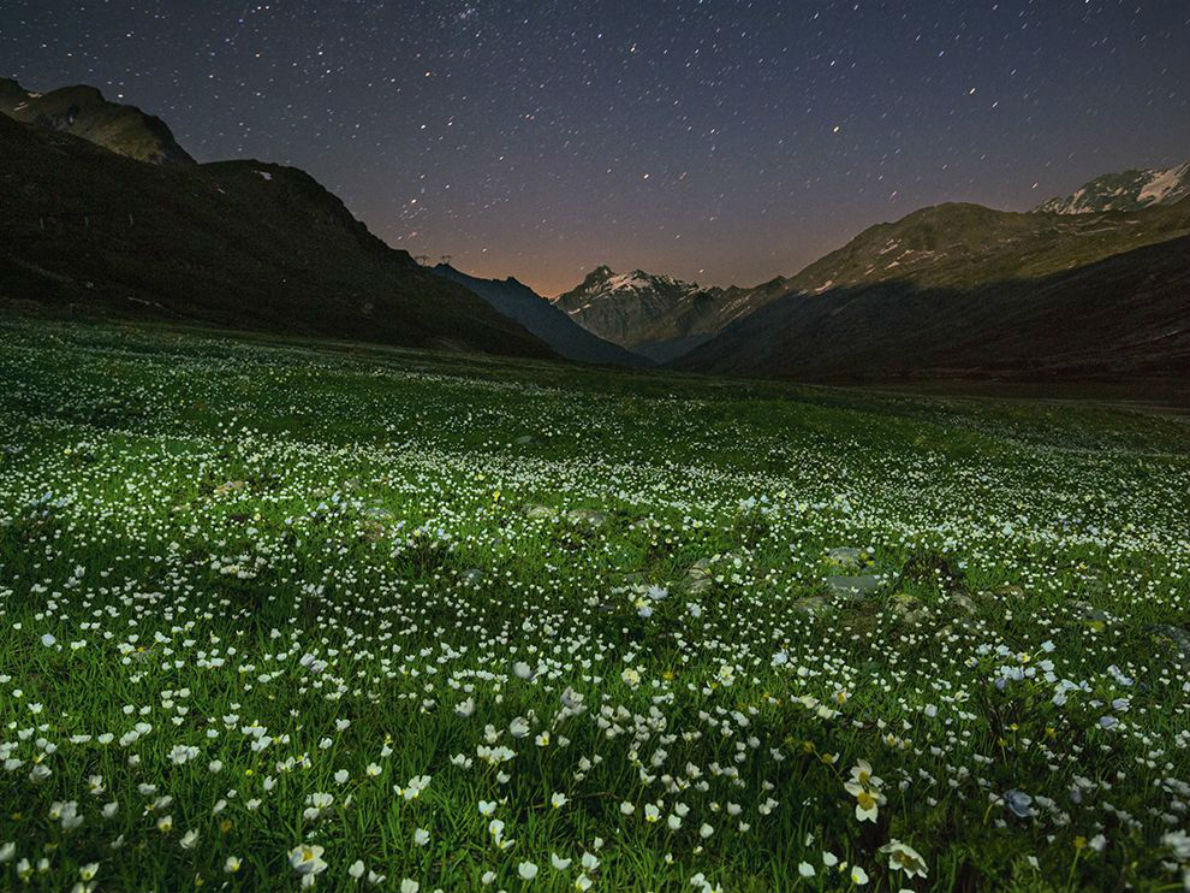

Supplement: Supplementary file 2 [file Data_Sheet_1.zip › Raw Images for Experiment 1/Landscapes/ls18.jpg]

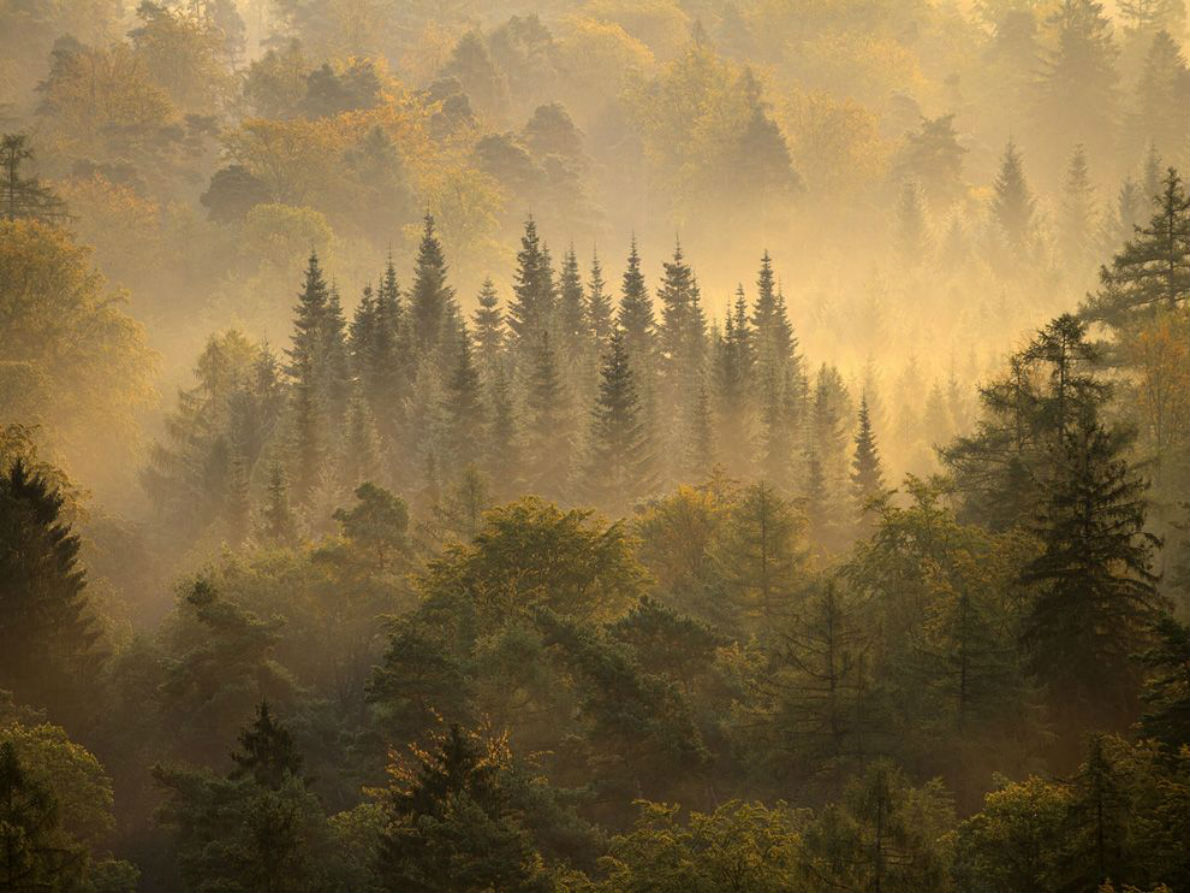

Supplement: Supplementary file 2 [file Data_Sheet_1.zip › Raw Images for Experiment 1/Landscapes/ls19.jpg]

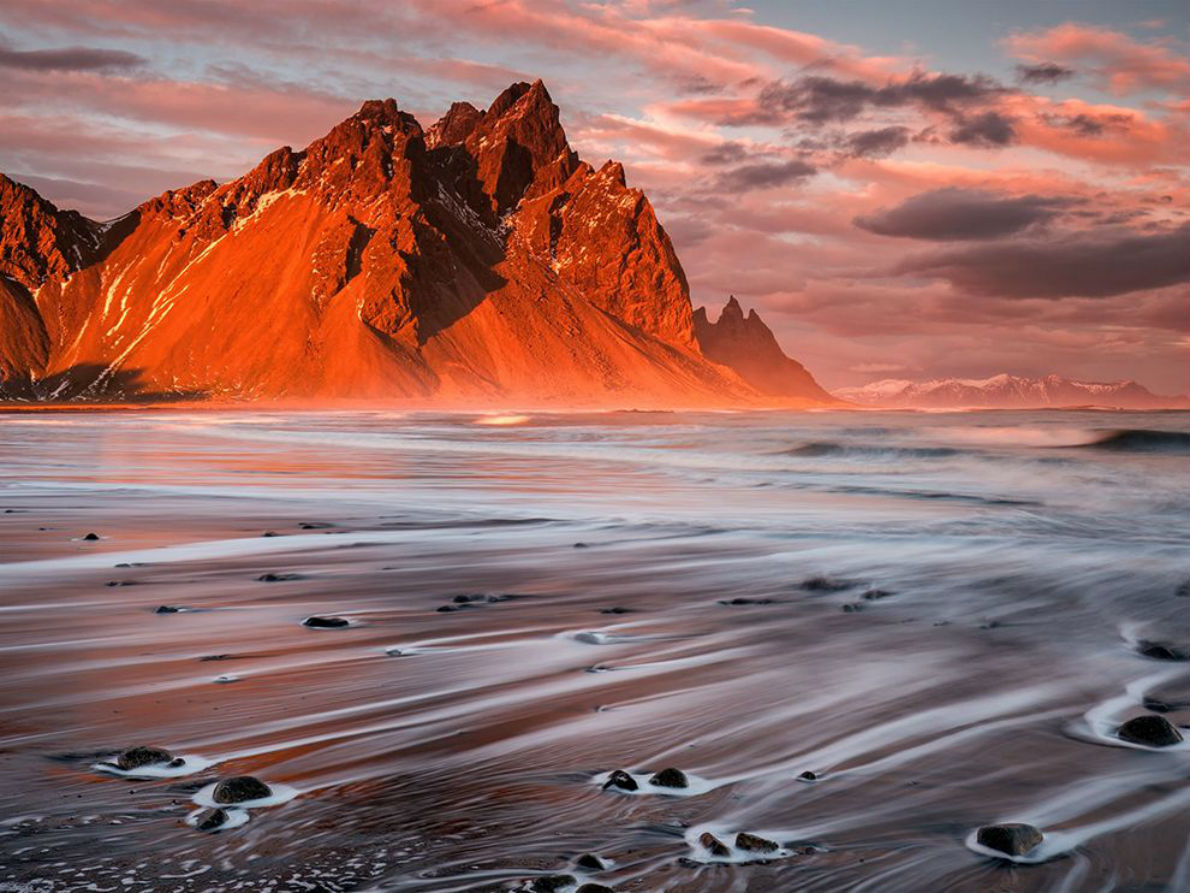

Supplement: Supplementary file 2 [file Data_Sheet_1.zip › Raw Images for Experiment 1/Landscapes/ls2.jpg]

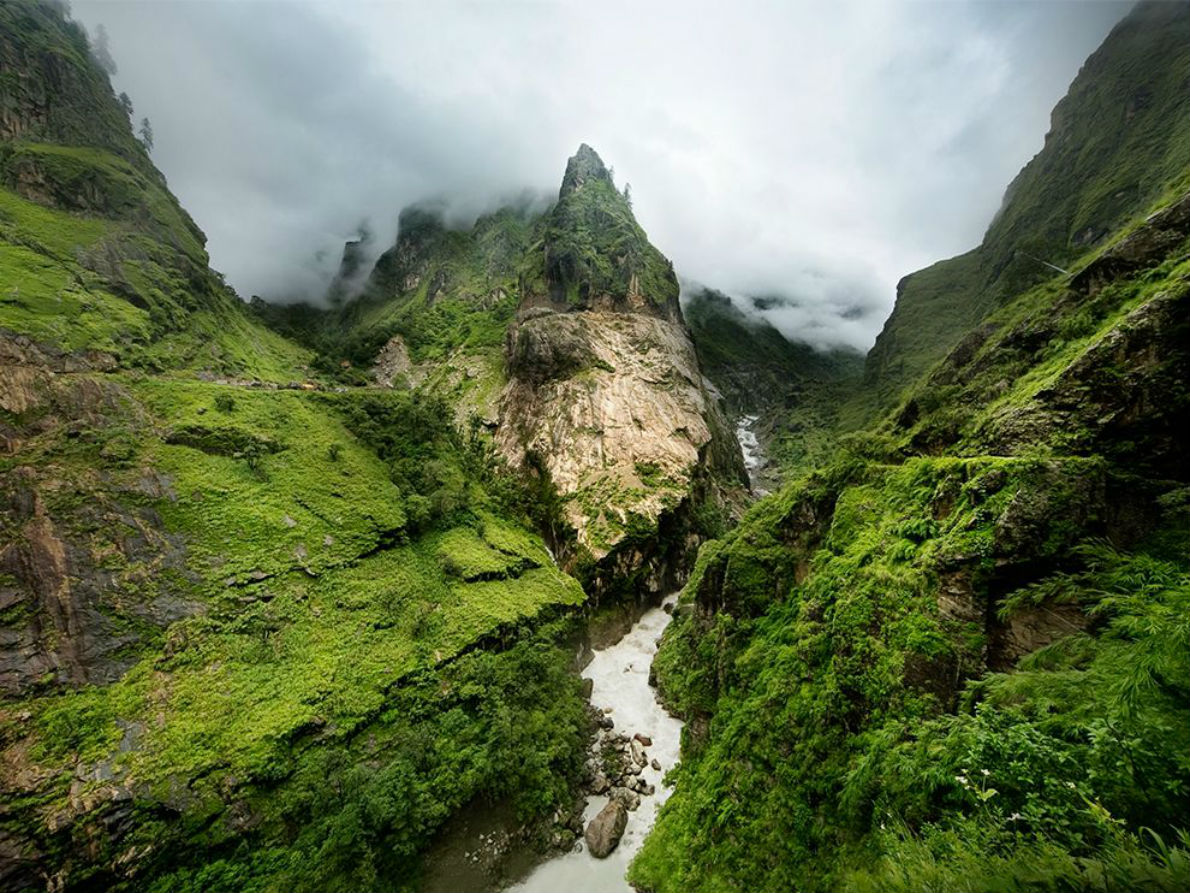

Supplement: Supplementary file 2 [file Data_Sheet_1.zip › Raw Images for Experiment 1/Landscapes/ls20.jpg]

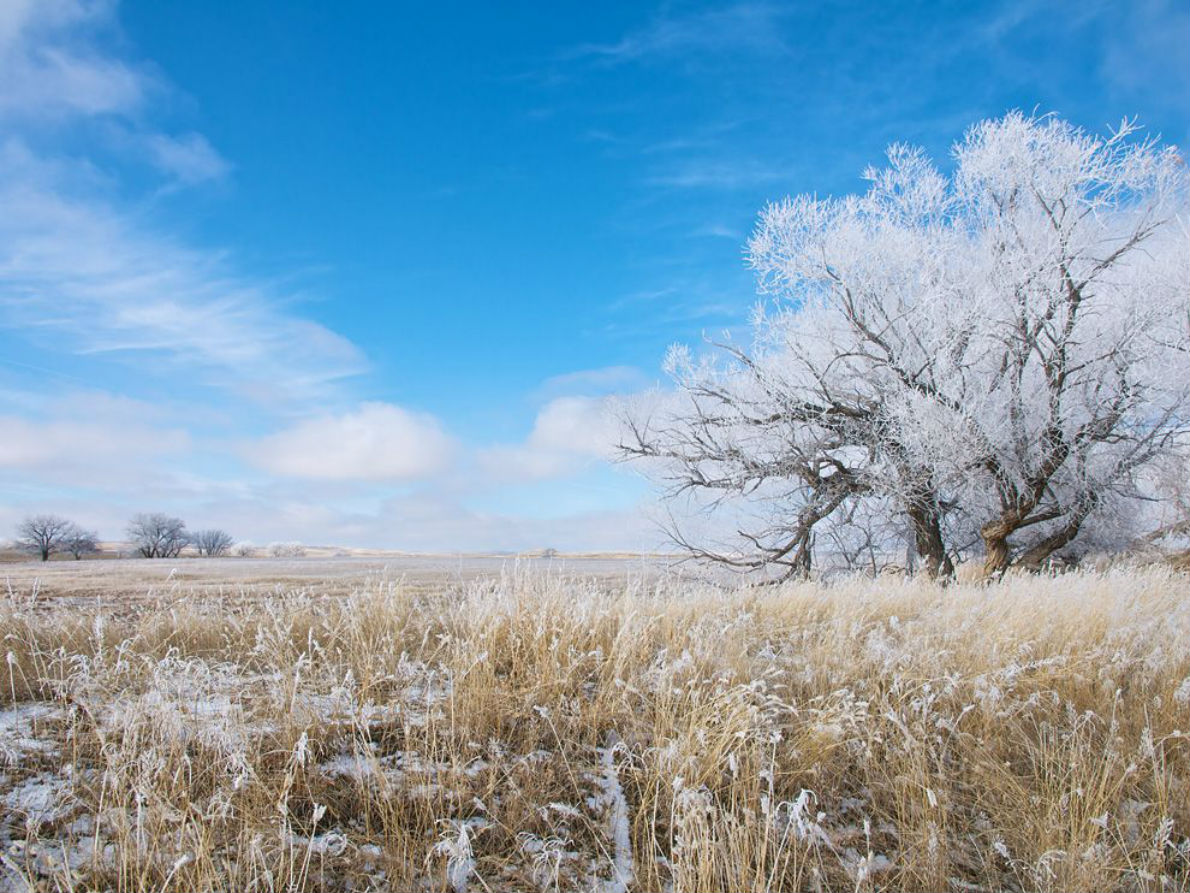

Supplement: Supplementary file 2 [file Data_Sheet_1.zip › Raw Images for Experiment 1/Landscapes/ls21.jpg]

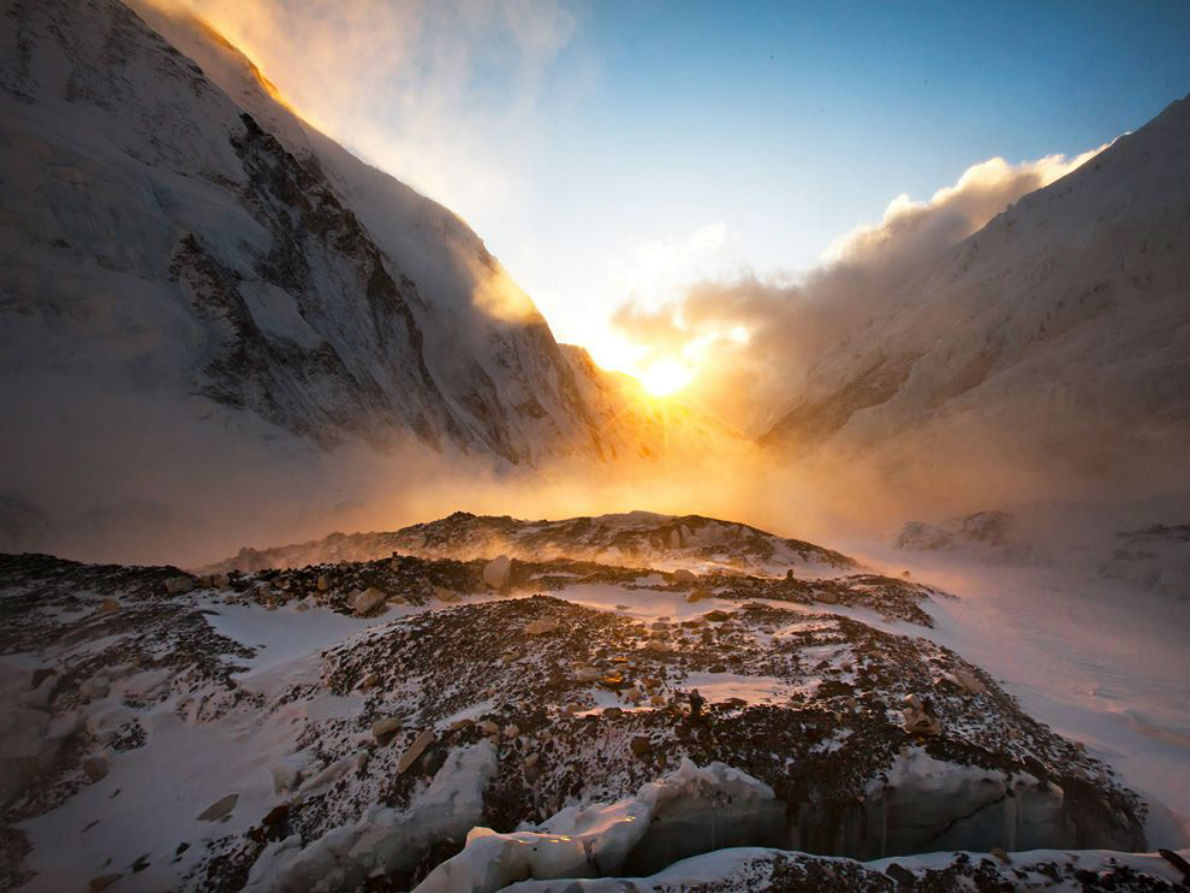

Supplement: Supplementary file 2 [file Data_Sheet_1.zip › Raw Images for Experiment 1/Landscapes/ls22.jpg]

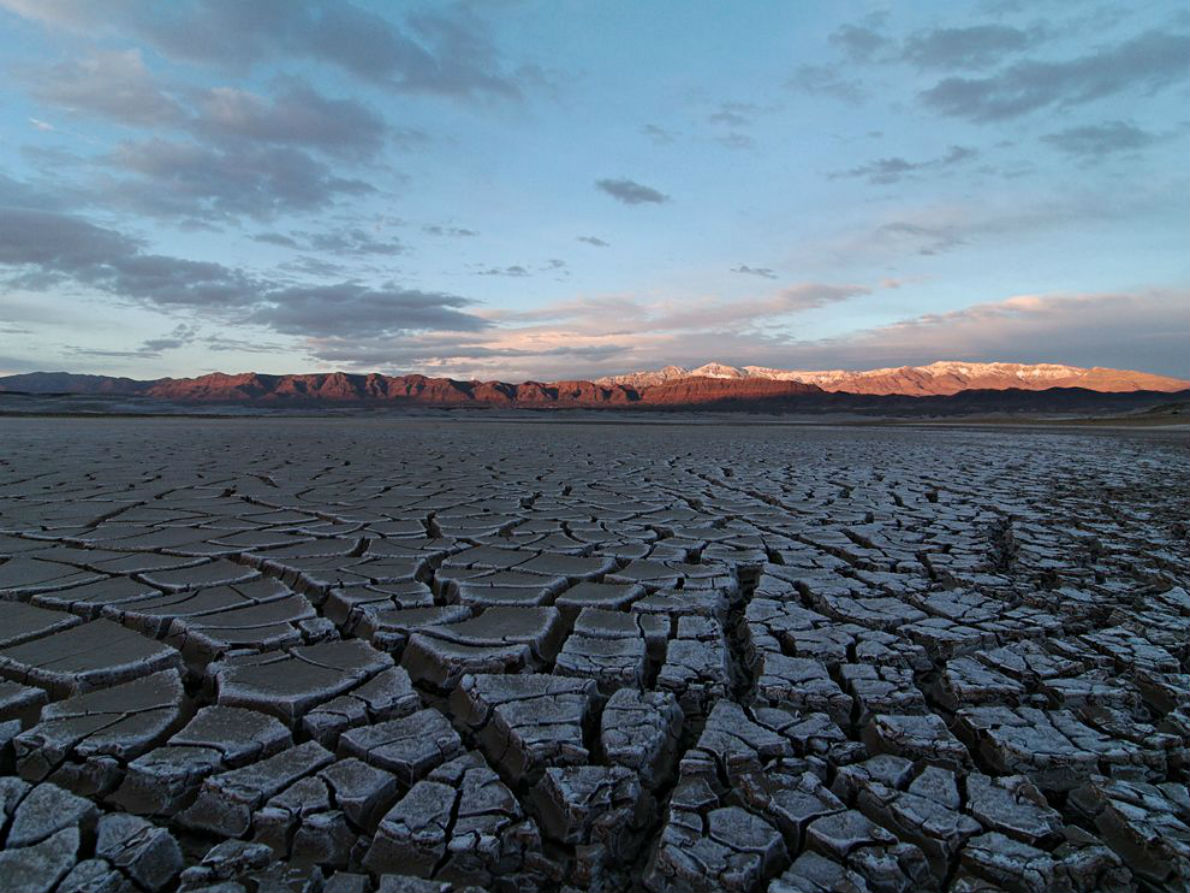

Supplement: Supplementary file 2 [file Data_Sheet_1.zip › Raw Images for Experiment 1/Landscapes/ls23.jpg]

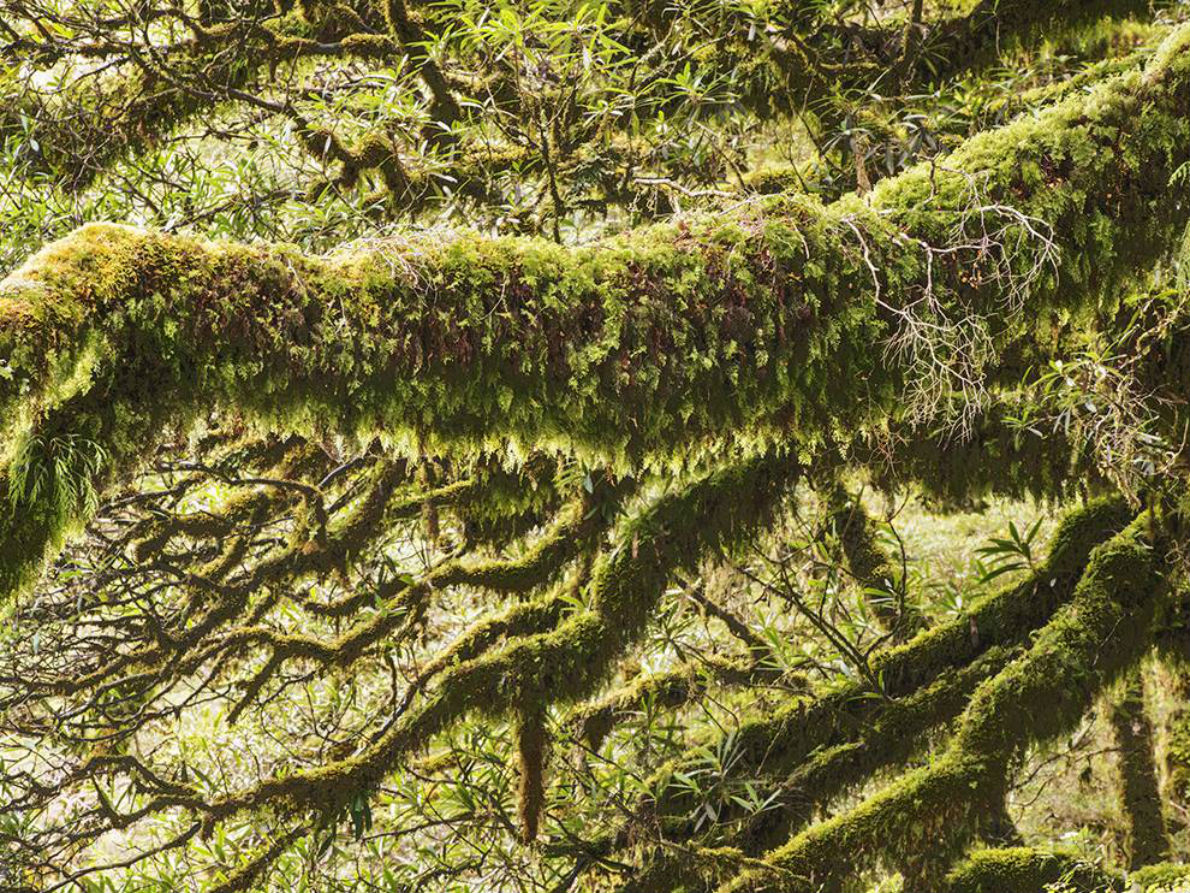

Supplement: Supplementary file 2 [file Data_Sheet_1.zip › Raw Images for Experiment 1/Landscapes/ls24.jpg]

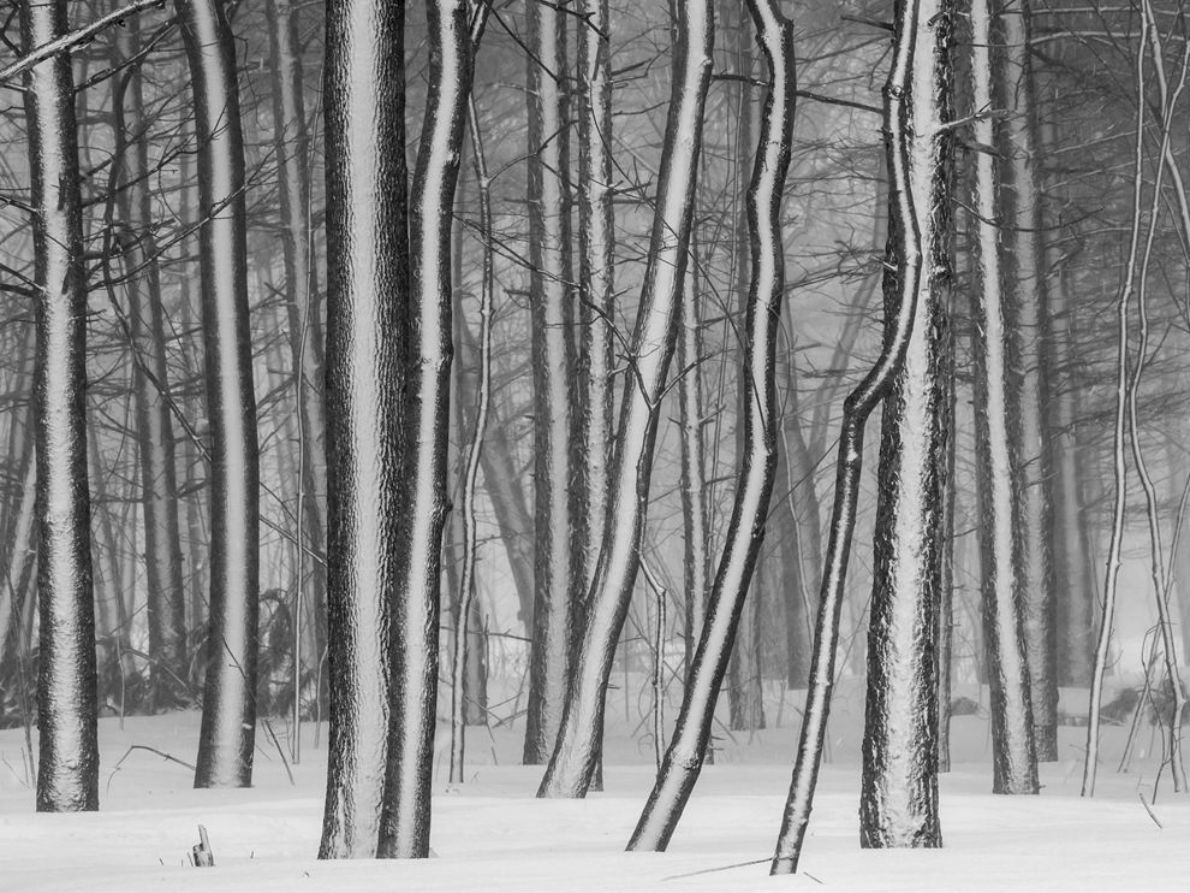

Supplement: Supplementary file 2 [file Data_Sheet_1.zip › Raw Images for Experiment 1/Landscapes/ls25.jpg]

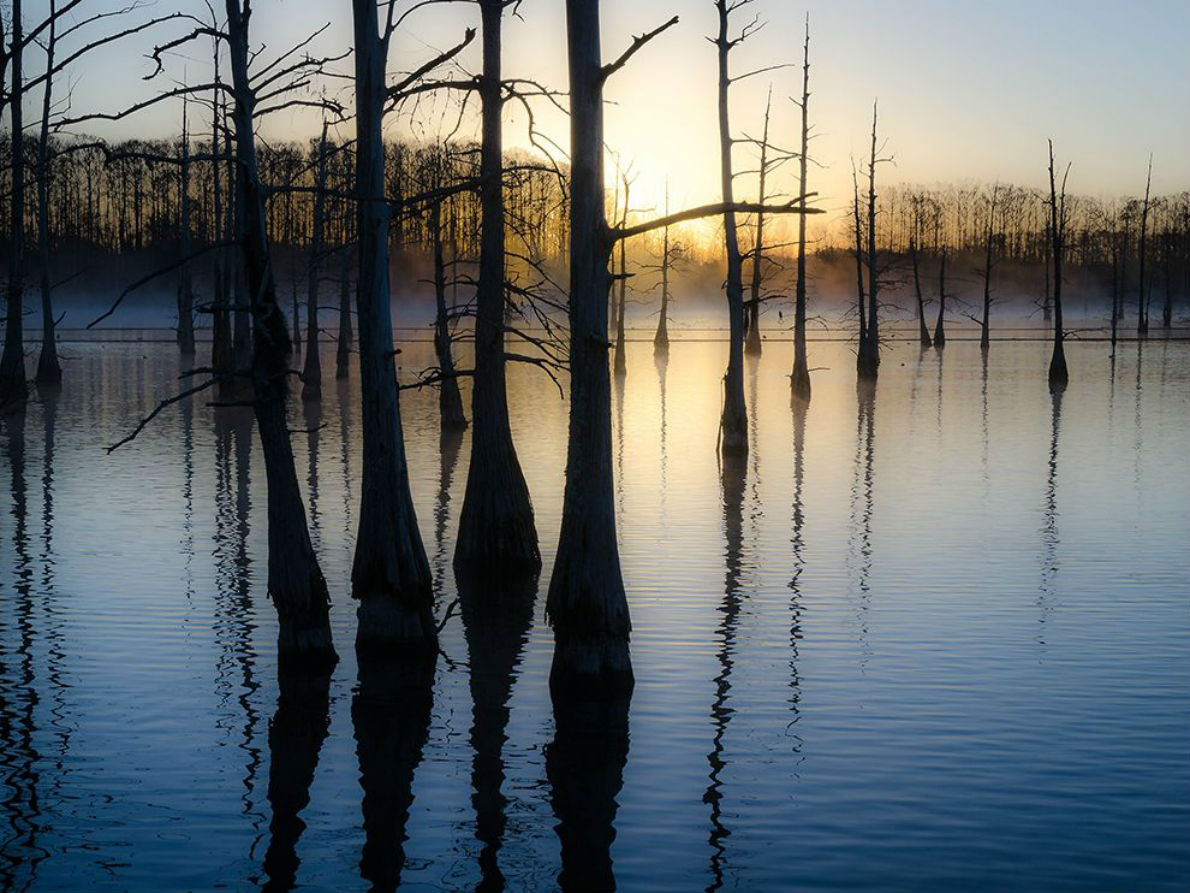

Supplement: Supplementary file 2 [file Data_Sheet_1.zip › Raw Images for Experiment 1/Landscapes/ls3.jpg]

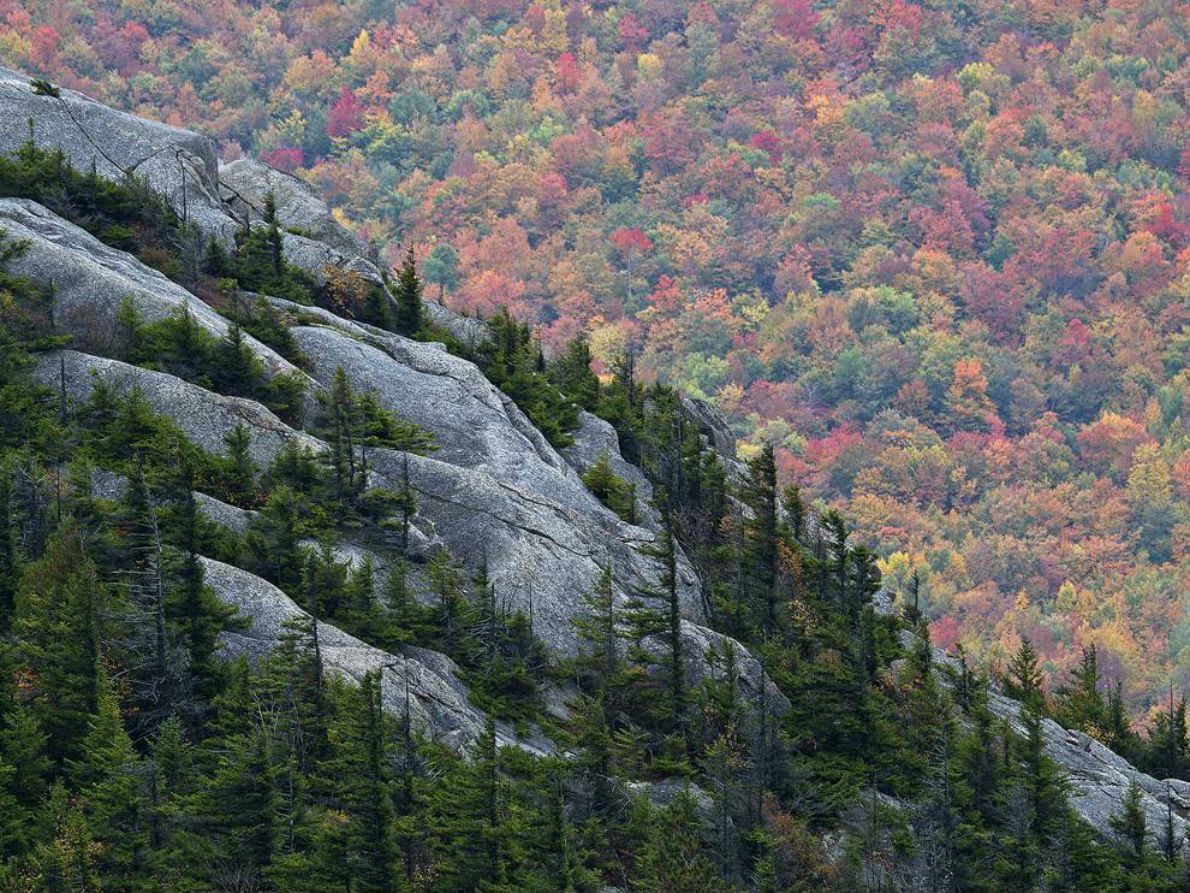

Supplement: Supplementary file 2 [file Data_Sheet_1.zip › Raw Images for Experiment 1/Landscapes/ls4.jpg]

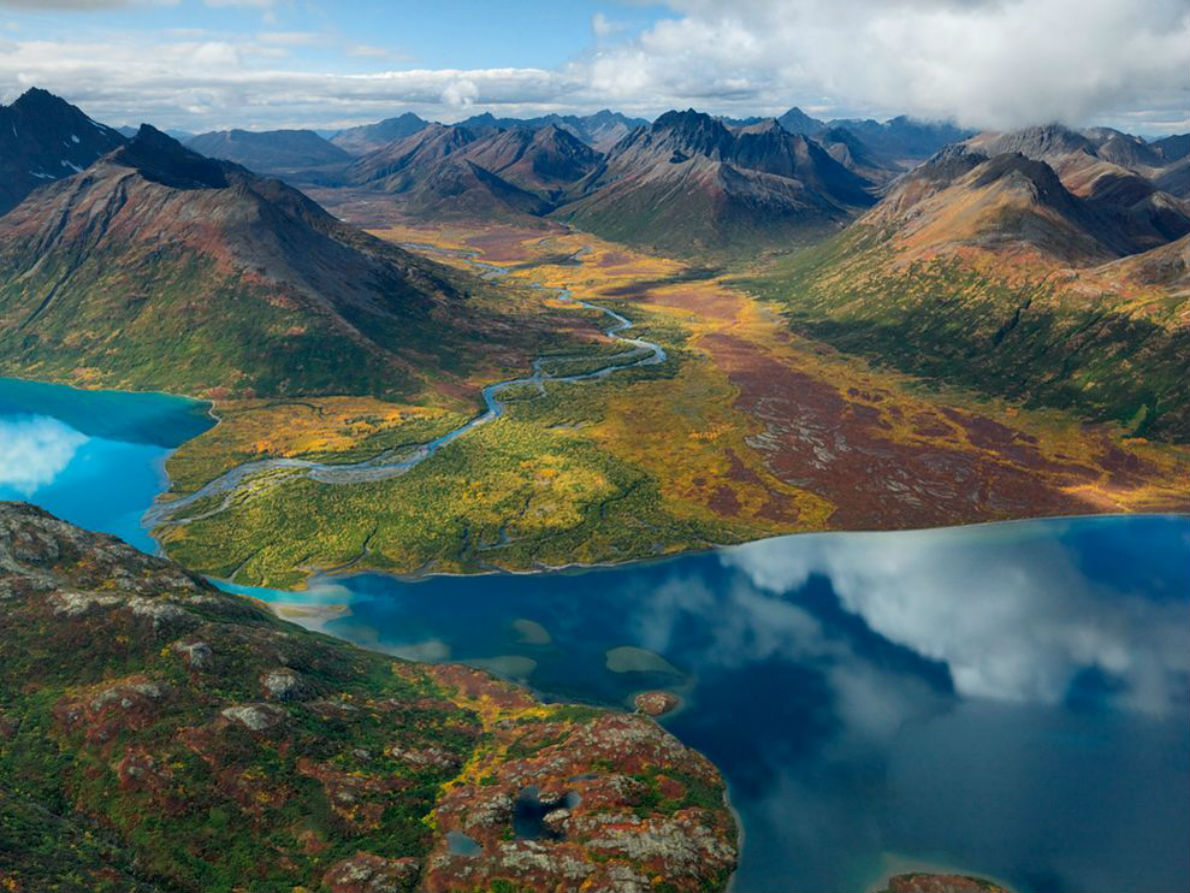

Supplement: Supplementary file 2 [file Data_Sheet_1.zip › Raw Images for Experiment 1/Landscapes/ls5.jpg]

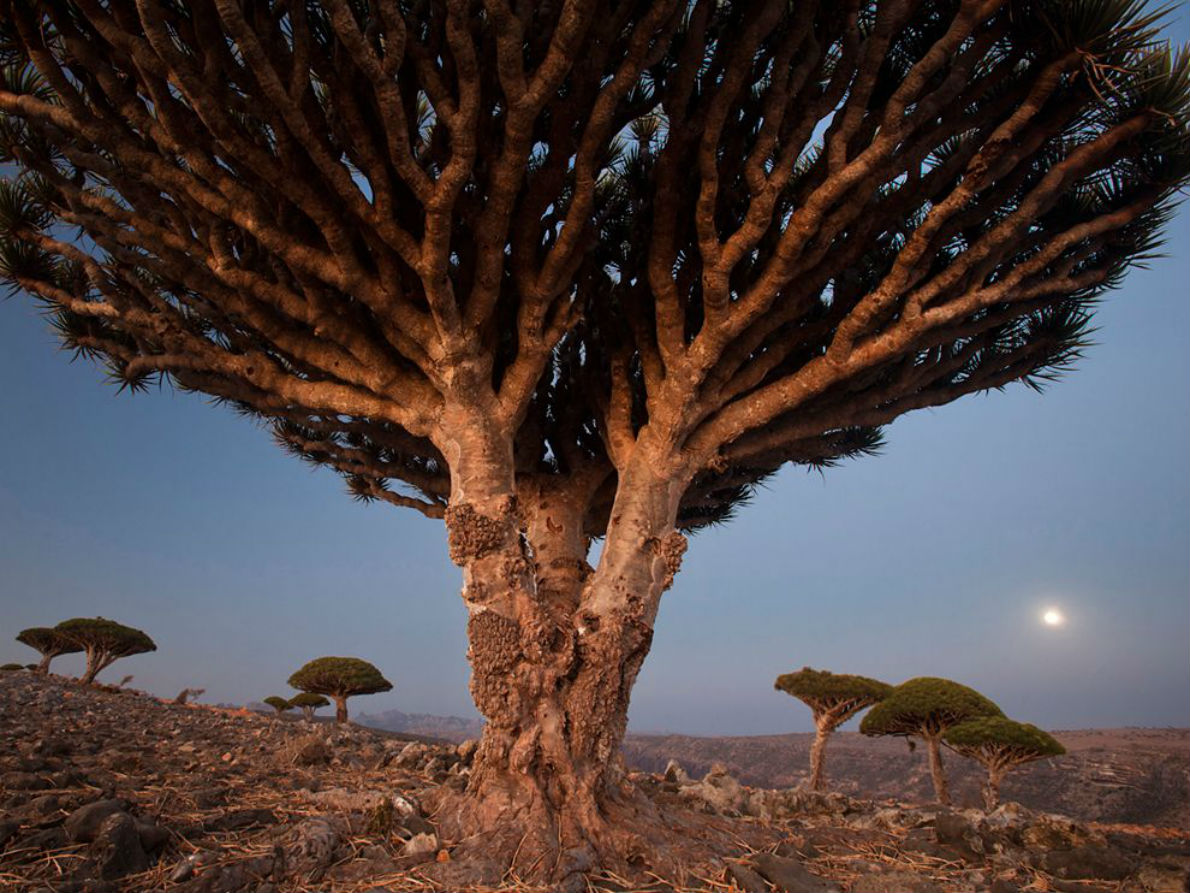

Supplement: Supplementary file 2 [file Data_Sheet_1.zip › Raw Images for Experiment 1/Landscapes/ls6.jpg]

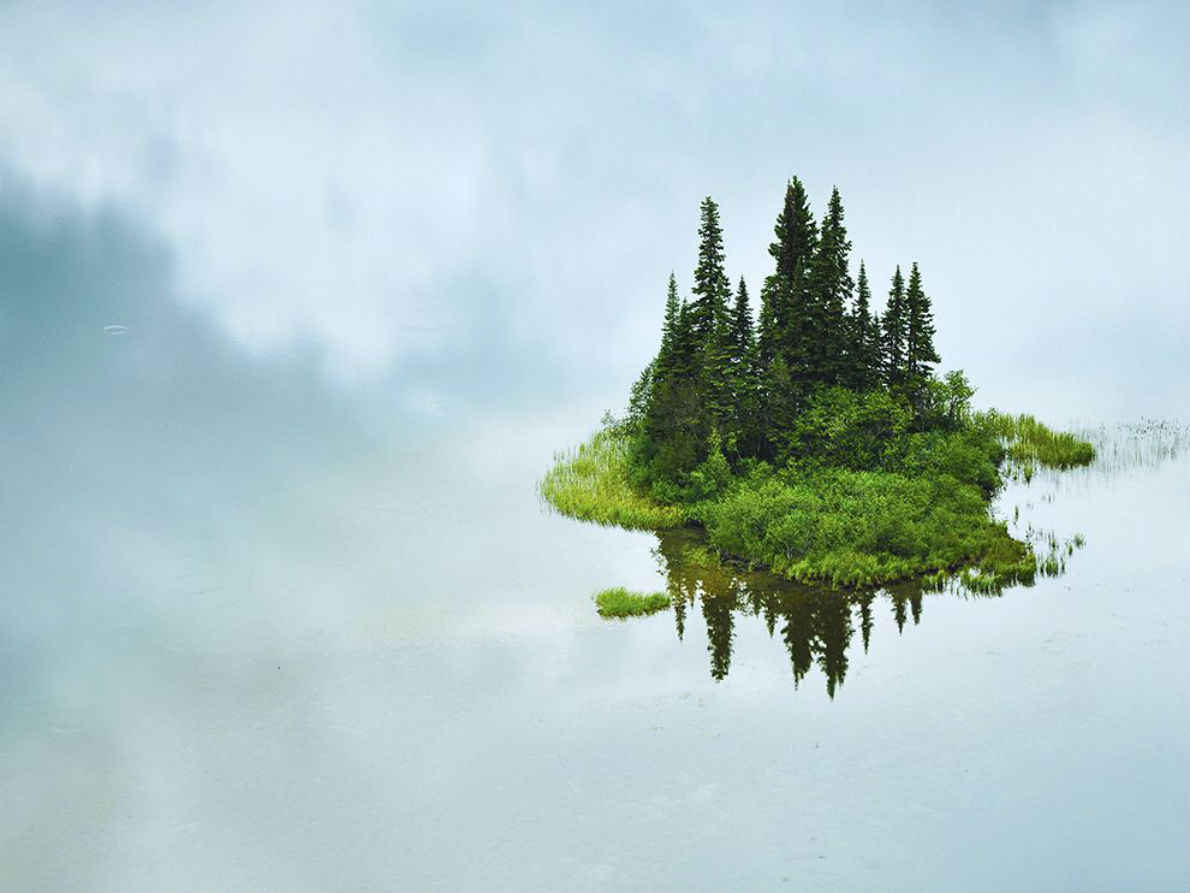

Supplement: Supplementary file 2 [file Data_Sheet_1.zip › Raw Images for Experiment 1/Landscapes/ls7.jpg]

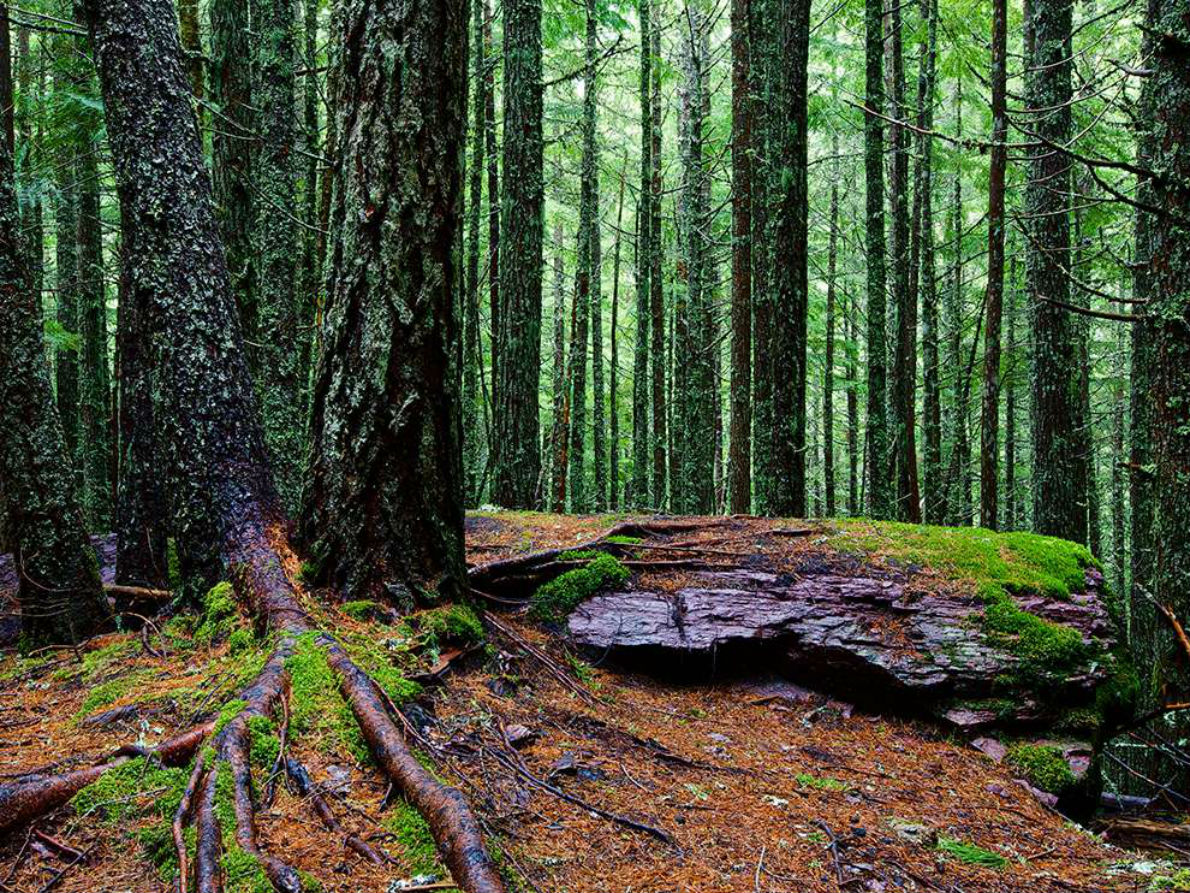

Supplement: Supplementary file 2 [file Data_Sheet_1.zip › Raw Images for Experiment 1/Landscapes/ls8.jpg]

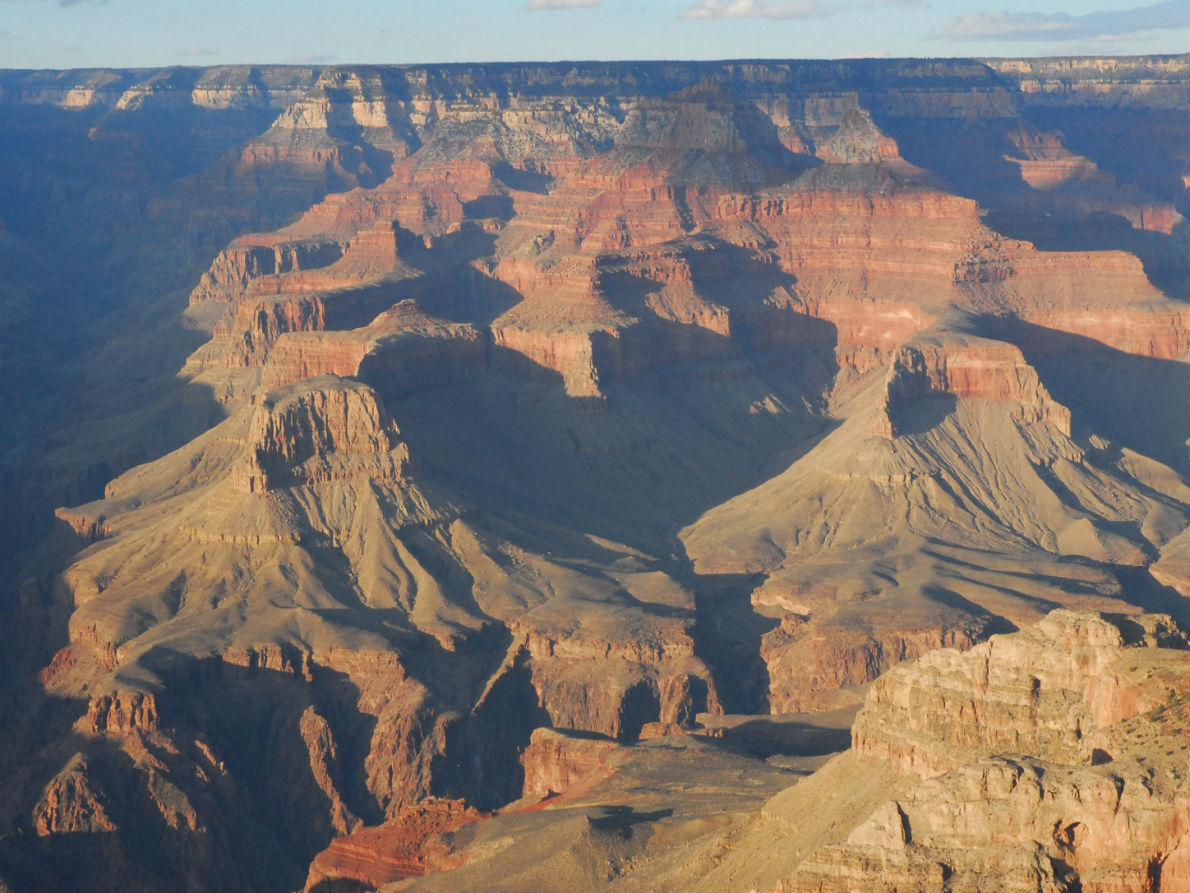

Supplement: Supplementary file 2 [file Data_Sheet_1.zip › Raw Images for Experiment 1/Landscapes/ls9.jpg]

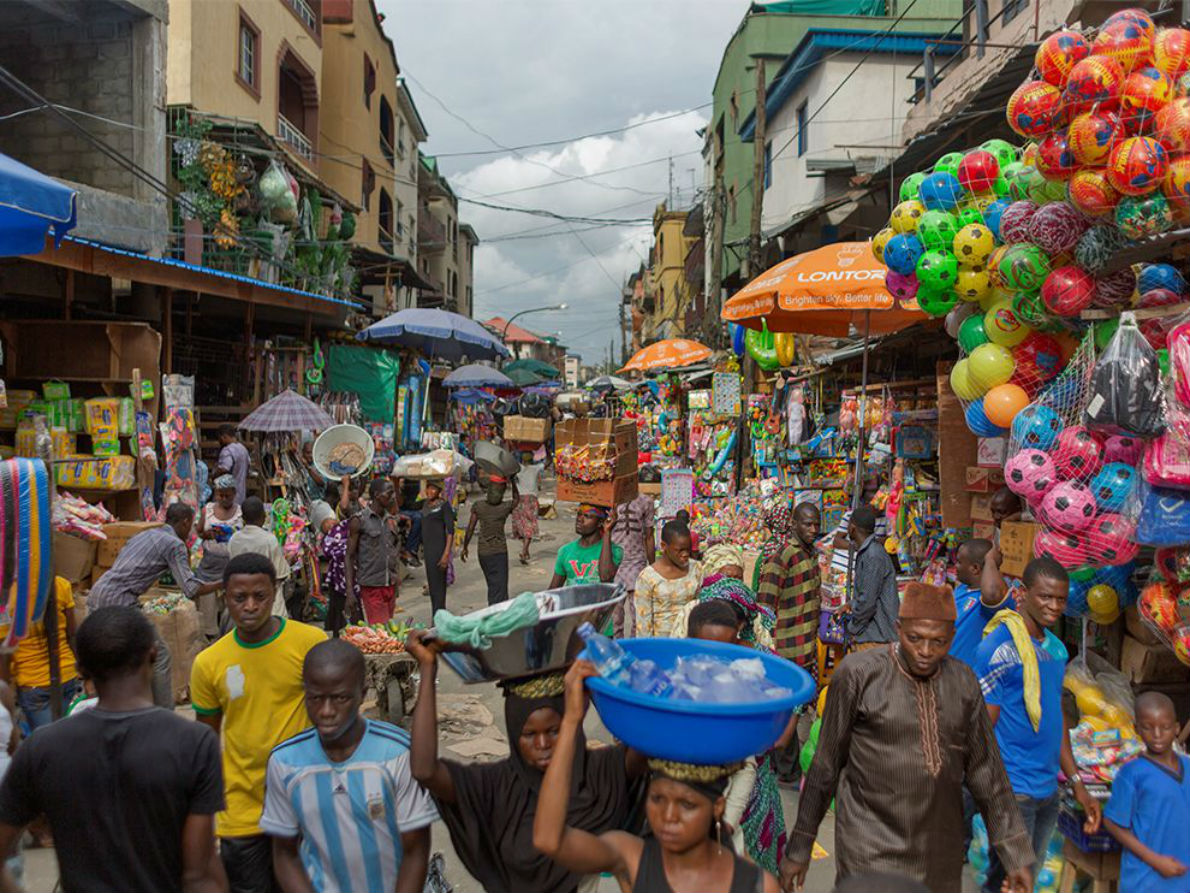

Supplement: Supplementary file 2 [file Data_Sheet_1.zip › Raw Images for Experiment 1/People/ppl011.jpg]

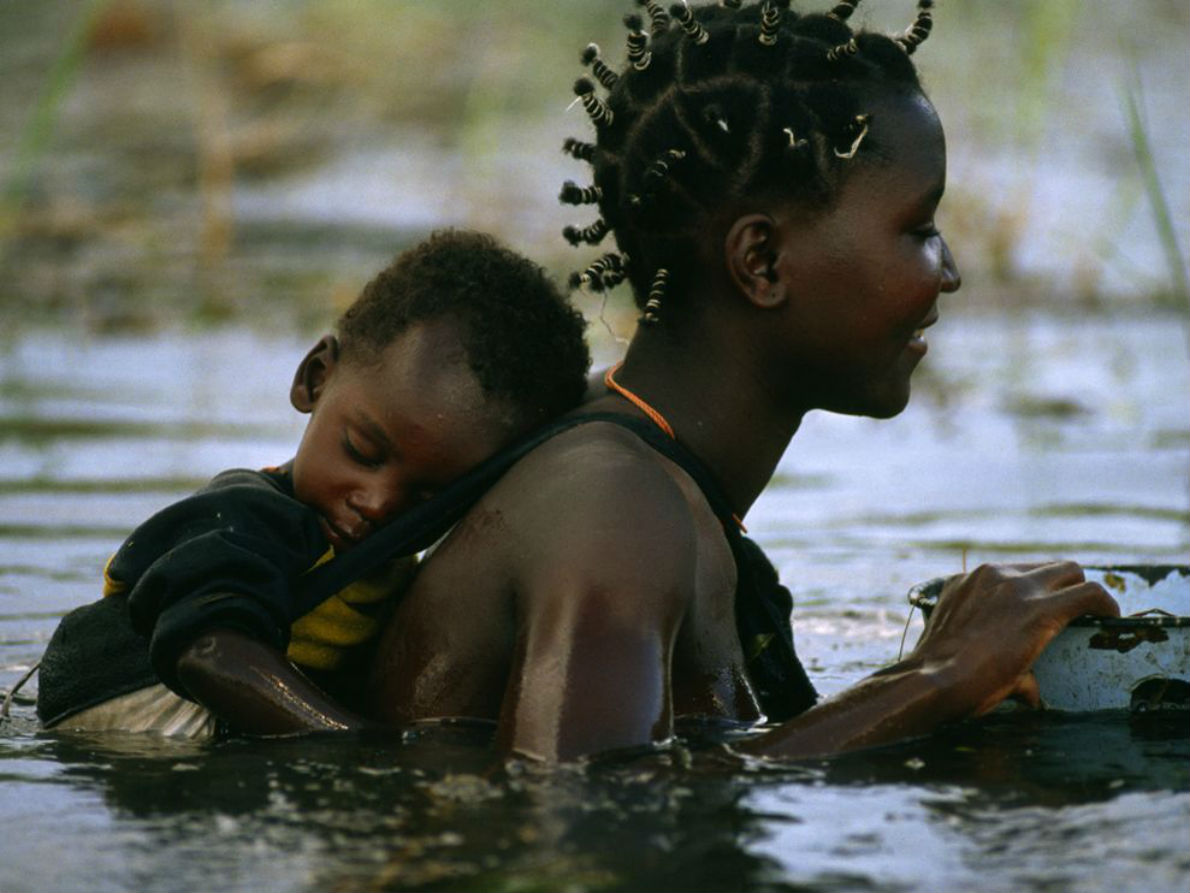

Supplement: Supplementary file 2 [file Data_Sheet_1.zip › Raw Images for Experiment 1/People/ppl012.jpg]

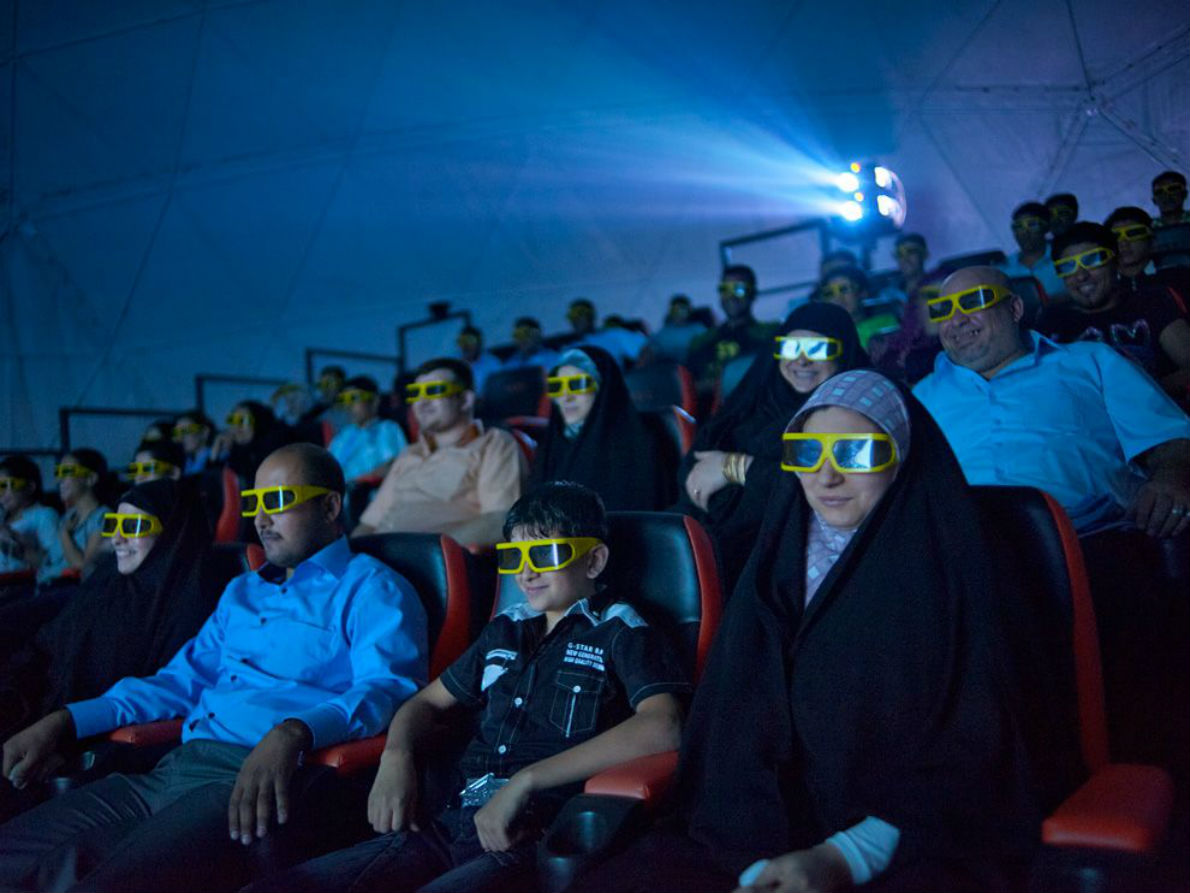

Supplement: Supplementary file 2 [file Data_Sheet_1.zip › Raw Images for Experiment 1/People/ppl013.jpg]

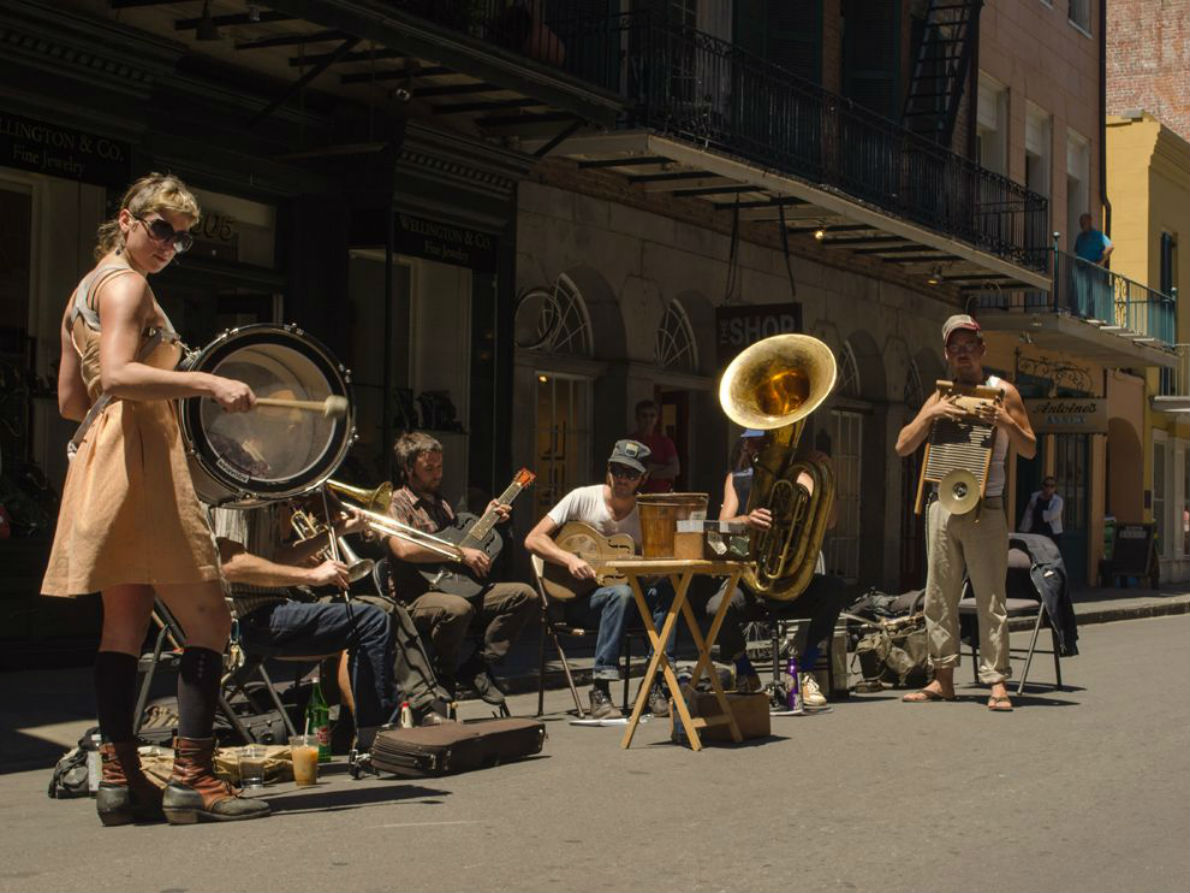

Supplement: Supplementary file 2 [file Data_Sheet_1.zip › Raw Images for Experiment 1/People/ppl014.jpg]

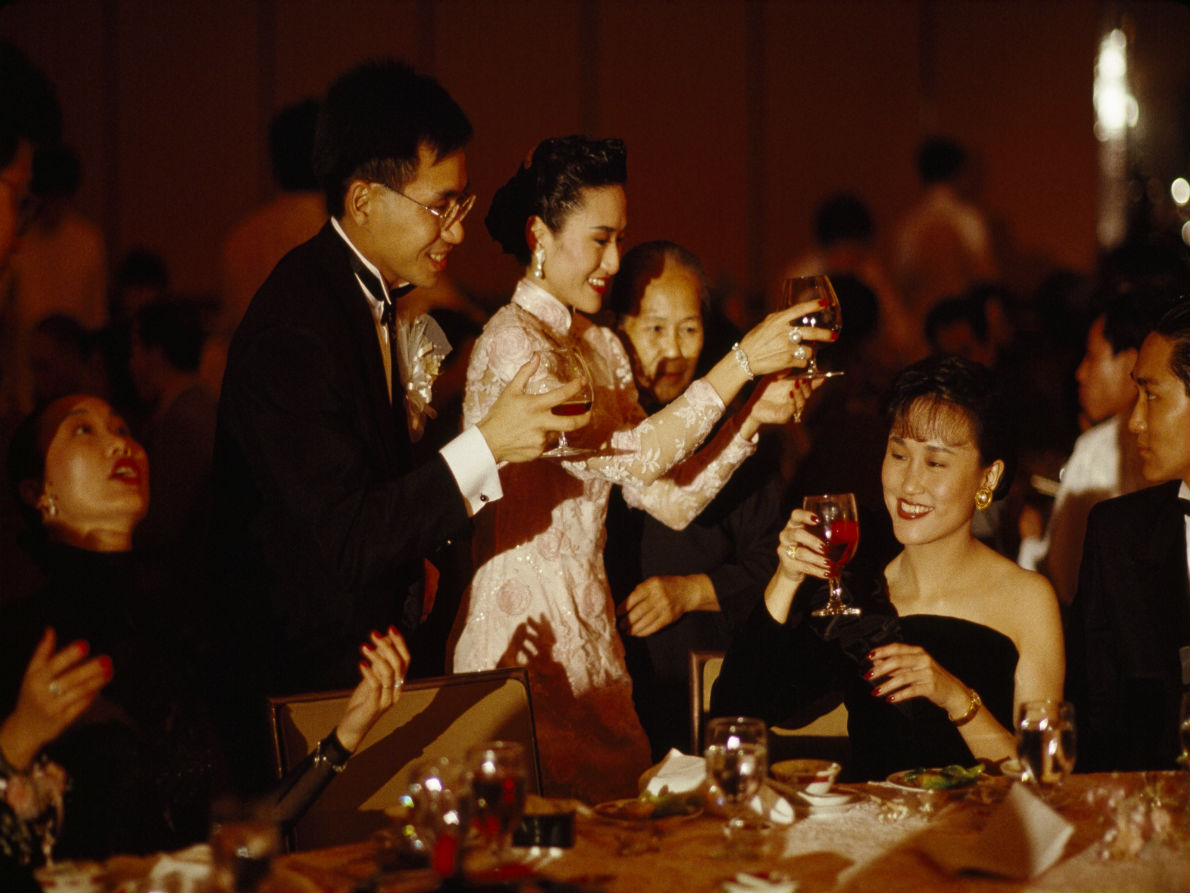

Supplement: Supplementary file 2 [file Data_Sheet_1.zip › Raw Images for Experiment 1/People/ppl015.jpg]

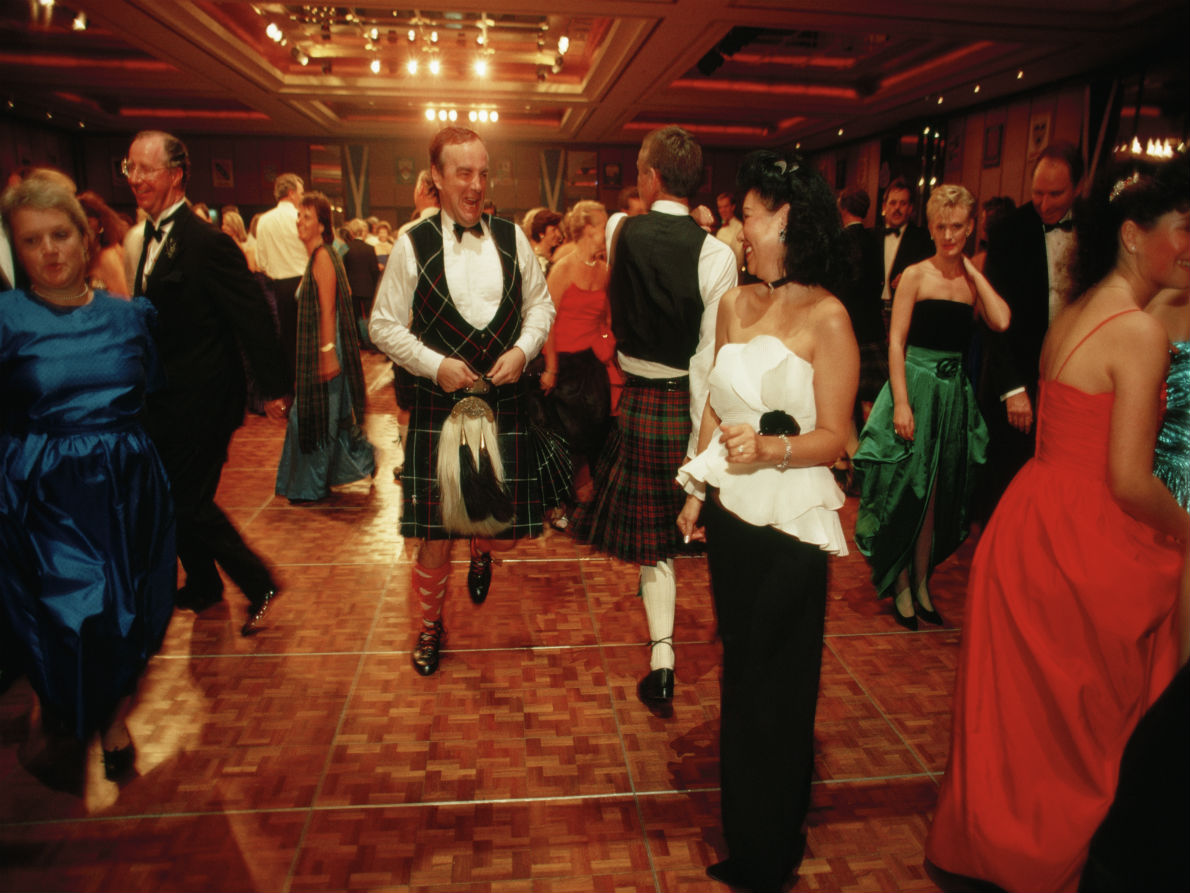

Supplement: Supplementary file 2 [file Data_Sheet_1.zip › Raw Images for Experiment 1/People/ppl016.jpg]

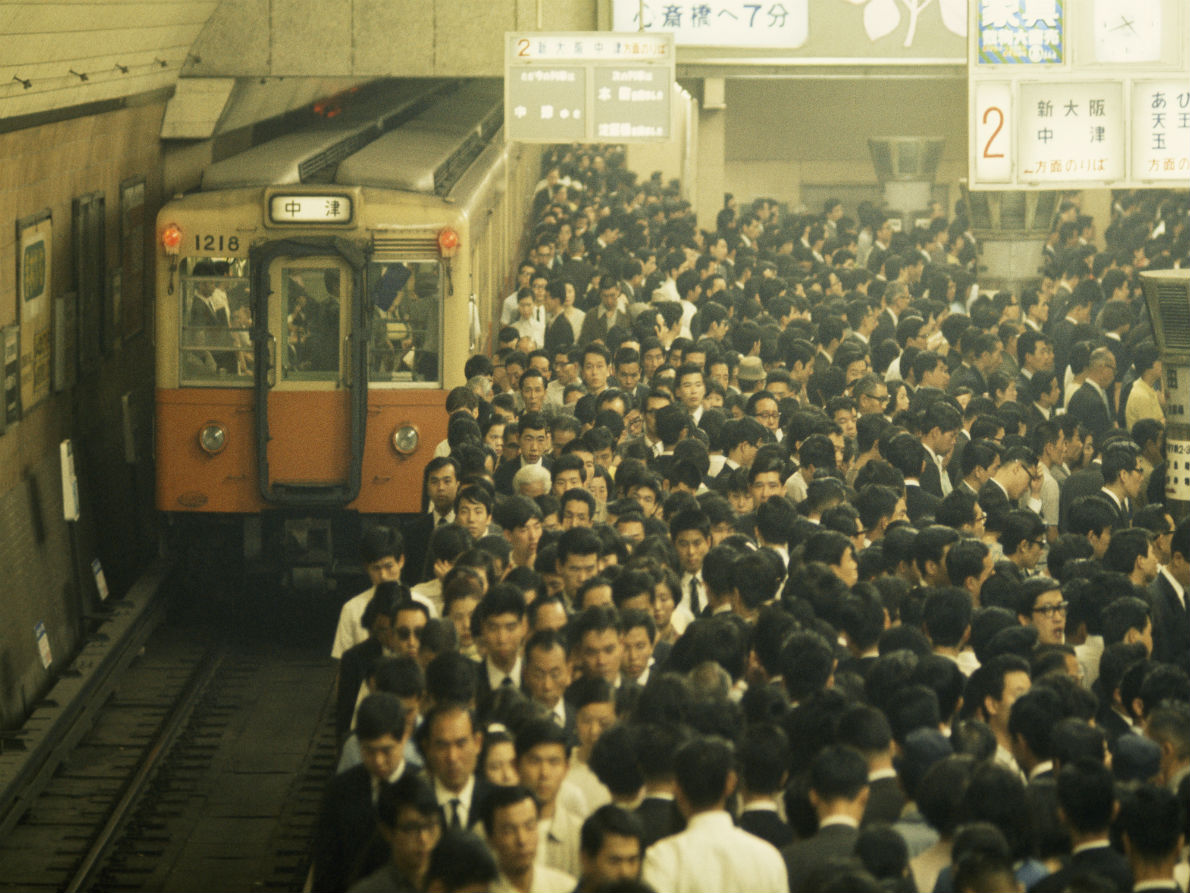

Supplement: Supplementary file 2 [file Data_Sheet_1.zip › Raw Images for Experiment 1/People/ppl017.jpg]

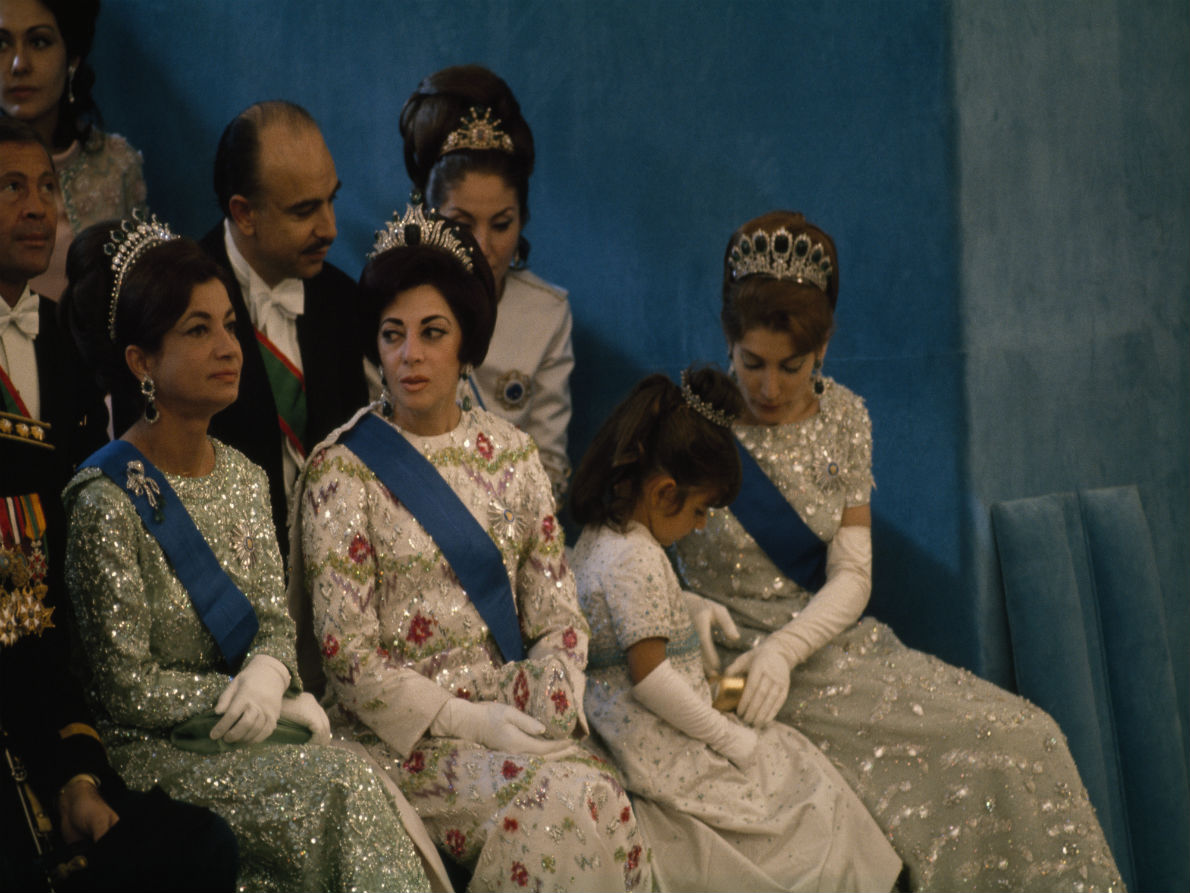

Supplement: Supplementary file 2 [file Data_Sheet_1.zip › Raw Images for Experiment 1/People/ppl018.jpg]

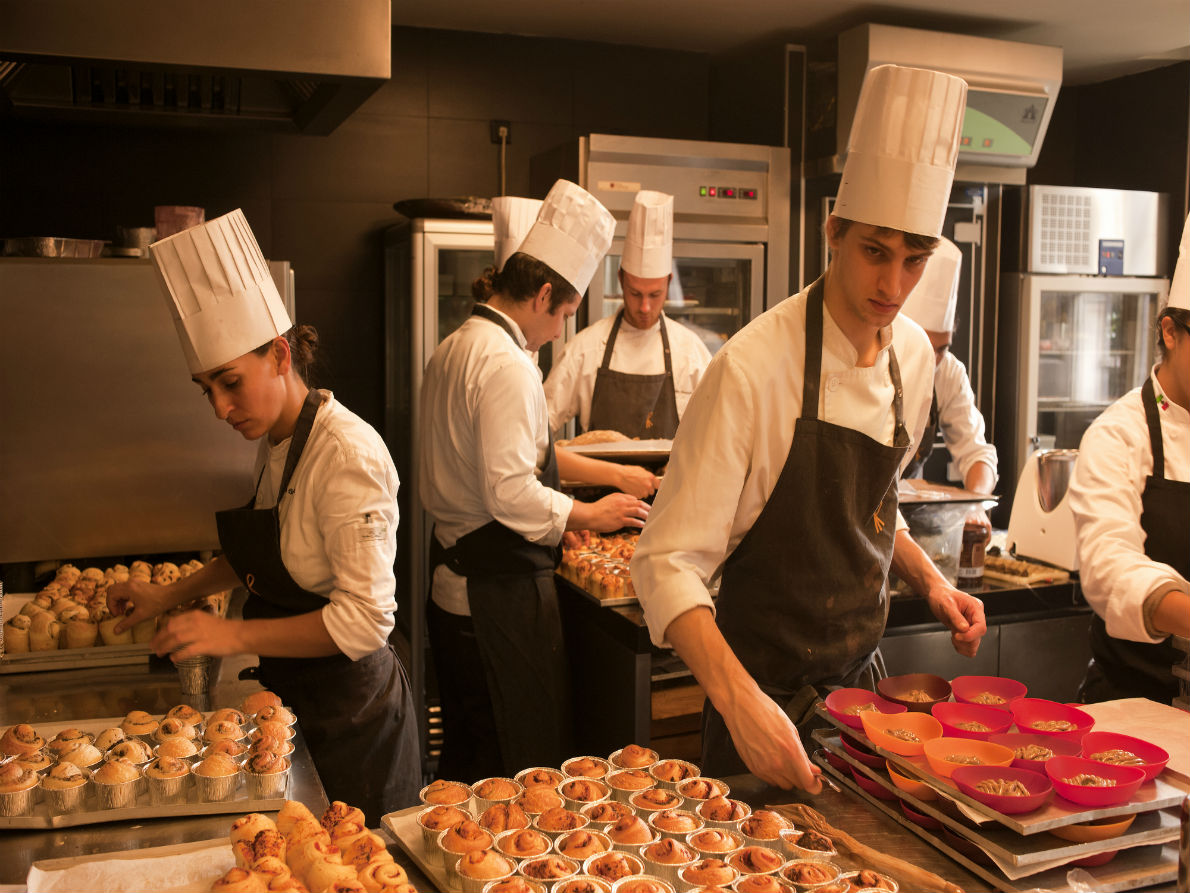

Supplement: Supplementary file 2 [file Data_Sheet_1.zip › Raw Images for Experiment 1/People/ppl019.jpg]

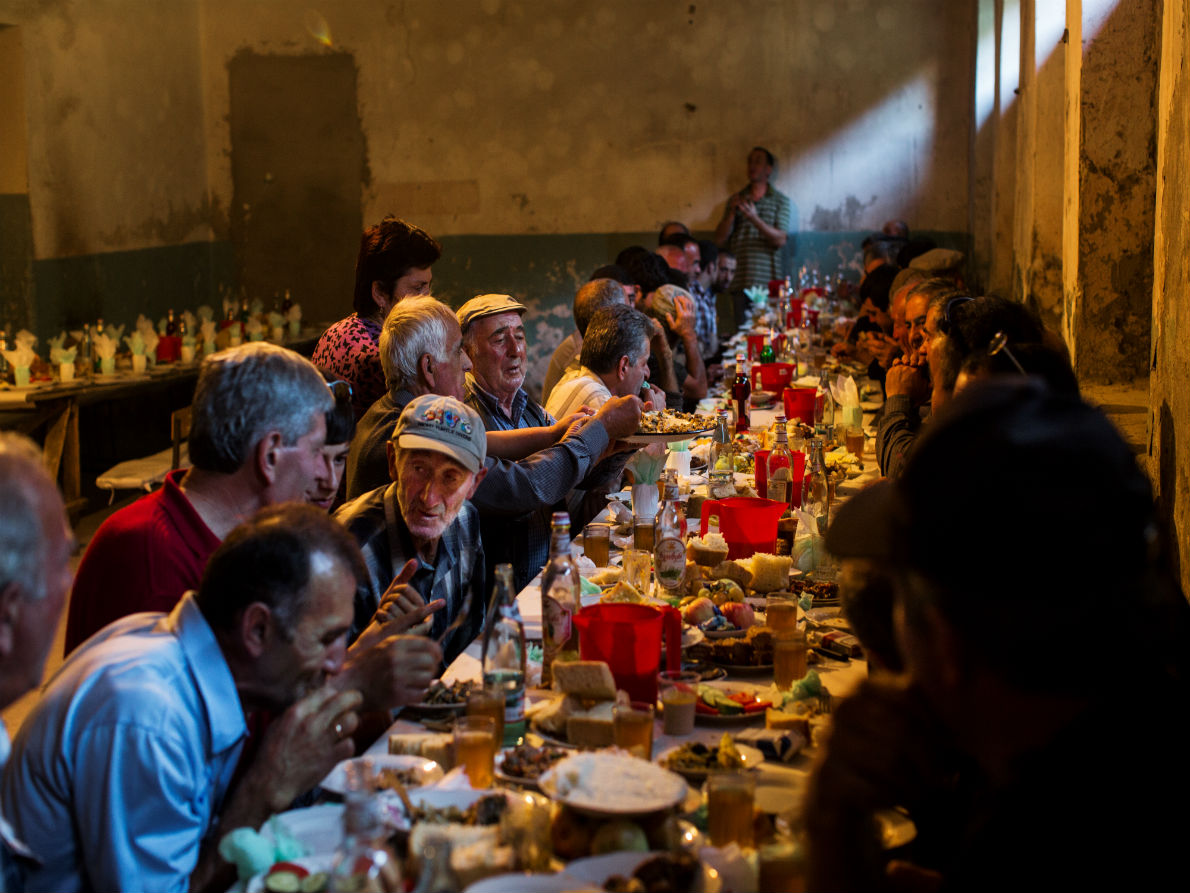

Supplement: Supplementary file 2 [file Data_Sheet_1.zip › Raw Images for Experiment 1/People/ppl020.jpg]

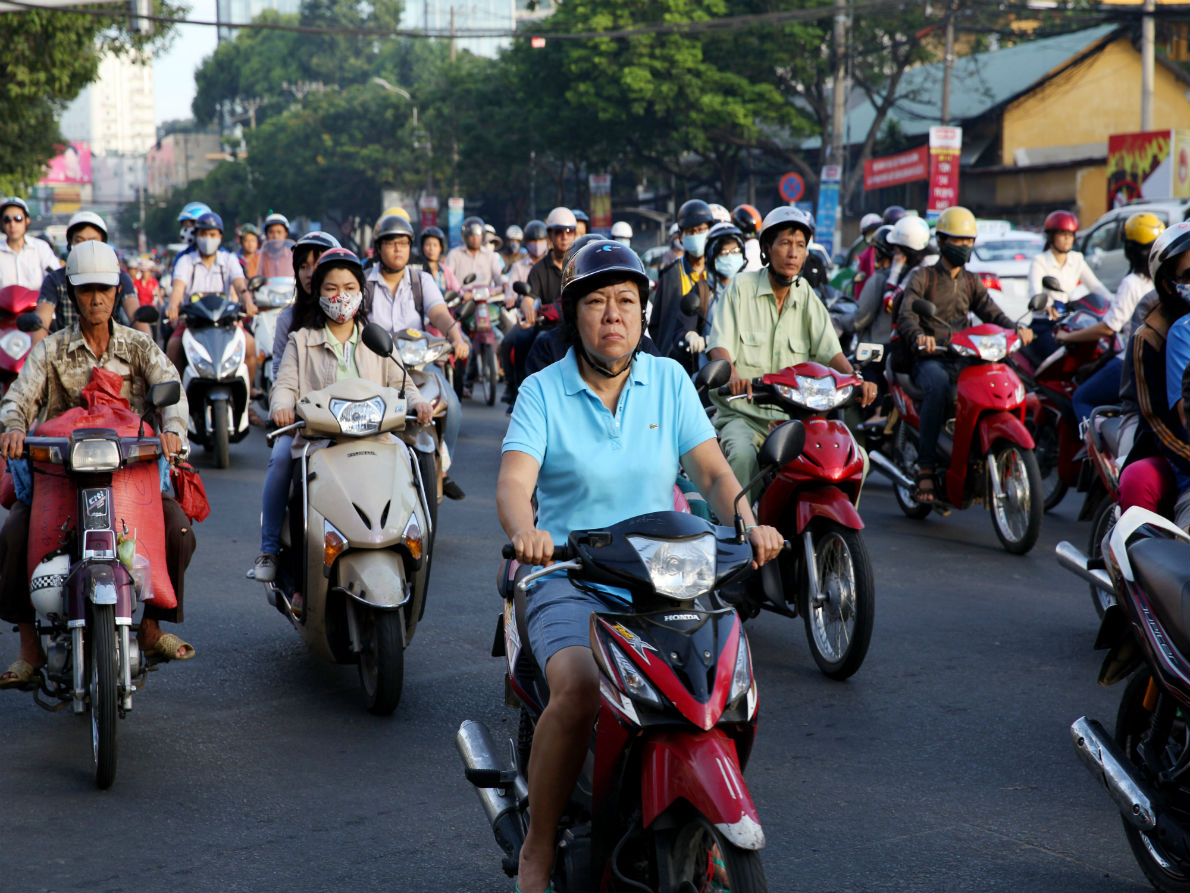

Supplement: Supplementary file 2 [file Data_Sheet_1.zip › Raw Images for Experiment 1/People/ppl021.jpg]

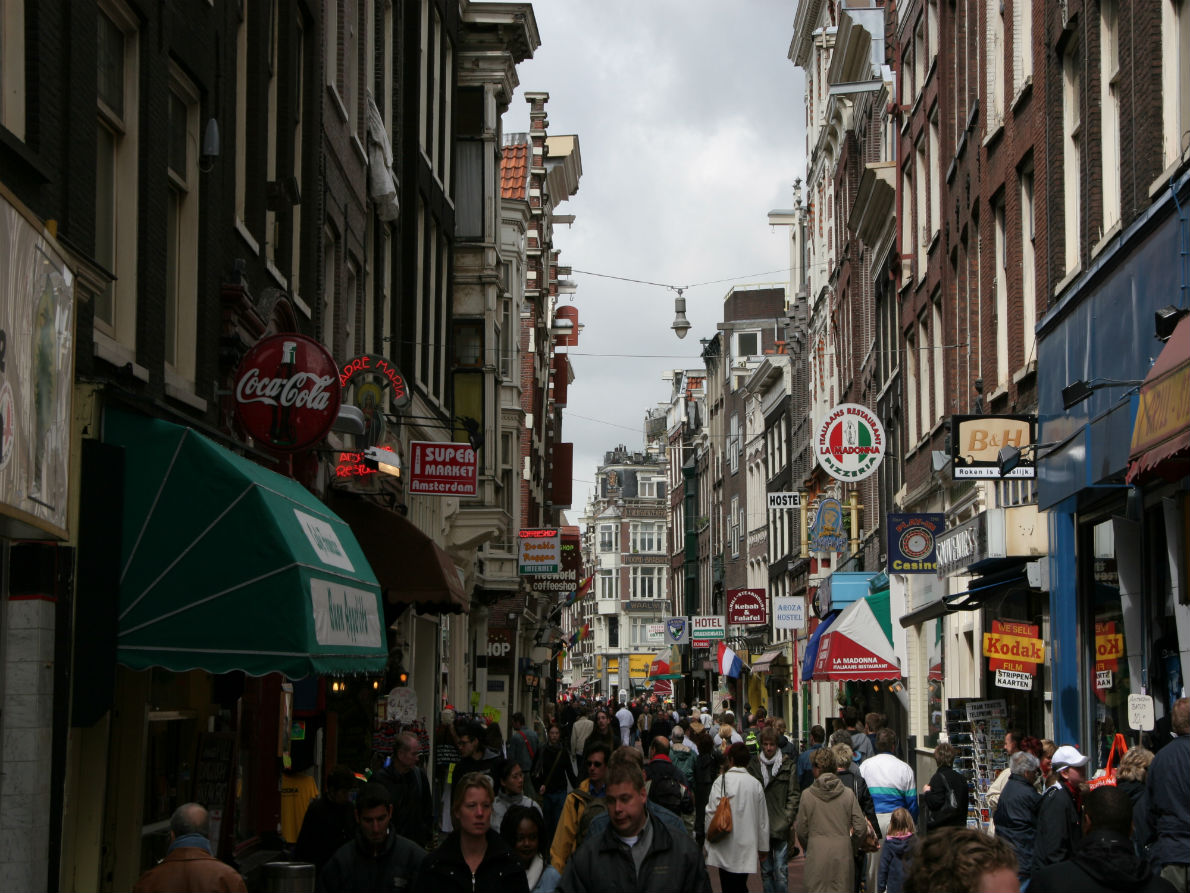

Supplement: Supplementary file 2 [file Data_Sheet_1.zip › Raw Images for Experiment 1/People/ppl022.jpg]

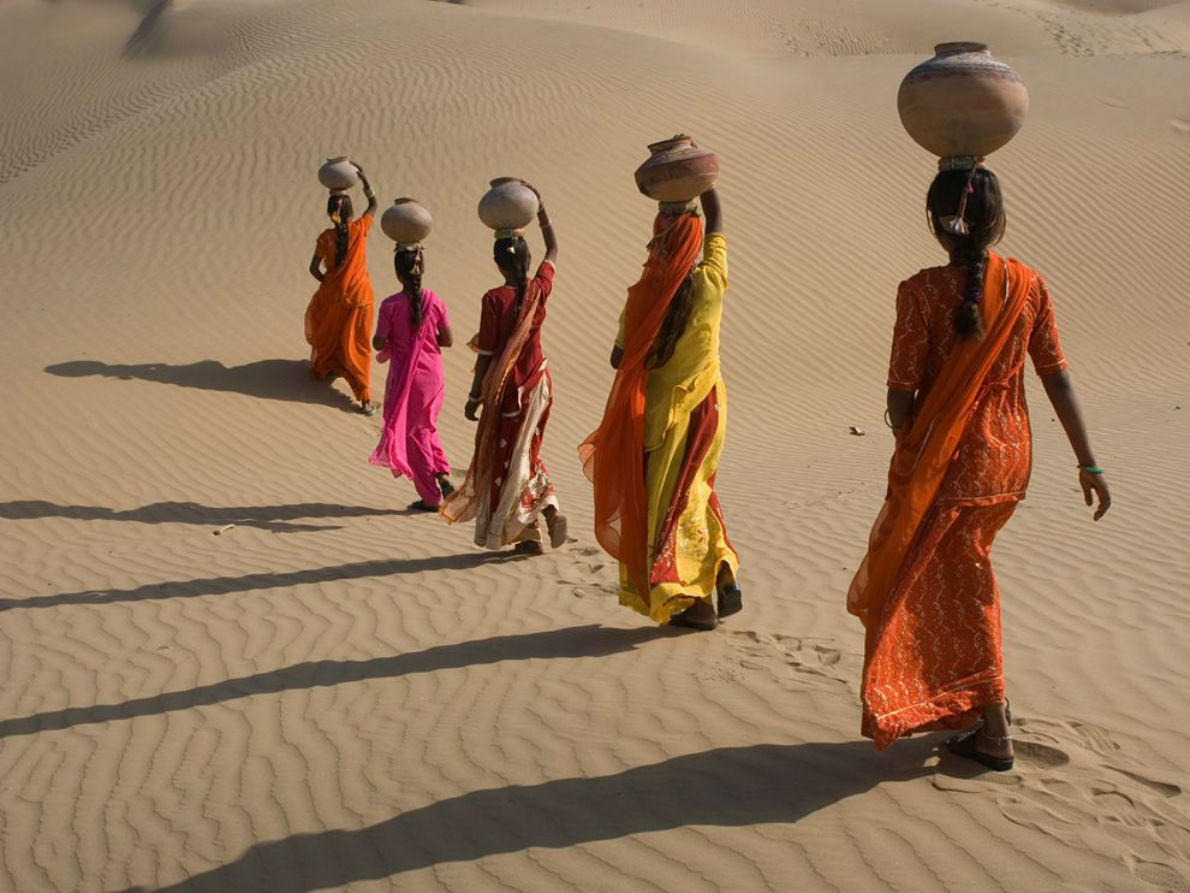

Supplement: Supplementary file 2 [file Data_Sheet_1.zip › Raw Images for Experiment 1/People/ppl024.jpg]

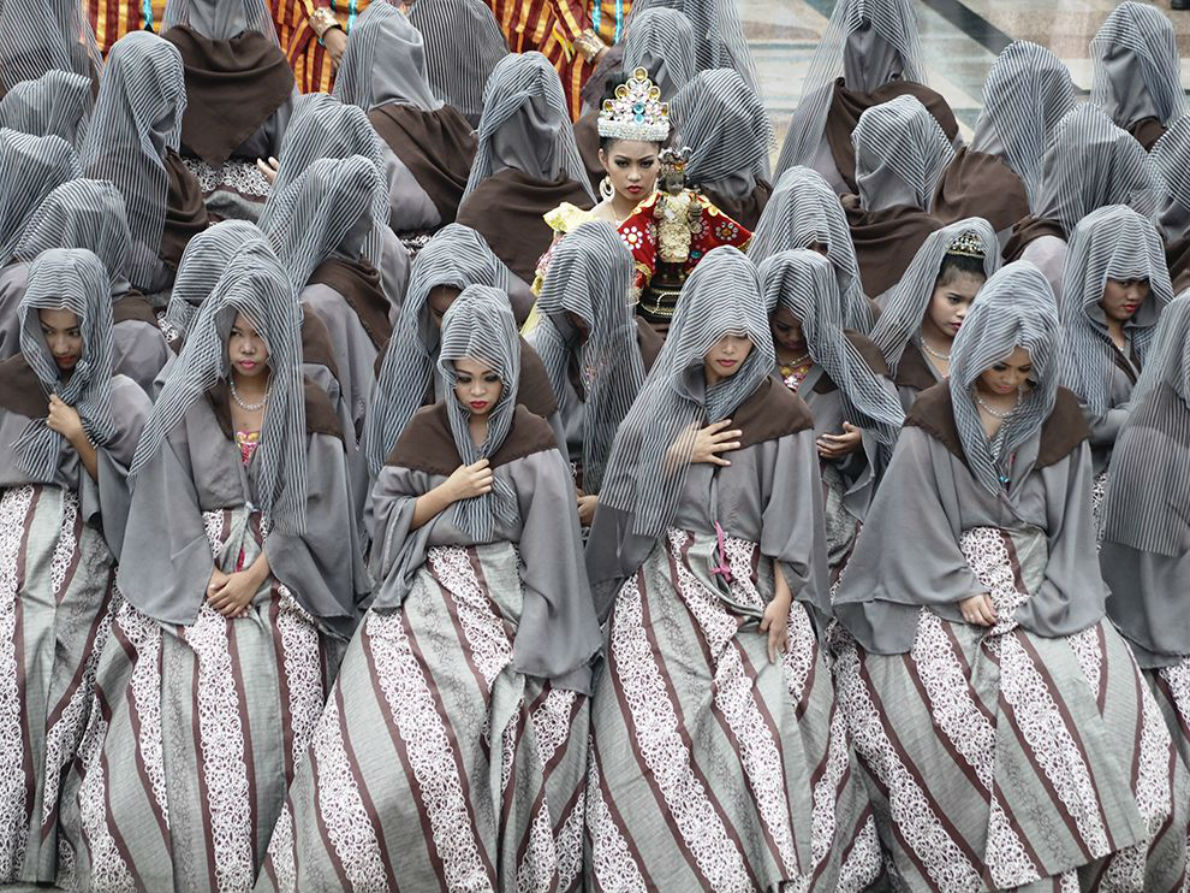

Supplement: Supplementary file 2 [file Data_Sheet_1.zip › Raw Images for Experiment 1/People/ppl025.jpg]

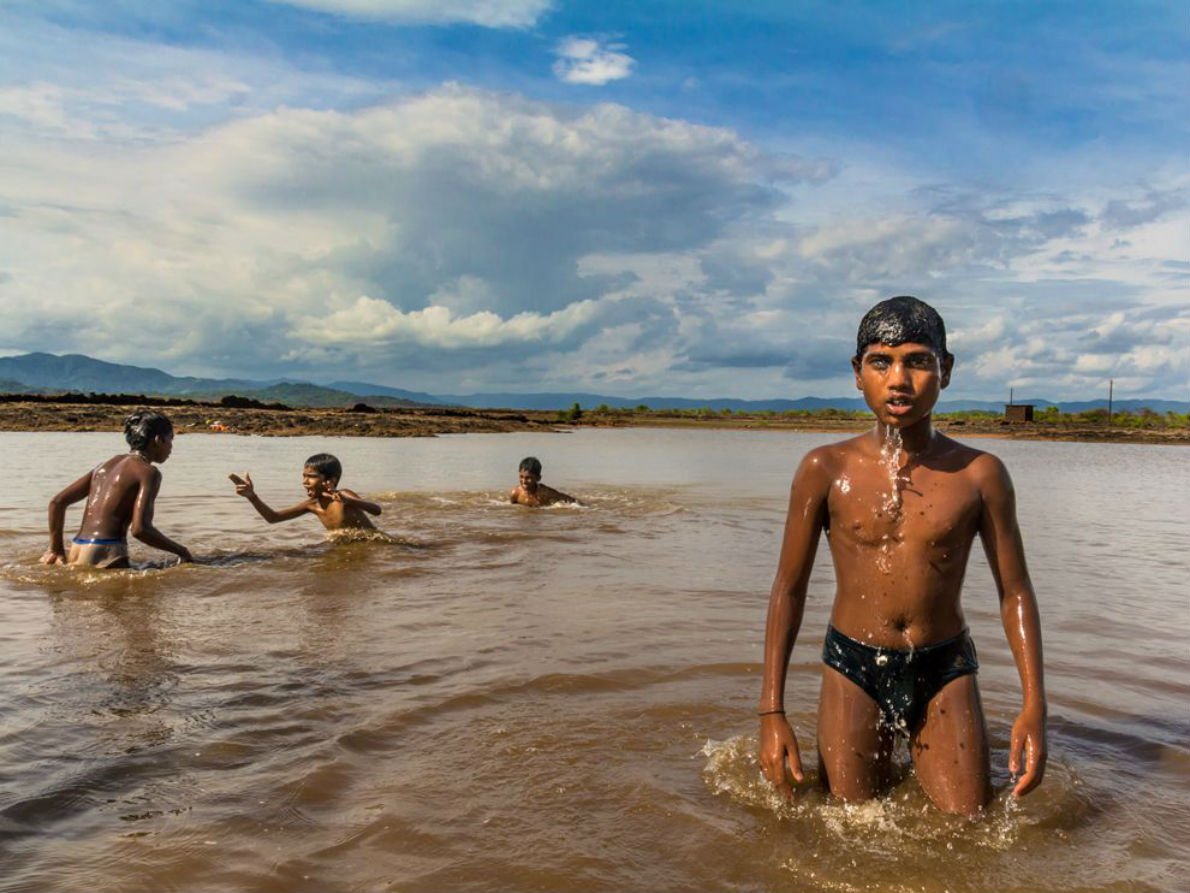

Supplement: Supplementary file 2 [file Data_Sheet_1.zip › Raw Images for Experiment 1/People/ppl026.jpg]

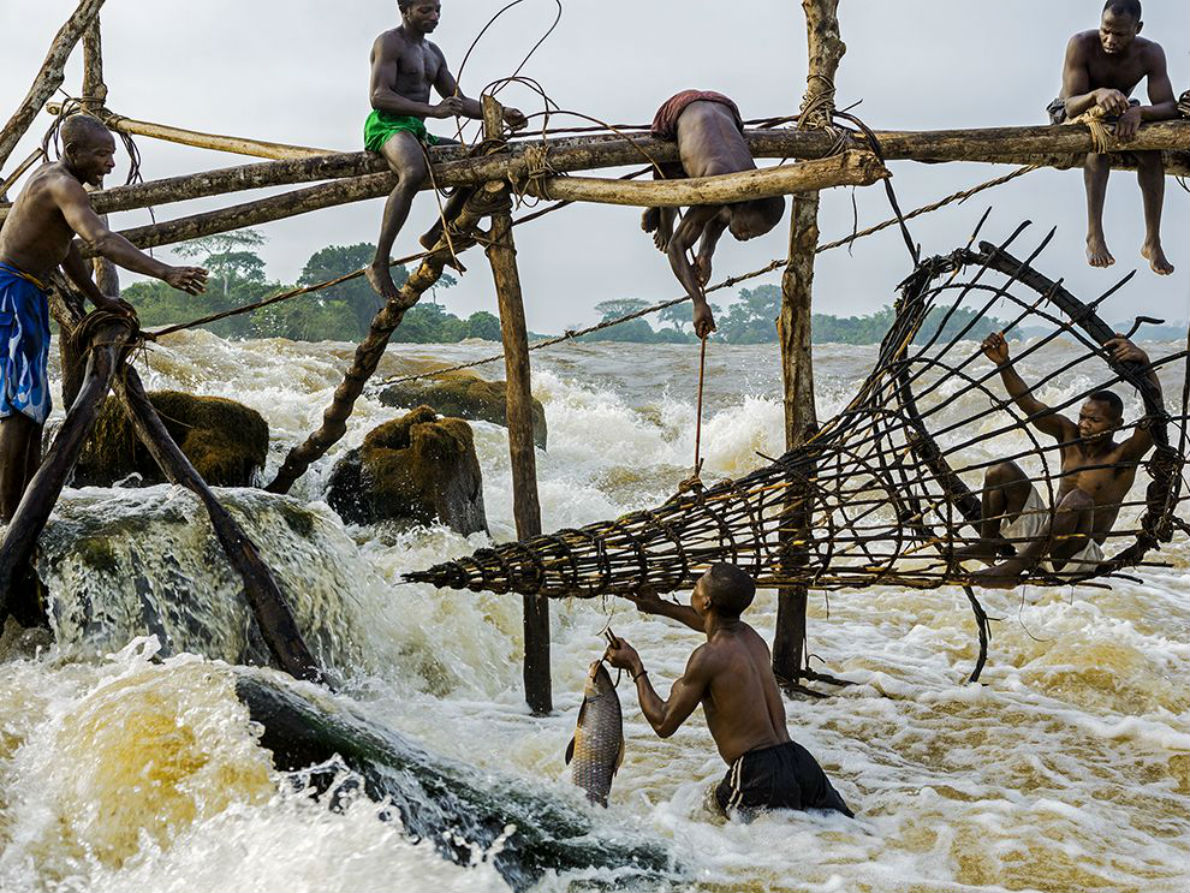

Supplement: Supplementary file 2 [file Data_Sheet_1.zip › Raw Images for Experiment 1/People/ppl027.jpg]

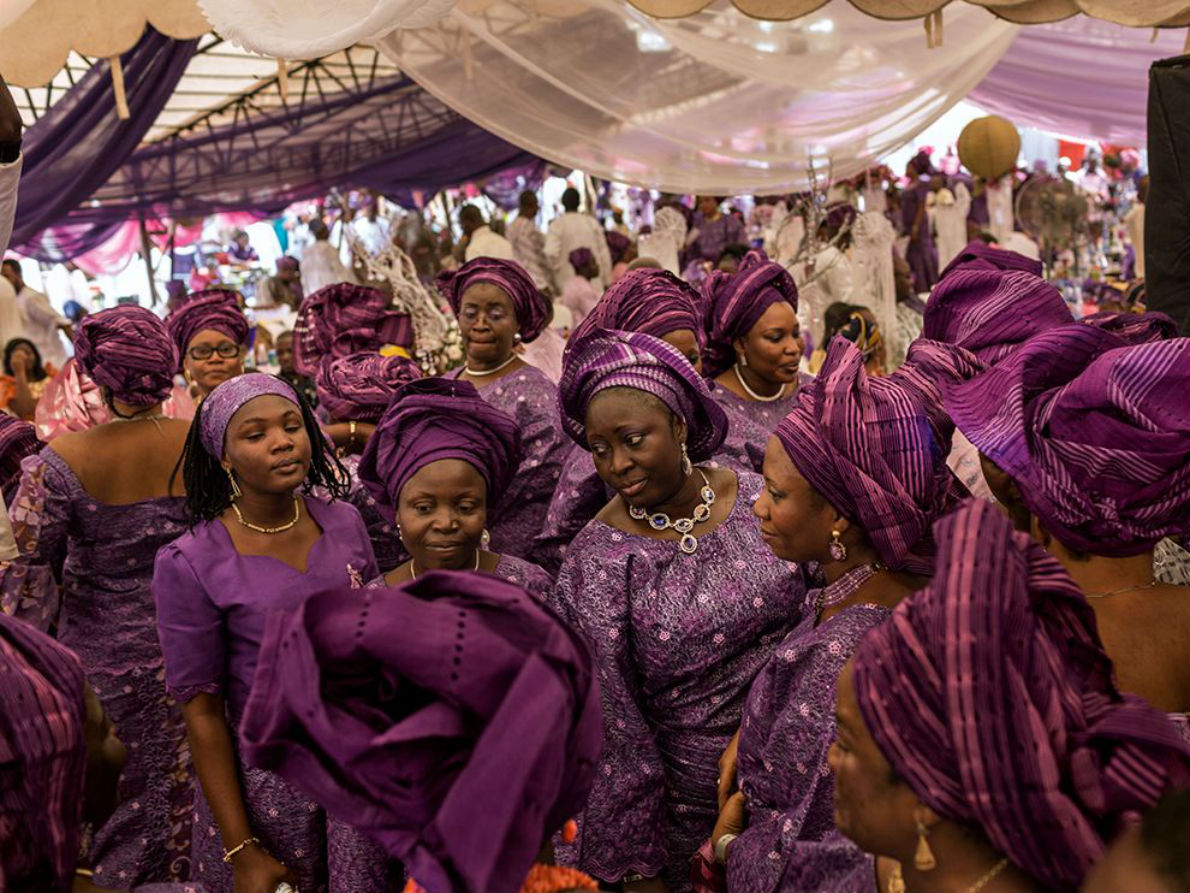

Supplement: Supplementary file 2 [file Data_Sheet_1.zip › Raw Images for Experiment 1/People/ppl028.jpg]

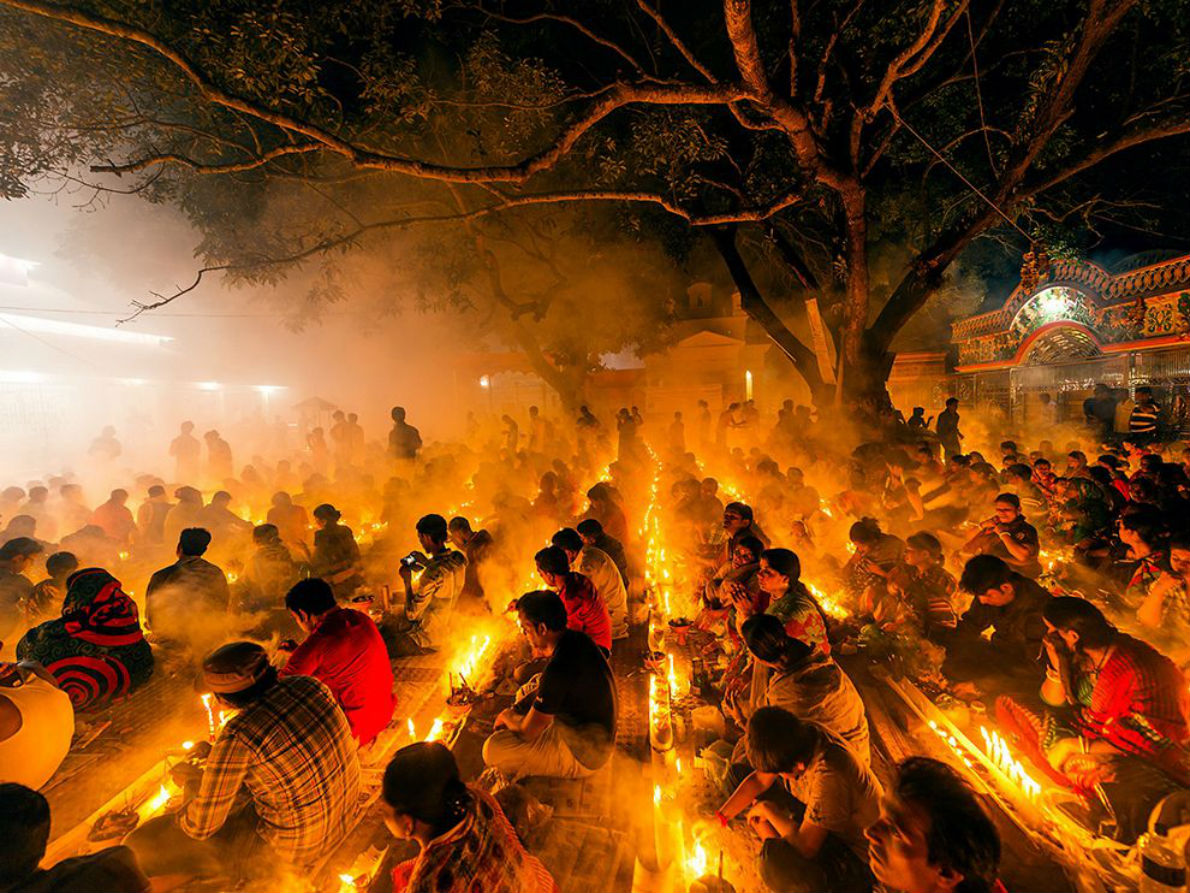

Supplement: Supplementary file 2 [file Data_Sheet_1.zip › Raw Images for Experiment 1/People/ppl1.jpg]

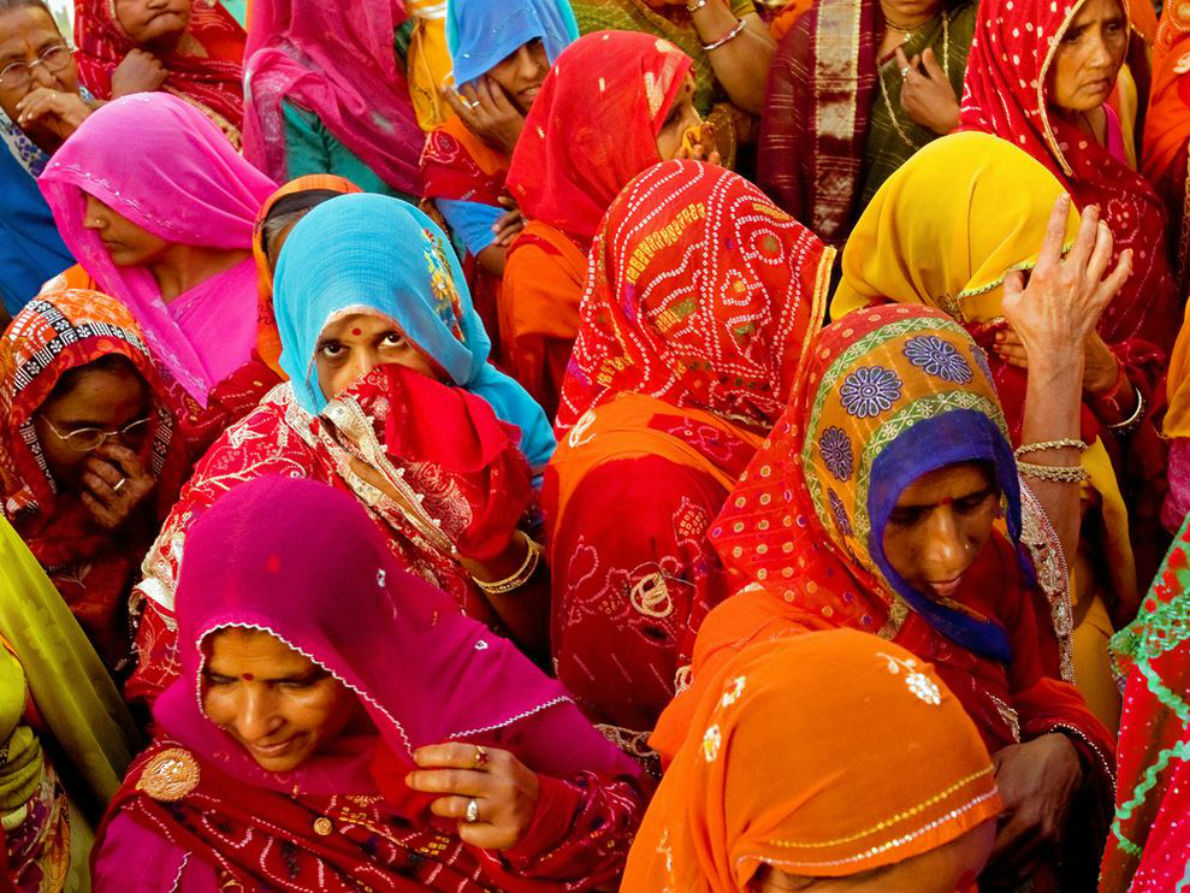

Supplement: Supplementary file 2 [file Data_Sheet_1.zip › Raw Images for Experiment 1/People/ppl2.jpg]

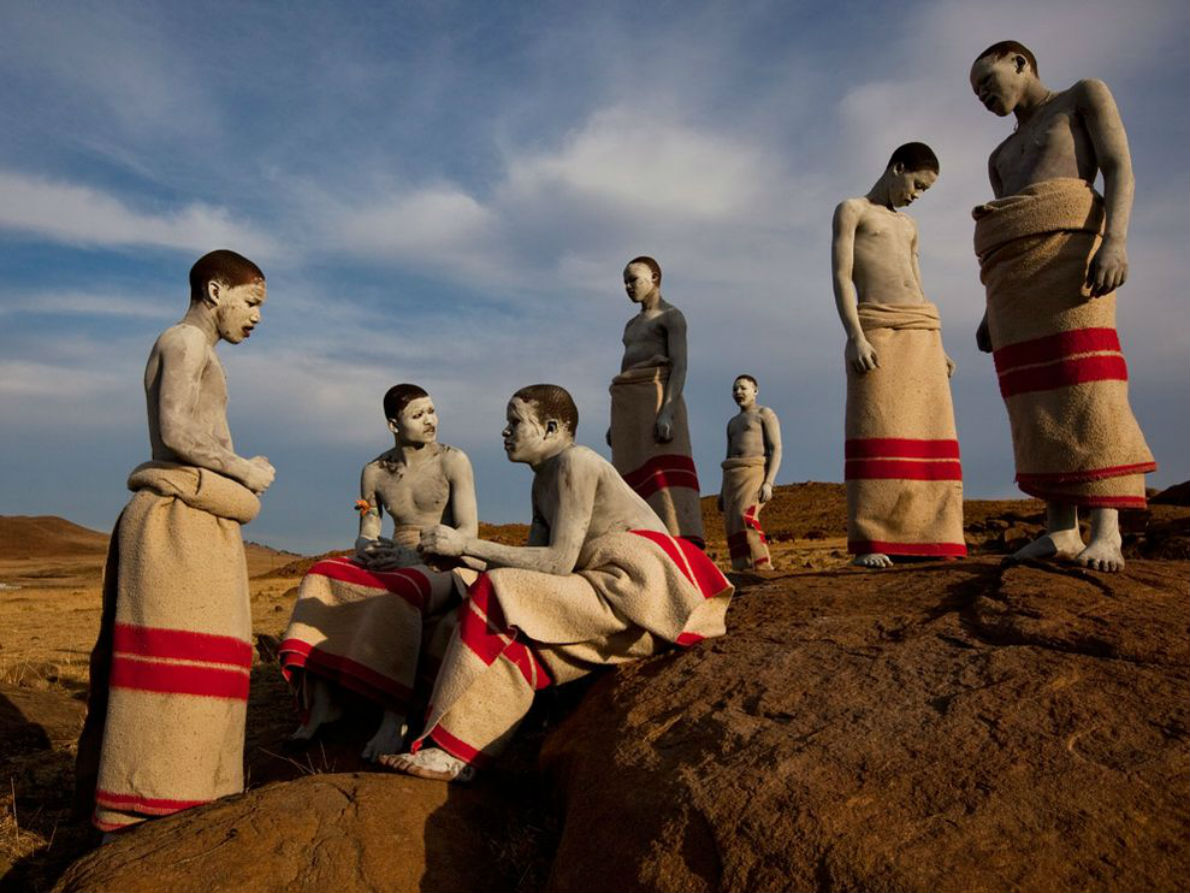

Supplement: Supplementary file 2 [file Data_Sheet_1.zip › Raw Images for Experiment 1/People/ppl3.jpg]

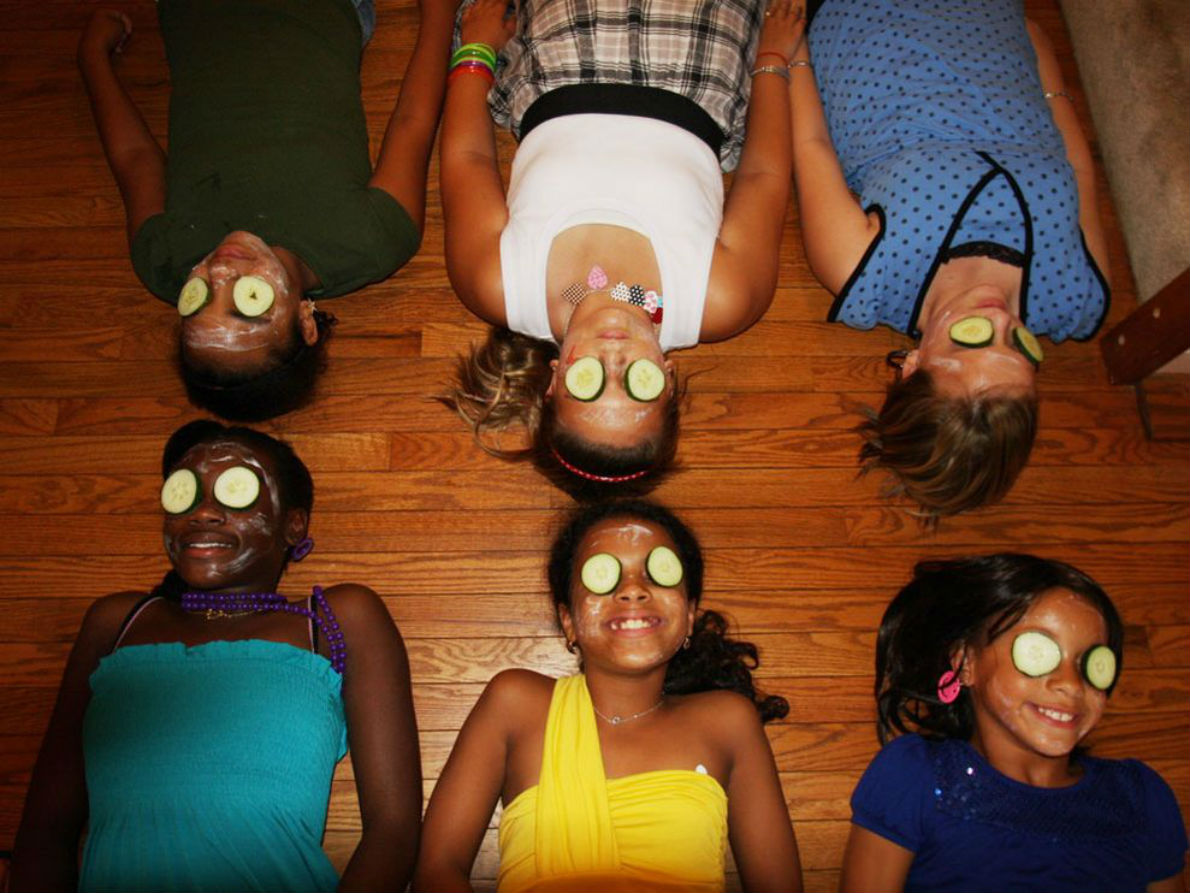

Supplement: Supplementary file 2 [file Data_Sheet_1.zip › Raw Images for Experiment 1/People/ppl4.jpg]

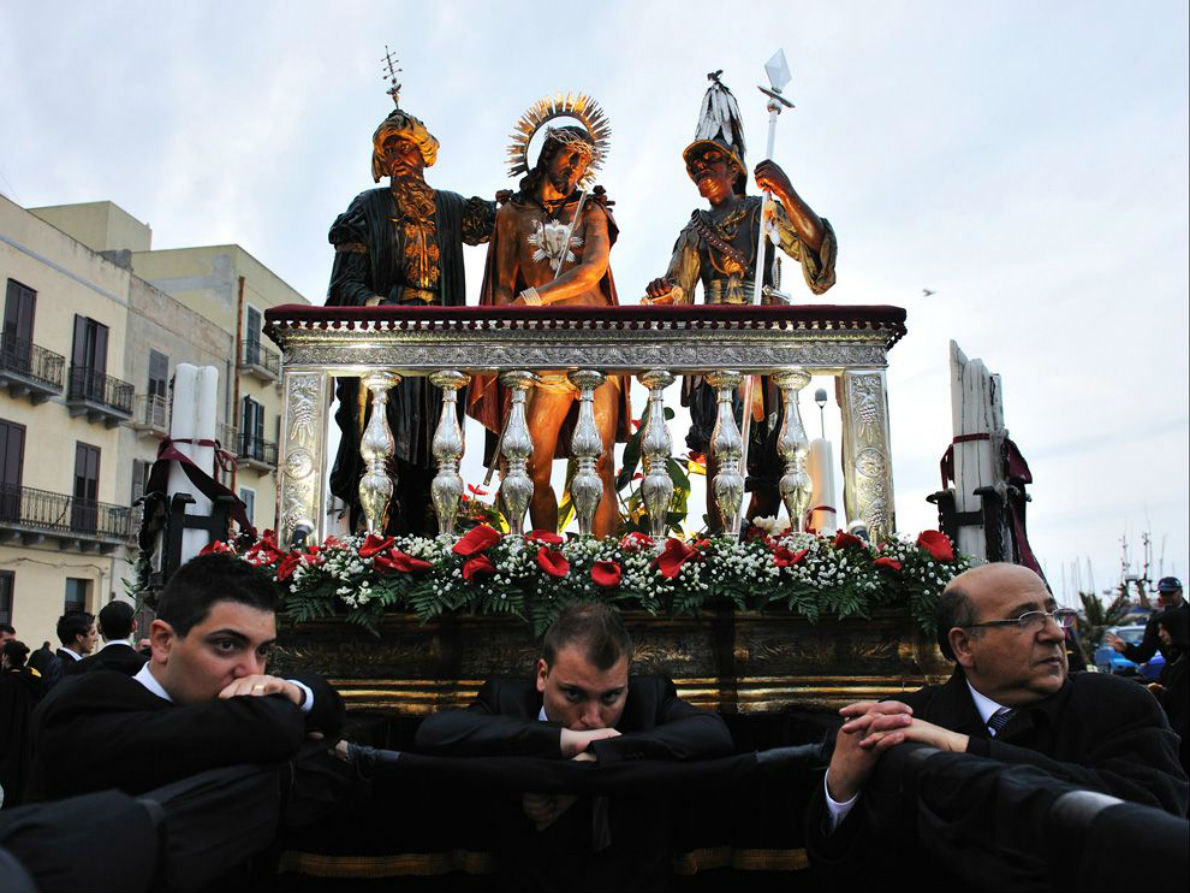

Supplement: Supplementary file 2 [file Data_Sheet_1.zip › Raw Images for Experiment 1/People/ppl5.jpg]

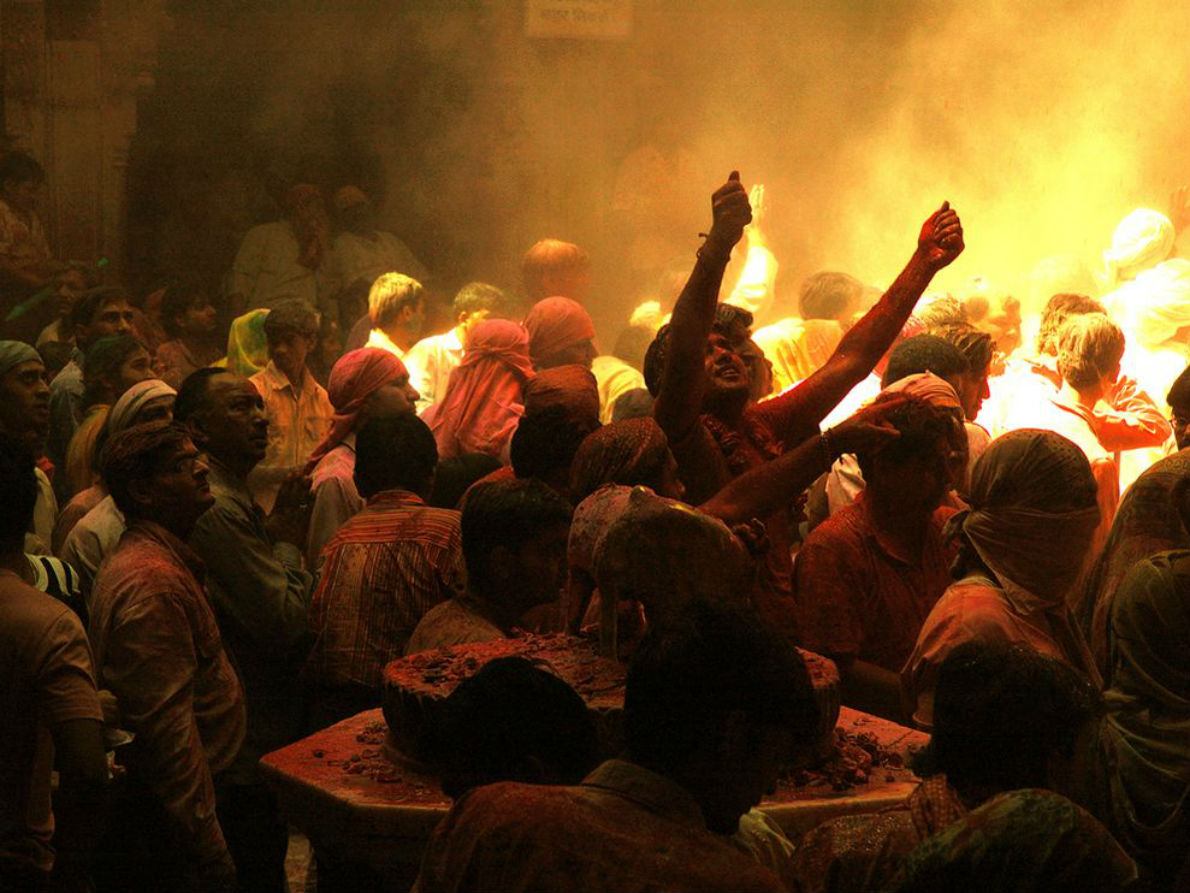

Supplement: Supplementary file 2 [file Data_Sheet_1.zip › Raw Images for Experiment 1/People/ppl7.jpg]

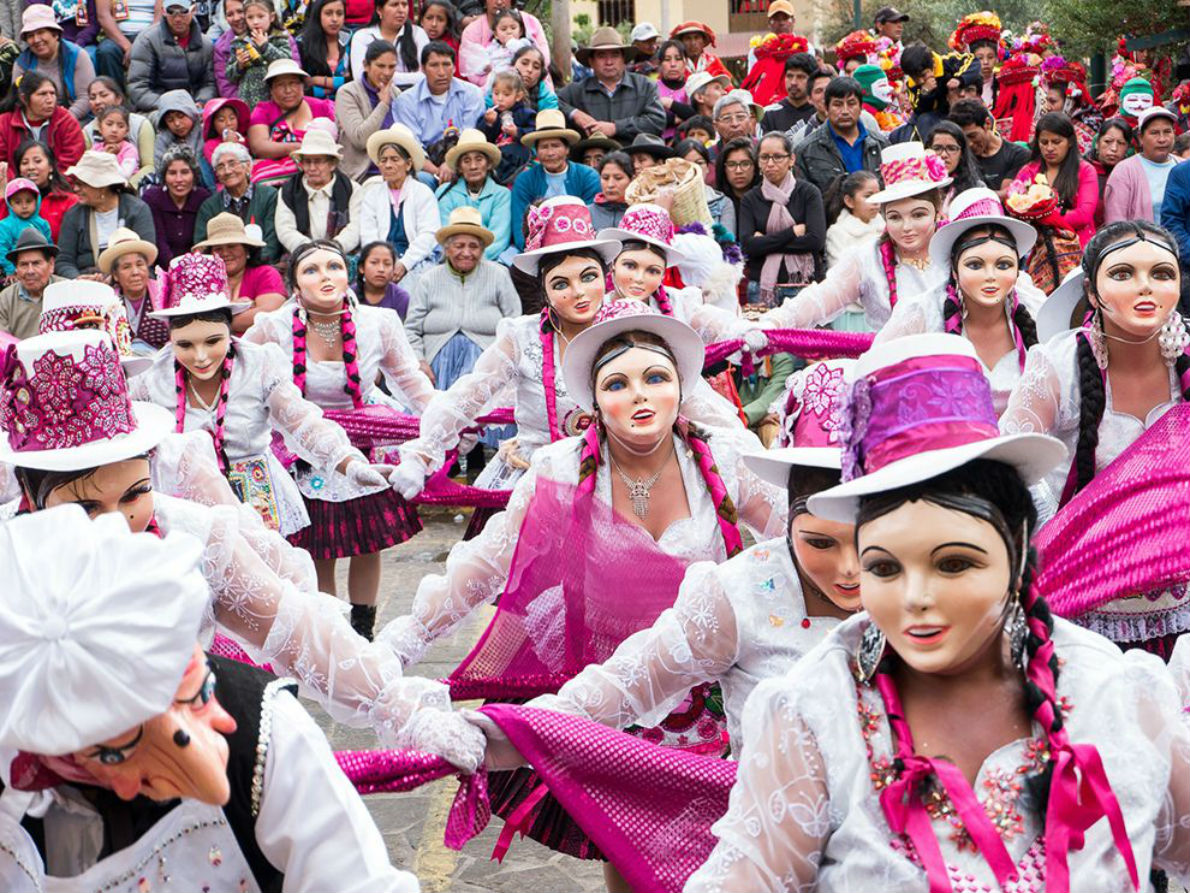

Supplement: Supplementary file 2 [file Data_Sheet_1.zip › Raw Images for Experiment 1/People/ppl8.jpg]

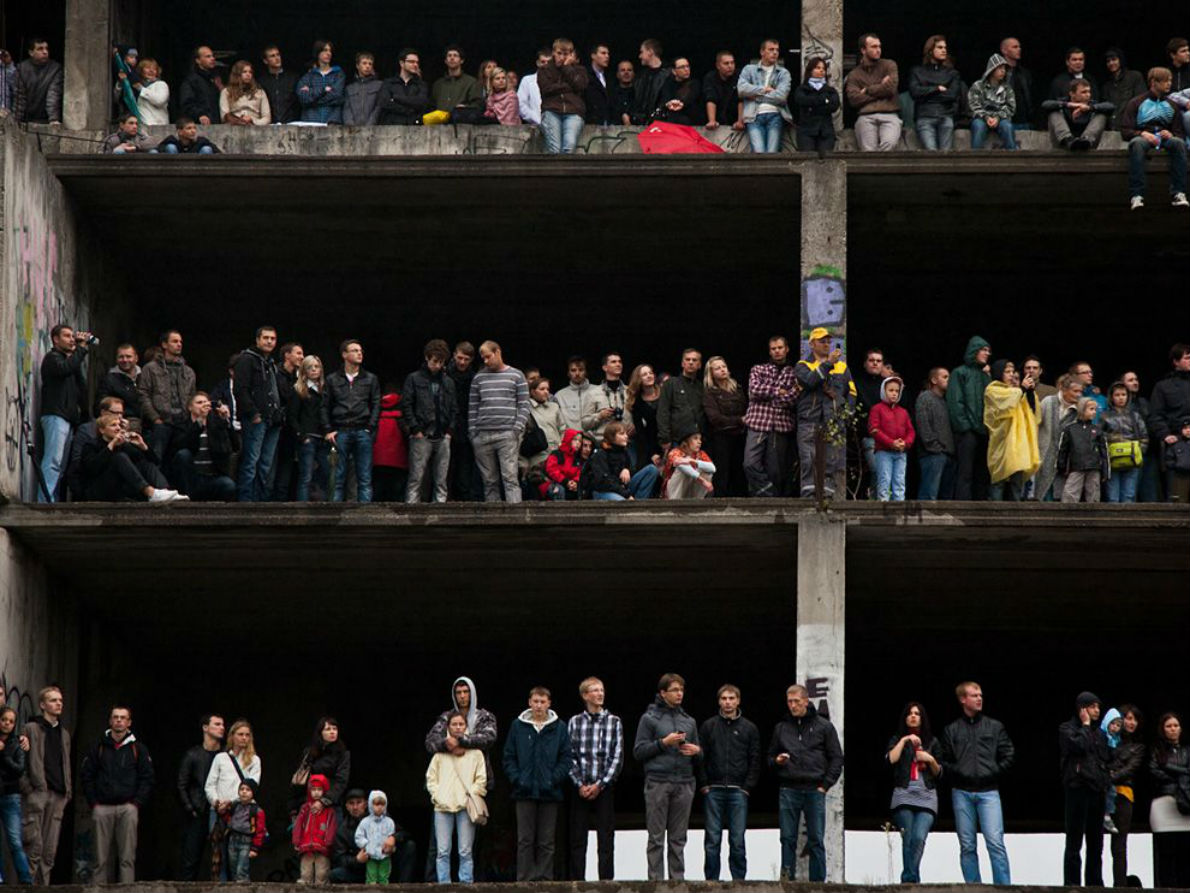

Supplement: Supplementary file 2 [file Data_Sheet_1.zip › Raw Images for Experiment 1/People/ppl9.jpg]

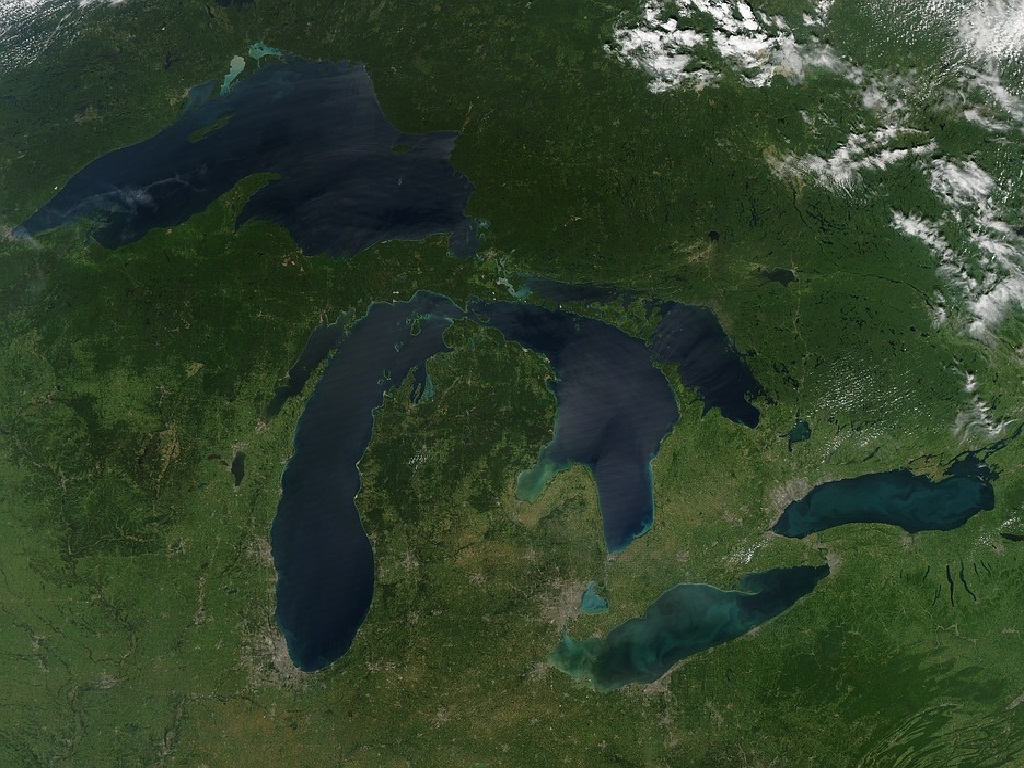

Supplement: Supplementary file 2 [file Data_Sheet_1.zip › Raw Images for Experiment 2/Aerials/aerial02.jpg]

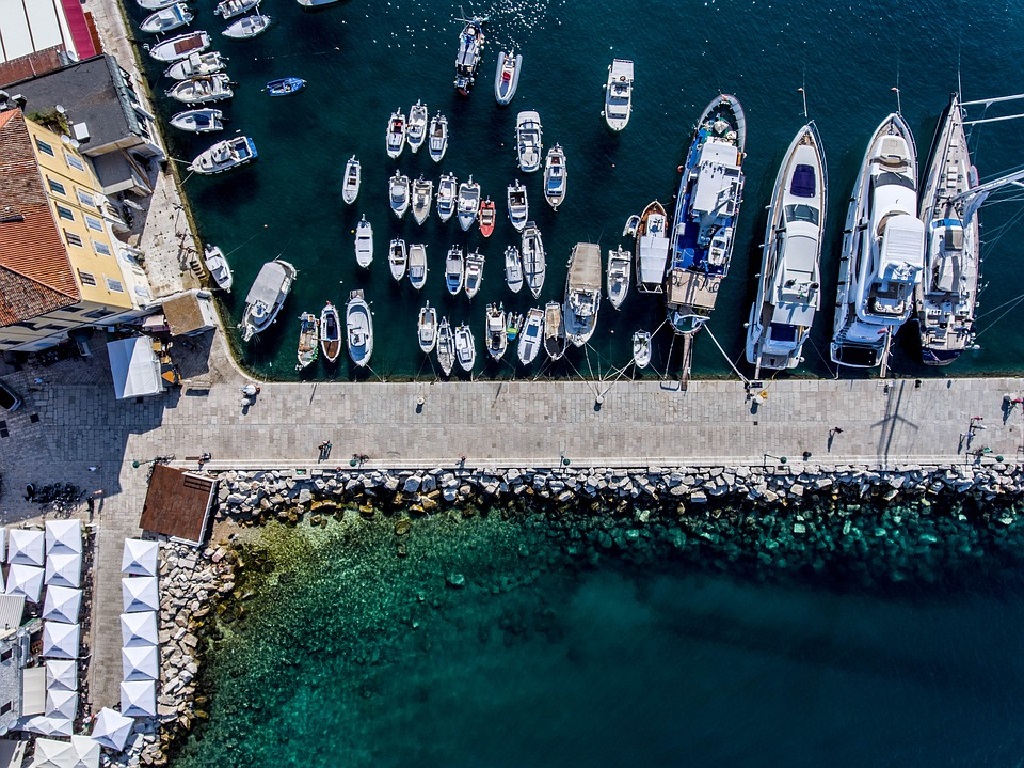

Supplement: Supplementary file 2 [file Data_Sheet_1.zip › Raw Images for Experiment 2/Aerials/aerial04.jpg]

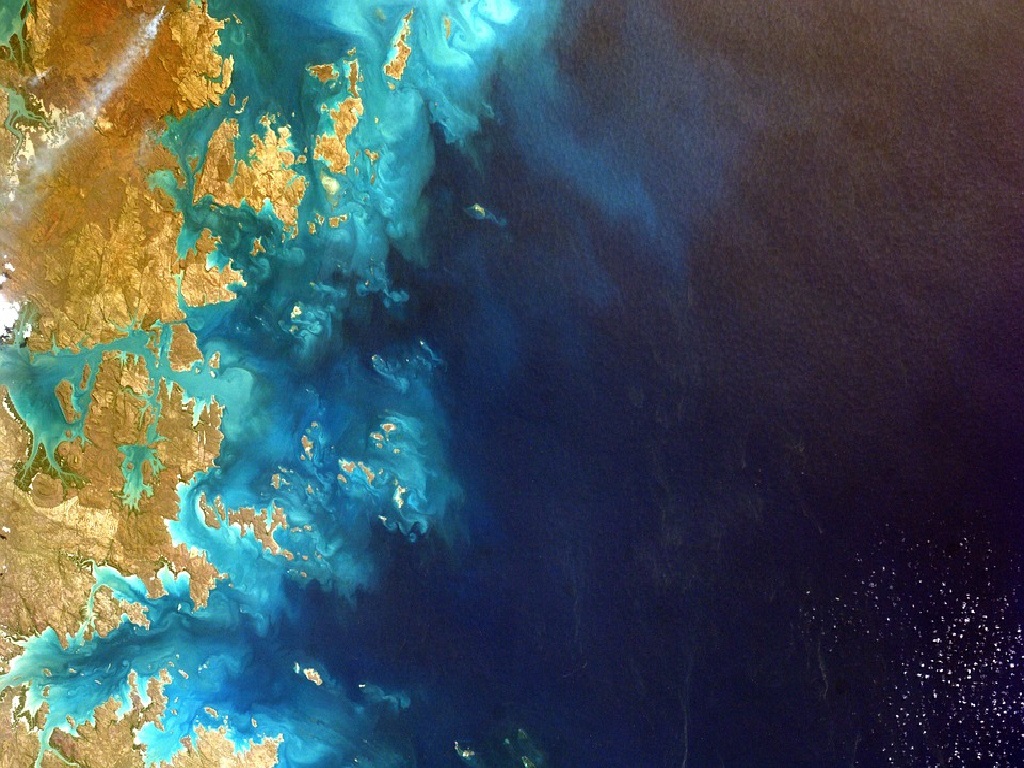

Supplement: Supplementary file 2 [file Data_Sheet_1.zip › Raw Images for Experiment 2/Aerials/aerial10.jpg]

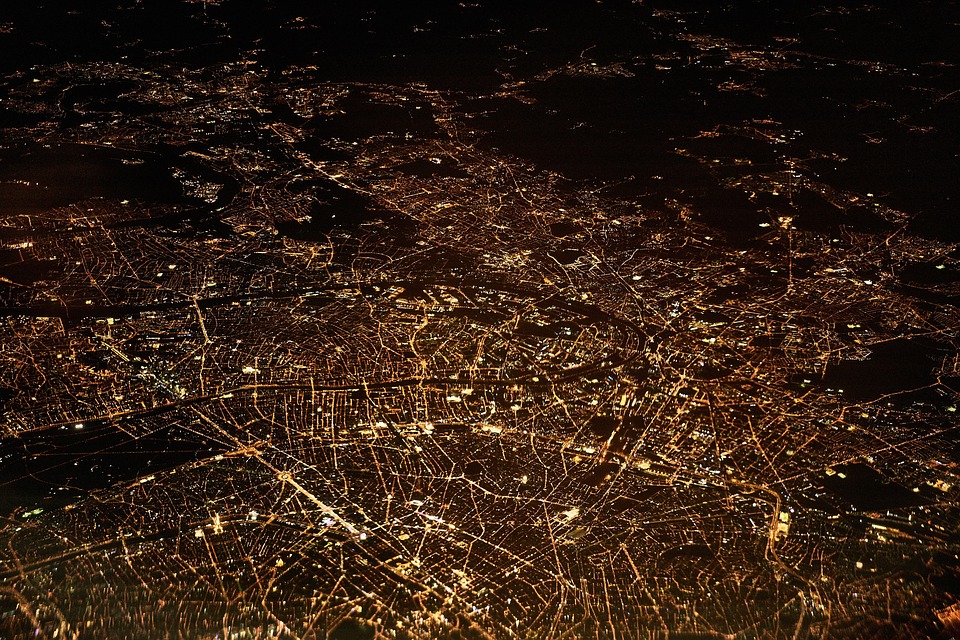

Supplement: Supplementary file 2 [file Data_Sheet_1.zip › Raw Images for Experiment 2/Aerials/aerial11.jpg]

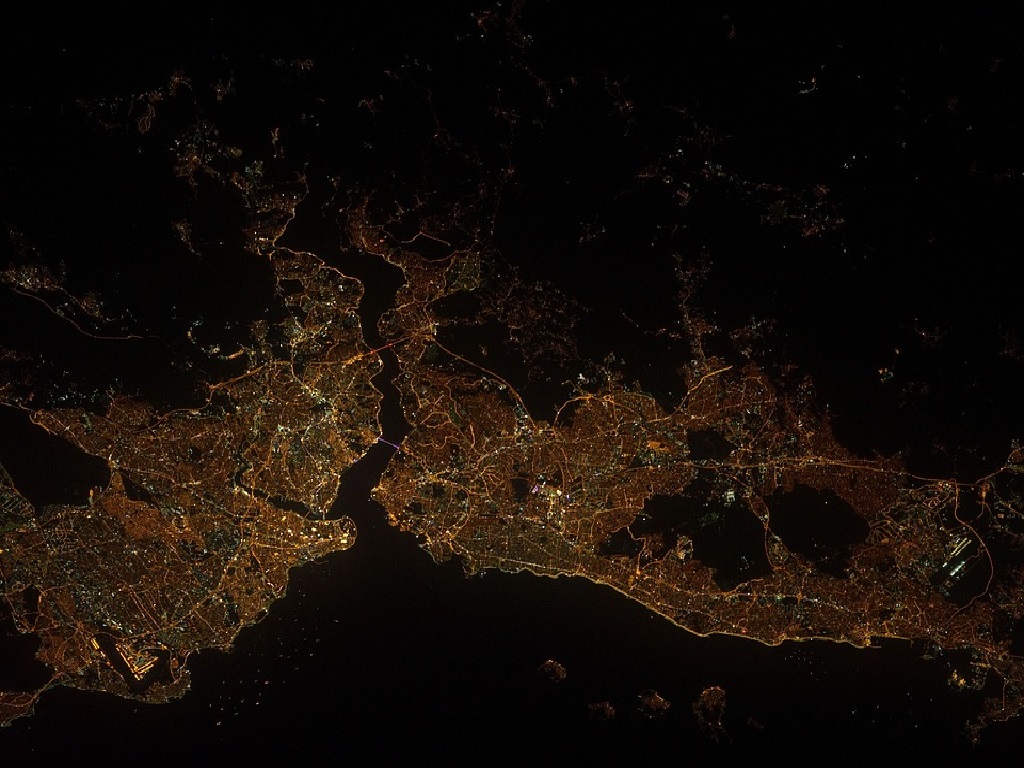

Supplement: Supplementary file 2 [file Data_Sheet_1.zip › Raw Images for Experiment 2/Aerials/aerial12.jpg]

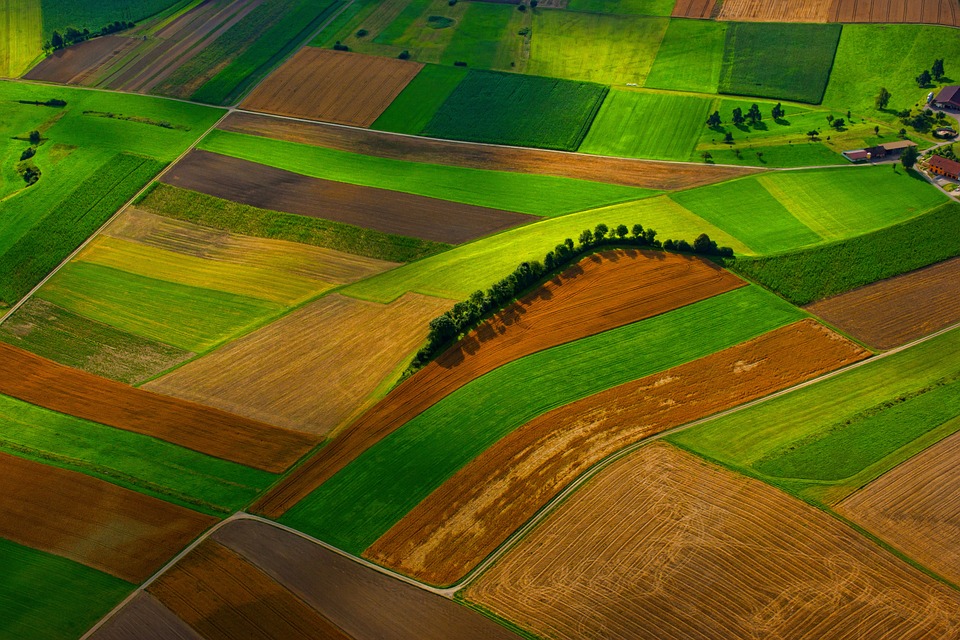

Supplement: Supplementary file 2 [file Data_Sheet_1.zip › Raw Images for Experiment 2/Aerials/aerial15.jpg]

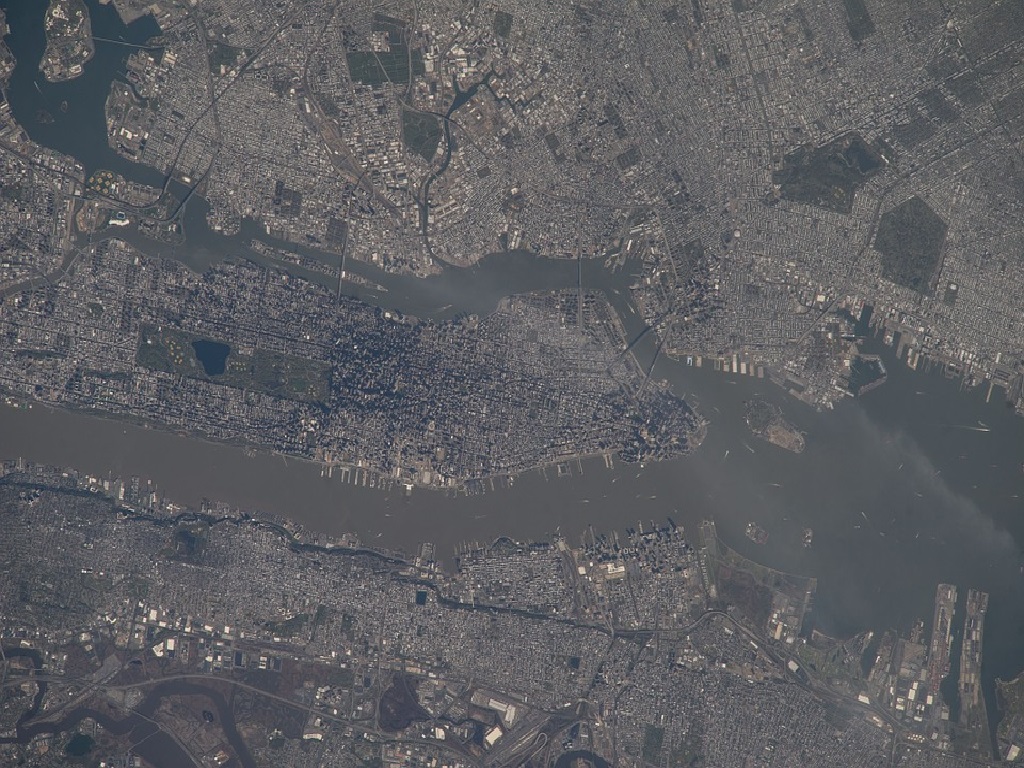

Supplement: Supplementary file 2 [file Data_Sheet_1.zip › Raw Images for Experiment 2/Aerials/aerial16.jpg]

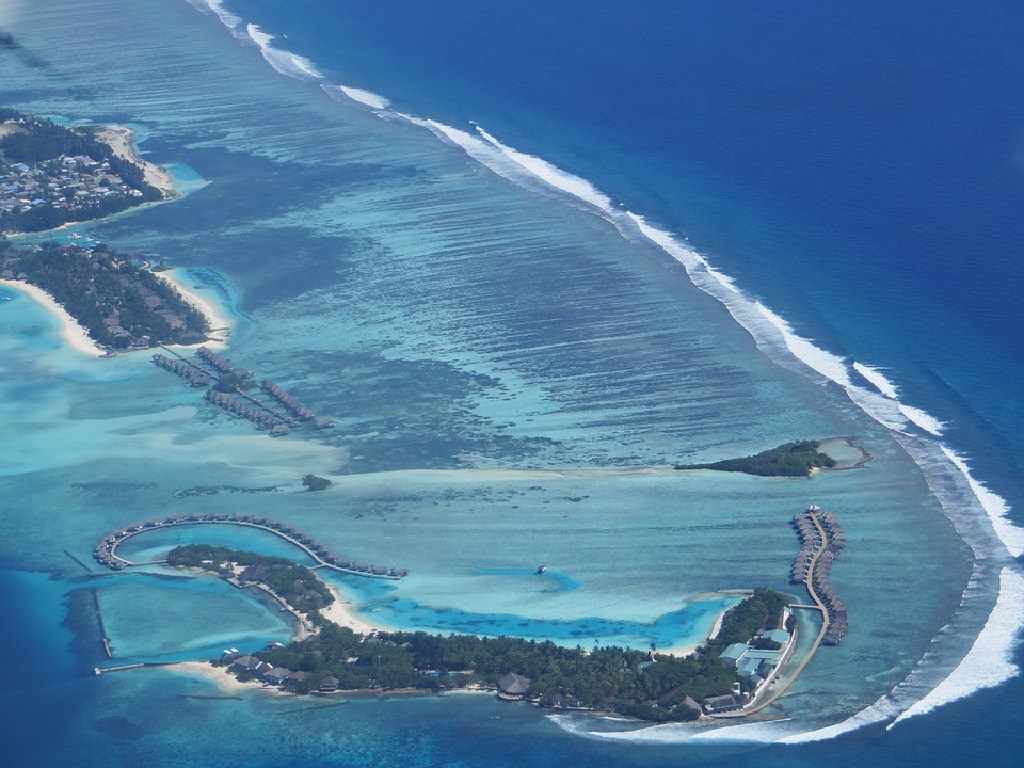

Supplement: Supplementary file 2 [file Data_Sheet_1.zip › Raw Images for Experiment 2/Aerials/aerial18.jpg]

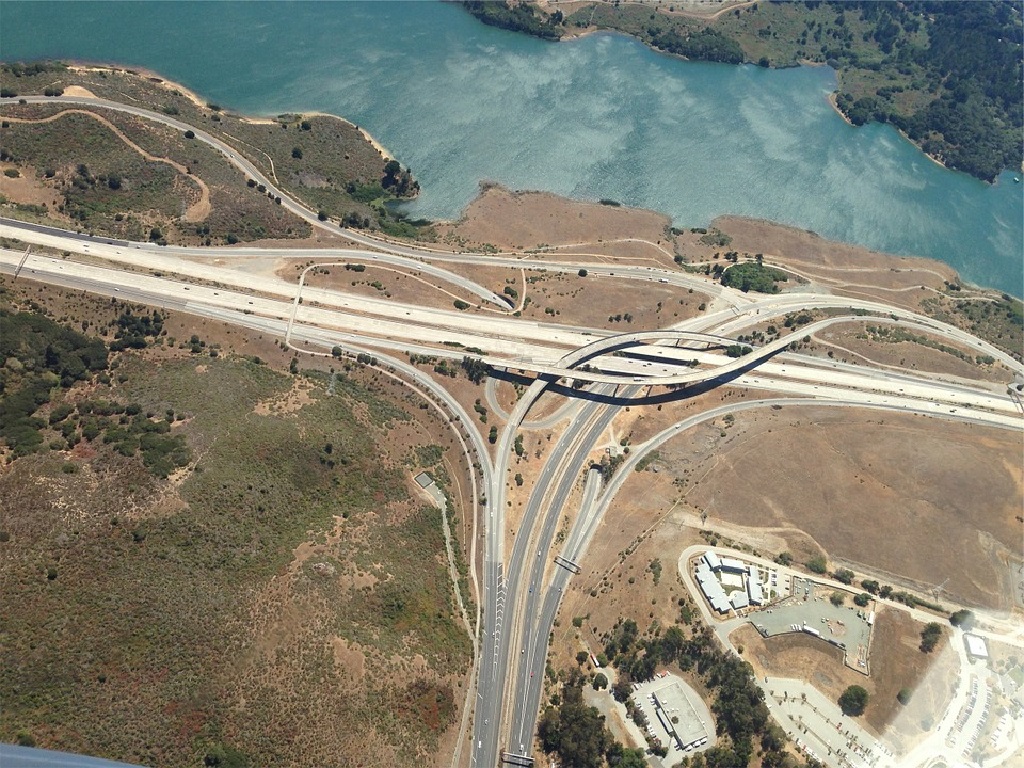

Supplement: Supplementary file 2 [file Data_Sheet_1.zip › Raw Images for Experiment 2/Aerials/aerial20.jpg]

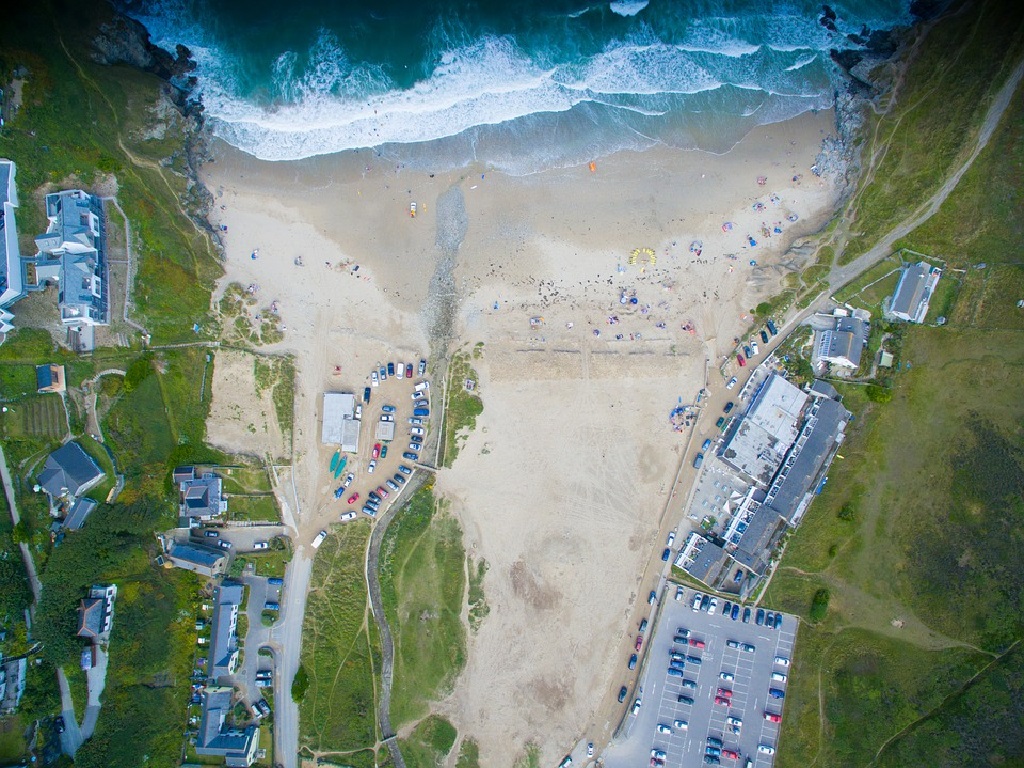

Supplement: Supplementary file 2 [file Data_Sheet_1.zip › Raw Images for Experiment 2/Aerials/aerial21.jpg]

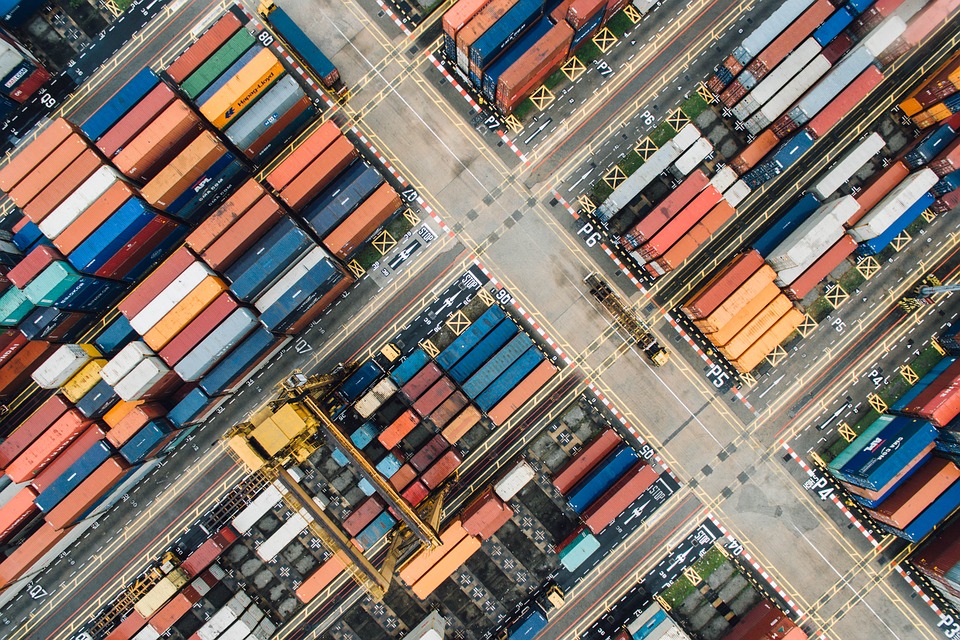

Supplement: Supplementary file 2 [file Data_Sheet_1.zip › Raw Images for Experiment 2/Aerials/aerial22.jpg]

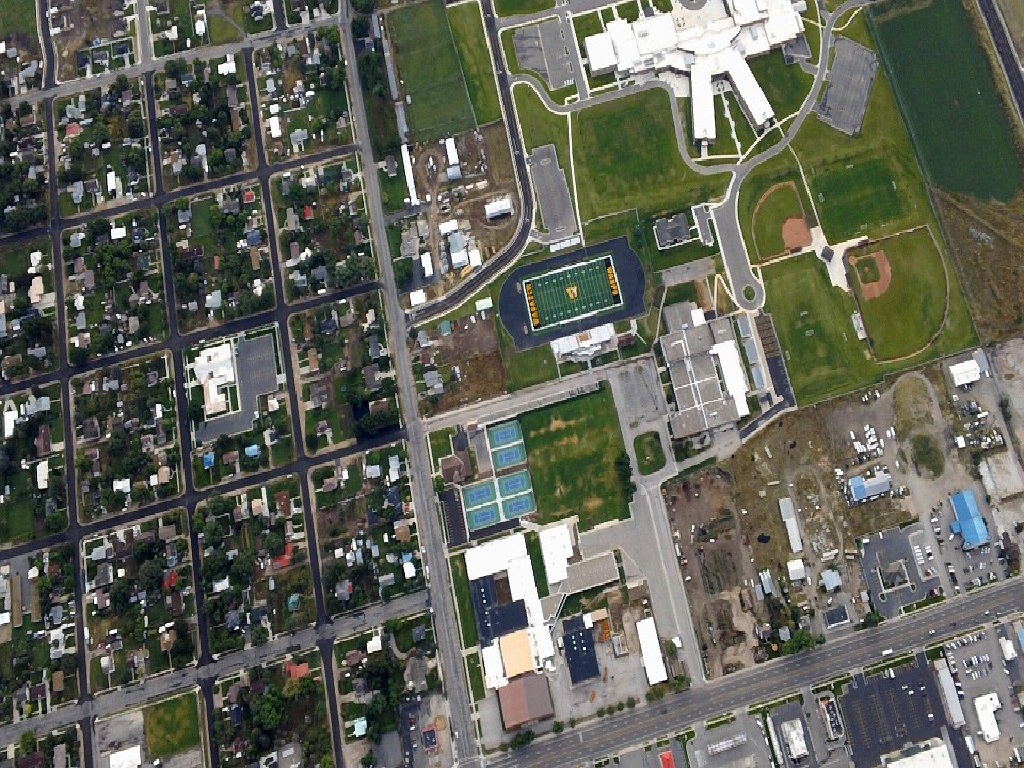

Supplement: Supplementary file 2 [file Data_Sheet_1.zip › Raw Images for Experiment 2/Aerials/aerial24.jpg]

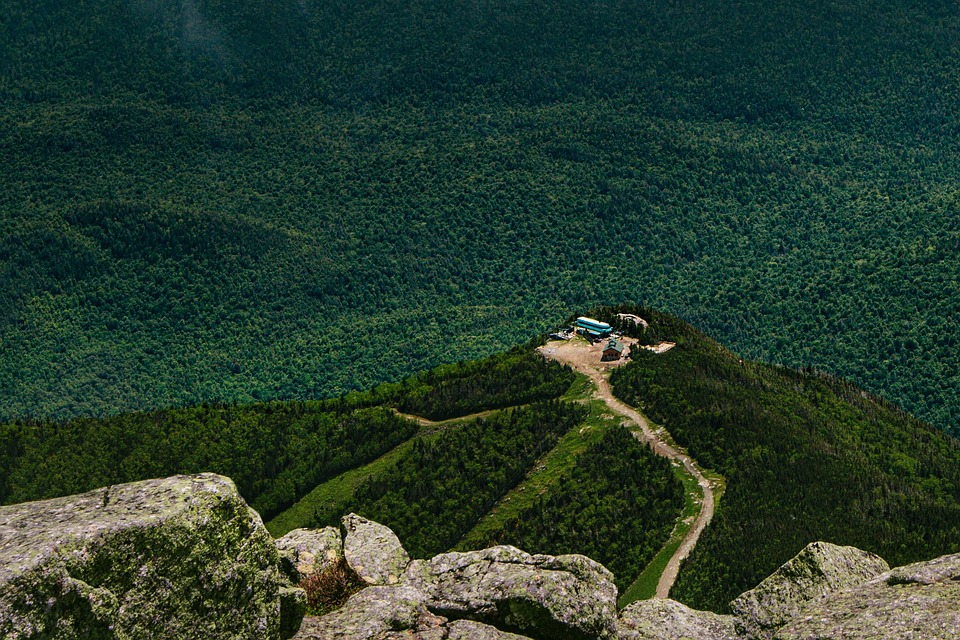

Supplement: Supplementary file 2 [file Data_Sheet_1.zip › Raw Images for Experiment 2/Aerials/aerial25.jpg]

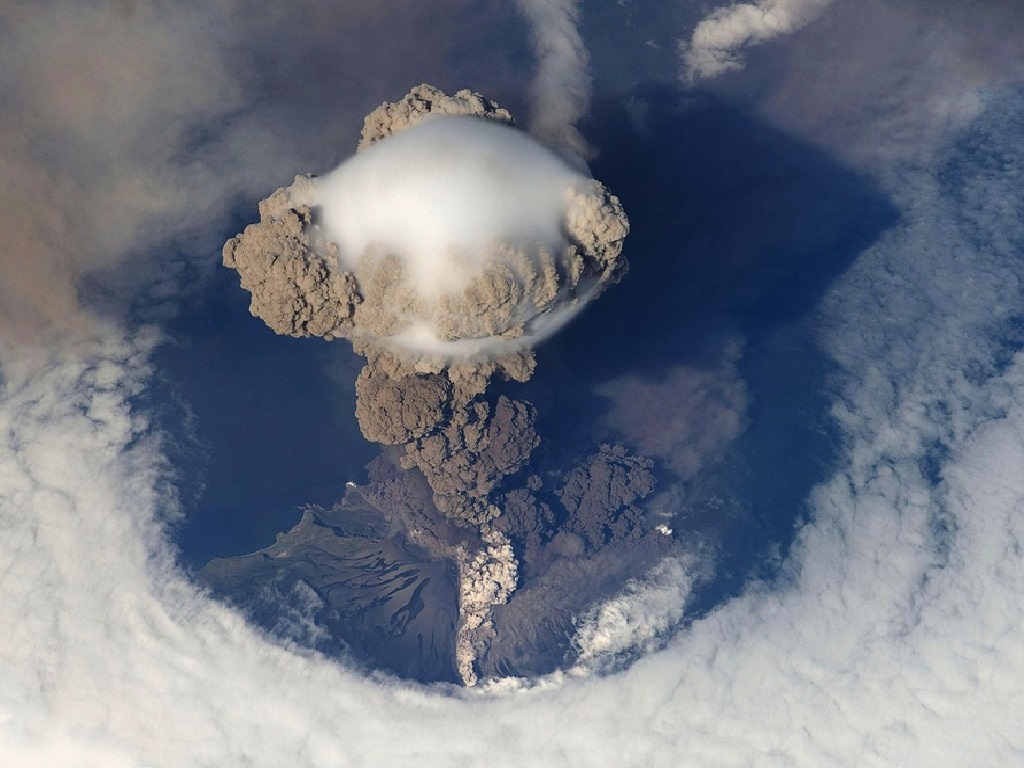

Supplement: Supplementary file 2 [file Data_Sheet_1.zip › Raw Images for Experiment 2/Aerials/aerial26.jpg]

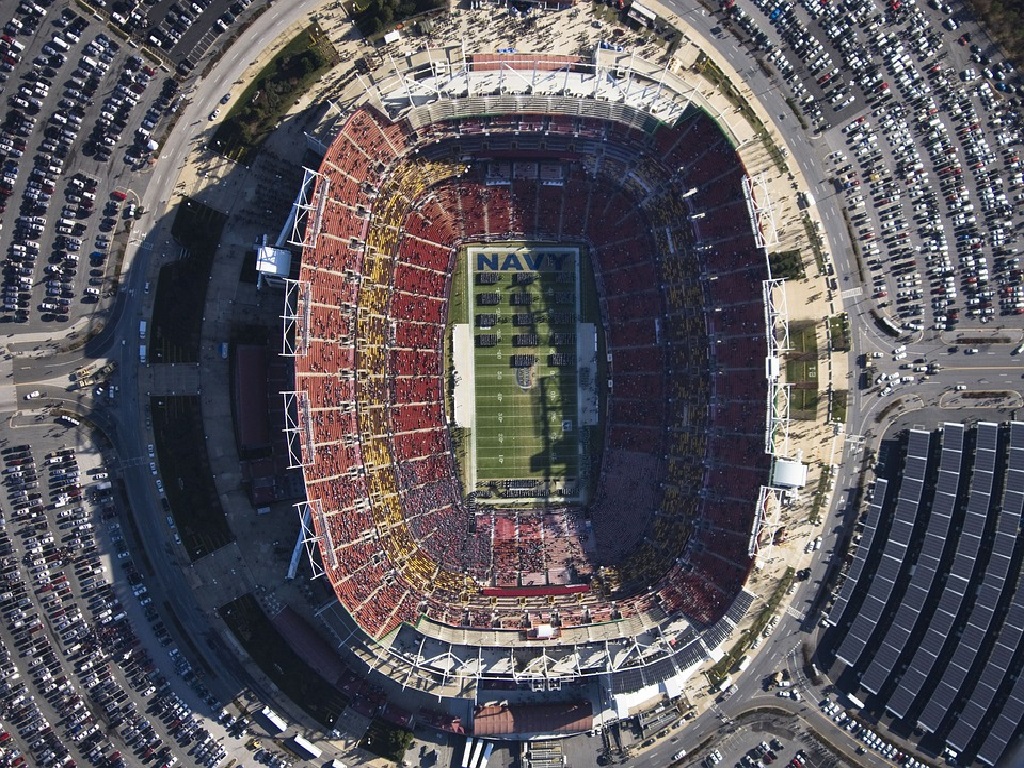

Supplement: Supplementary file 2 [file Data_Sheet_1.zip › Raw Images for Experiment 2/Aerials/aerial28.jpg]

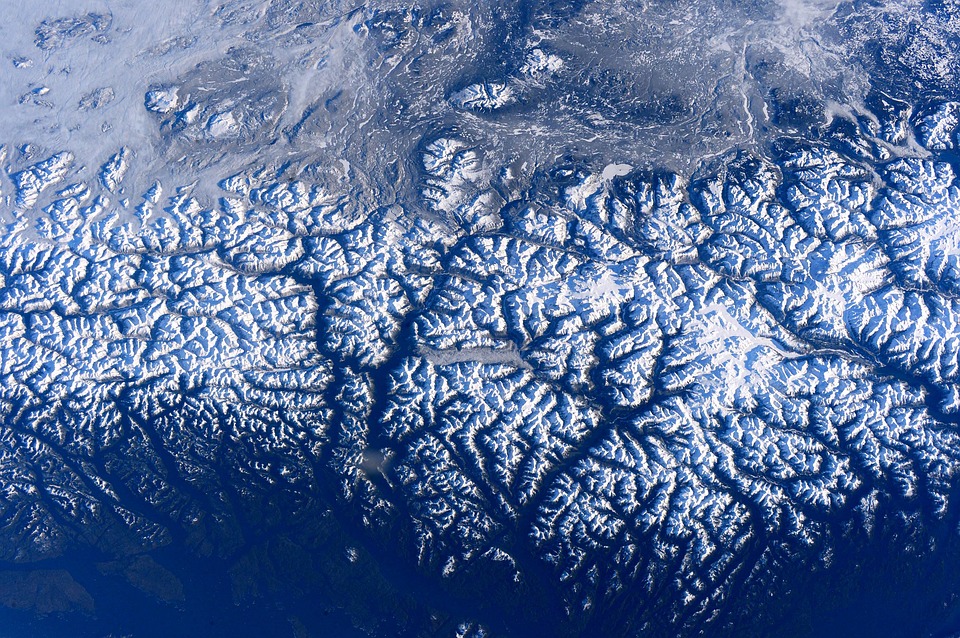

Supplement: Supplementary file 2 [file Data_Sheet_1.zip › Raw Images for Experiment 2/Aerials/aerial30.jpg]

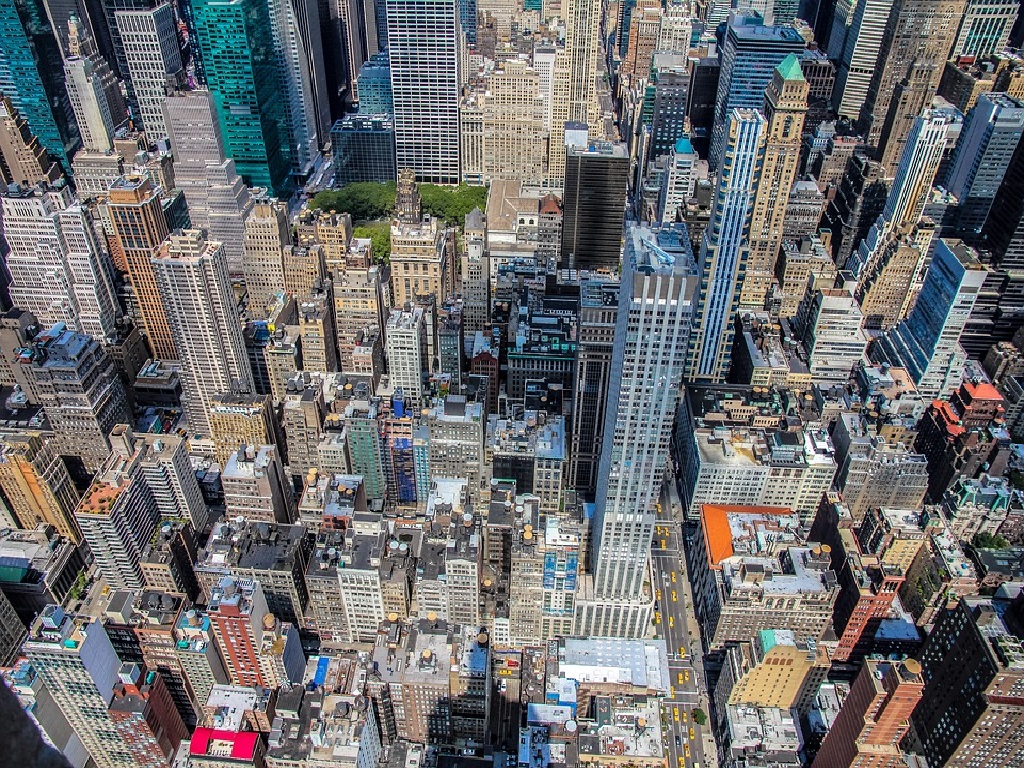

Supplement: Supplementary file 2 [file Data_Sheet_1.zip › Raw Images for Experiment 2/City/city02.jpg]

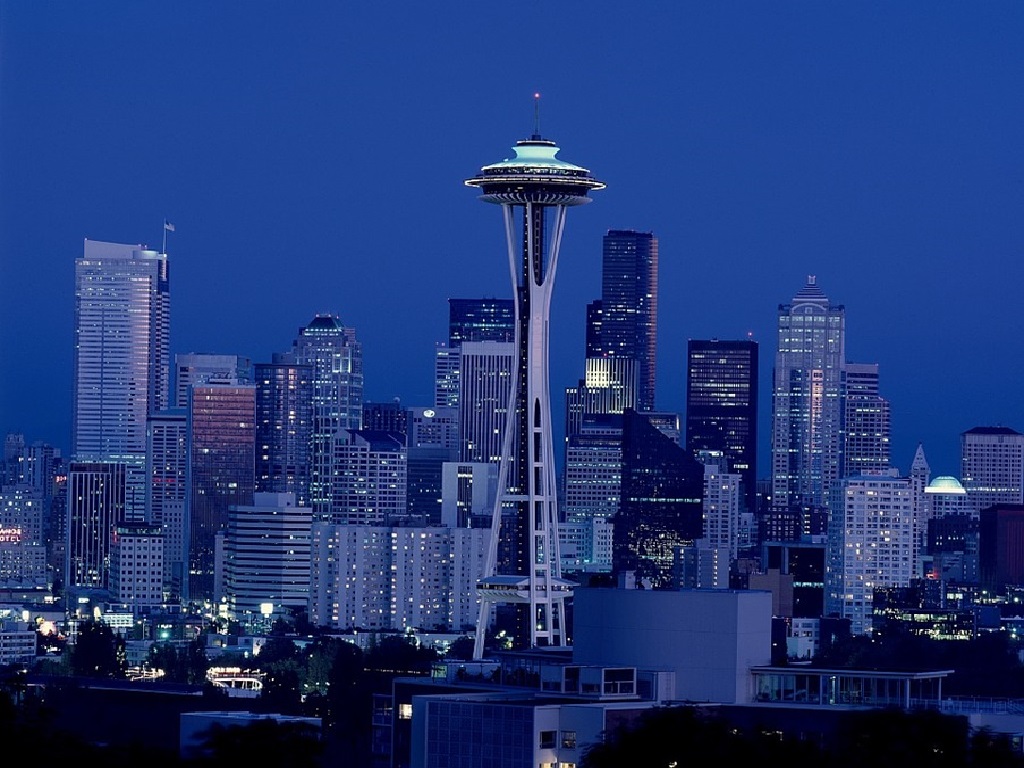

Supplement: Supplementary file 2 [file Data_Sheet_1.zip › Raw Images for Experiment 2/City/city04.jpg]

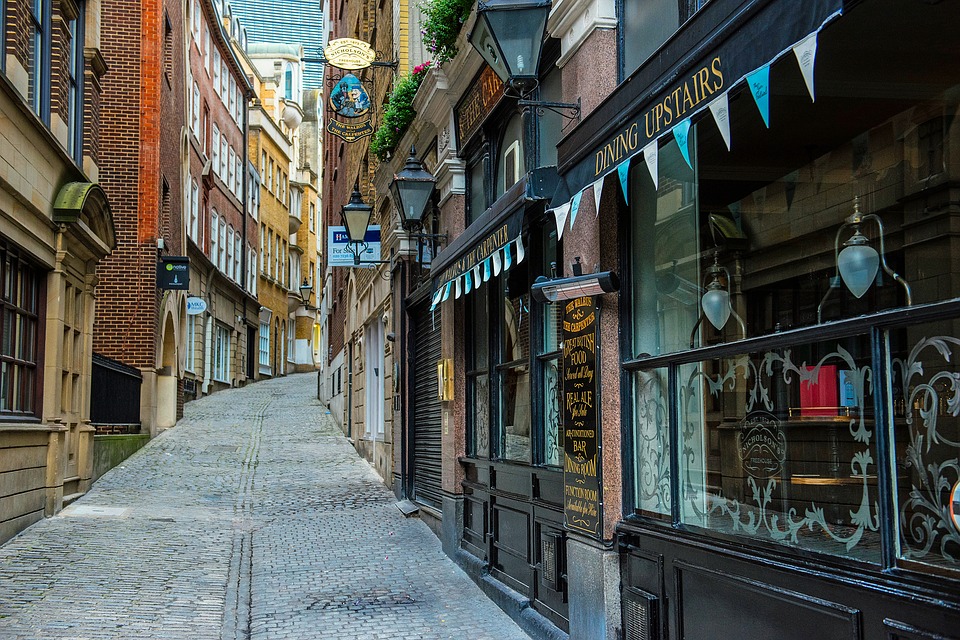

Supplement: Supplementary file 2 [file Data_Sheet_1.zip › Raw Images for Experiment 2/City/city05.jpg]

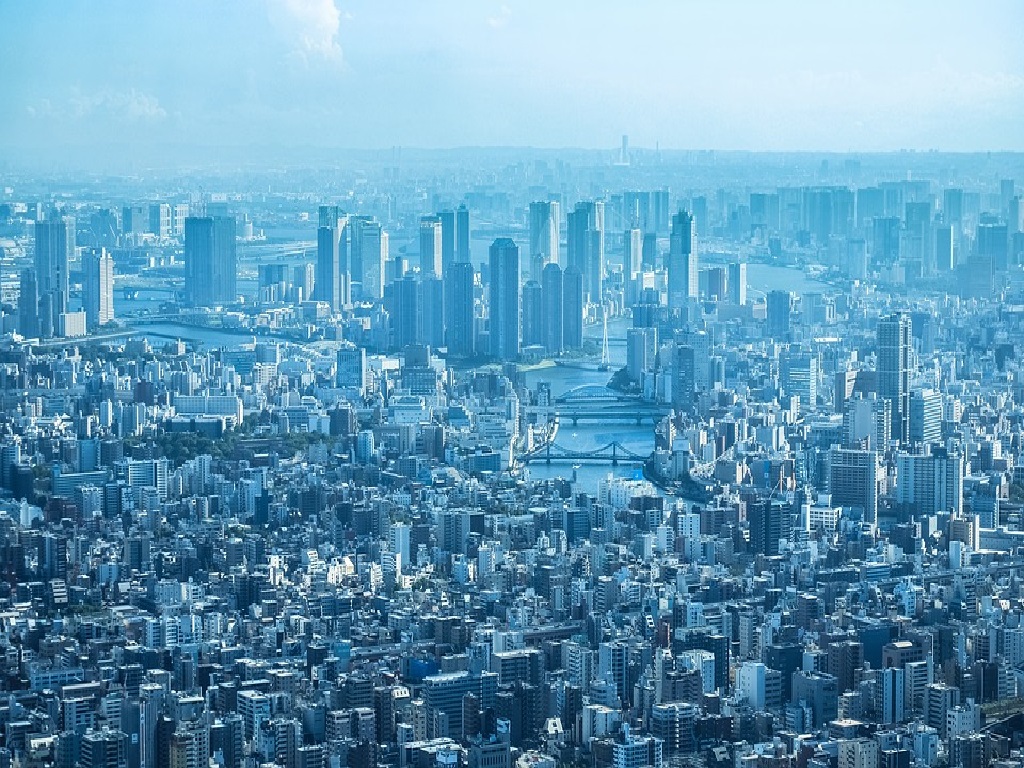

Supplement: Supplementary file 2 [file Data_Sheet_1.zip › Raw Images for Experiment 2/City/city06.jpg]

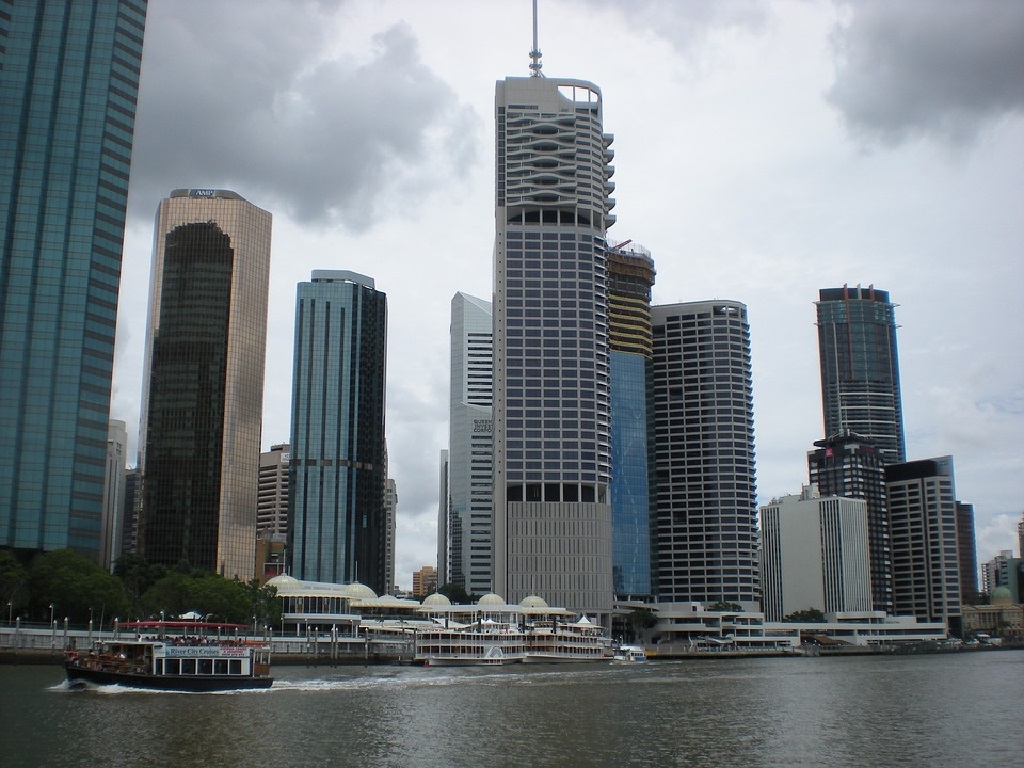

Supplement: Supplementary file 2 [file Data_Sheet_1.zip › Raw Images for Experiment 2/City/city08.jpg]

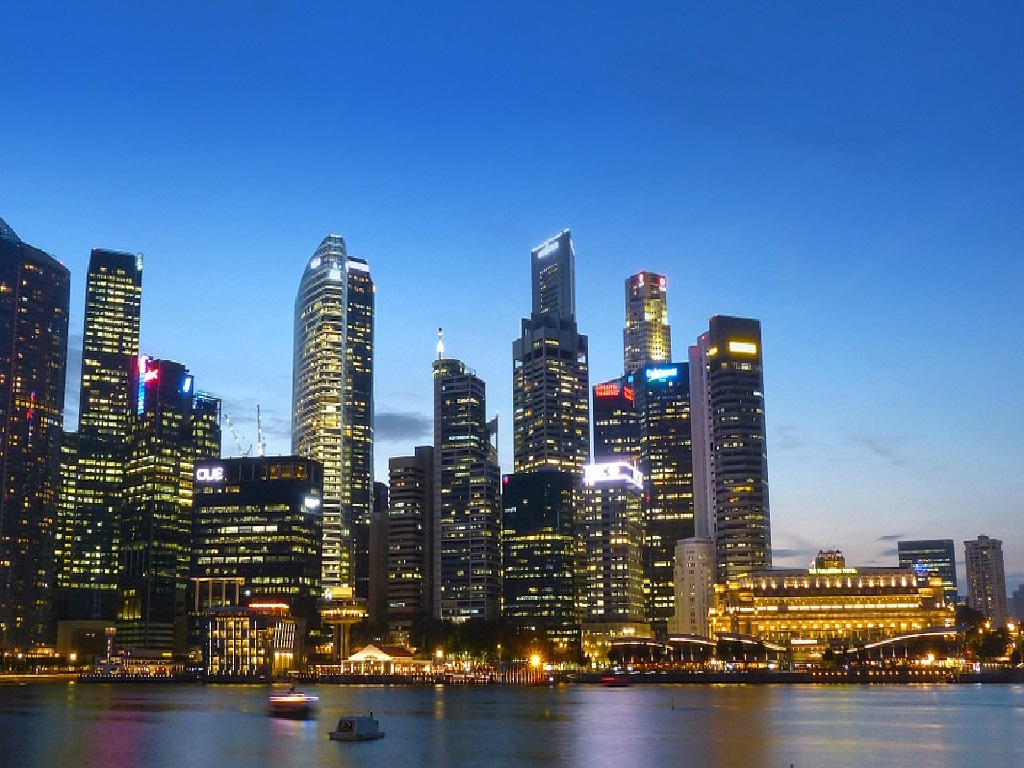

Supplement: Supplementary file 2 [file Data_Sheet_1.zip › Raw Images for Experiment 2/City/city10.jpg]

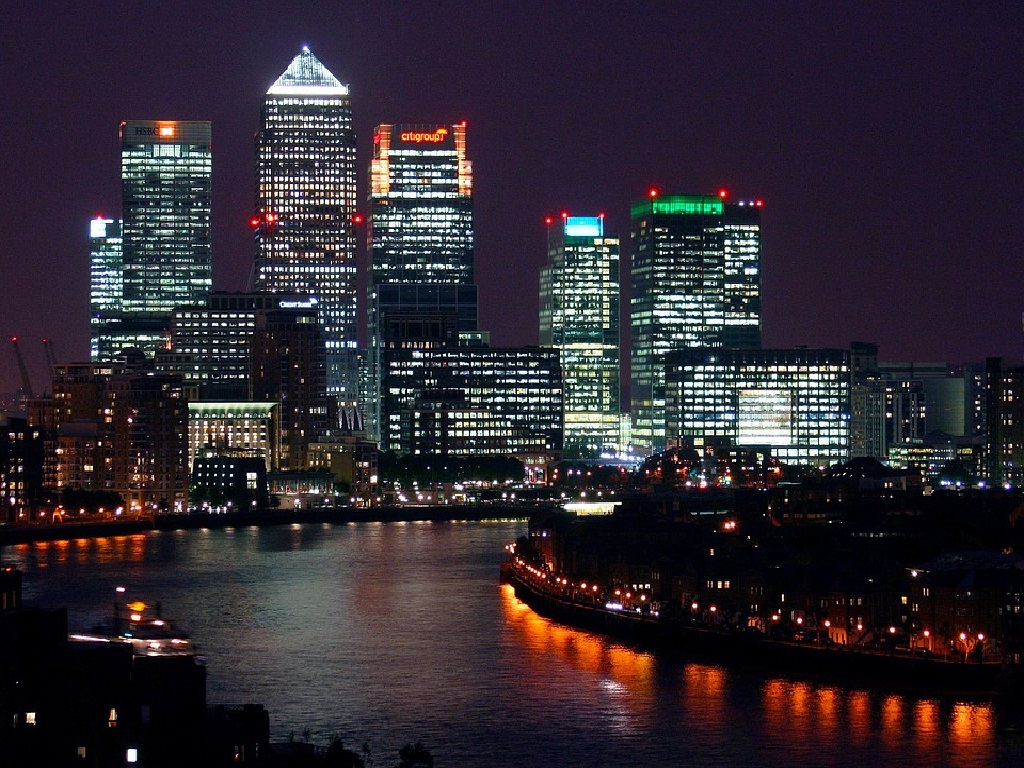

Supplement: Supplementary file 2 [file Data_Sheet_1.zip › Raw Images for Experiment 2/City/city13.jpg]

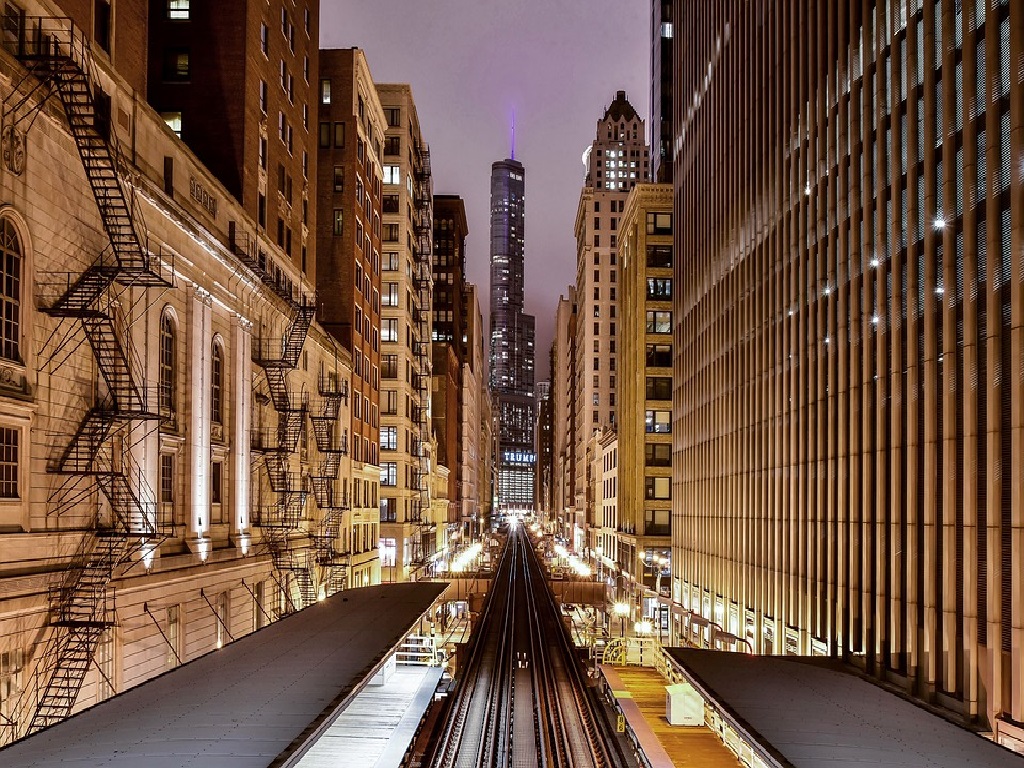

Supplement: Supplementary file 2 [file Data_Sheet_1.zip › Raw Images for Experiment 2/City/city15.jpg]

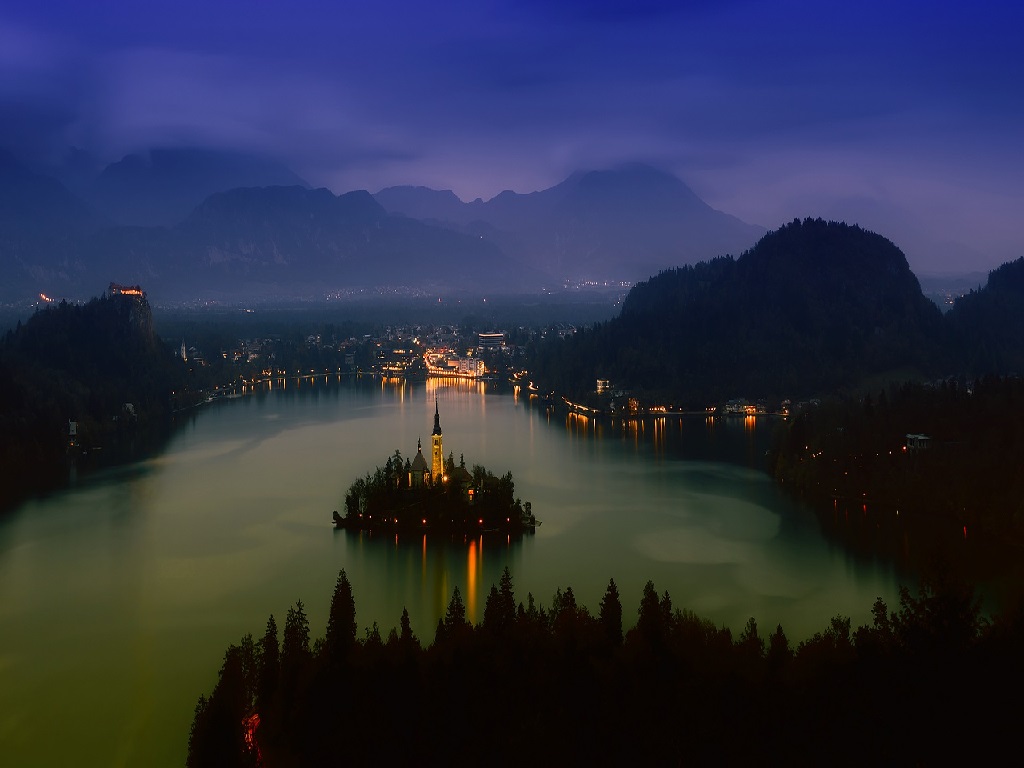

Supplement: Supplementary file 2 [file Data_Sheet_1.zip › Raw Images for Experiment 2/City/city17.jpg]

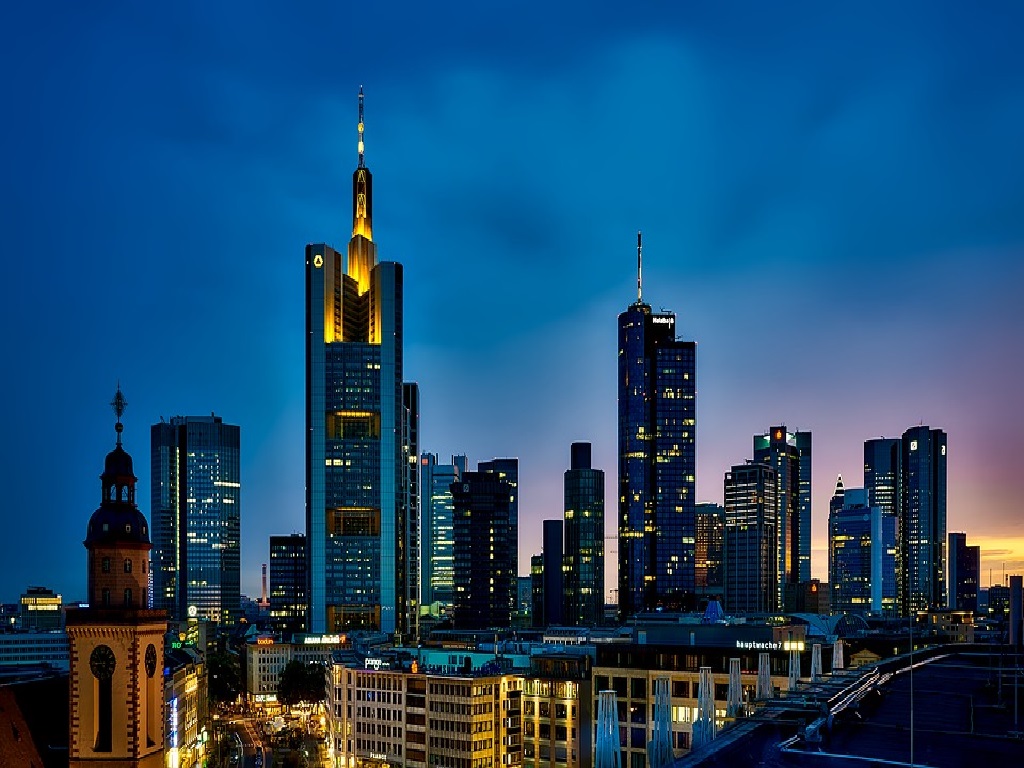

Supplement: Supplementary file 2 [file Data_Sheet_1.zip › Raw Images for Experiment 2/City/city19.jpg]

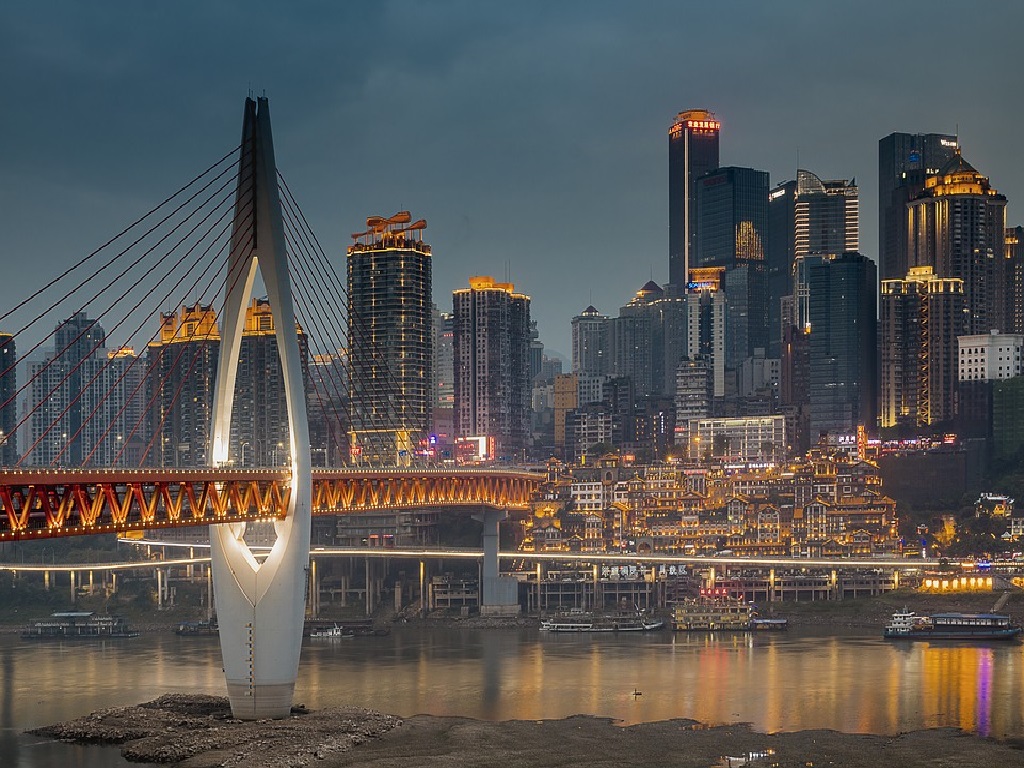

Supplement: Supplementary file 2 [file Data_Sheet_1.zip › Raw Images for Experiment 2/City/city24.jpg]

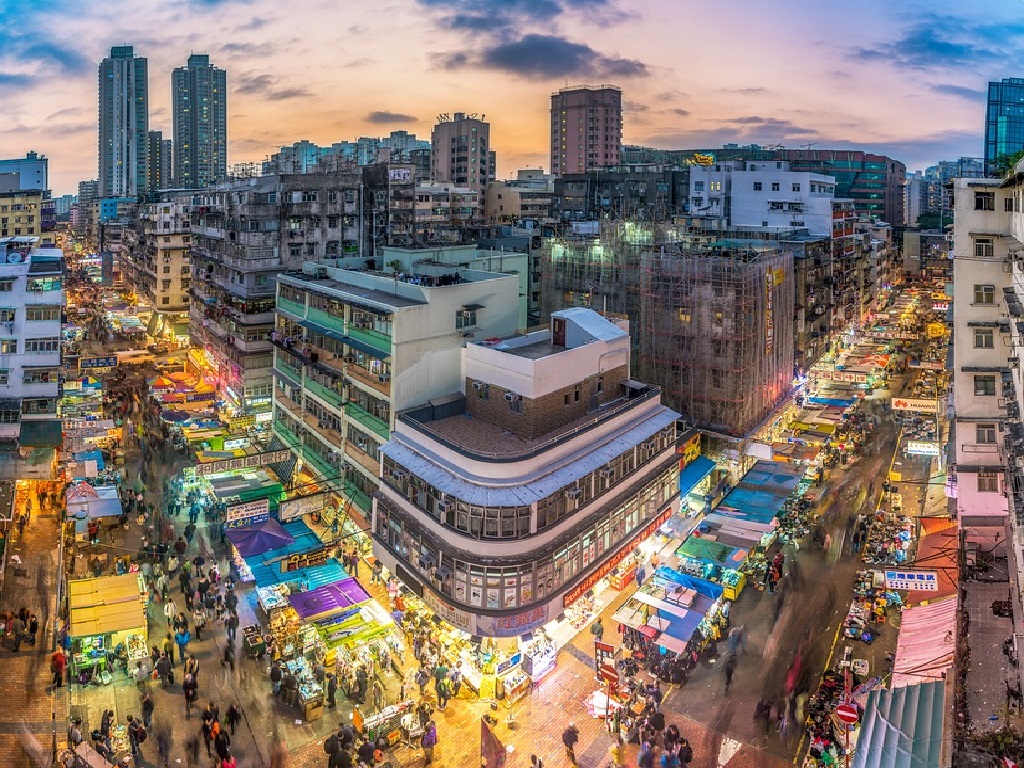

Supplement: Supplementary file 2 [file Data_Sheet_1.zip › Raw Images for Experiment 2/City/city26.jpg]

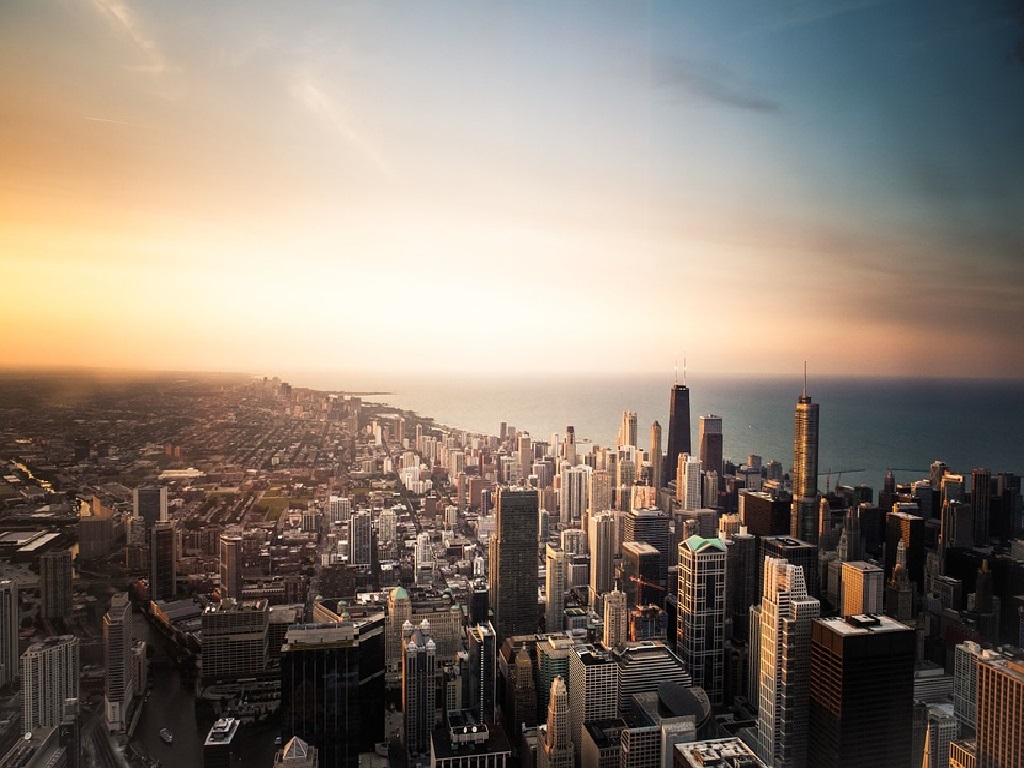

Supplement: Supplementary file 2 [file Data_Sheet_1.zip › Raw Images for Experiment 2/City/city28.jpg]

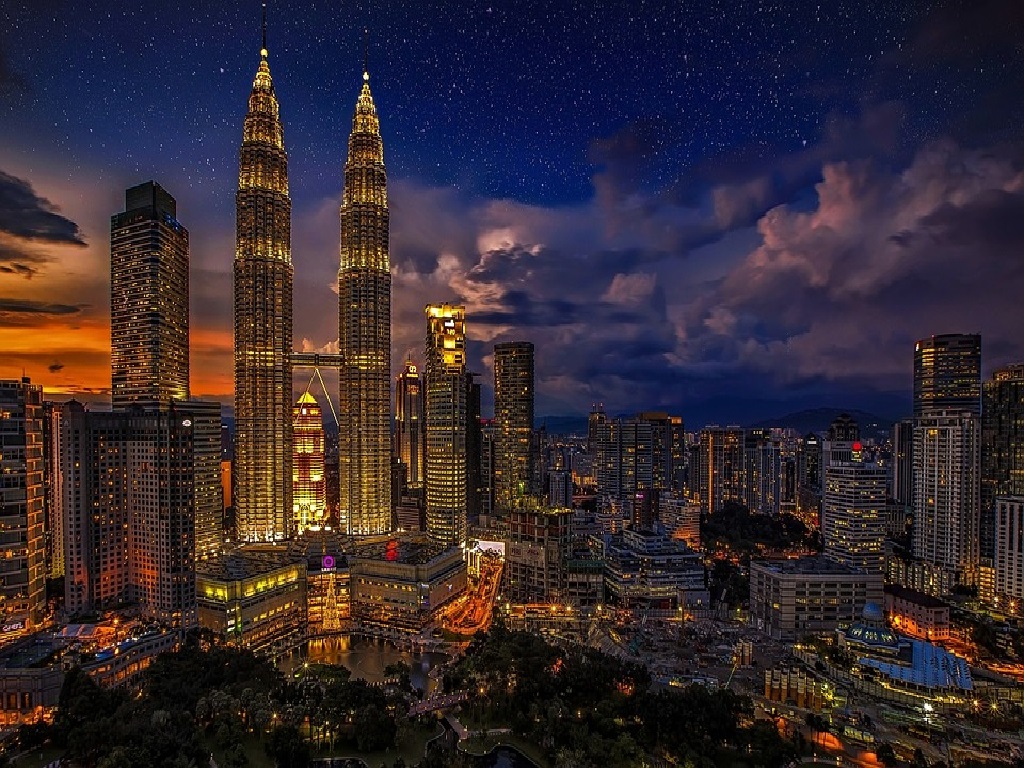

Supplement: Supplementary file 2 [file Data_Sheet_1.zip › Raw Images for Experiment 2/City/city29.jpg]

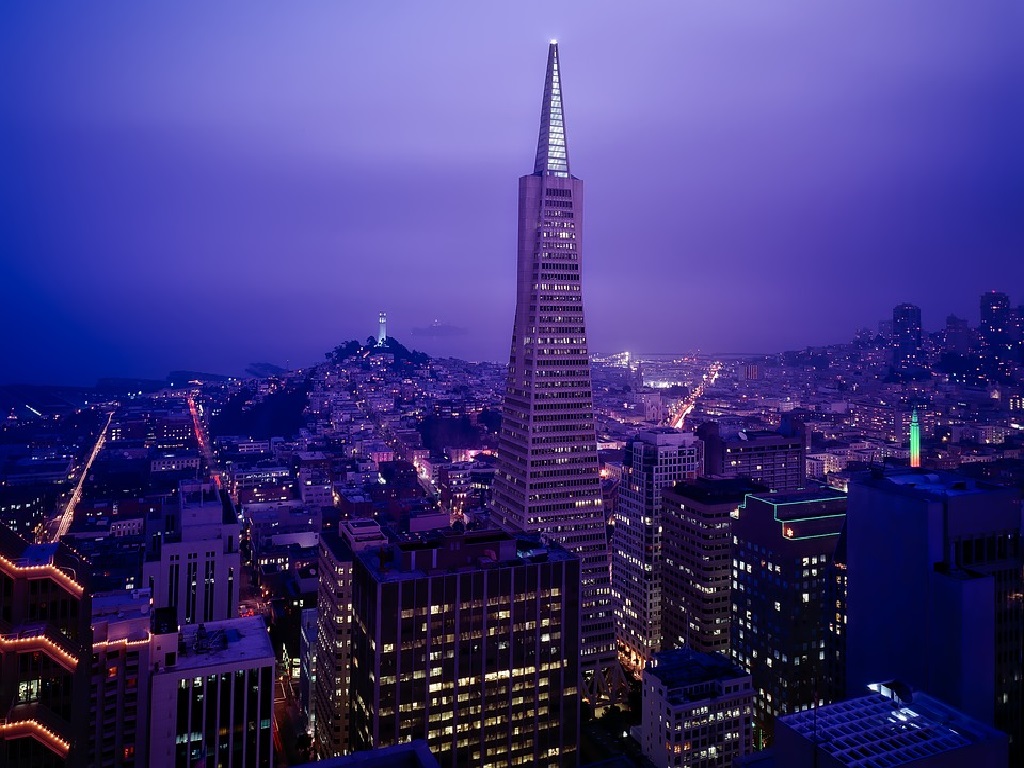

Supplement: Supplementary file 2 [file Data_Sheet_1.zip › Raw Images for Experiment 2/City/city30.jpg]

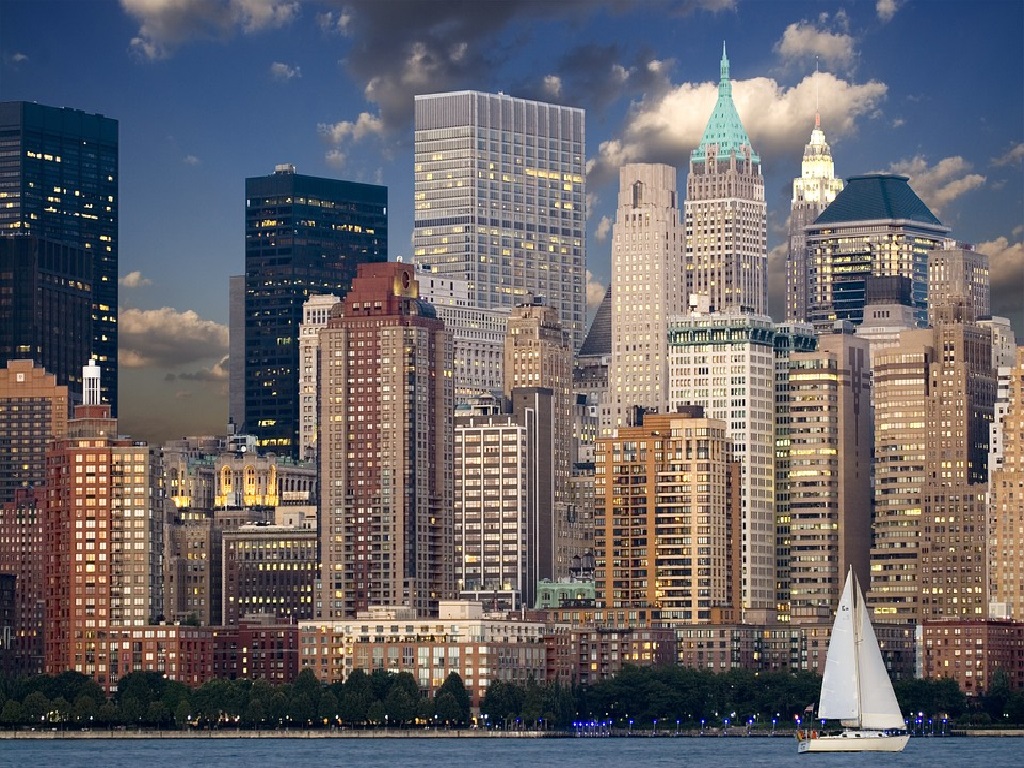

Supplement: Supplementary file 2 [file Data_Sheet_1.zip › Raw Images for Experiment 2/City/city32.jpg]

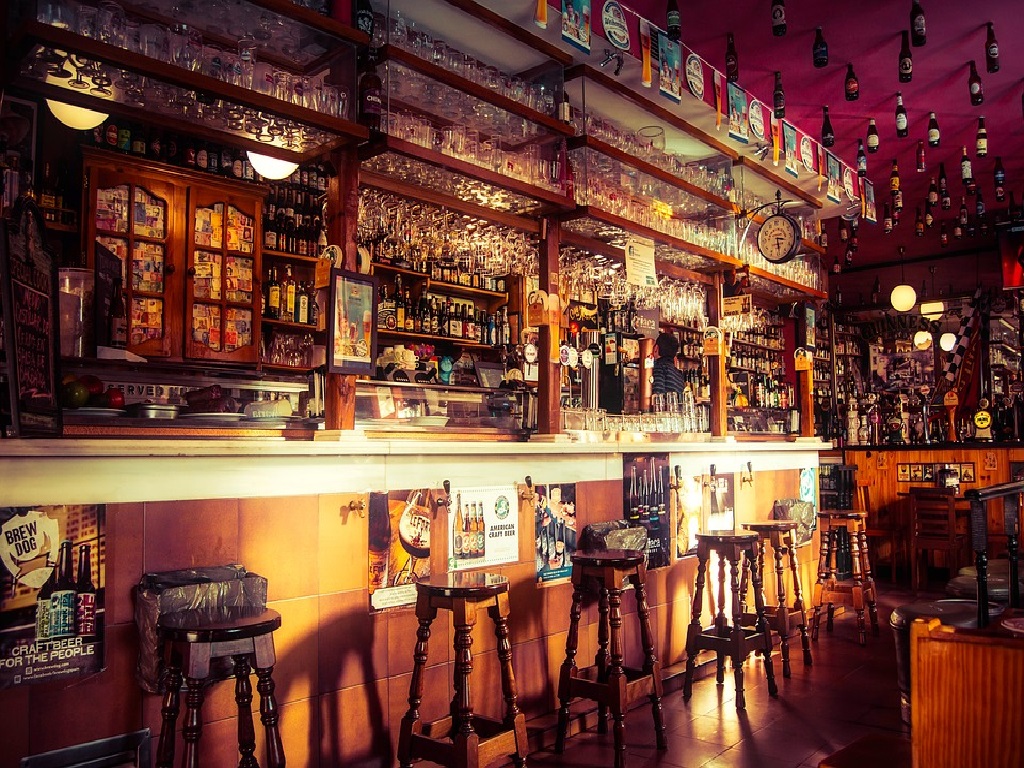

Supplement: Supplementary file 2 [file Data_Sheet_1.zip › Raw Images for Experiment 2/Indoors/indoor01.jpg]

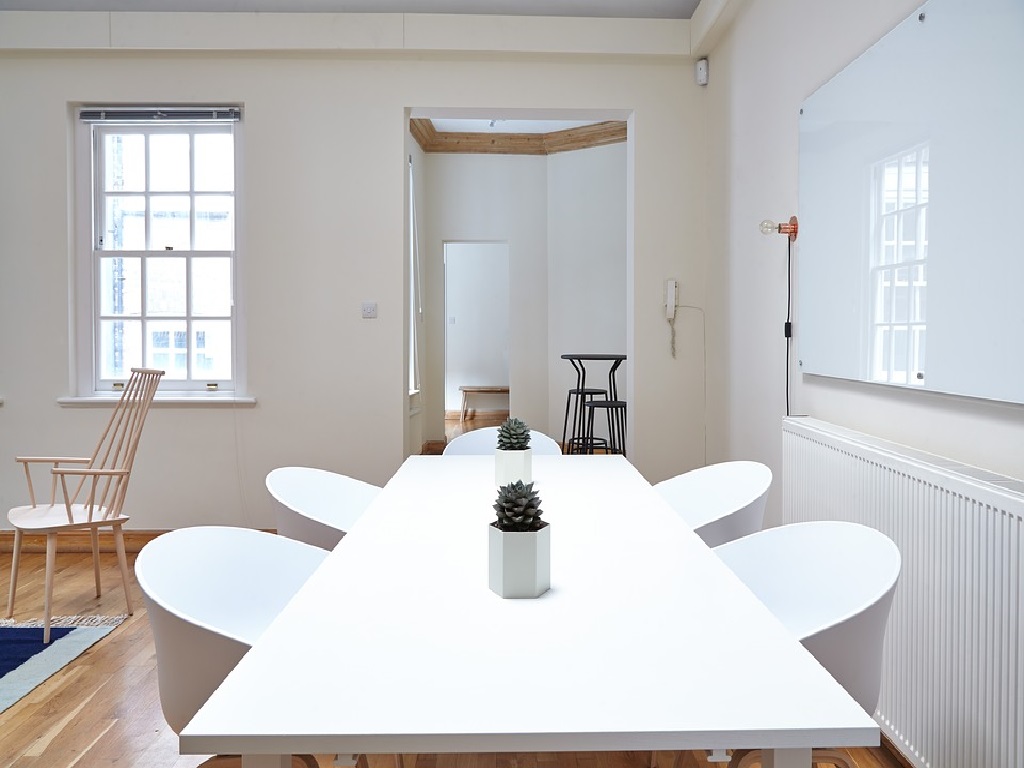

Supplement: Supplementary file 2 [file Data_Sheet_1.zip › Raw Images for Experiment 2/Indoors/indoor02.jpg]

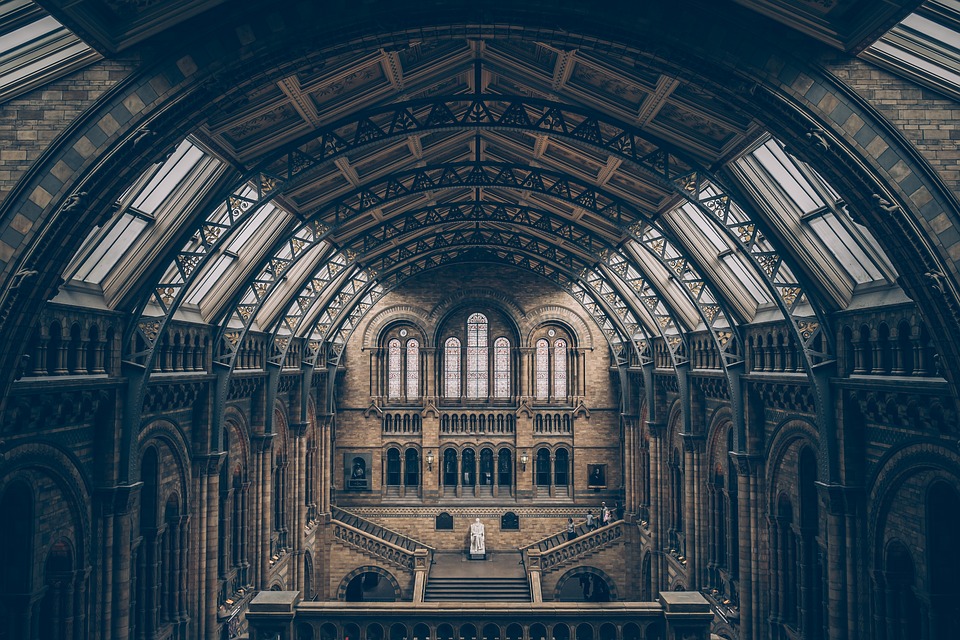

Supplement: Supplementary file 2 [file Data_Sheet_1.zip › Raw Images for Experiment 2/Indoors/indoor03.jpg]

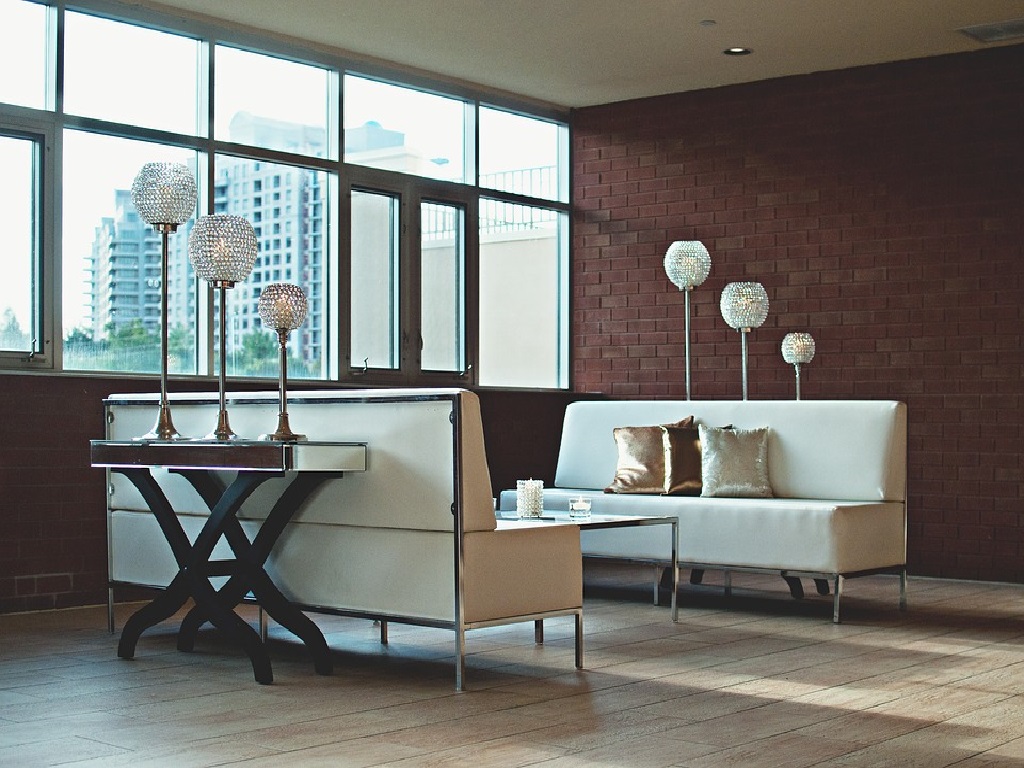

Supplement: Supplementary file 2 [file Data_Sheet_1.zip › Raw Images for Experiment 2/Indoors/indoor04.jpg]

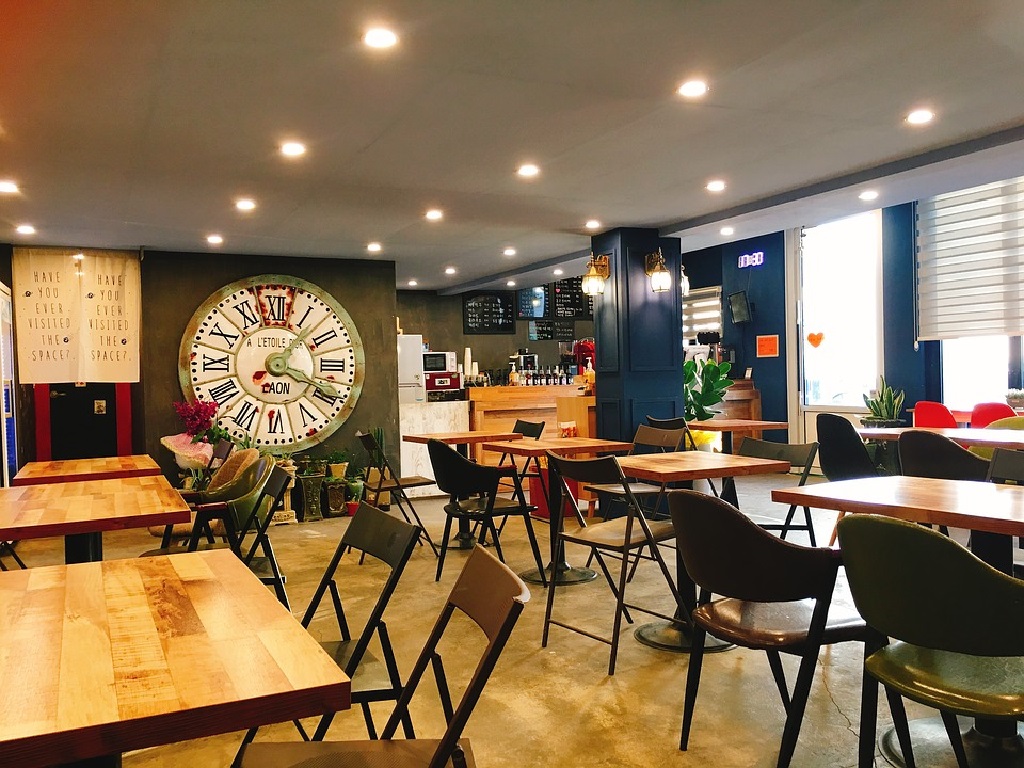

Supplement: Supplementary file 2 [file Data_Sheet_1.zip › Raw Images for Experiment 2/Indoors/indoor06.jpg]

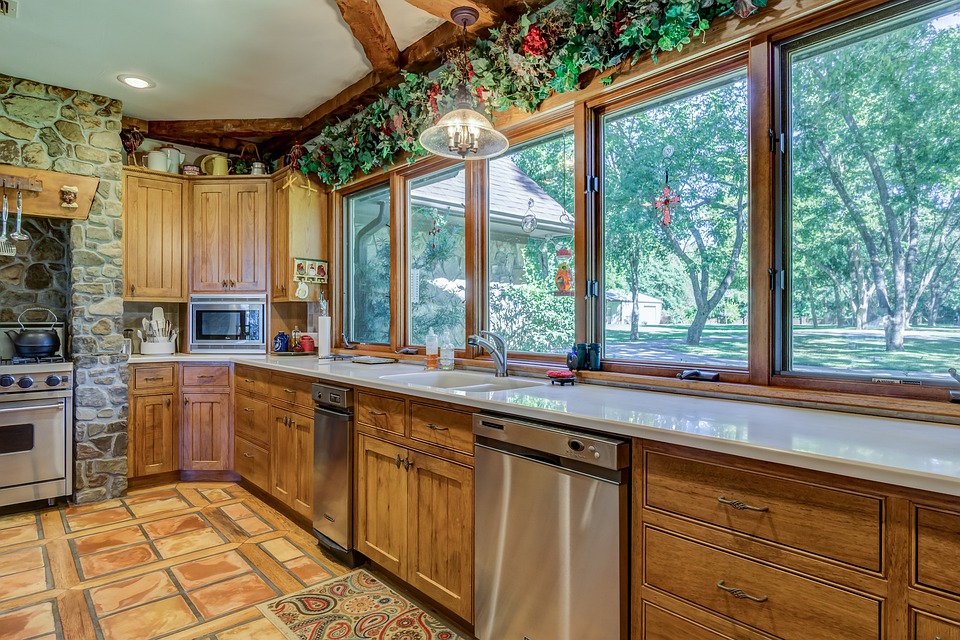

Supplement: Supplementary file 2 [file Data_Sheet_1.zip › Raw Images for Experiment 2/Indoors/indoor08.jpg]

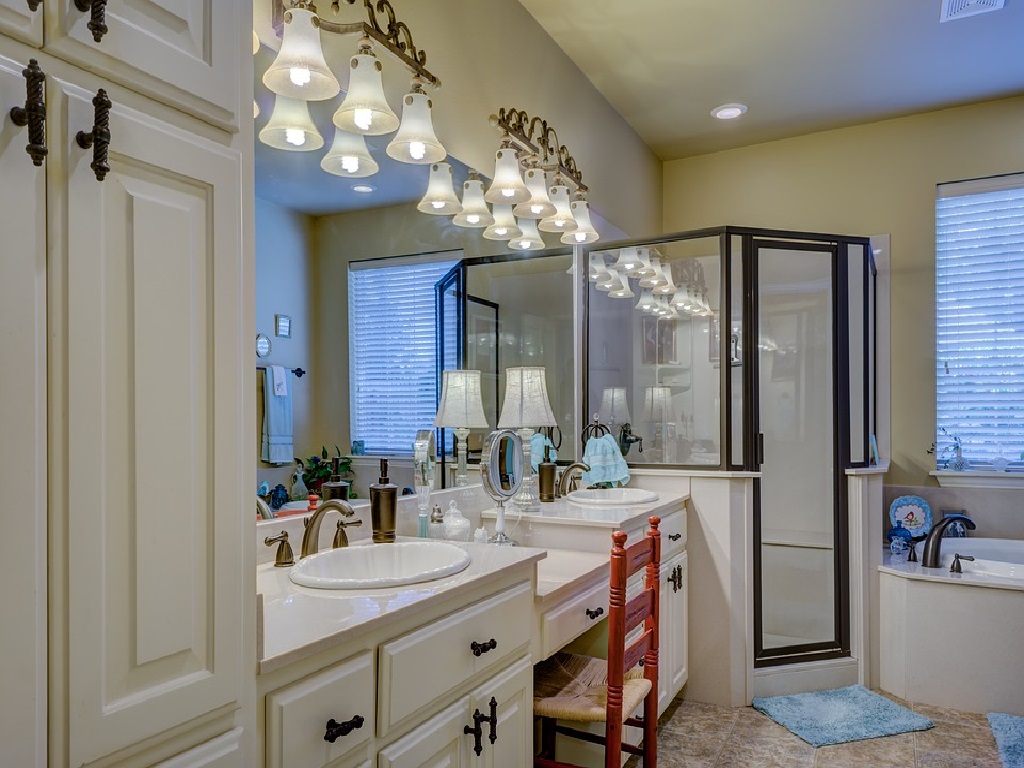

Supplement: Supplementary file 2 [file Data_Sheet_1.zip › Raw Images for Experiment 2/Indoors/indoor09.jpg]

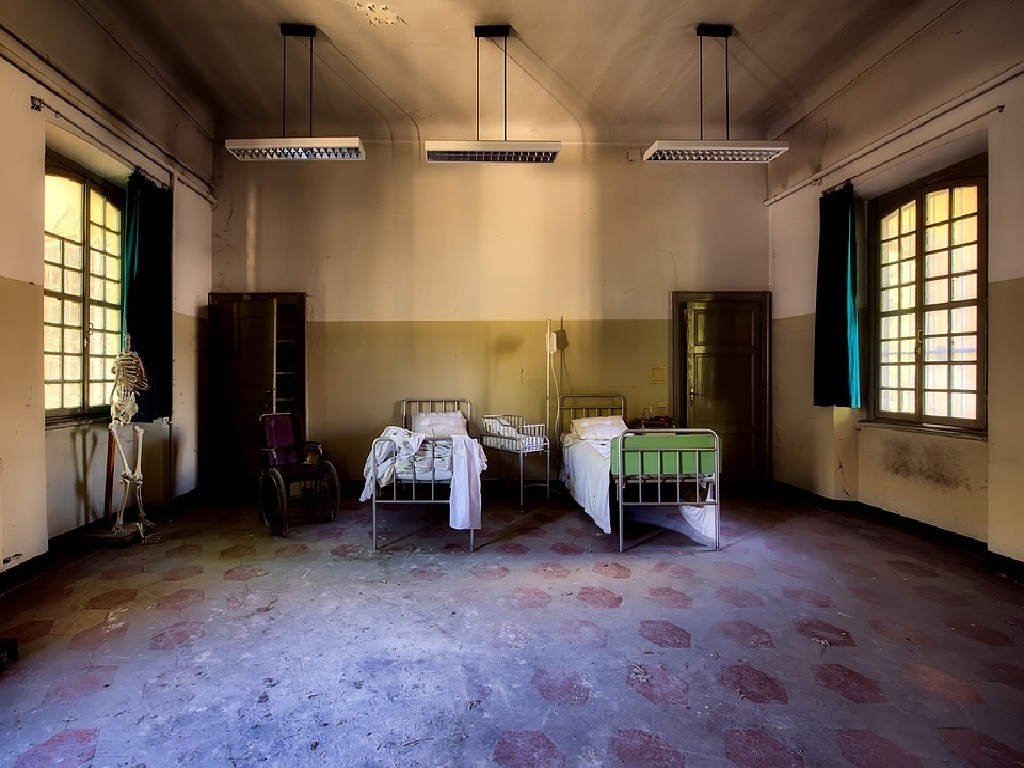

Supplement: Supplementary file 2 [file Data_Sheet_1.zip › Raw Images for Experiment 2/Indoors/indoor11.jpg]

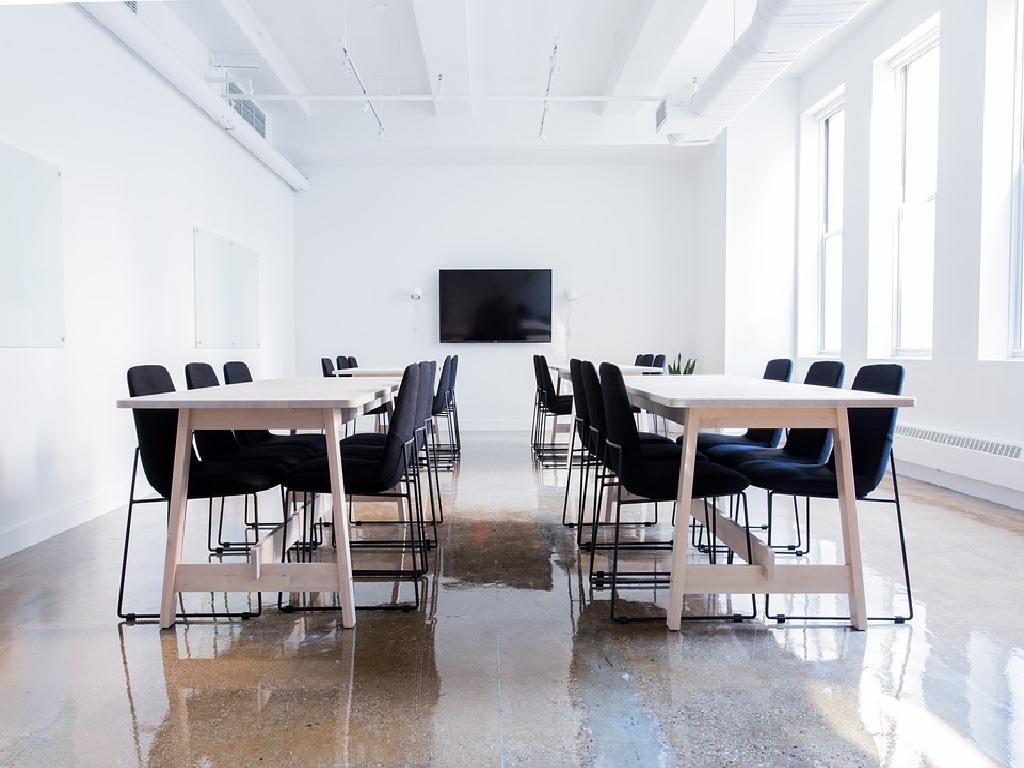

Supplement: Supplementary file 2 [file Data_Sheet_1.zip › Raw Images for Experiment 2/Indoors/indoor13.jpg]

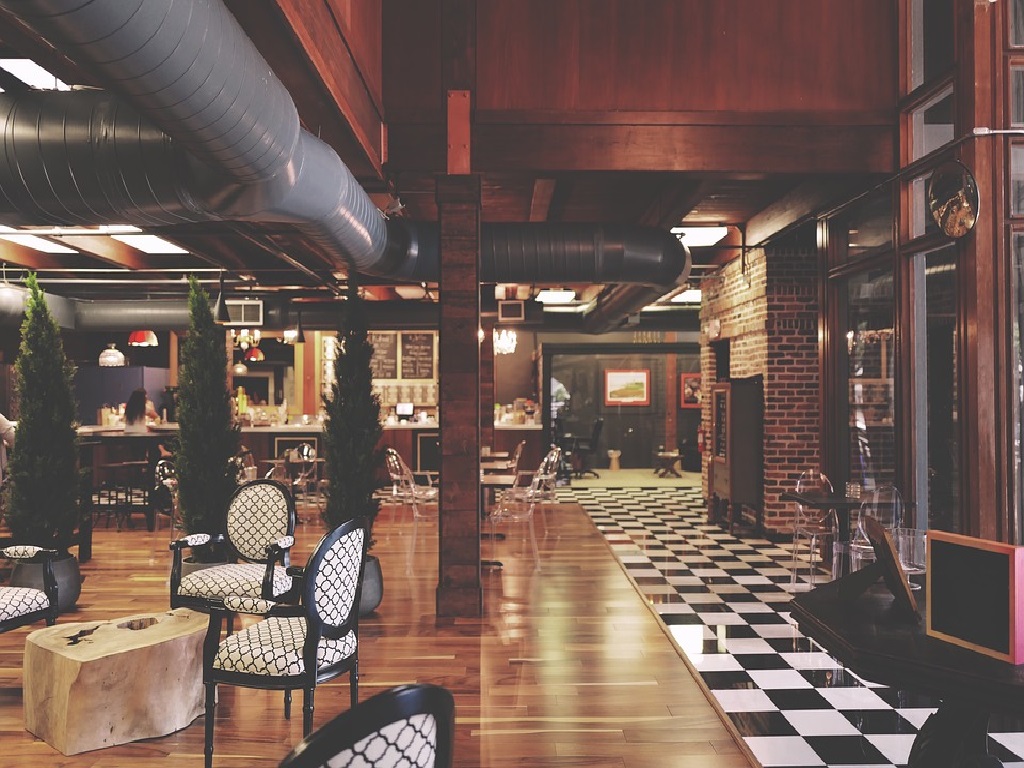

Supplement: Supplementary file 2 [file Data_Sheet_1.zip › Raw Images for Experiment 2/Indoors/indoor15.jpg]

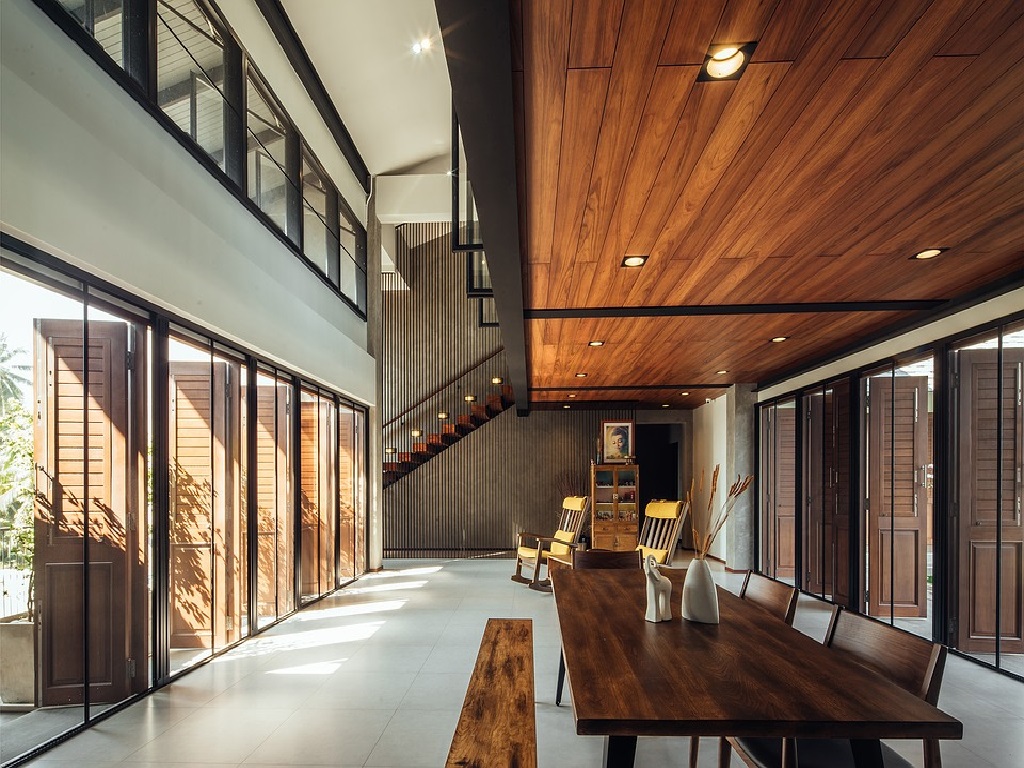

Supplement: Supplementary file 2 [file Data_Sheet_1.zip › Raw Images for Experiment 2/Indoors/indoor19.jpg]

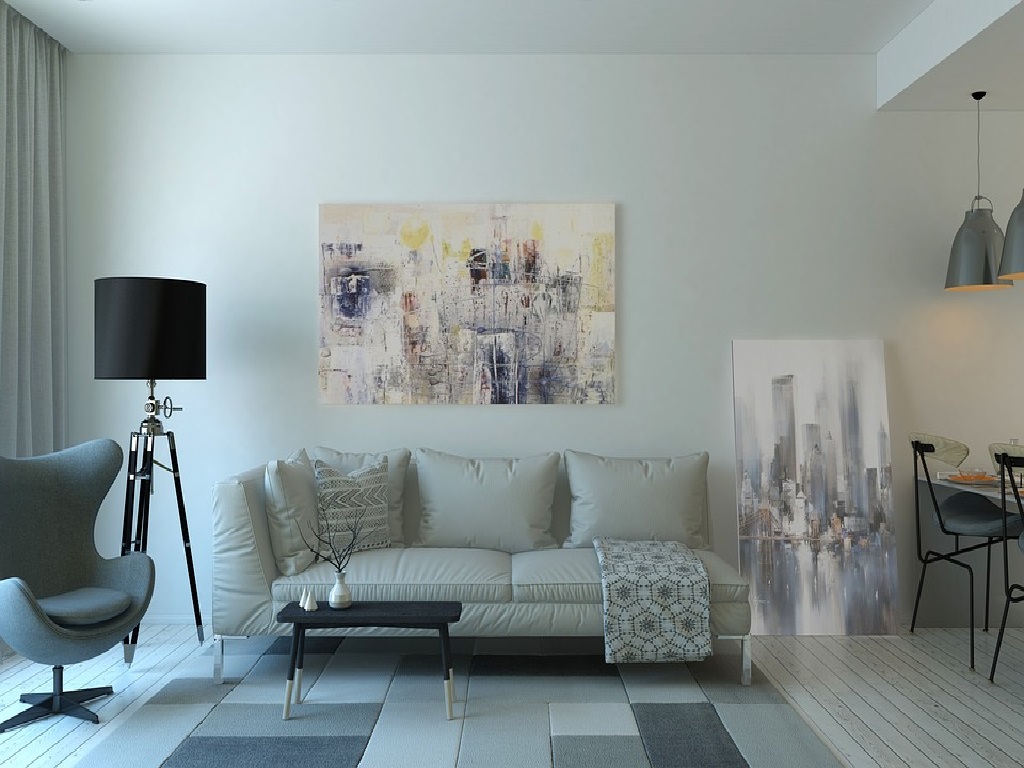

Supplement: Supplementary file 2 [file Data_Sheet_1.zip › Raw Images for Experiment 2/Indoors/indoor27.jpg]

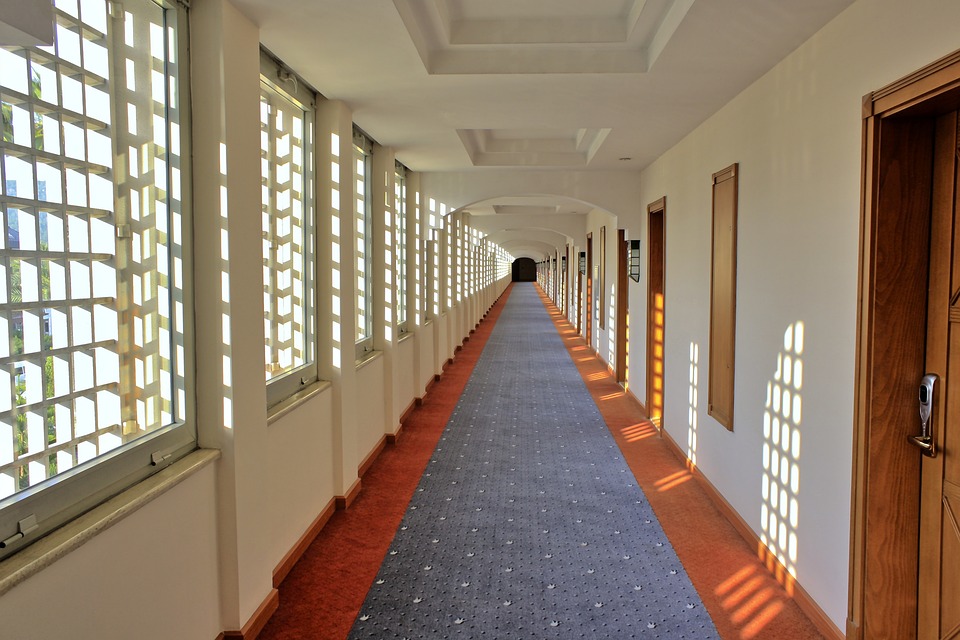

Supplement: Supplementary file 2 [file Data_Sheet_1.zip › Raw Images for Experiment 2/Indoors/indoor28.jpg]

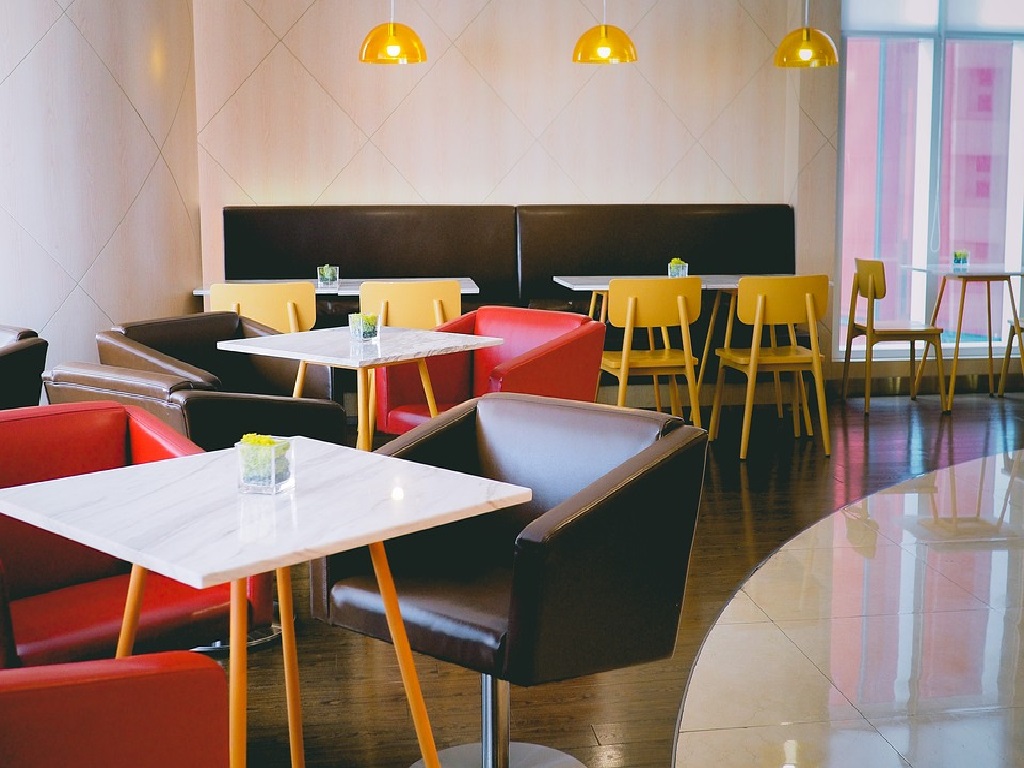

Supplement: Supplementary file 2 [file Data_Sheet_1.zip › Raw Images for Experiment 2/Indoors/indoor29.jpg]

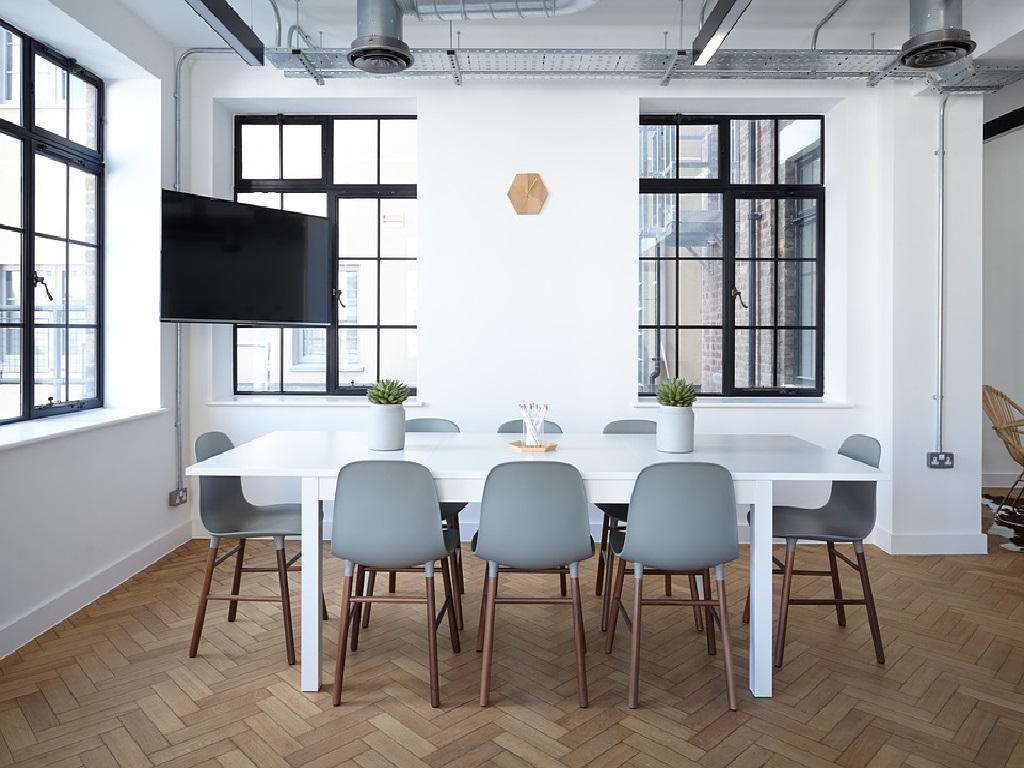

Supplement: Supplementary file 2 [file Data_Sheet_1.zip › Raw Images for Experiment 2/Indoors/indoor31.jpg]

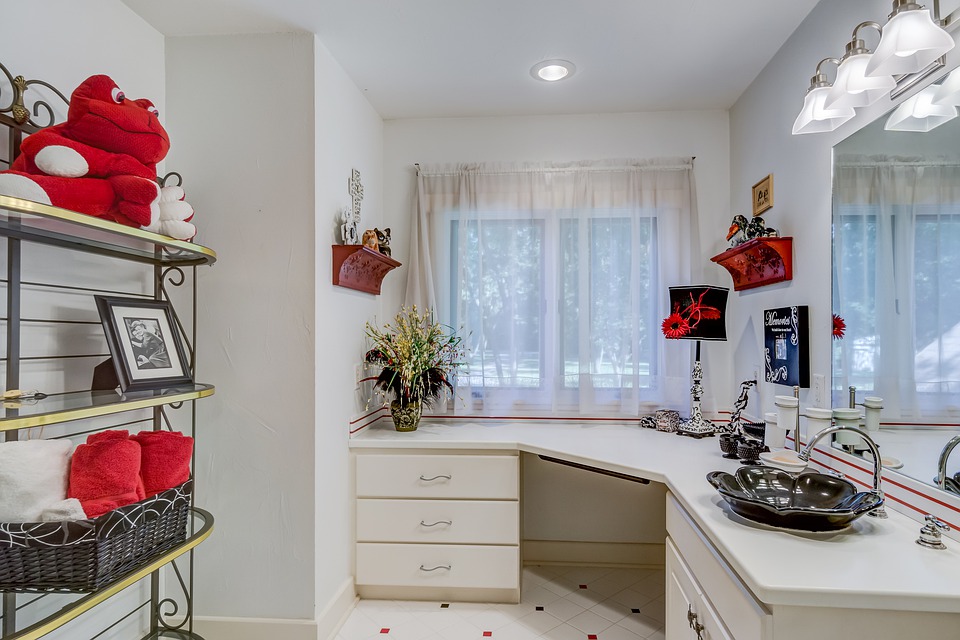

Supplement: Supplementary file 2 [file Data_Sheet_1.zip › Raw Images for Experiment 2/Indoors/indoor32.jpg]

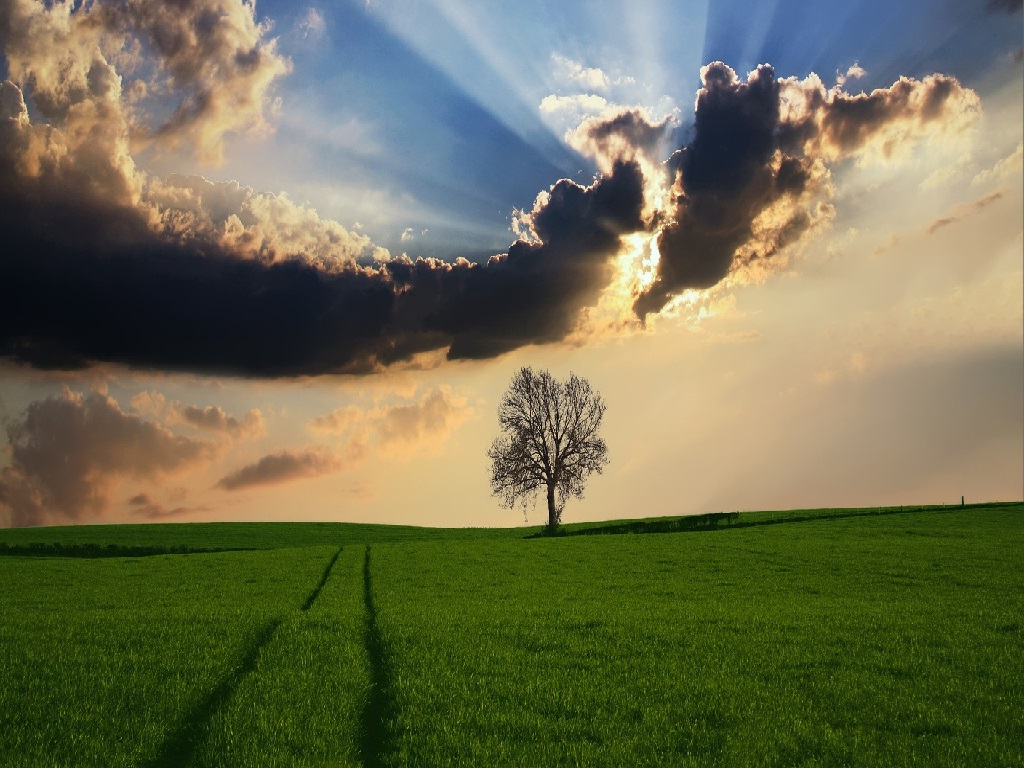

Supplement: Supplementary file 2 [file Data_Sheet_1.zip › Raw Images for Experiment 2/Land/land01.jpg]

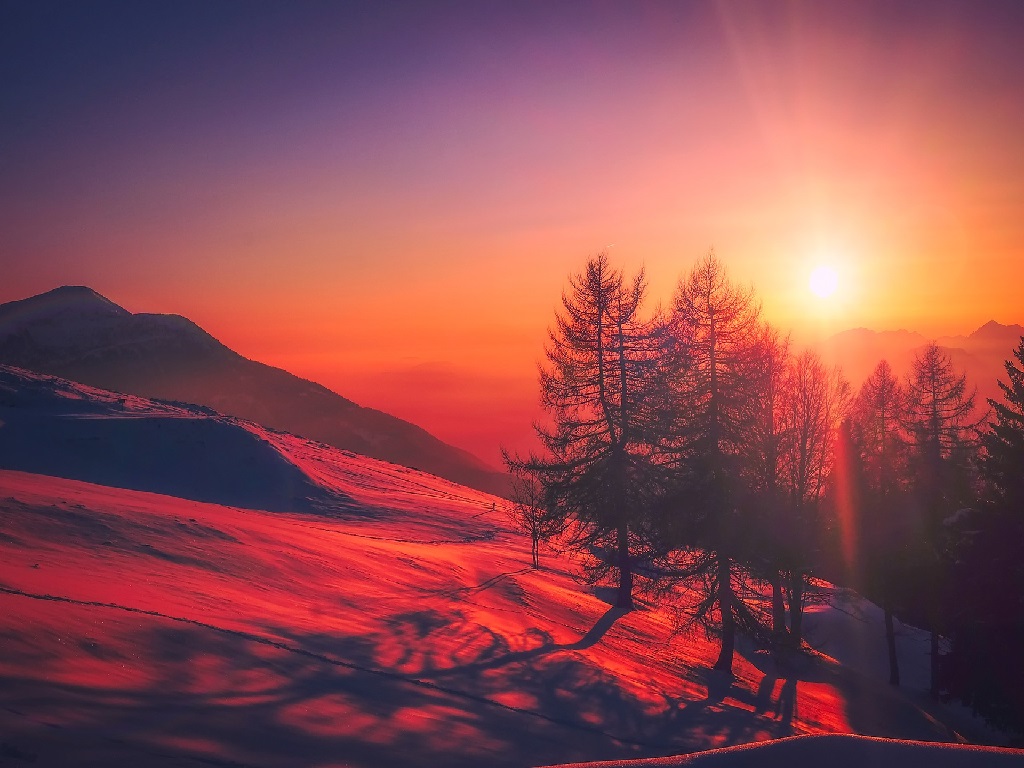

Supplement: Supplementary file 2 [file Data_Sheet_1.zip › Raw Images for Experiment 2/Land/land02.jpg]
