# Supplementary figures and images for: What Makes an Image Interesting and How Can We Explain It (part 1 of 3)
Source: Front Psychol. 2021 Sep 1;12:668651. doi: 10.3389/fpsyg.2021.668651 (PMC8440840; doi:10.3389/fpsyg.2021.668651)

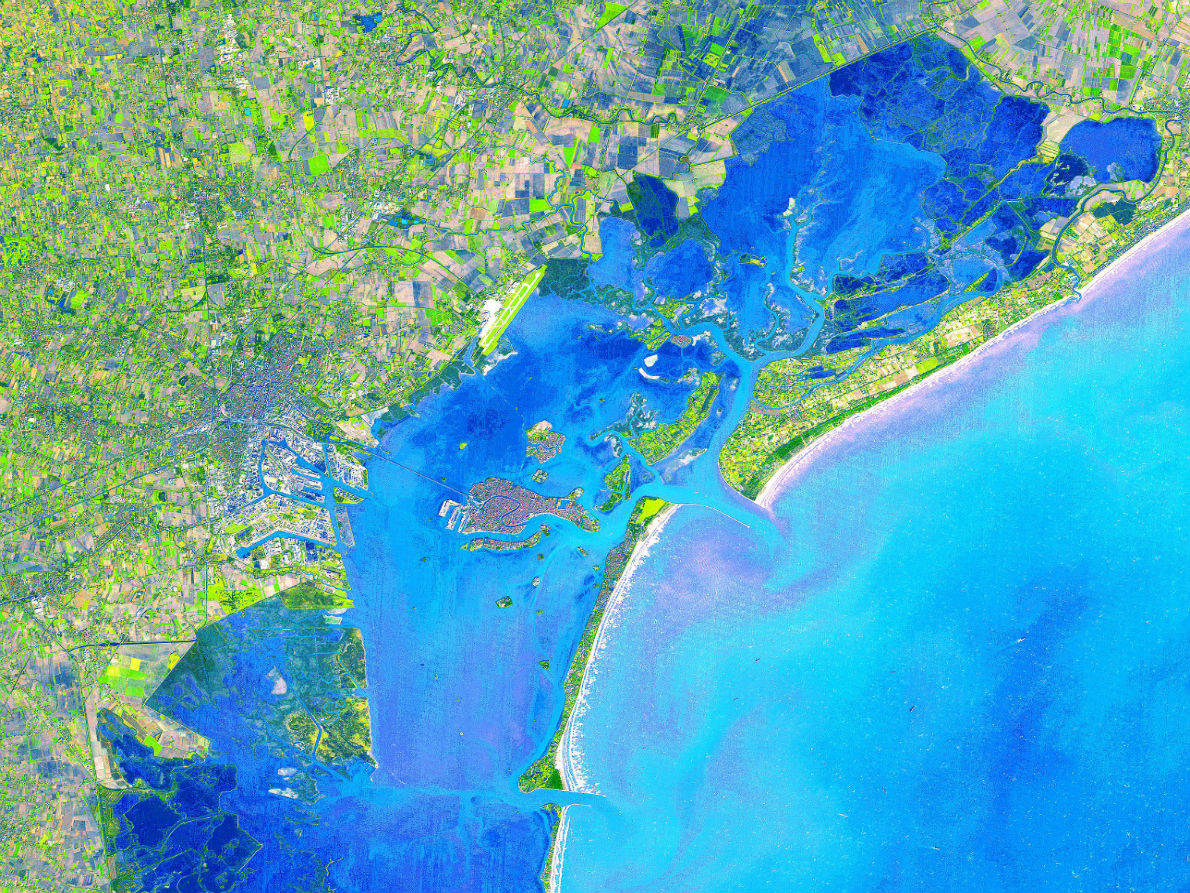

Supplement: Supplementary file 2 [file Data_Sheet_1.zip › Raw Images for Experiment 1/Aerials/aer1.jpg]

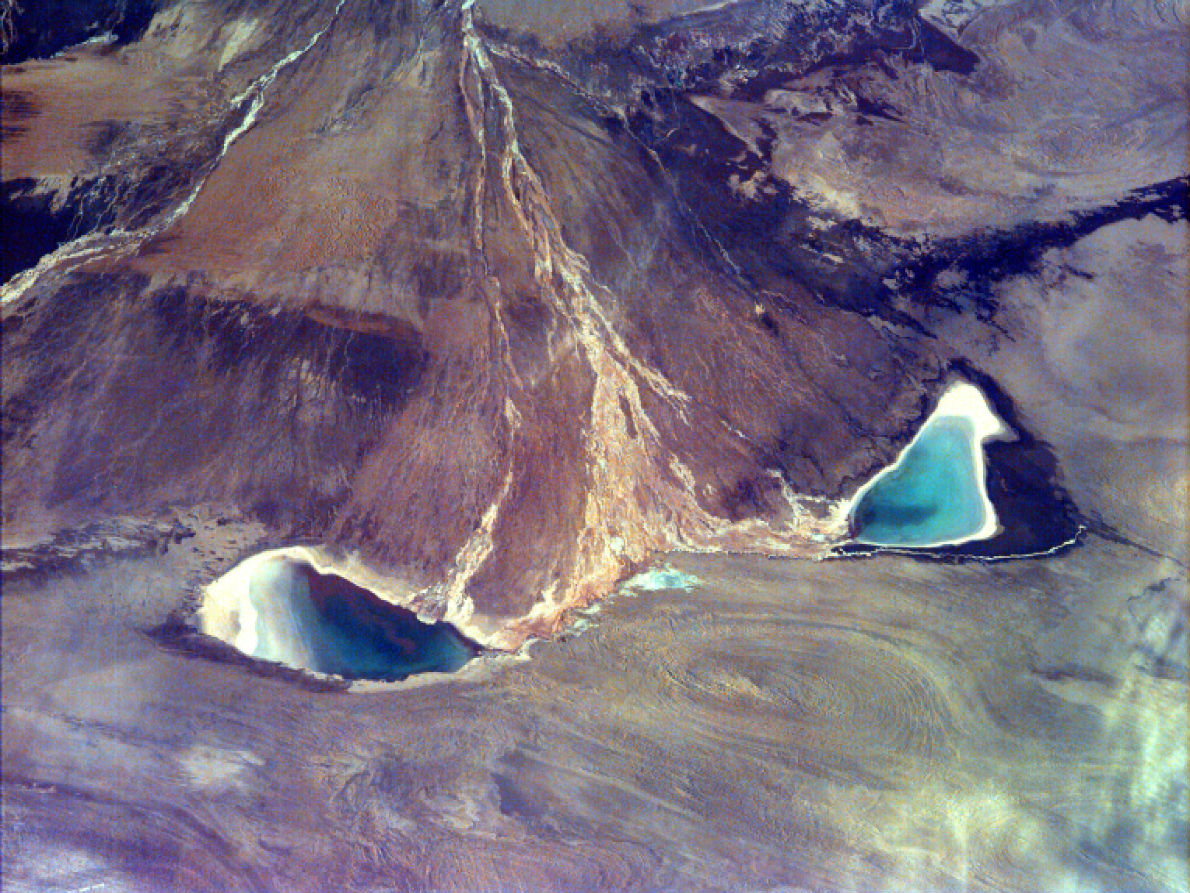

Supplement: Supplementary file 2 [file Data_Sheet_1.zip › Raw Images for Experiment 1/Aerials/aer10.jpg]

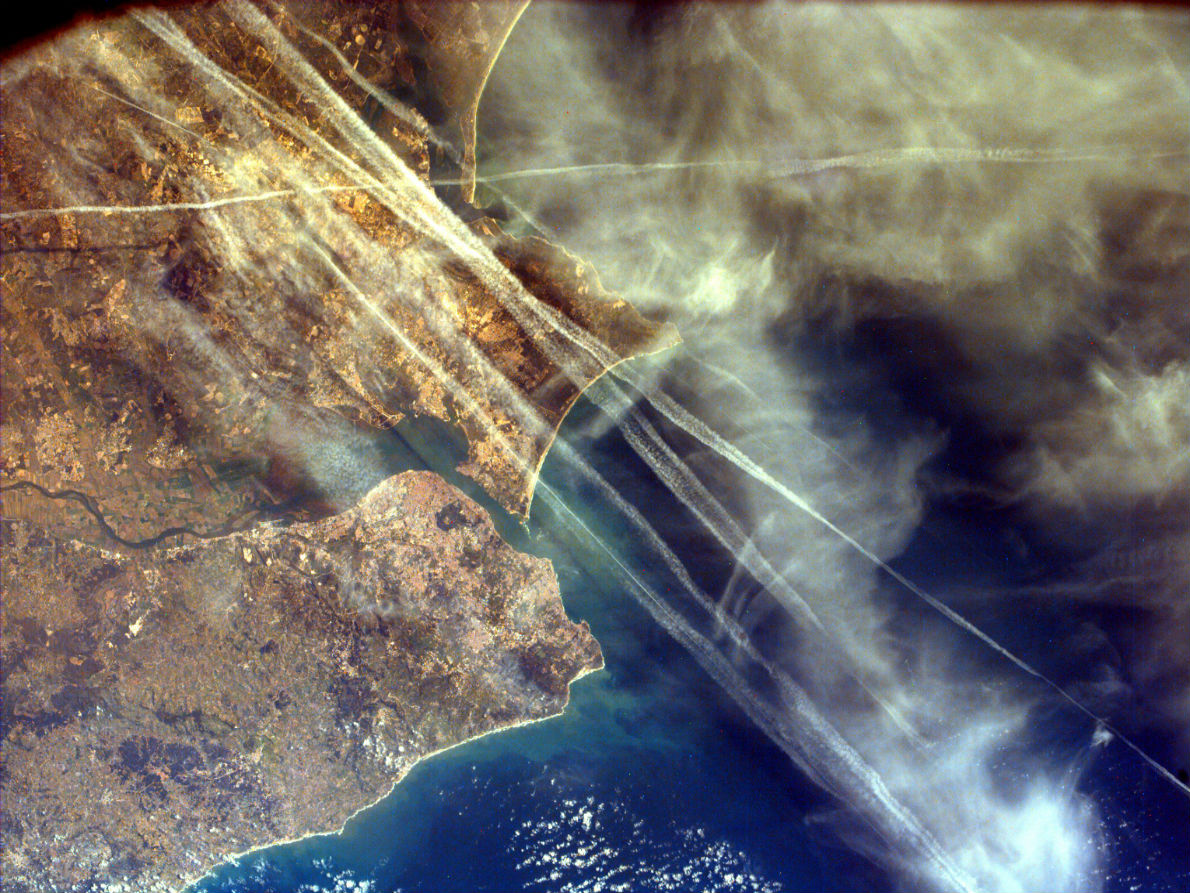

Supplement: Supplementary file 2 [file Data_Sheet_1.zip › Raw Images for Experiment 1/Aerials/aer11.jpg]

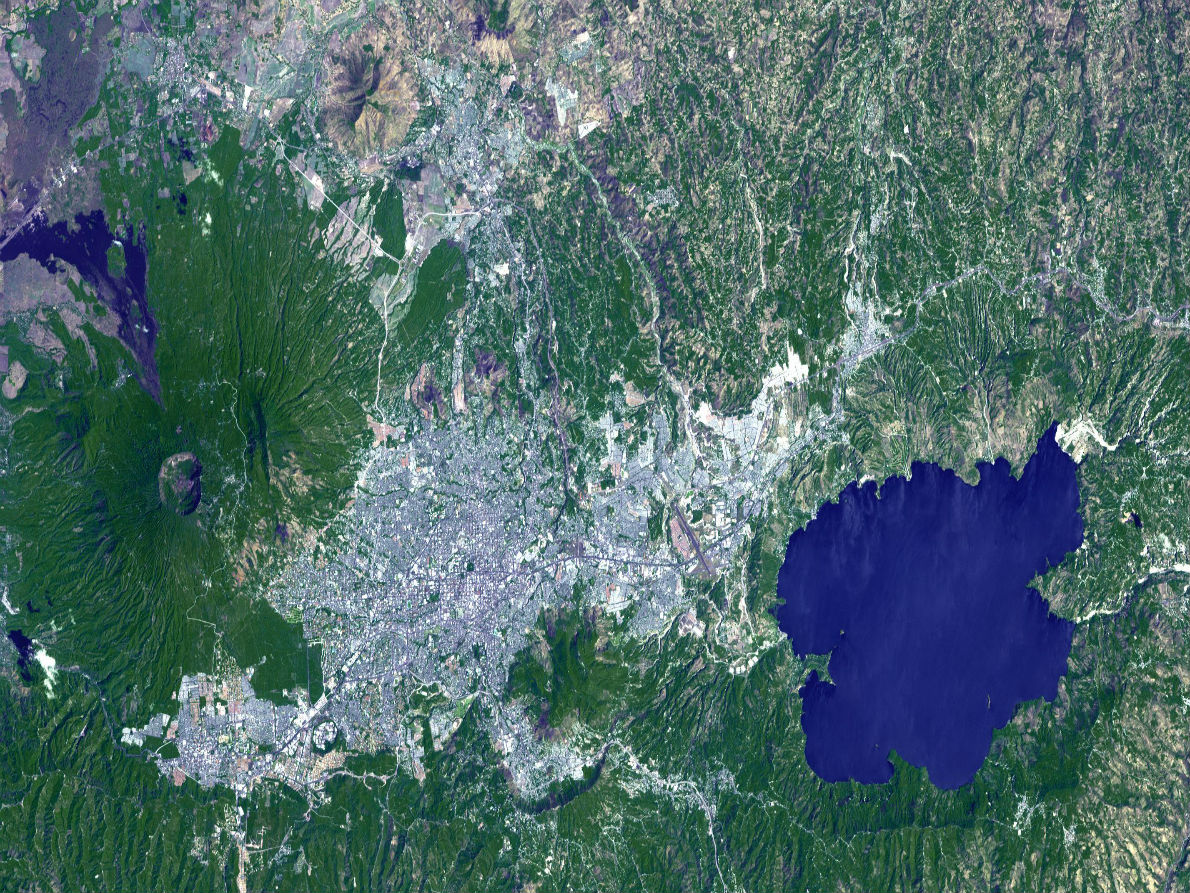

Supplement: Supplementary file 2 [file Data_Sheet_1.zip › Raw Images for Experiment 1/Aerials/aer12.jpg]

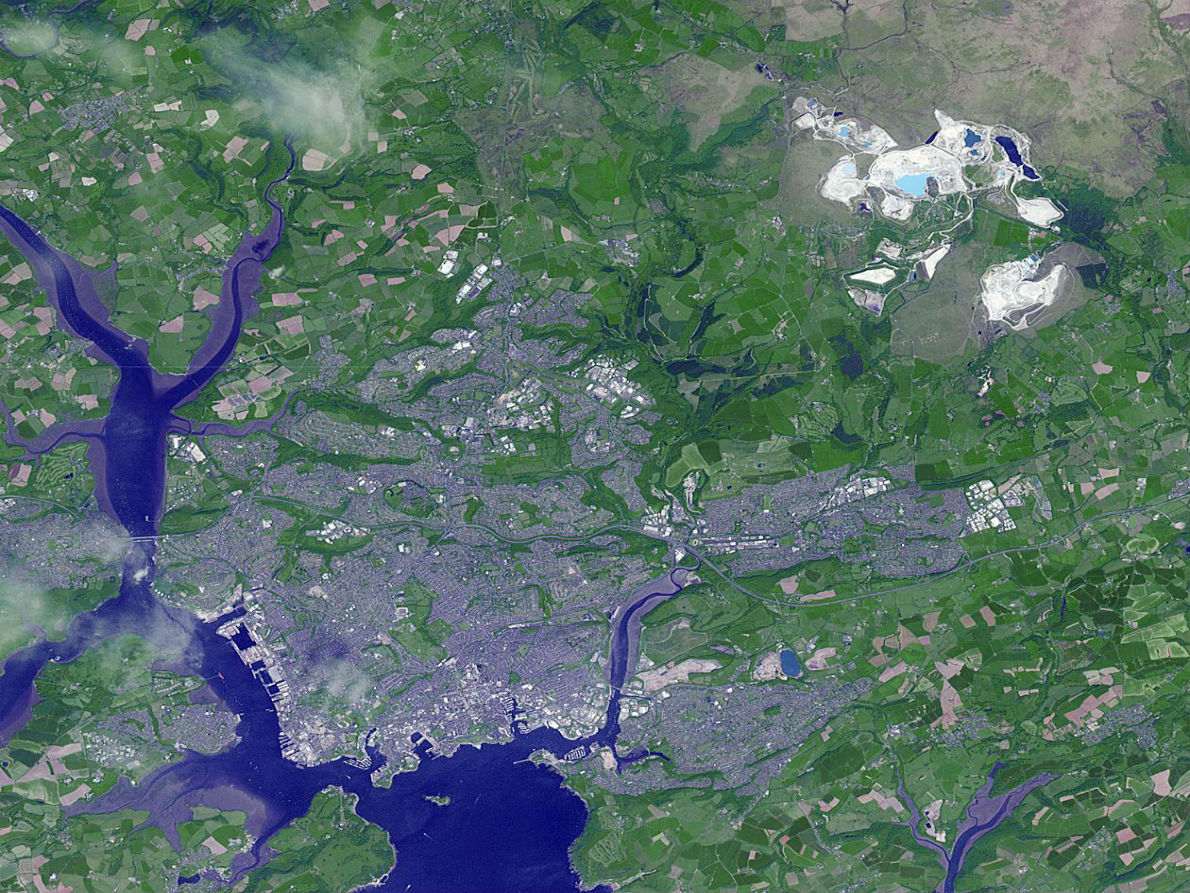

Supplement: Supplementary file 2 [file Data_Sheet_1.zip › Raw Images for Experiment 1/Aerials/aer13.jpg]

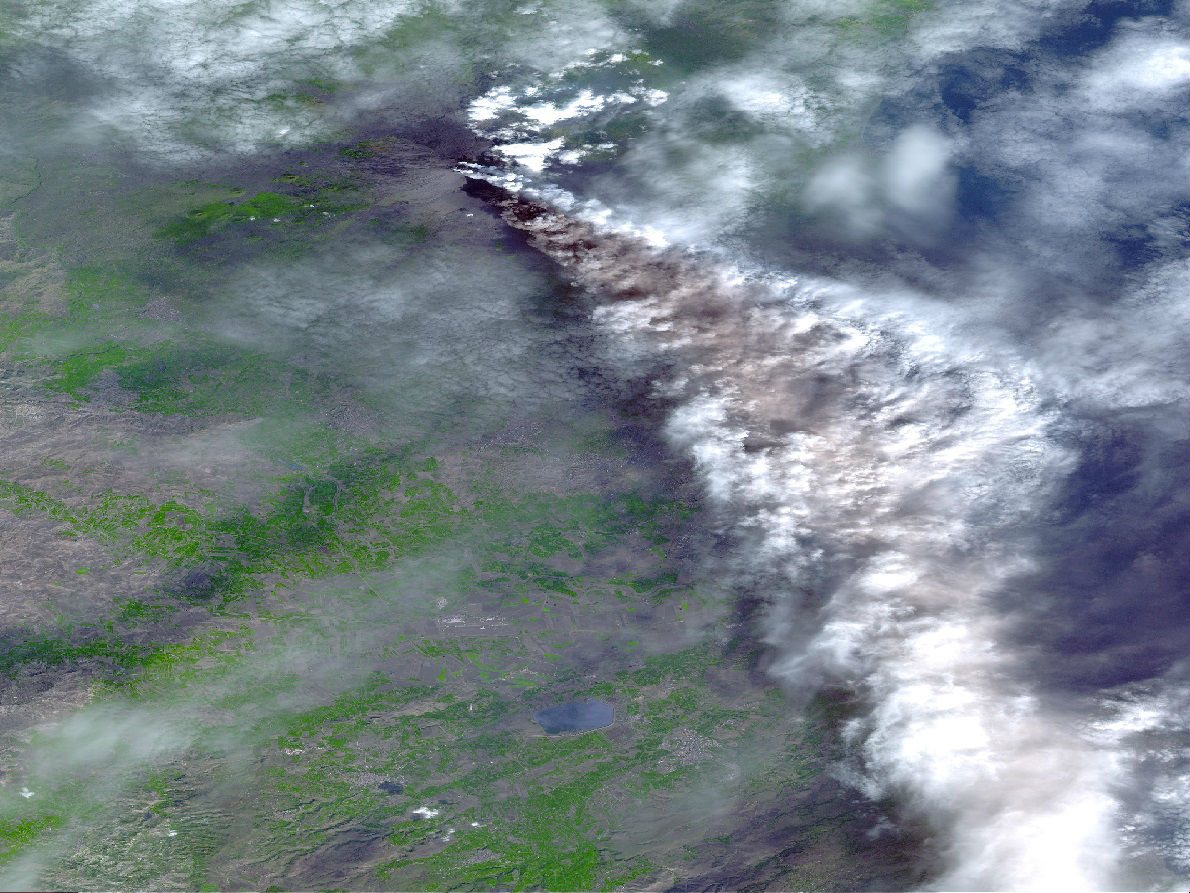

Supplement: Supplementary file 2 [file Data_Sheet_1.zip › Raw Images for Experiment 1/Aerials/aer14.jpg]

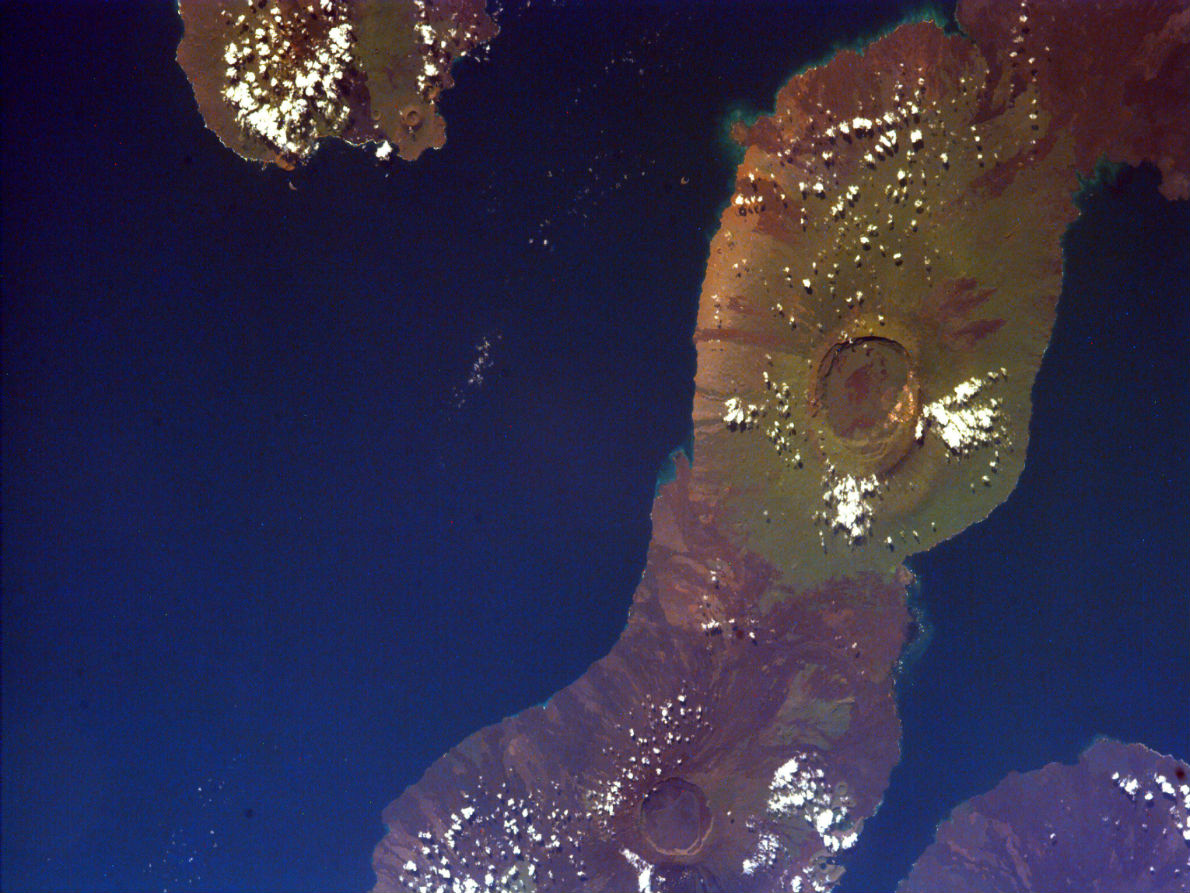

Supplement: Supplementary file 2 [file Data_Sheet_1.zip › Raw Images for Experiment 1/Aerials/aer15.jpg]

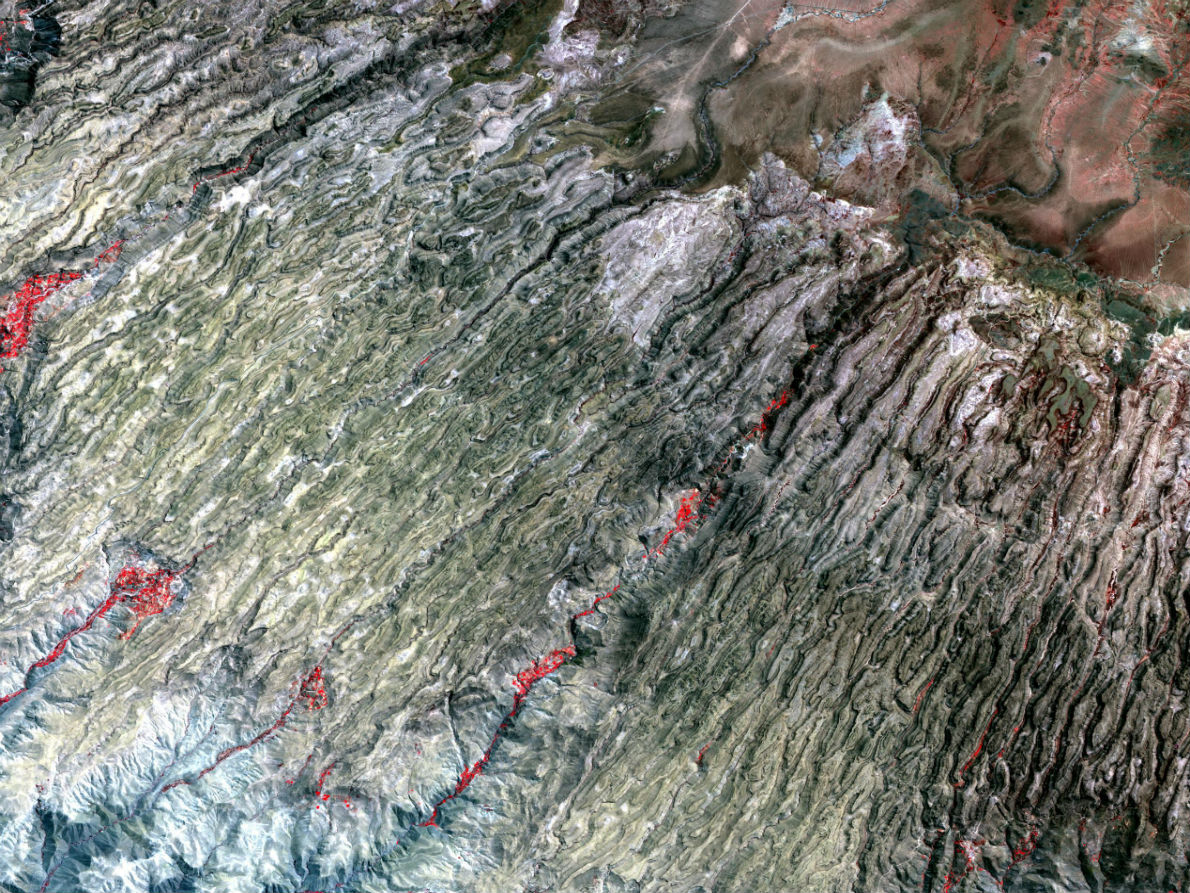

Supplement: Supplementary file 2 [file Data_Sheet_1.zip › Raw Images for Experiment 1/Aerials/aer16.jpg]

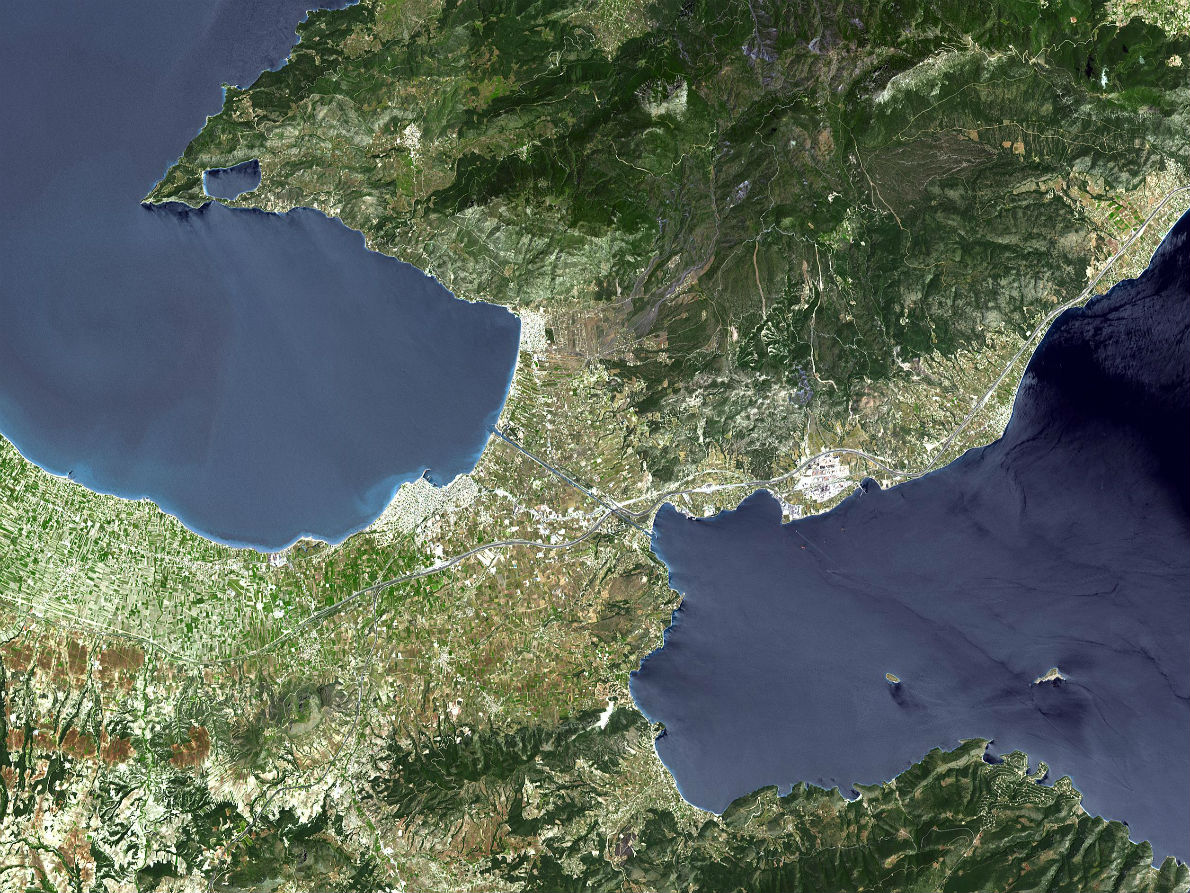

Supplement: Supplementary file 2 [file Data_Sheet_1.zip › Raw Images for Experiment 1/Aerials/aer17.jpg]

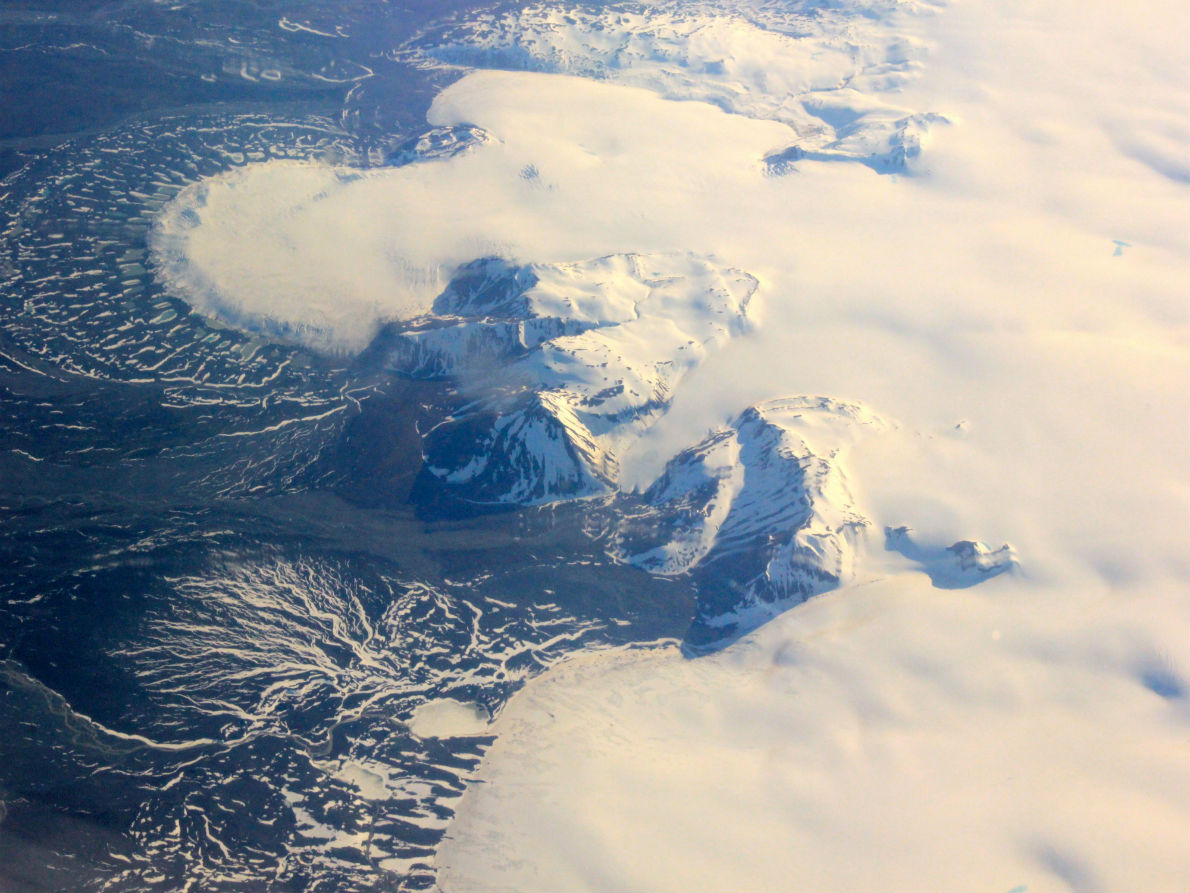

Supplement: Supplementary file 2 [file Data_Sheet_1.zip › Raw Images for Experiment 1/Aerials/aer18.jpg]

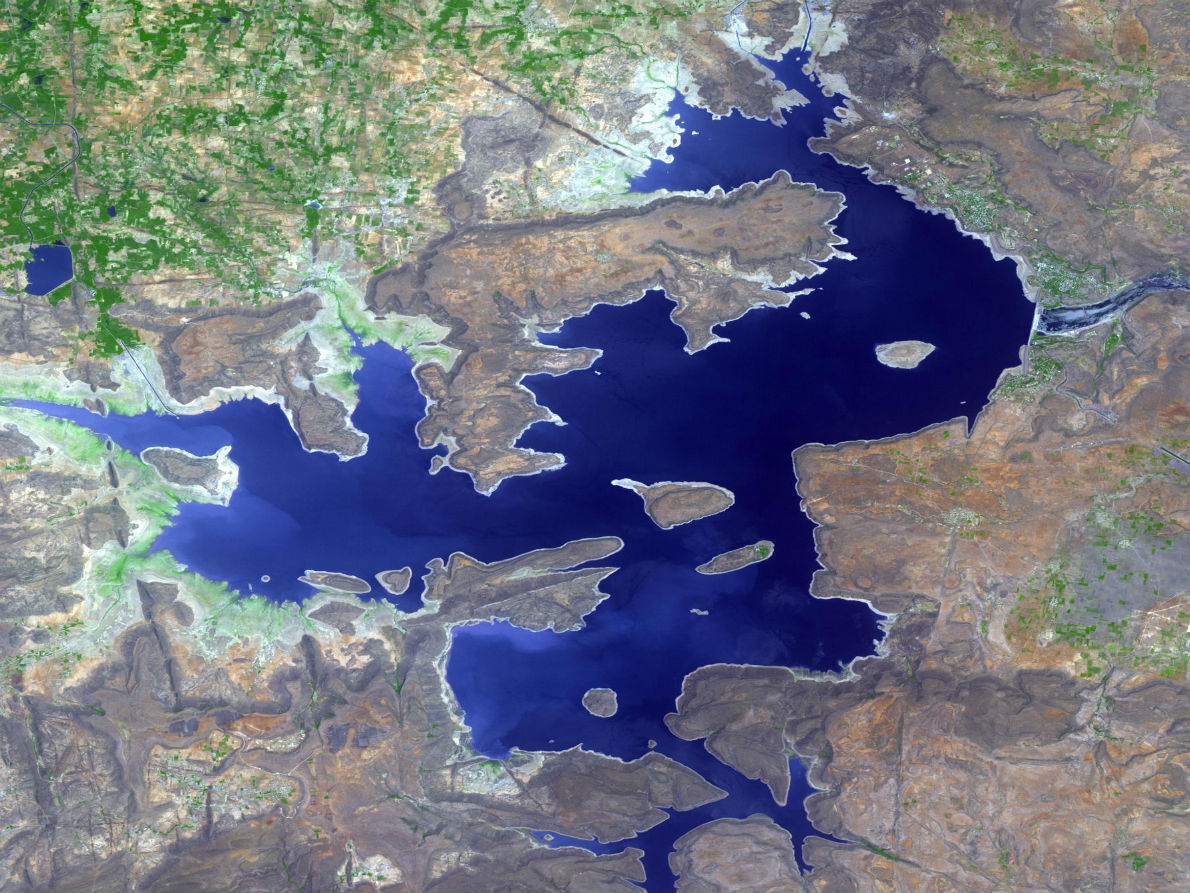

Supplement: Supplementary file 2 [file Data_Sheet_1.zip › Raw Images for Experiment 1/Aerials/aer19.jpg]

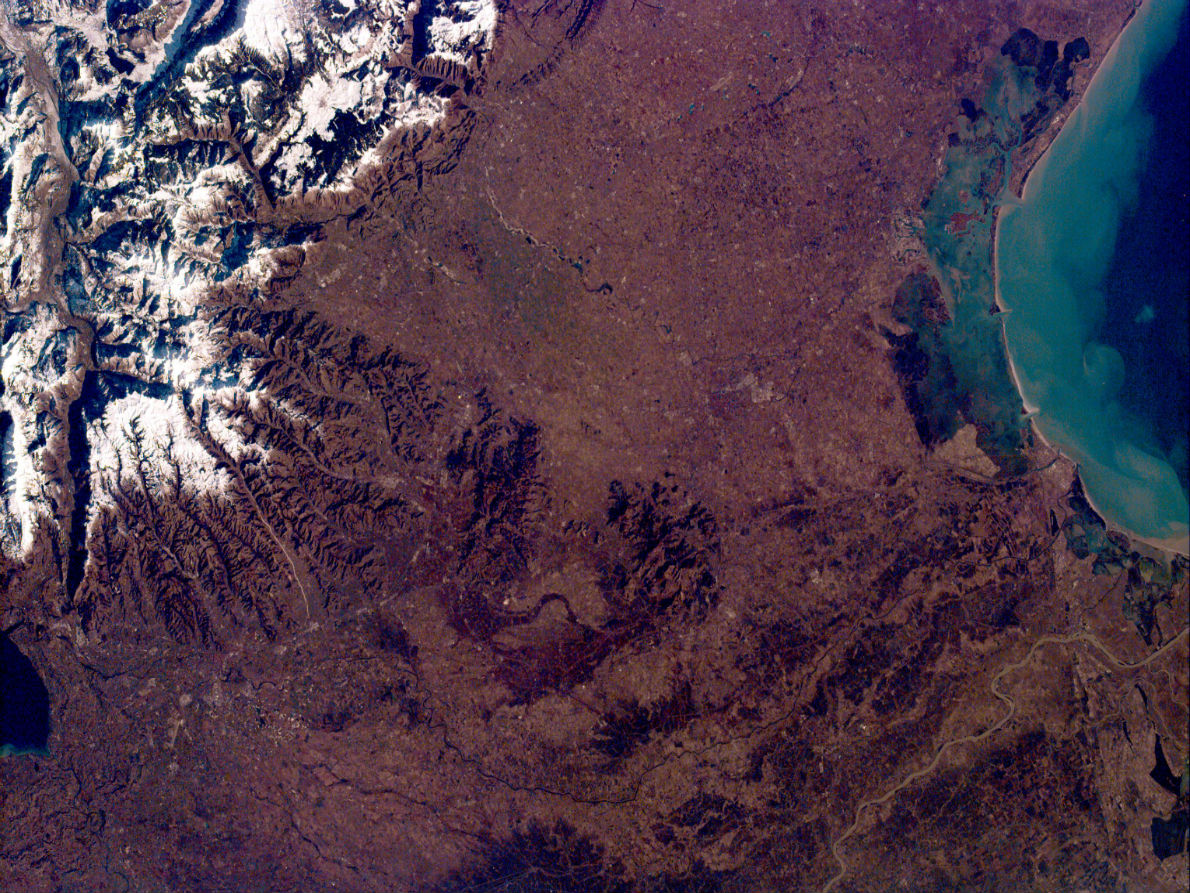

Supplement: Supplementary file 2 [file Data_Sheet_1.zip › Raw Images for Experiment 1/Aerials/aer2.jpg]

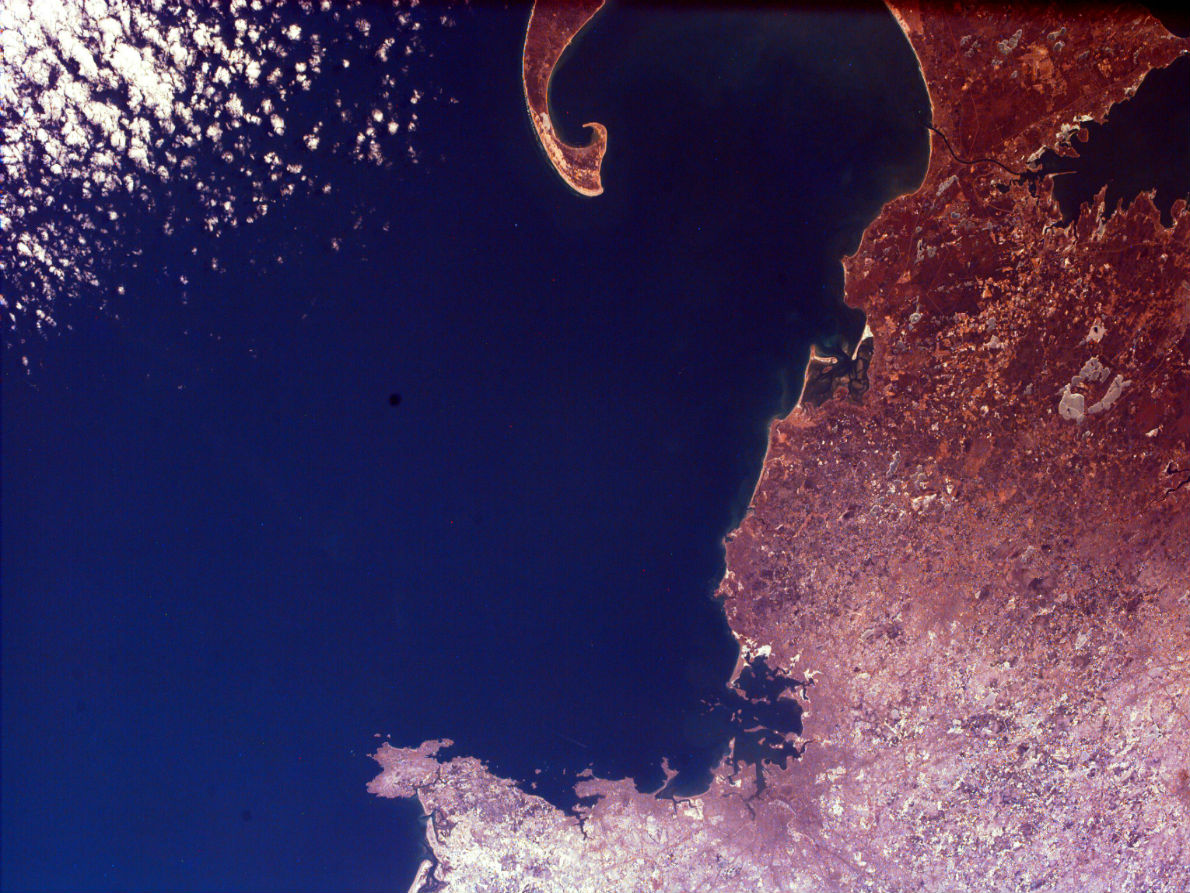

Supplement: Supplementary file 2 [file Data_Sheet_1.zip › Raw Images for Experiment 1/Aerials/aer20.jpg]

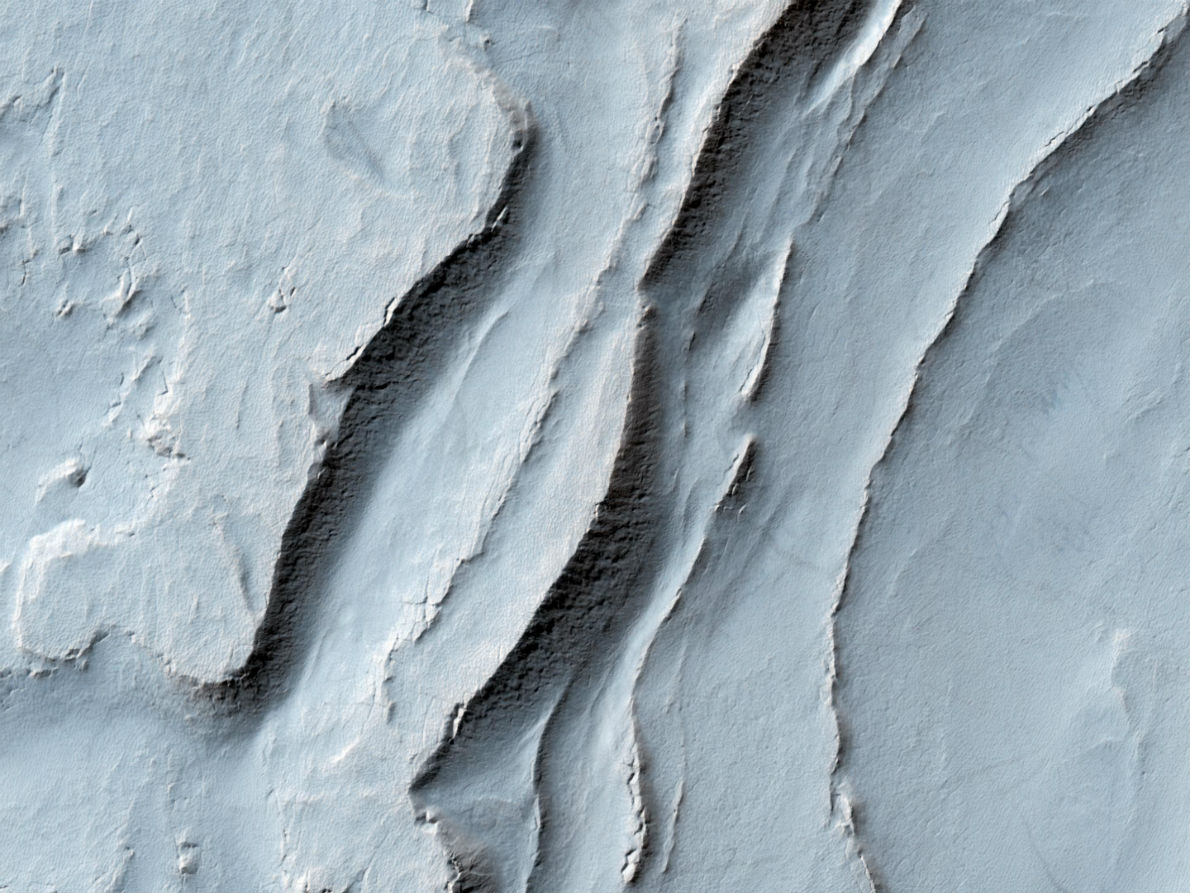

Supplement: Supplementary file 2 [file Data_Sheet_1.zip › Raw Images for Experiment 1/Aerials/aer21.jpg]

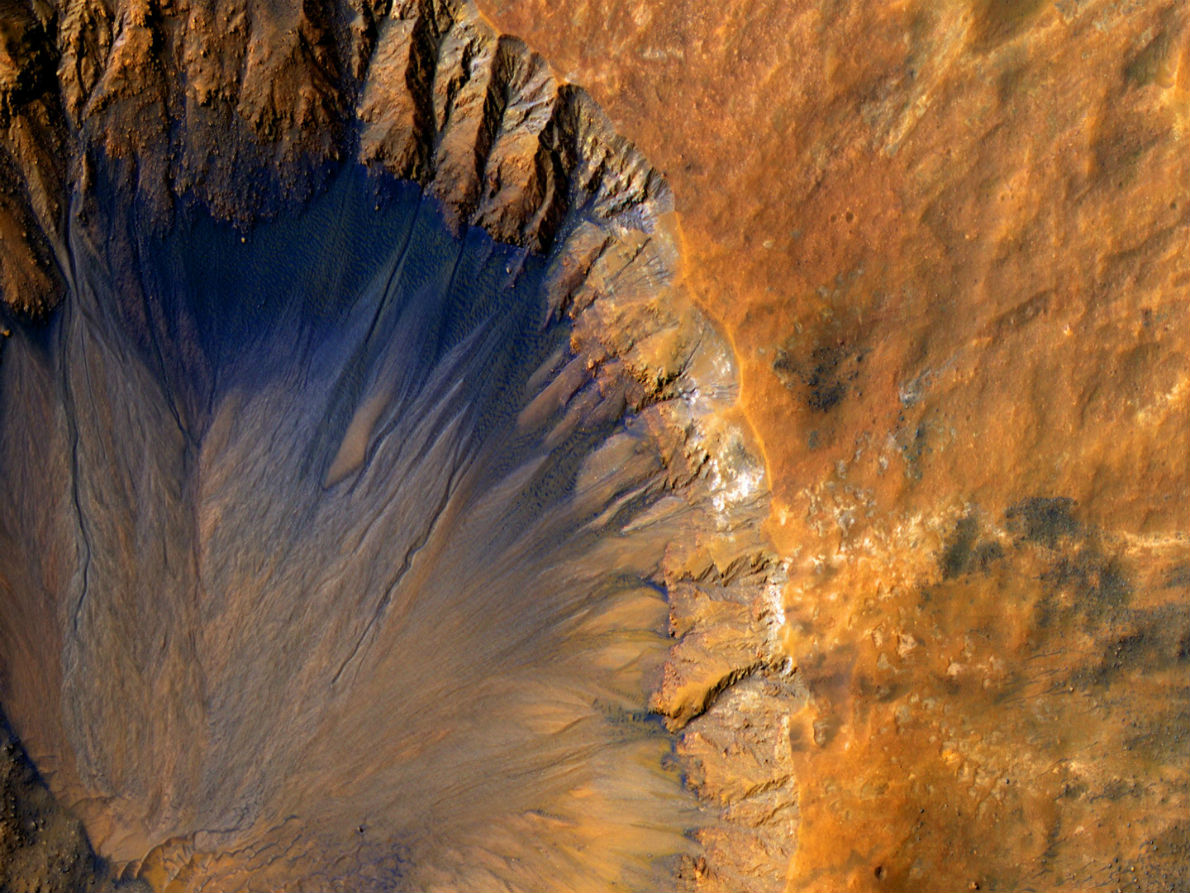

Supplement: Supplementary file 2 [file Data_Sheet_1.zip › Raw Images for Experiment 1/Aerials/aer22.jpg]

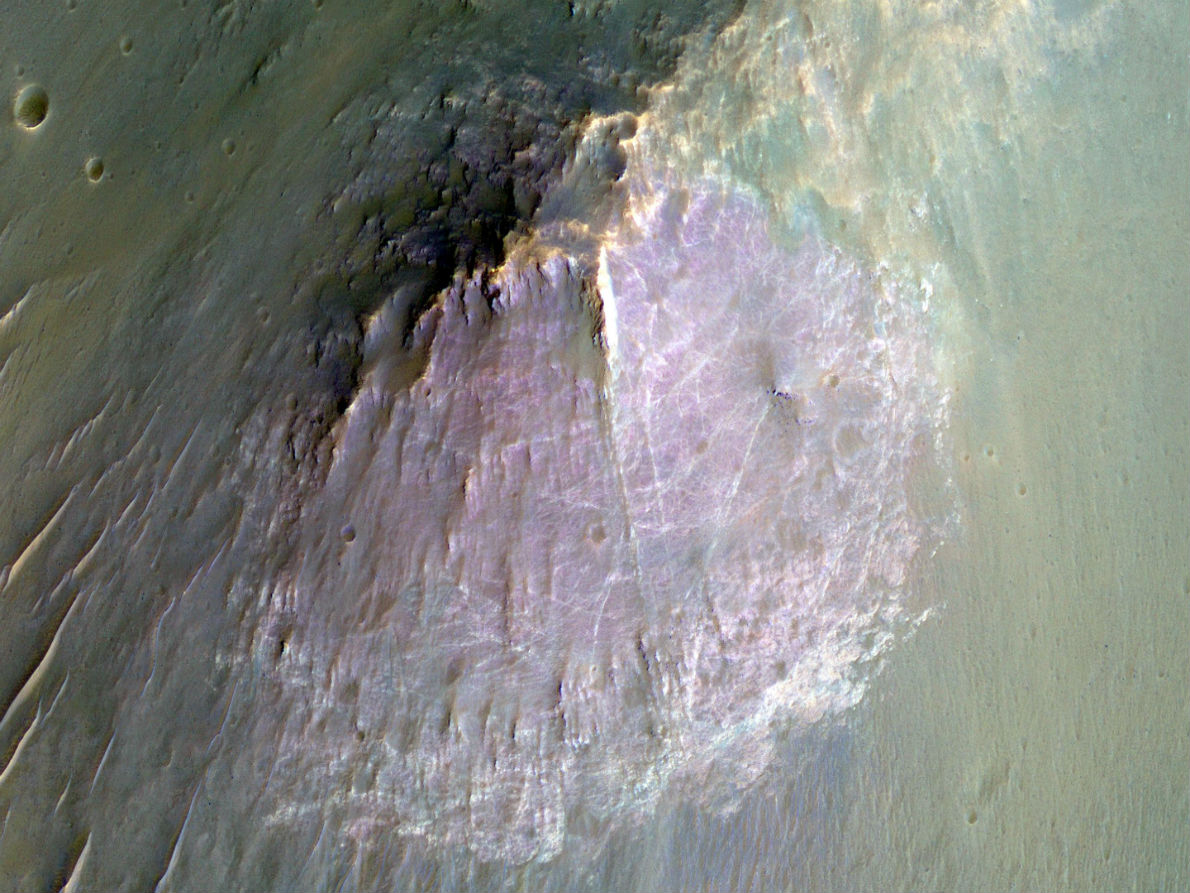

Supplement: Supplementary file 2 [file Data_Sheet_1.zip › Raw Images for Experiment 1/Aerials/aer23.jpg]

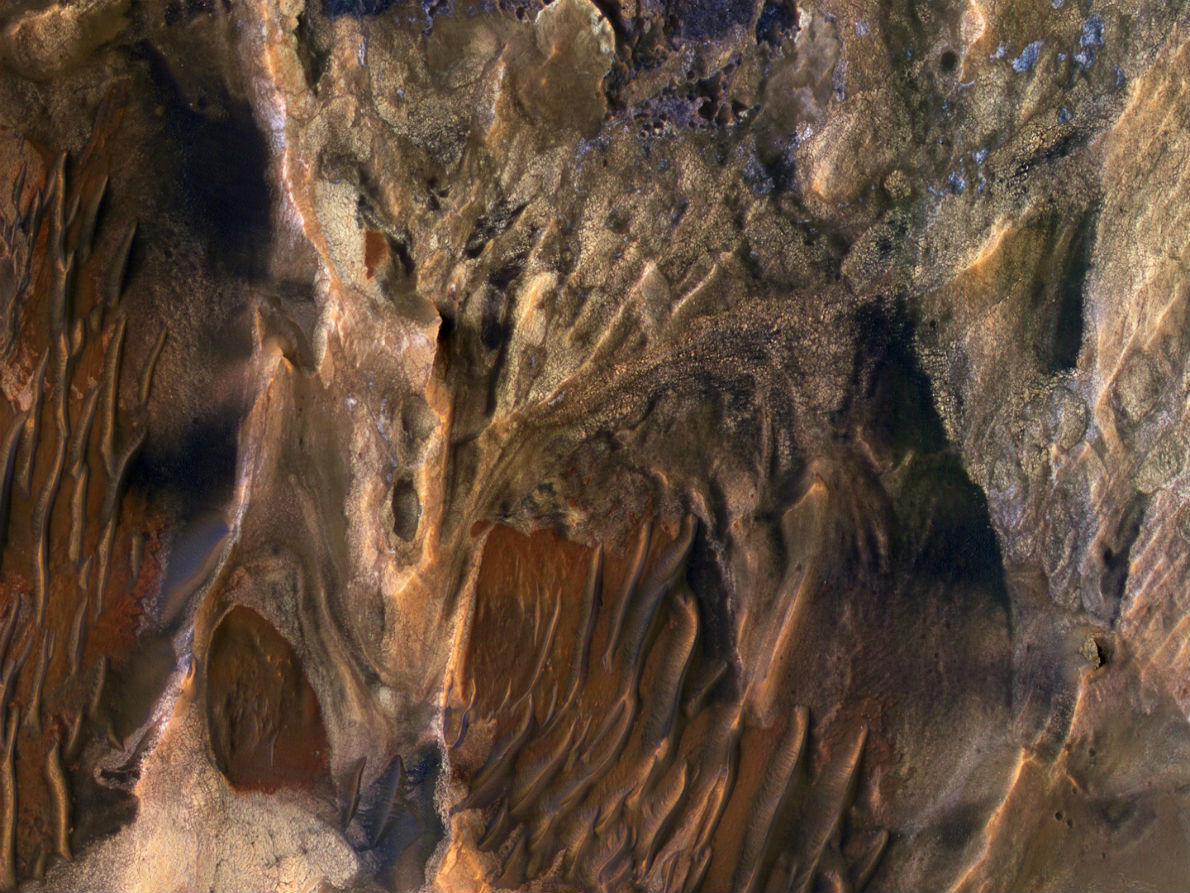

Supplement: Supplementary file 2 [file Data_Sheet_1.zip › Raw Images for Experiment 1/Aerials/aer24.jpg]

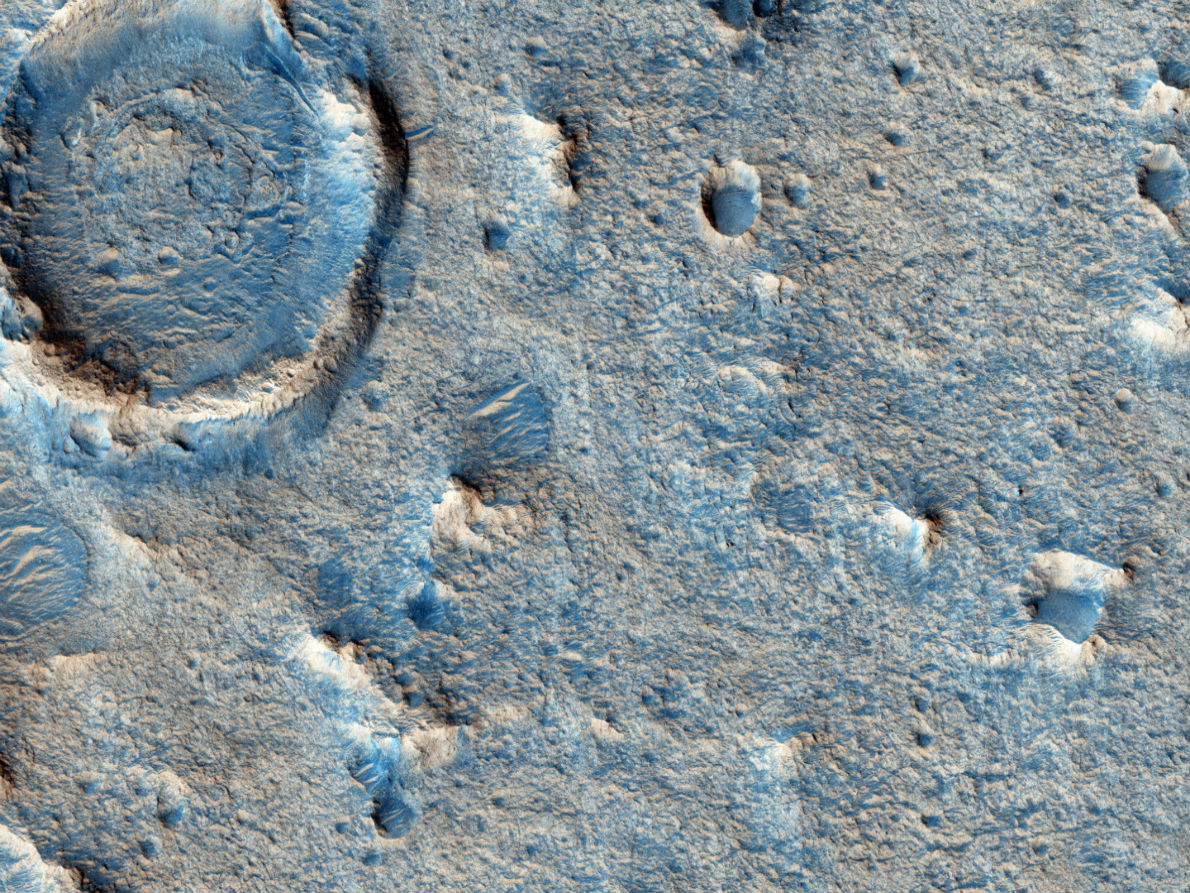

Supplement: Supplementary file 2 [file Data_Sheet_1.zip › Raw Images for Experiment 1/Aerials/aer25.jpg]

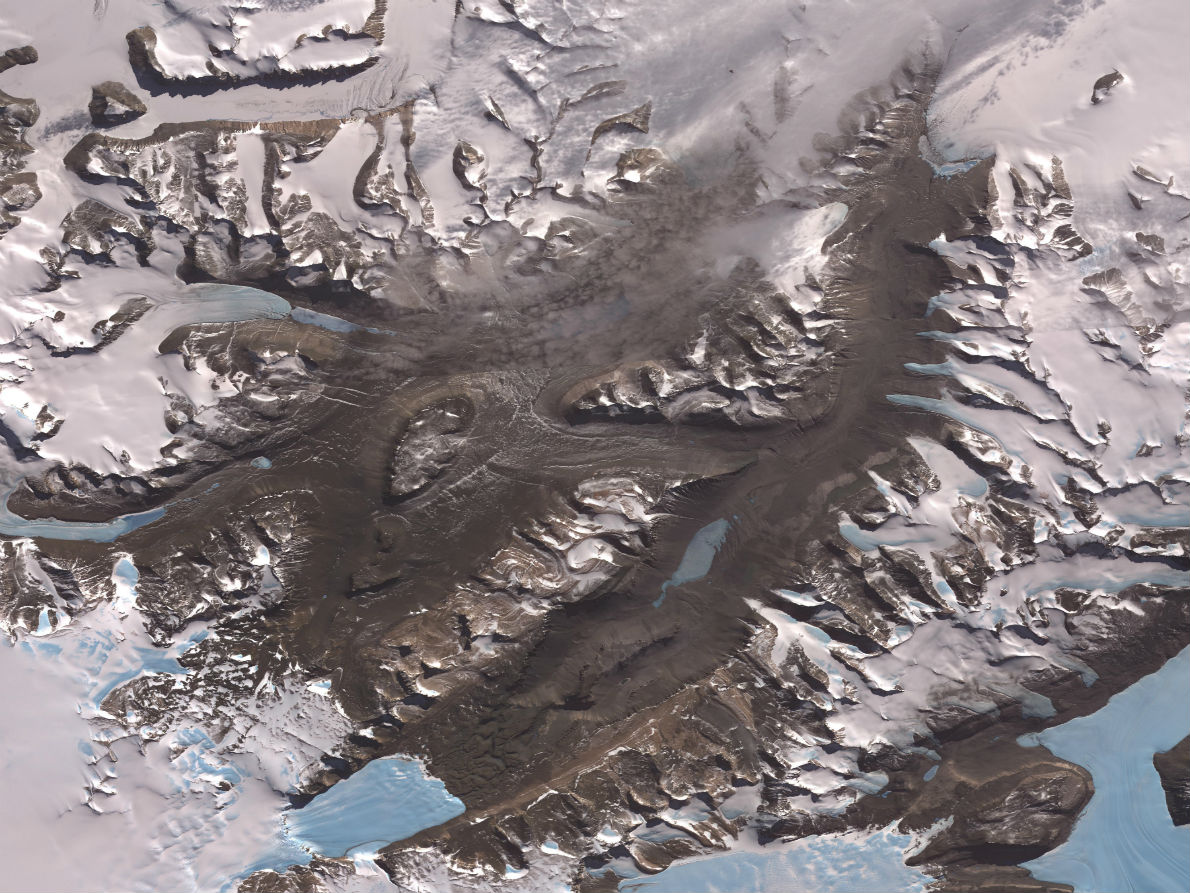

Supplement: Supplementary file 2 [file Data_Sheet_1.zip › Raw Images for Experiment 1/Aerials/aer3.jpg]

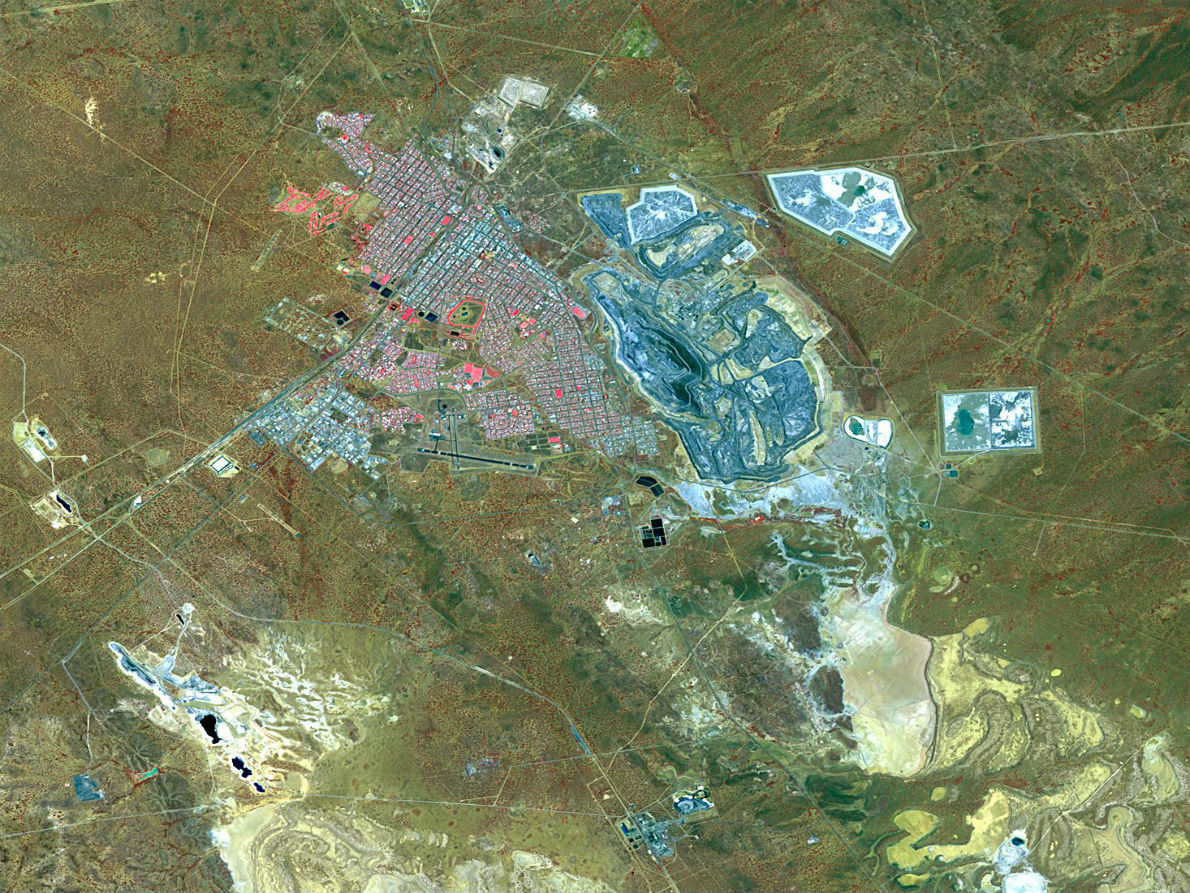

Supplement: Supplementary file 2 [file Data_Sheet_1.zip › Raw Images for Experiment 1/Aerials/aer4.jpg]

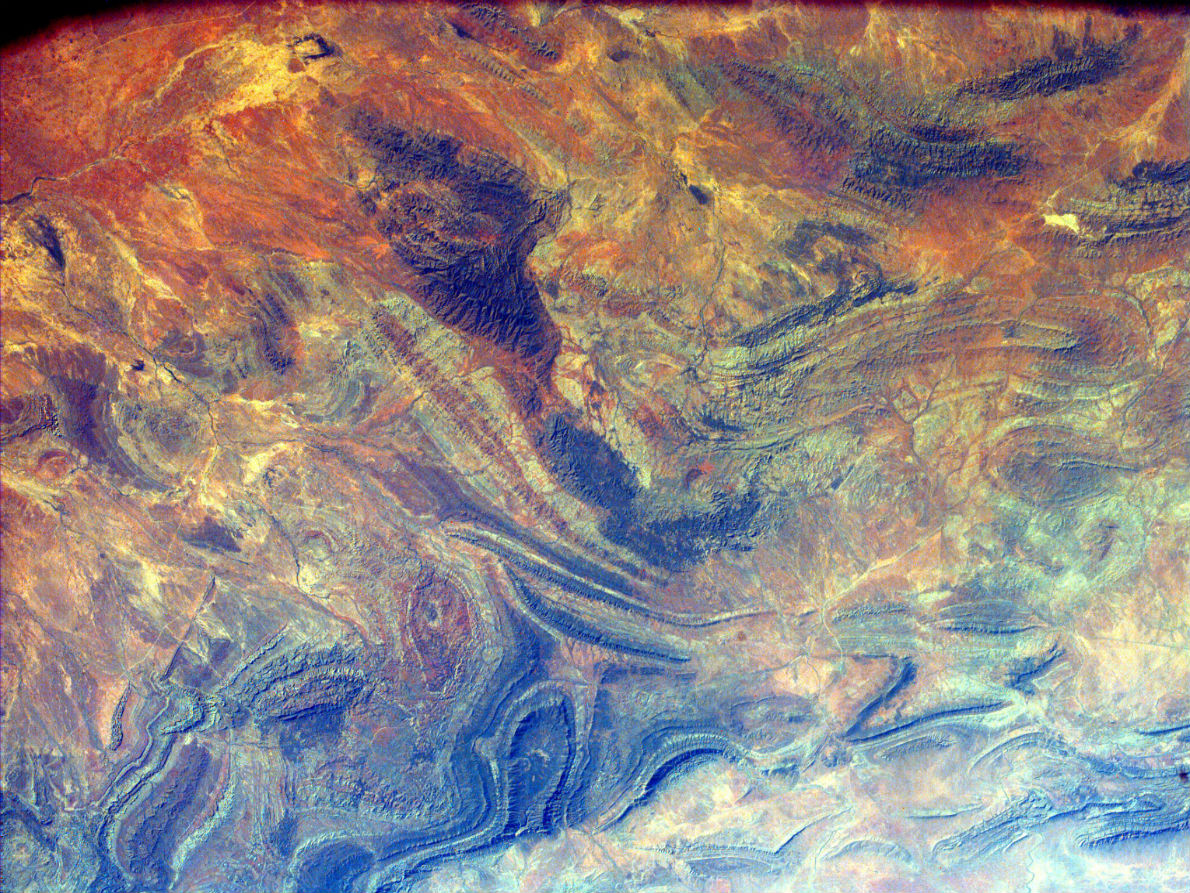

Supplement: Supplementary file 2 [file Data_Sheet_1.zip › Raw Images for Experiment 1/Aerials/aer5.jpg]

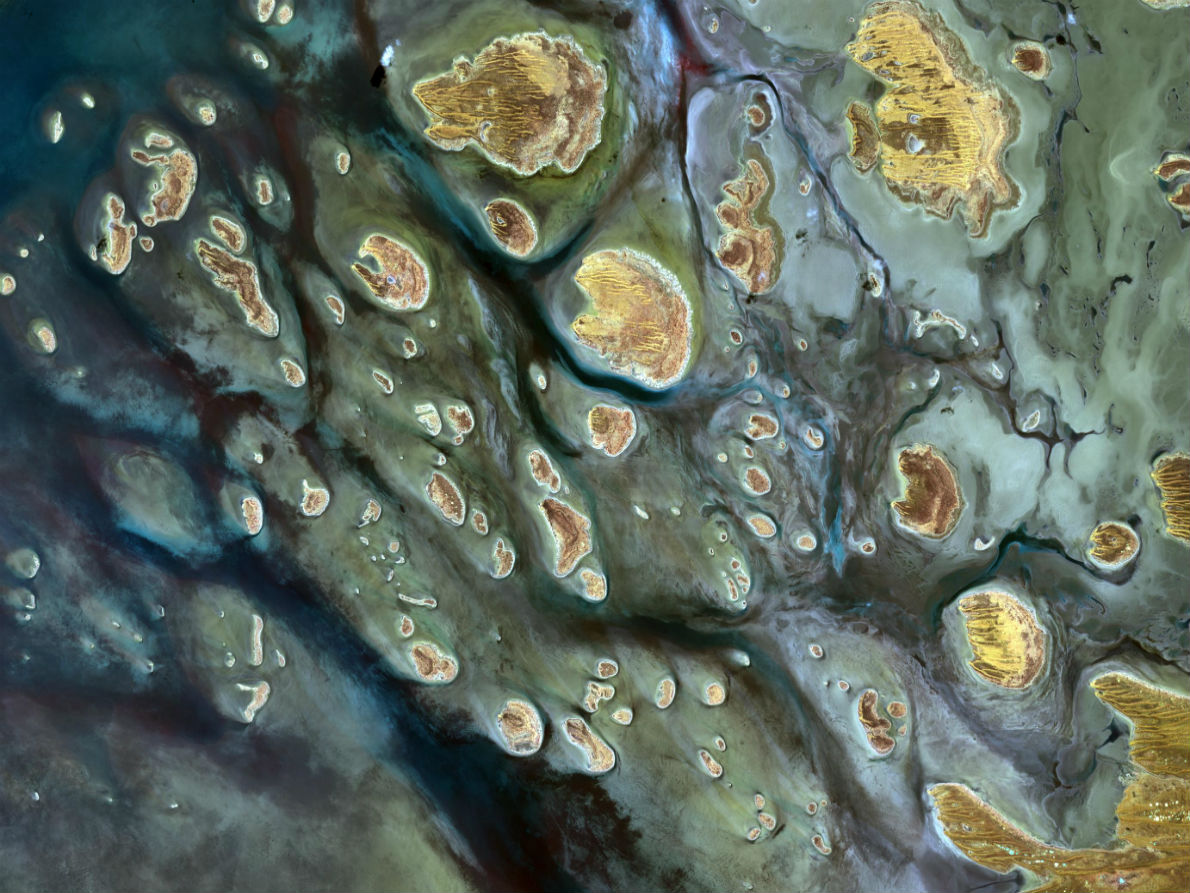

Supplement: Supplementary file 2 [file Data_Sheet_1.zip › Raw Images for Experiment 1/Aerials/aer6.jpg]

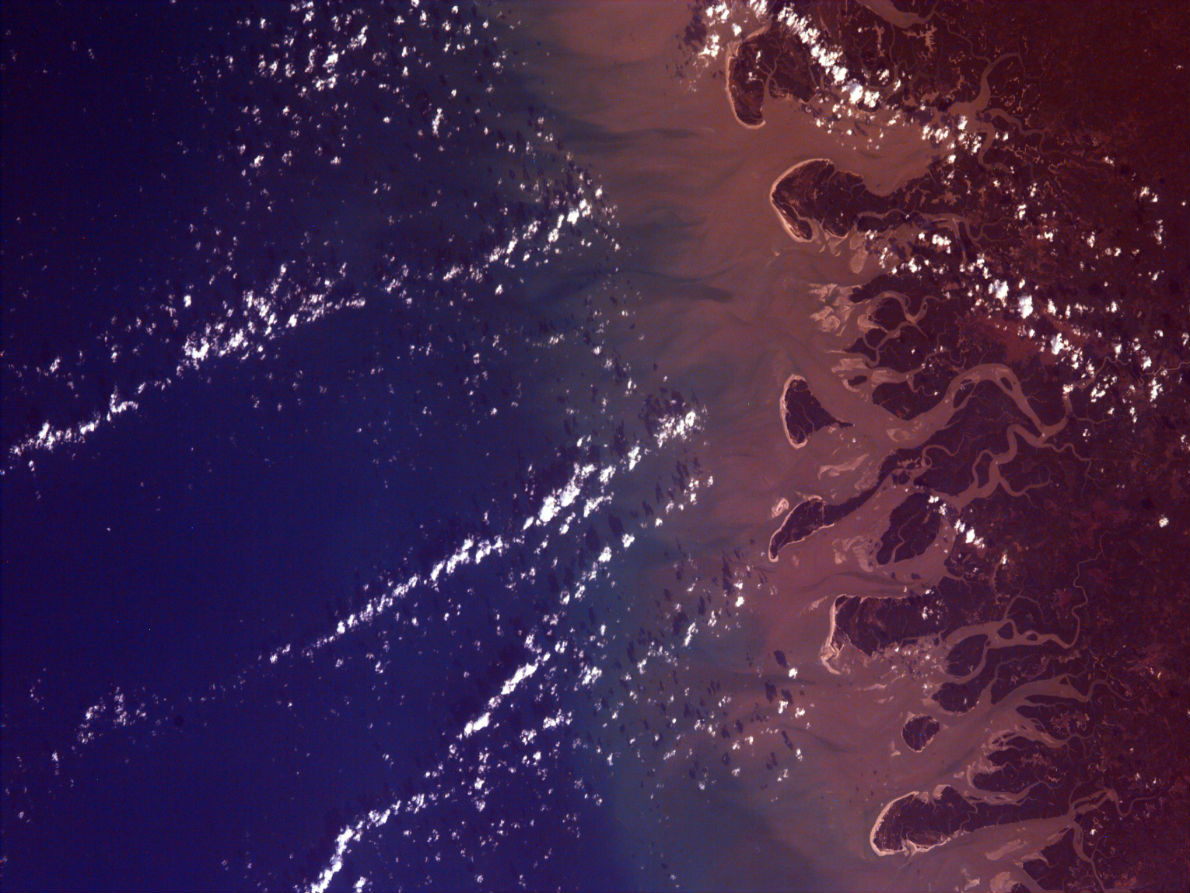

Supplement: Supplementary file 2 [file Data_Sheet_1.zip › Raw Images for Experiment 1/Aerials/aer7.jpg]

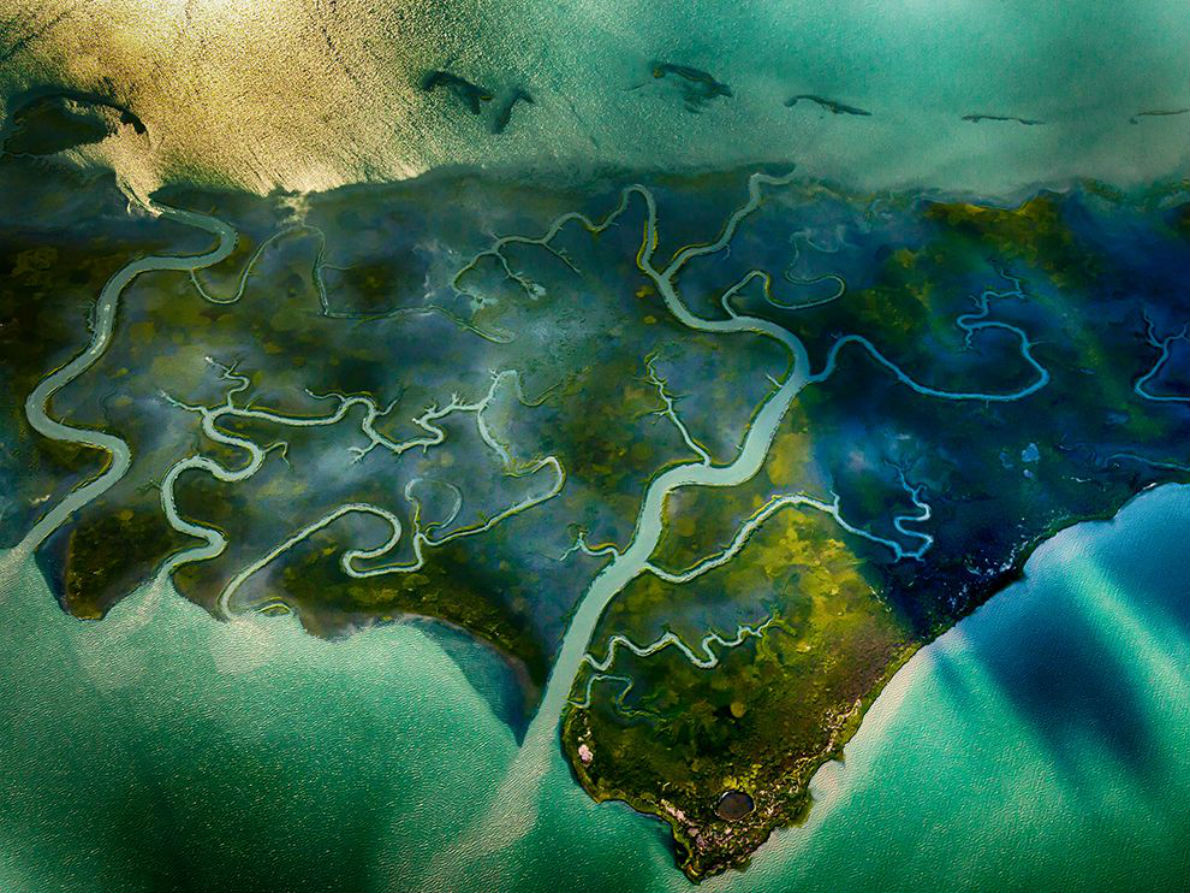

Supplement: Supplementary file 2 [file Data_Sheet_1.zip › Raw Images for Experiment 1/Aerials/aer8.jpg]

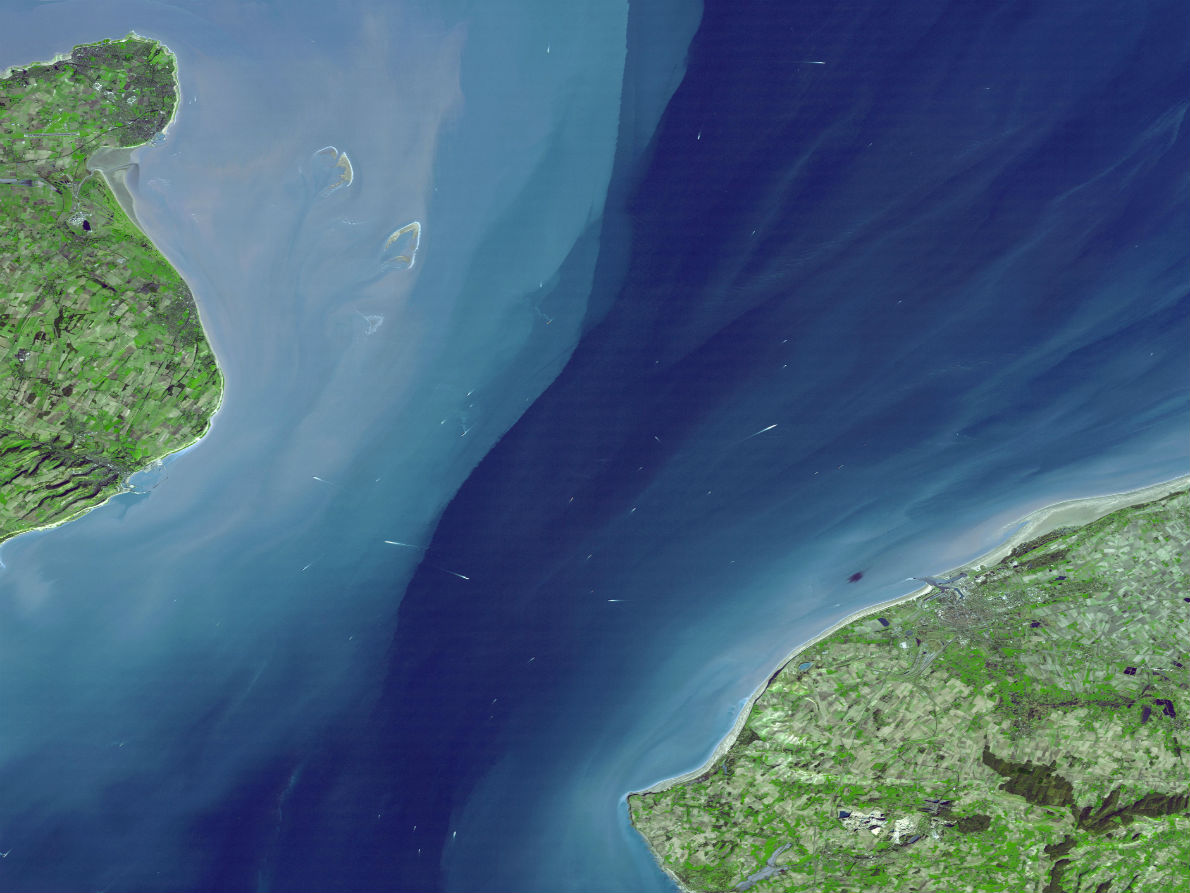

Supplement: Supplementary file 2 [file Data_Sheet_1.zip › Raw Images for Experiment 1/Aerials/aer9.jpg]

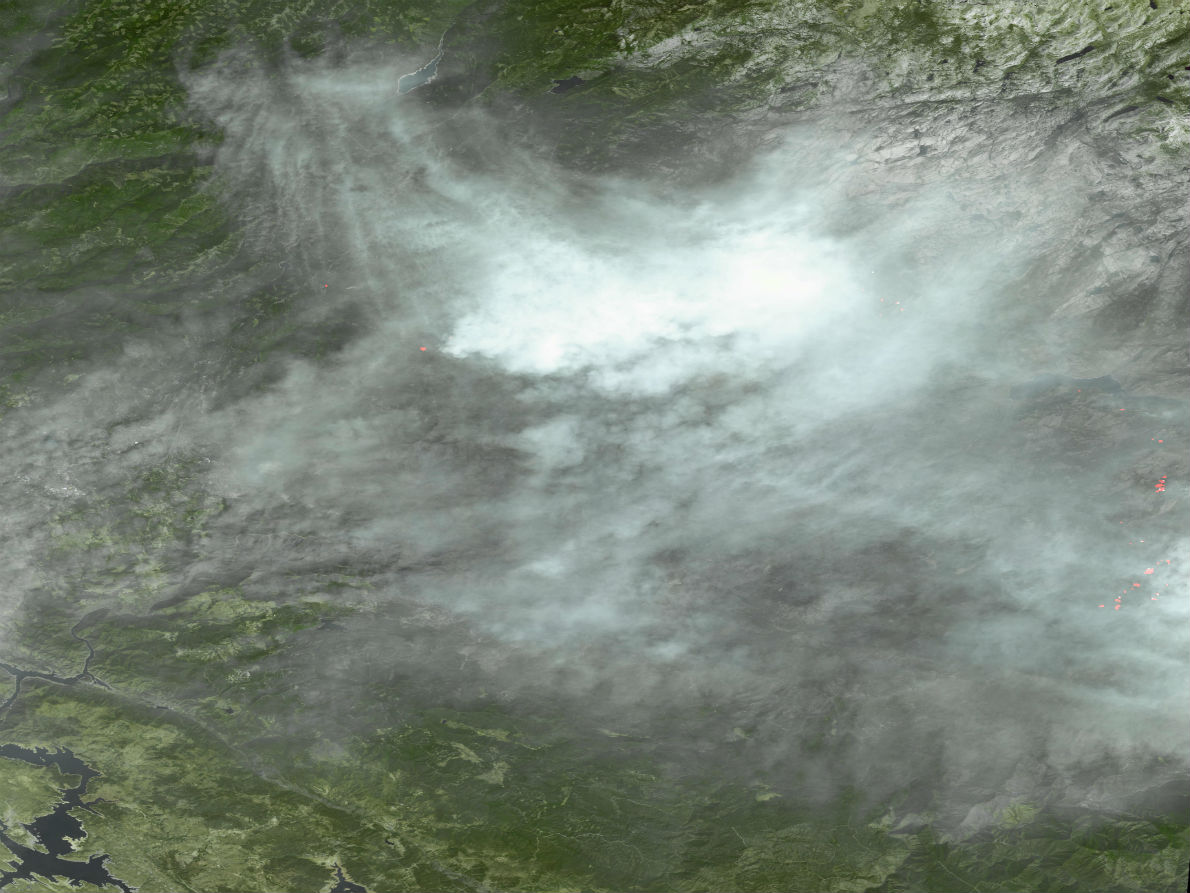

Supplement: Supplementary file 2 [file Data_Sheet_1.zip › Raw Images for Experiment 1/All/aer26.jpg]

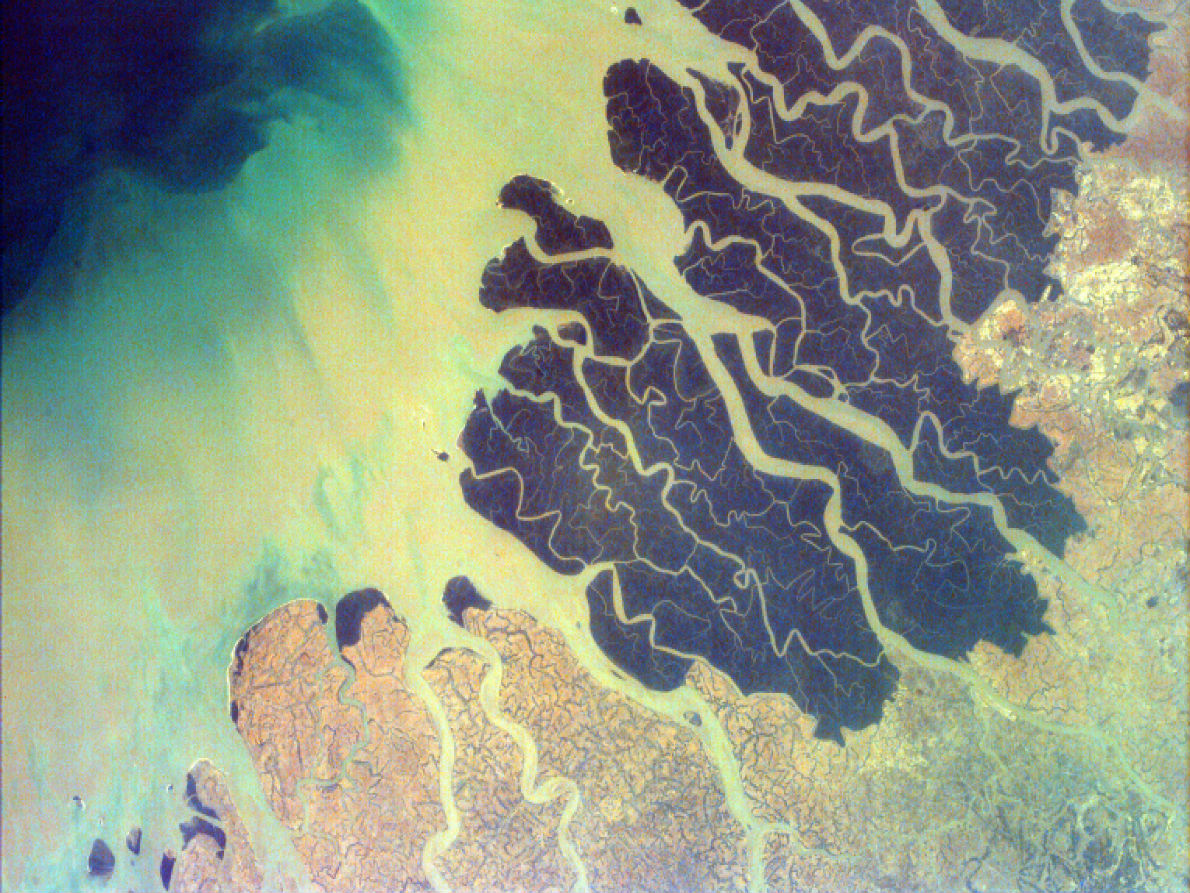

Supplement: Supplementary file 2 [file Data_Sheet_1.zip › Raw Images for Experiment 1/All/aer27.jpg]

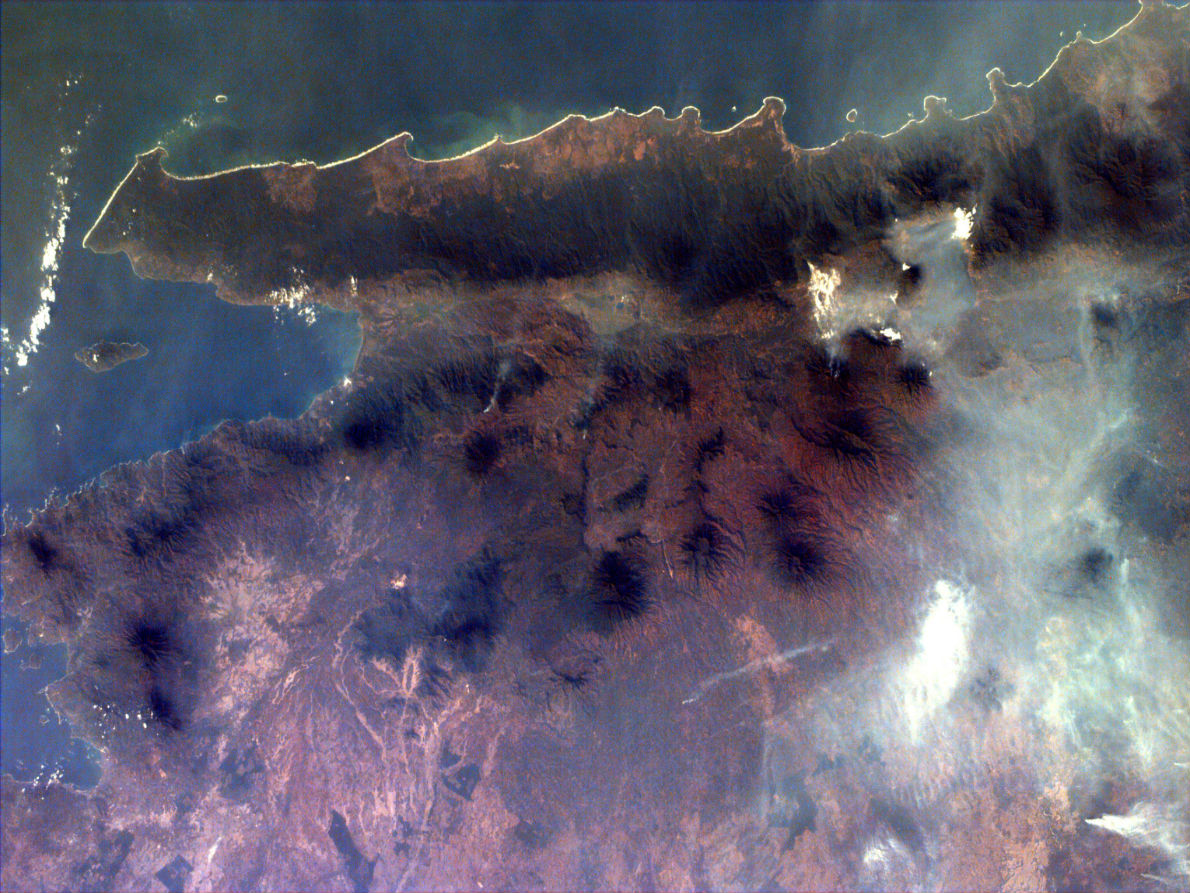

Supplement: Supplementary file 2 [file Data_Sheet_1.zip › Raw Images for Experiment 1/All/aer28.jpg]

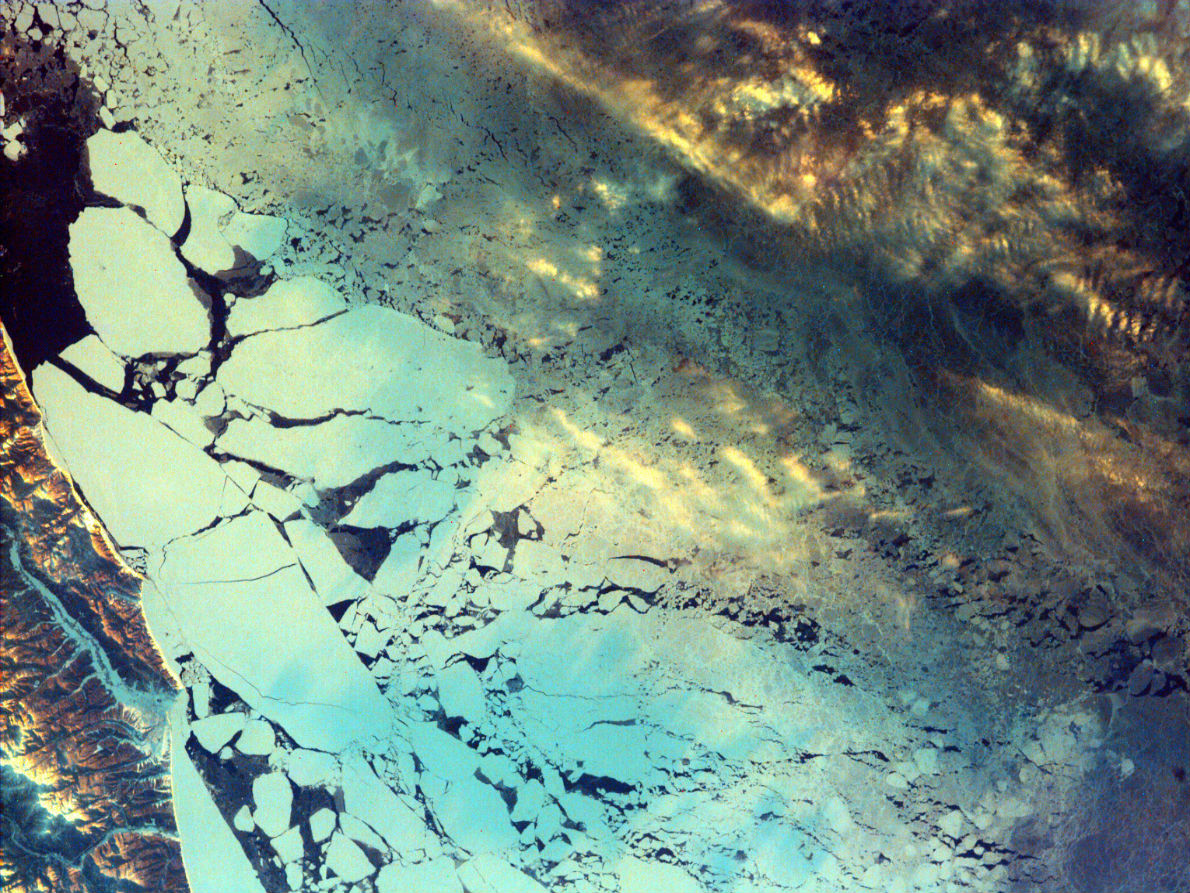

Supplement: Supplementary file 2 [file Data_Sheet_1.zip › Raw Images for Experiment 1/All/aer29.jpg]

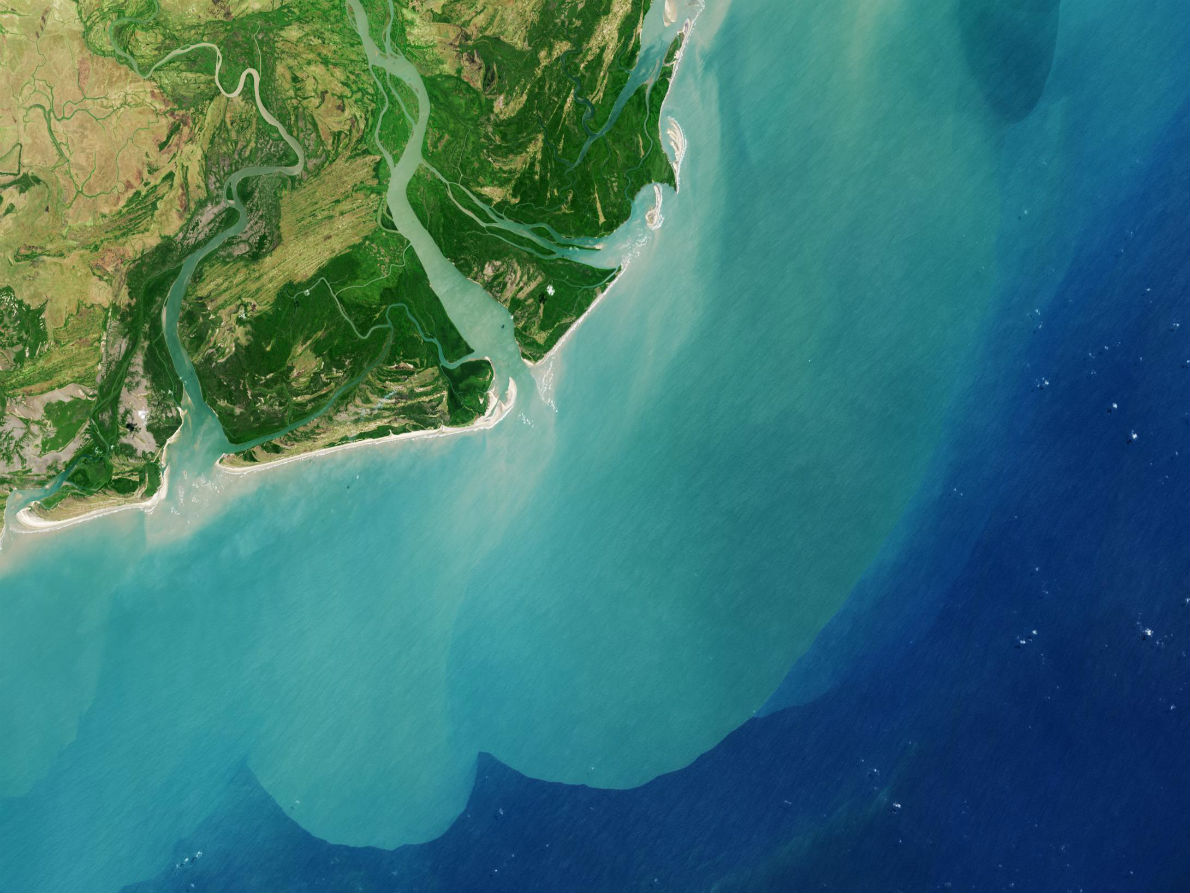

Supplement: Supplementary file 2 [file Data_Sheet_1.zip › Raw Images for Experiment 1/All/aer36.jpg]

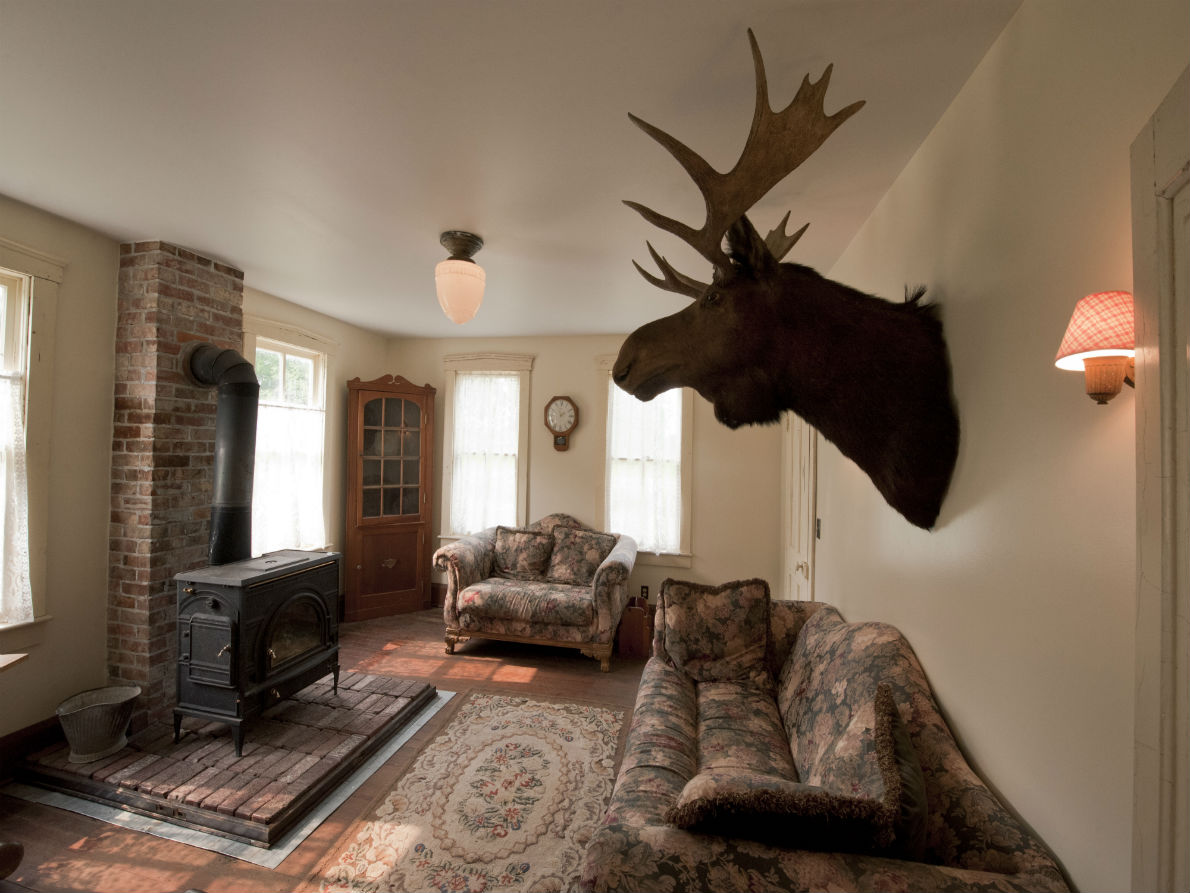

Supplement: Supplementary file 2 [file Data_Sheet_1.zip › Raw Images for Experiment 1/All/id17.jpg]

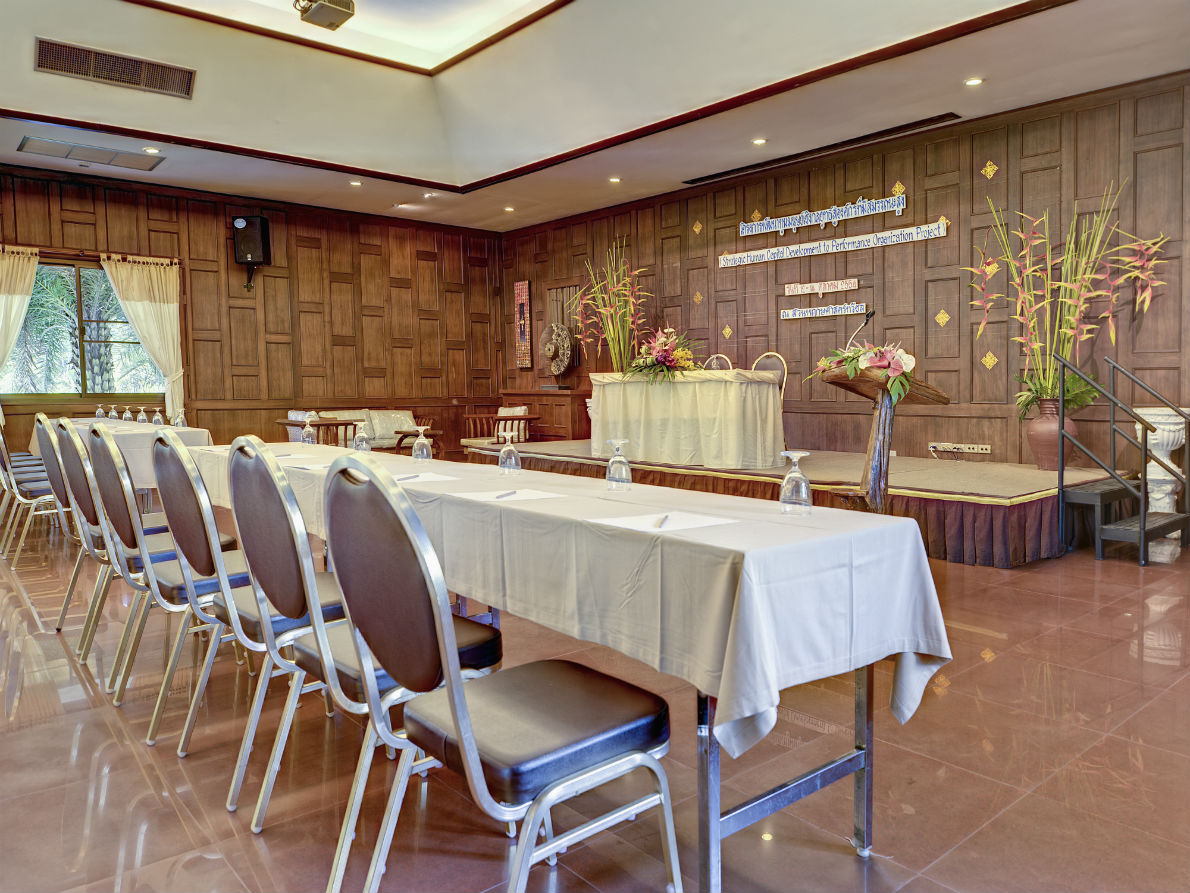

Supplement: Supplementary file 2 [file Data_Sheet_1.zip › Raw Images for Experiment 1/All/id28.jpg]

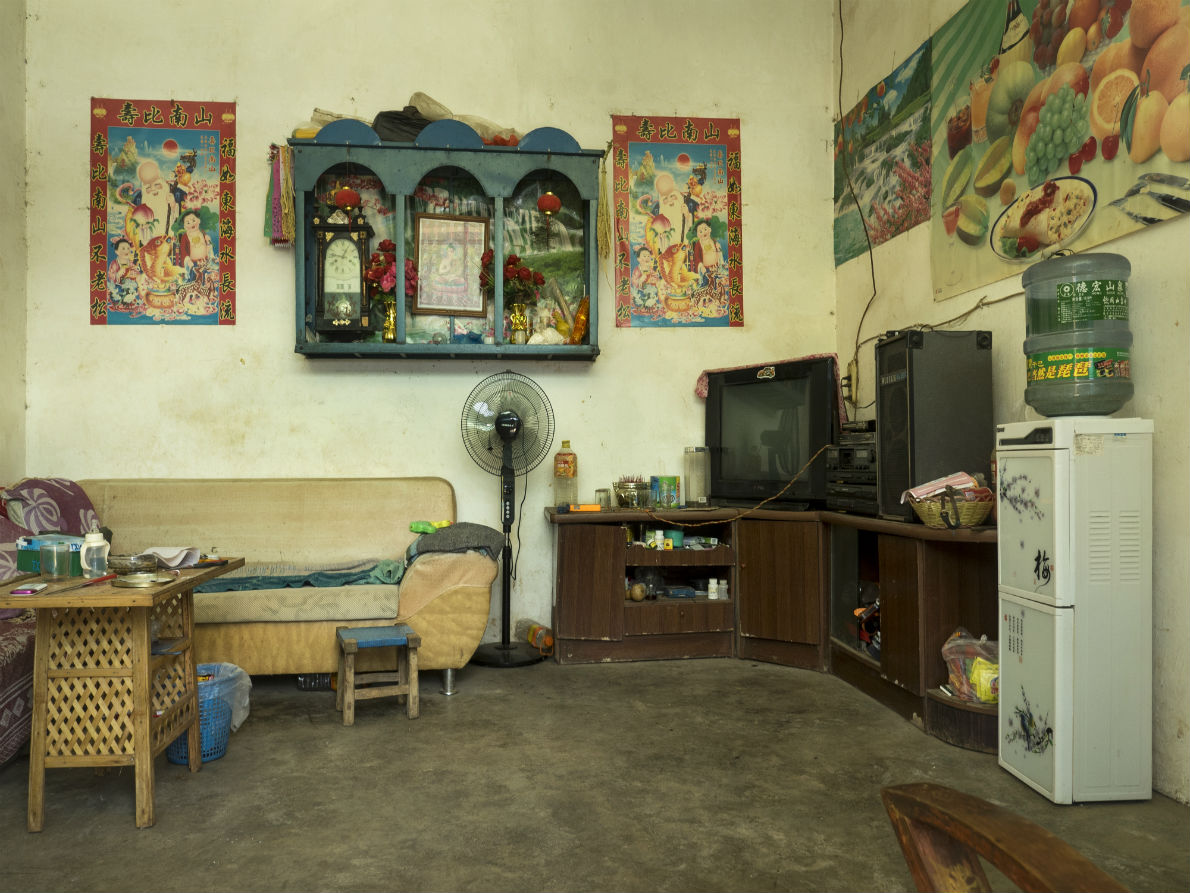

Supplement: Supplementary file 2 [file Data_Sheet_1.zip › Raw Images for Experiment 1/All/id34.jpg]

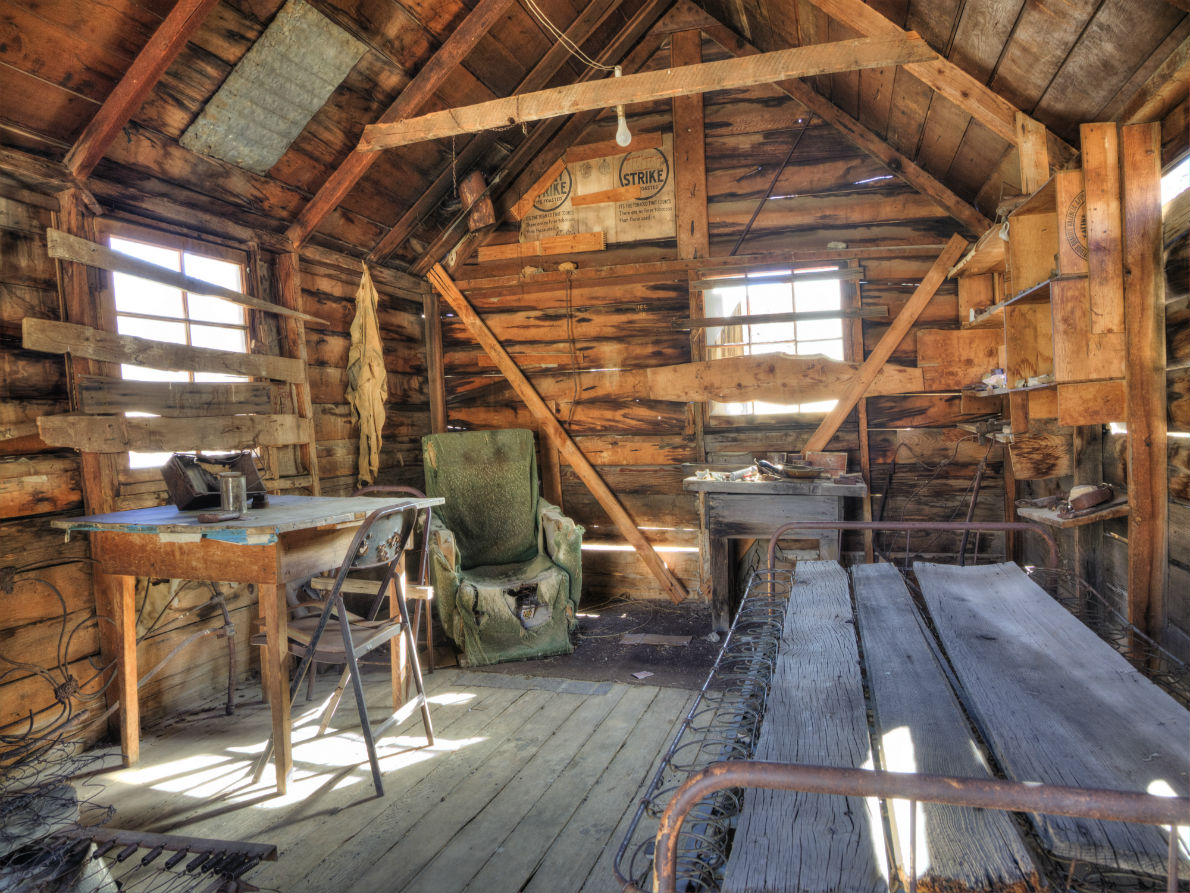

Supplement: Supplementary file 2 [file Data_Sheet_1.zip › Raw Images for Experiment 1/All/id38.jpg]

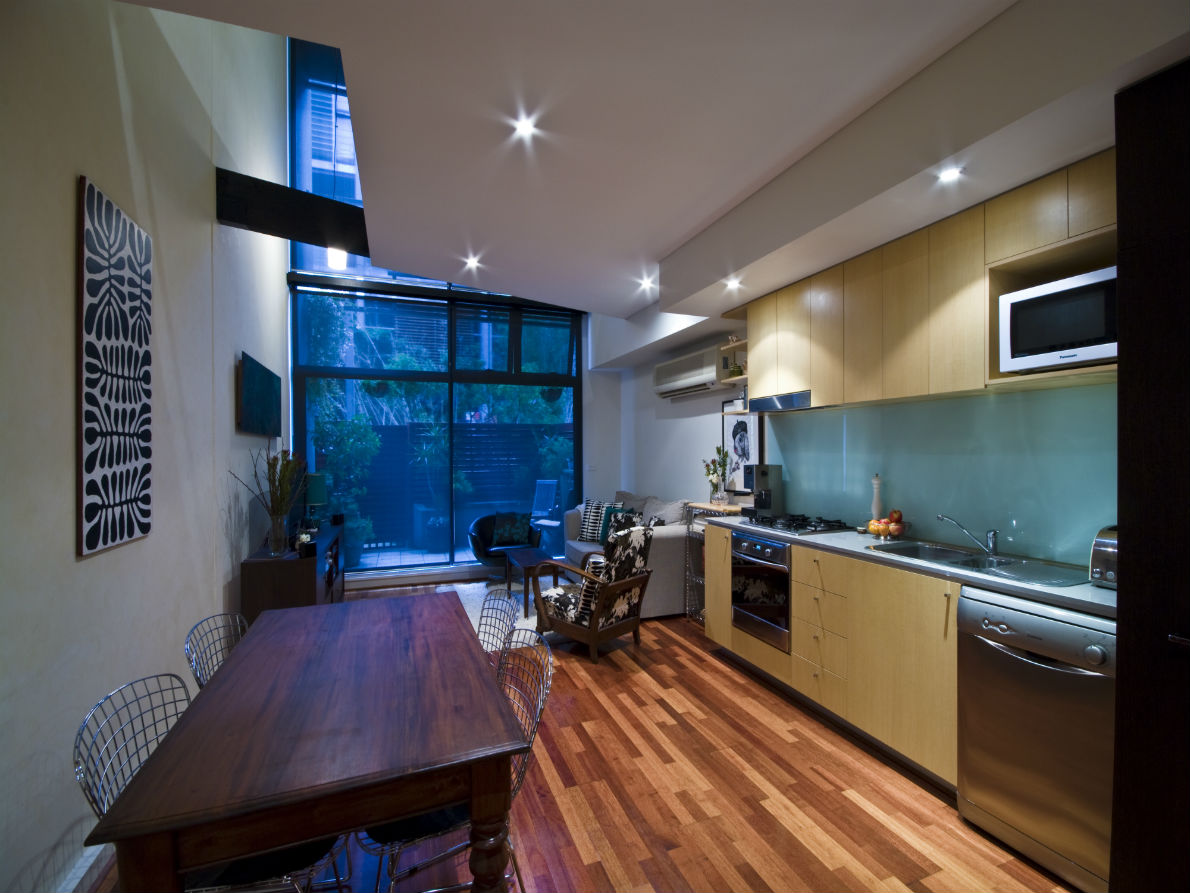

Supplement: Supplementary file 2 [file Data_Sheet_1.zip › Raw Images for Experiment 1/All/id7.jpg]

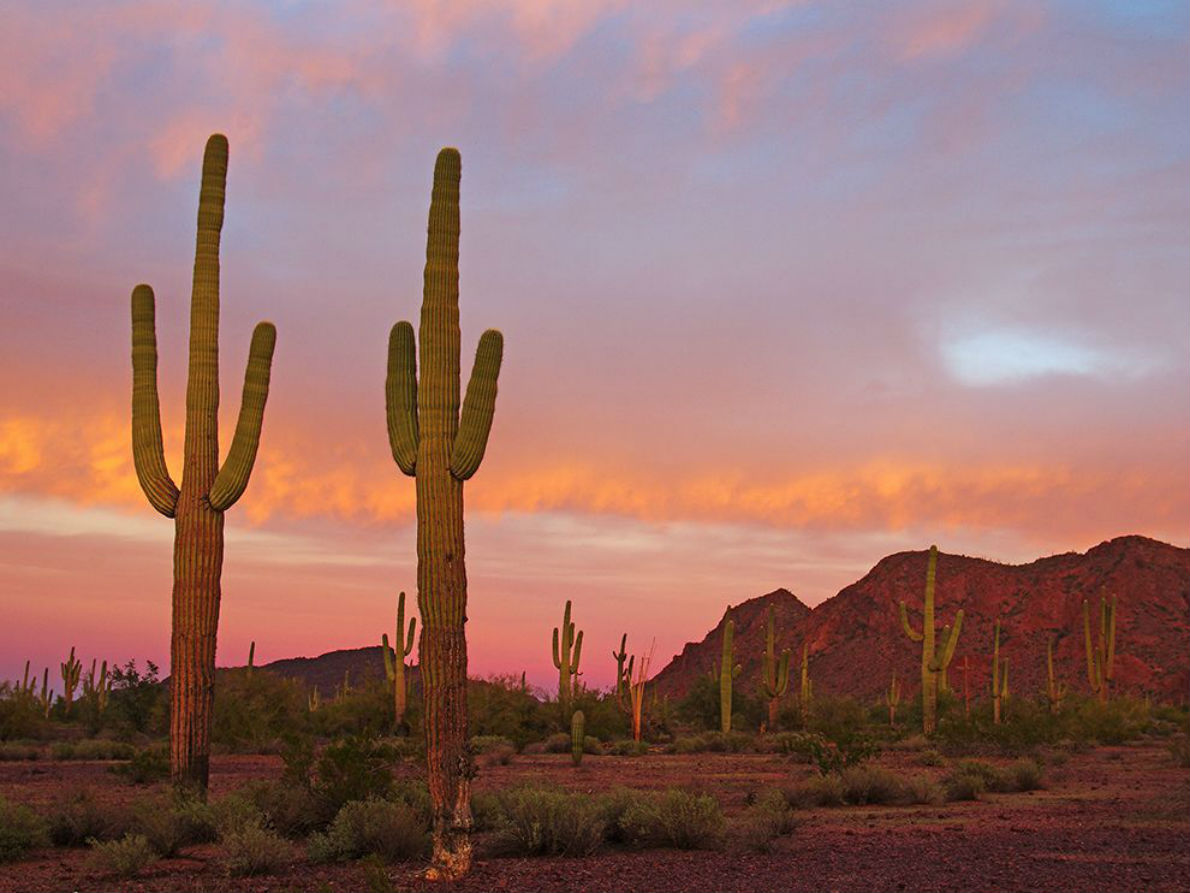

Supplement: Supplementary file 2 [file Data_Sheet_1.zip › Raw Images for Experiment 1/All/ls26.jpg]

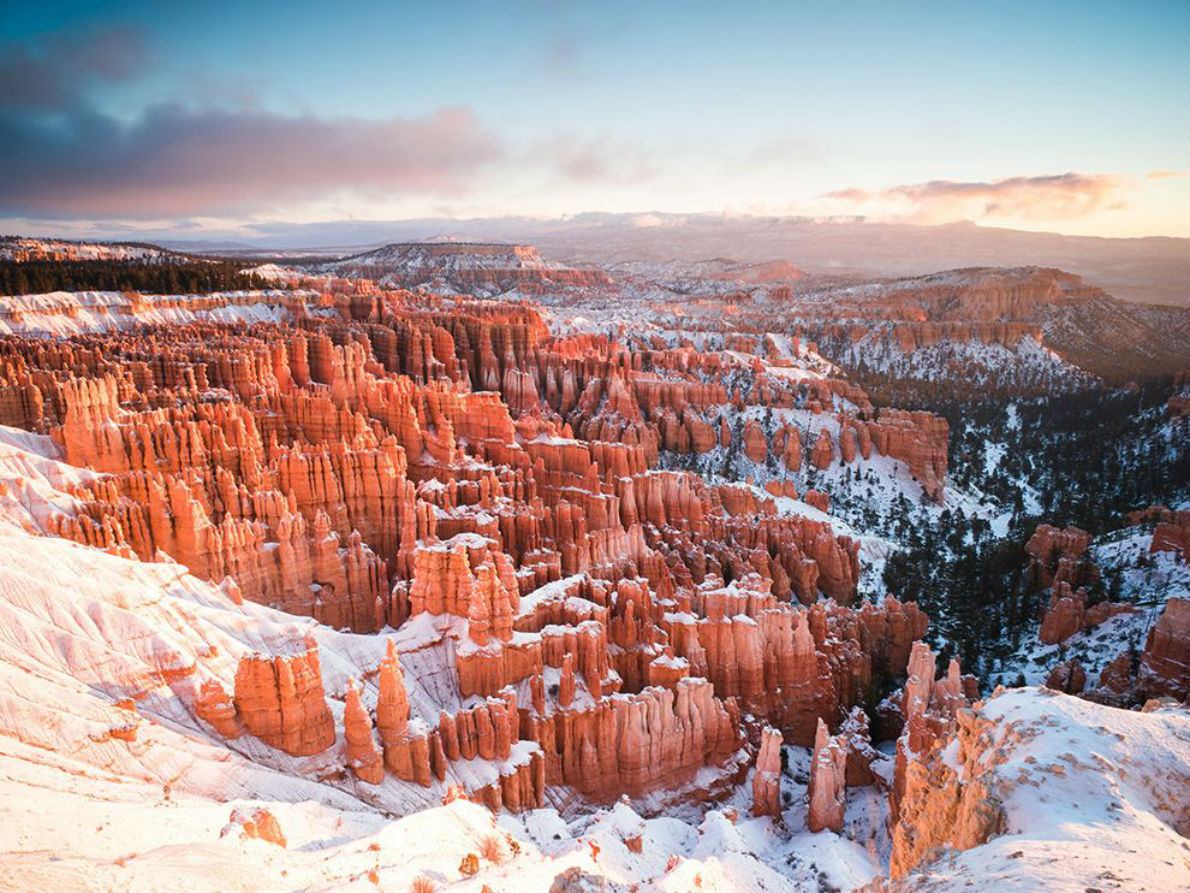

Supplement: Supplementary file 2 [file Data_Sheet_1.zip › Raw Images for Experiment 1/All/ls27.jpg]

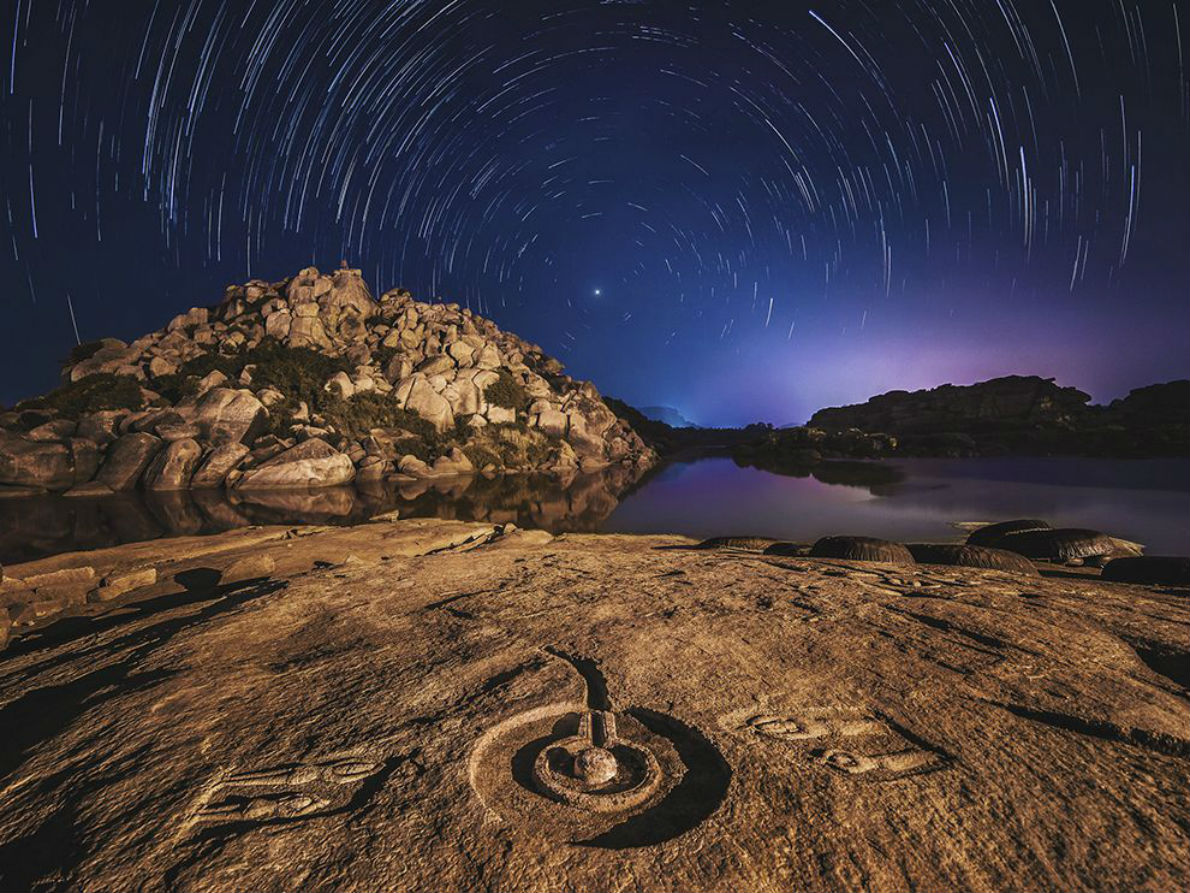

Supplement: Supplementary file 2 [file Data_Sheet_1.zip › Raw Images for Experiment 1/All/ls28.jpg]

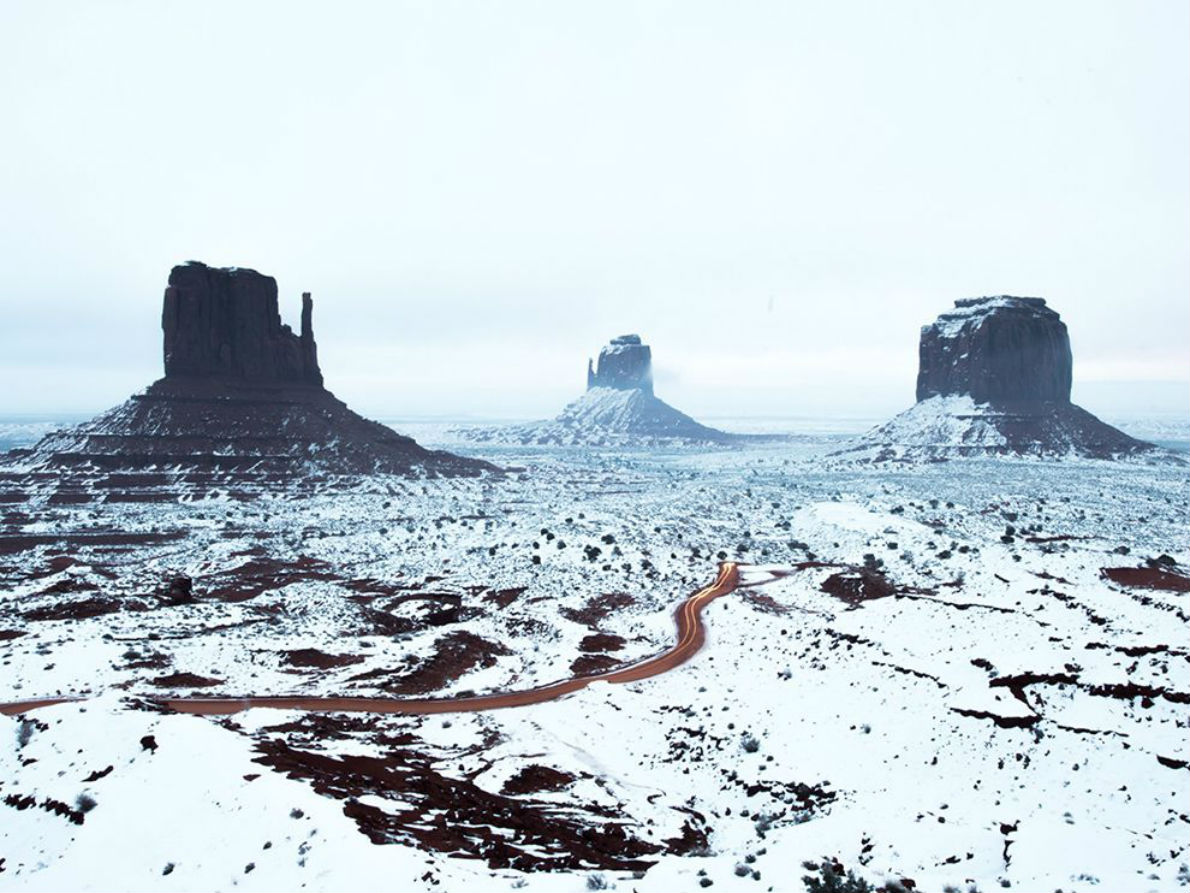

Supplement: Supplementary file 2 [file Data_Sheet_1.zip › Raw Images for Experiment 1/All/ls29.jpg]

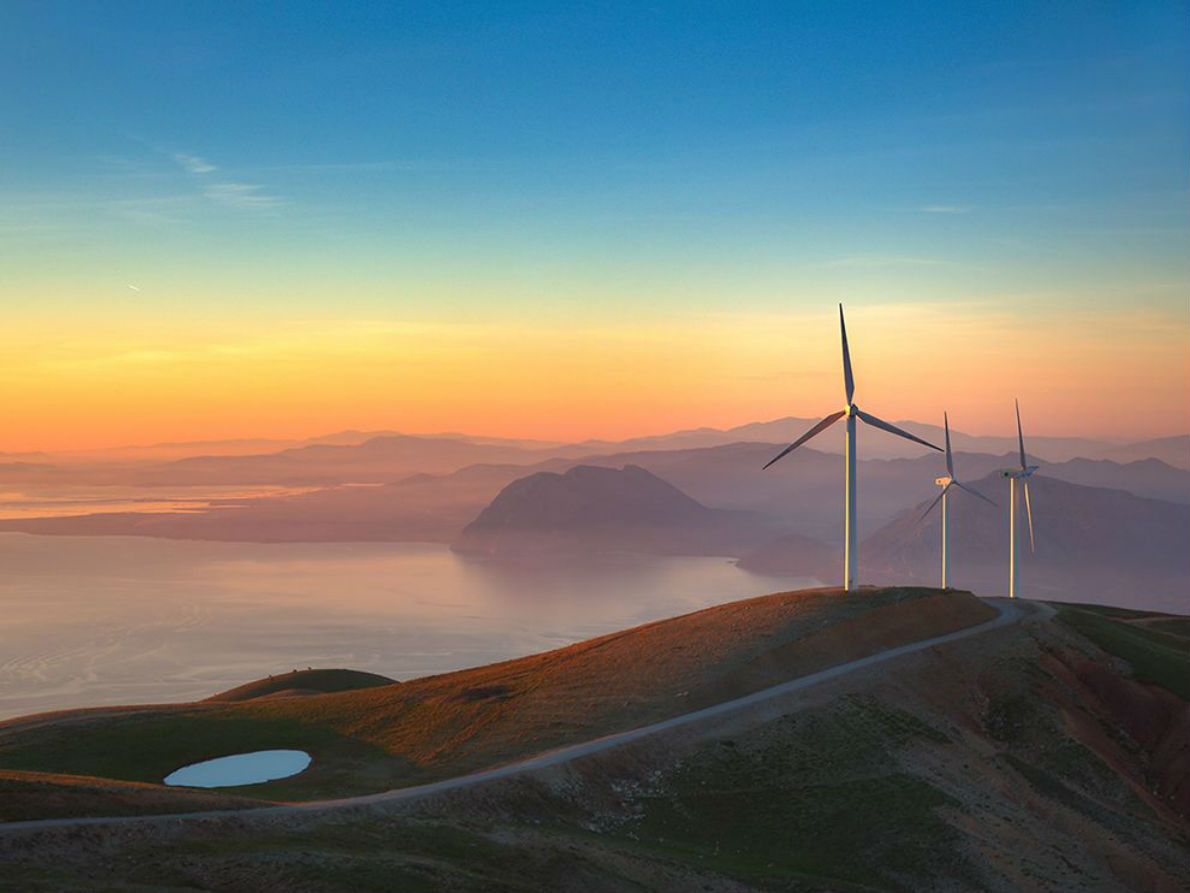

Supplement: Supplementary file 2 [file Data_Sheet_1.zip › Raw Images for Experiment 1/All/ls30.jpg]

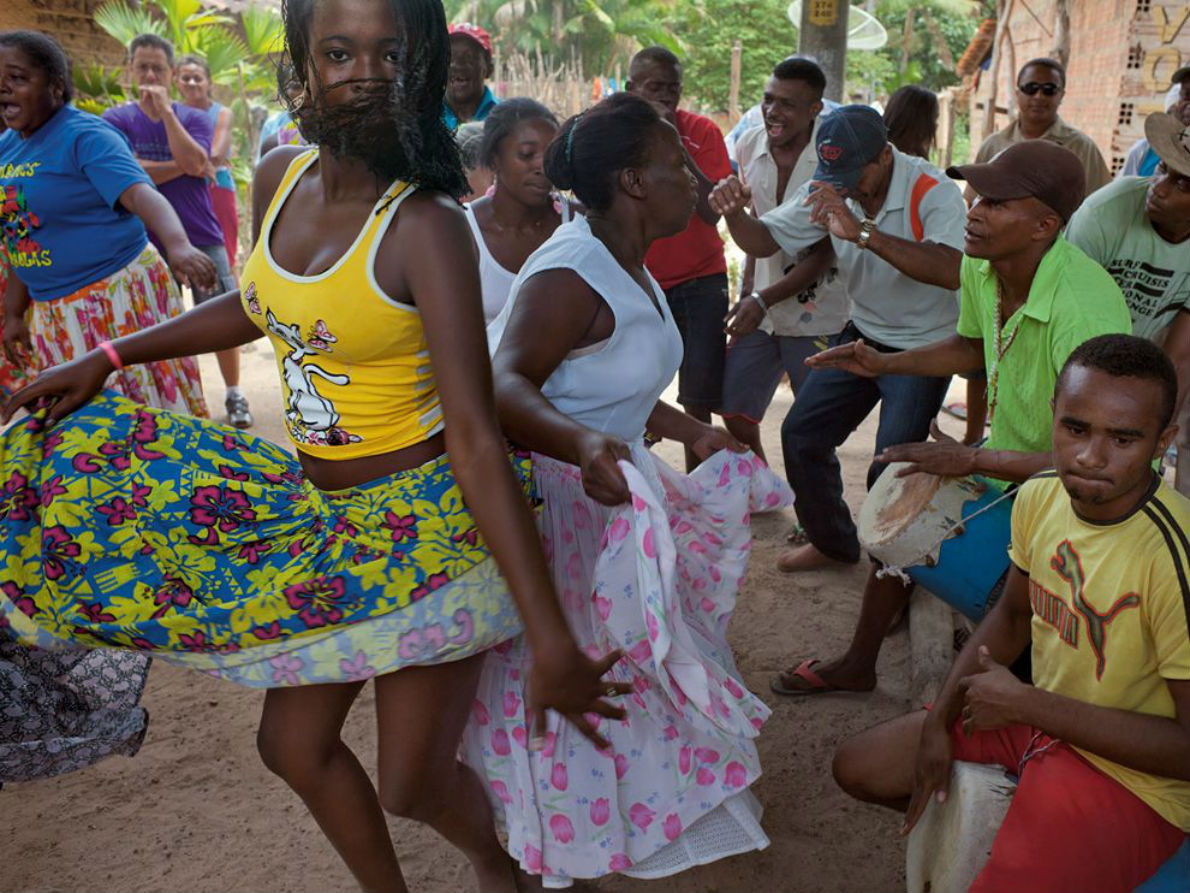

Supplement: Supplementary file 2 [file Data_Sheet_1.zip › Raw Images for Experiment 1/All/ppl29.jpg]

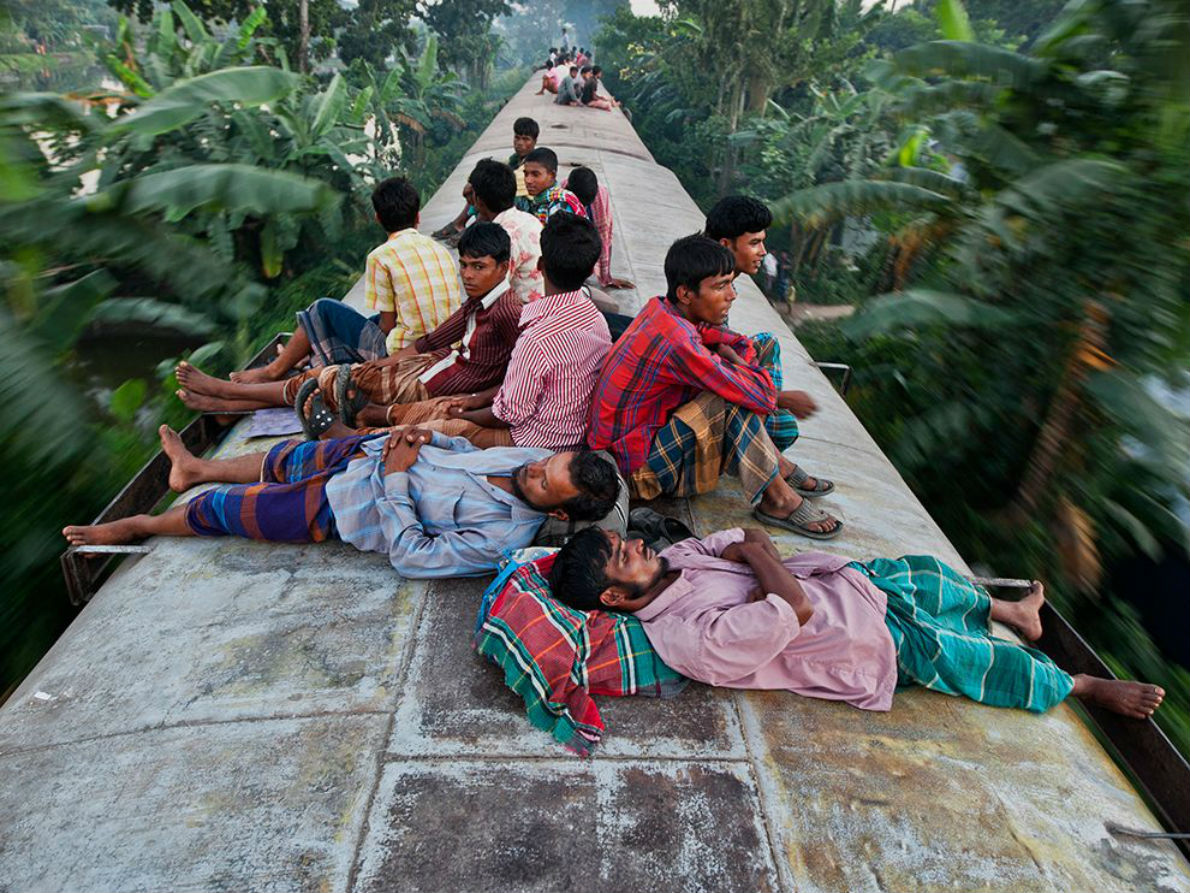

Supplement: Supplementary file 2 [file Data_Sheet_1.zip › Raw Images for Experiment 1/All/ppl30.jpg]

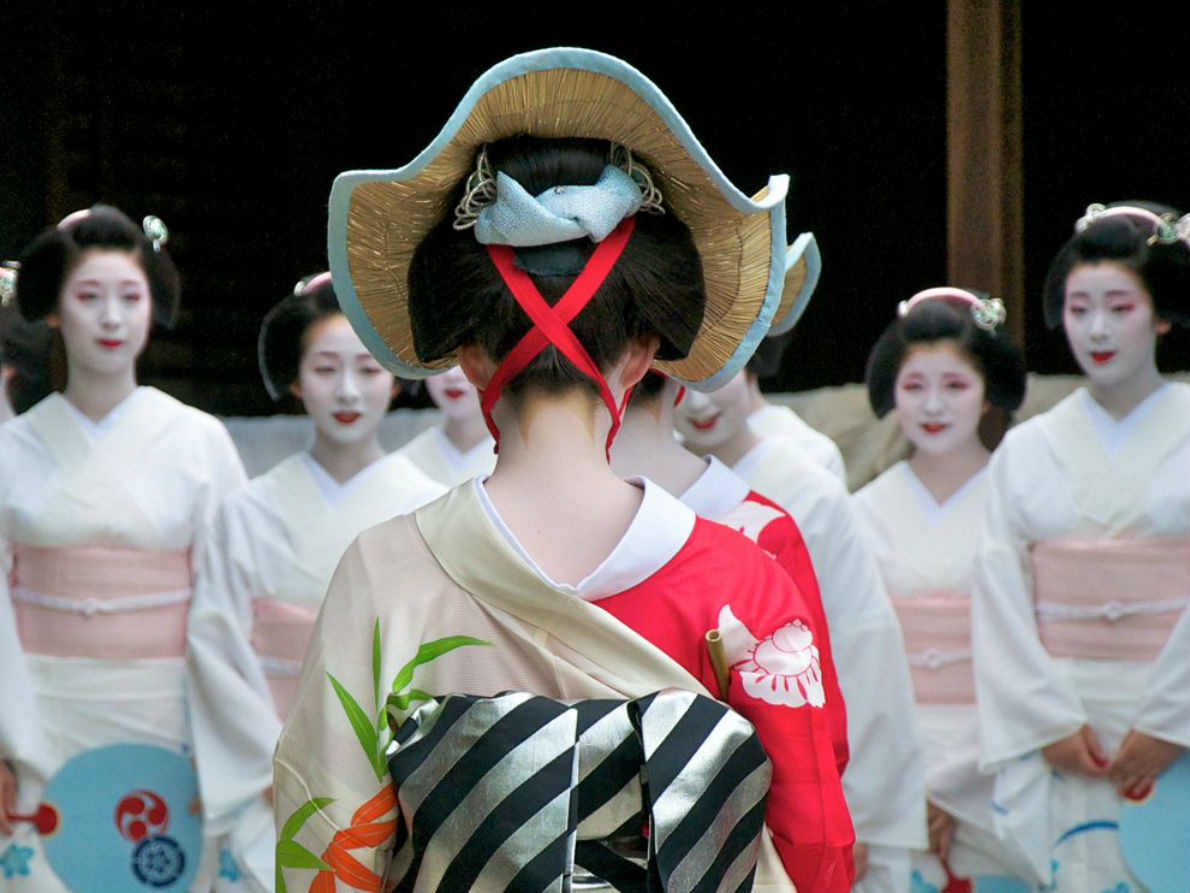

Supplement: Supplementary file 2 [file Data_Sheet_1.zip › Raw Images for Experiment 1/All/ppl31.jpg]

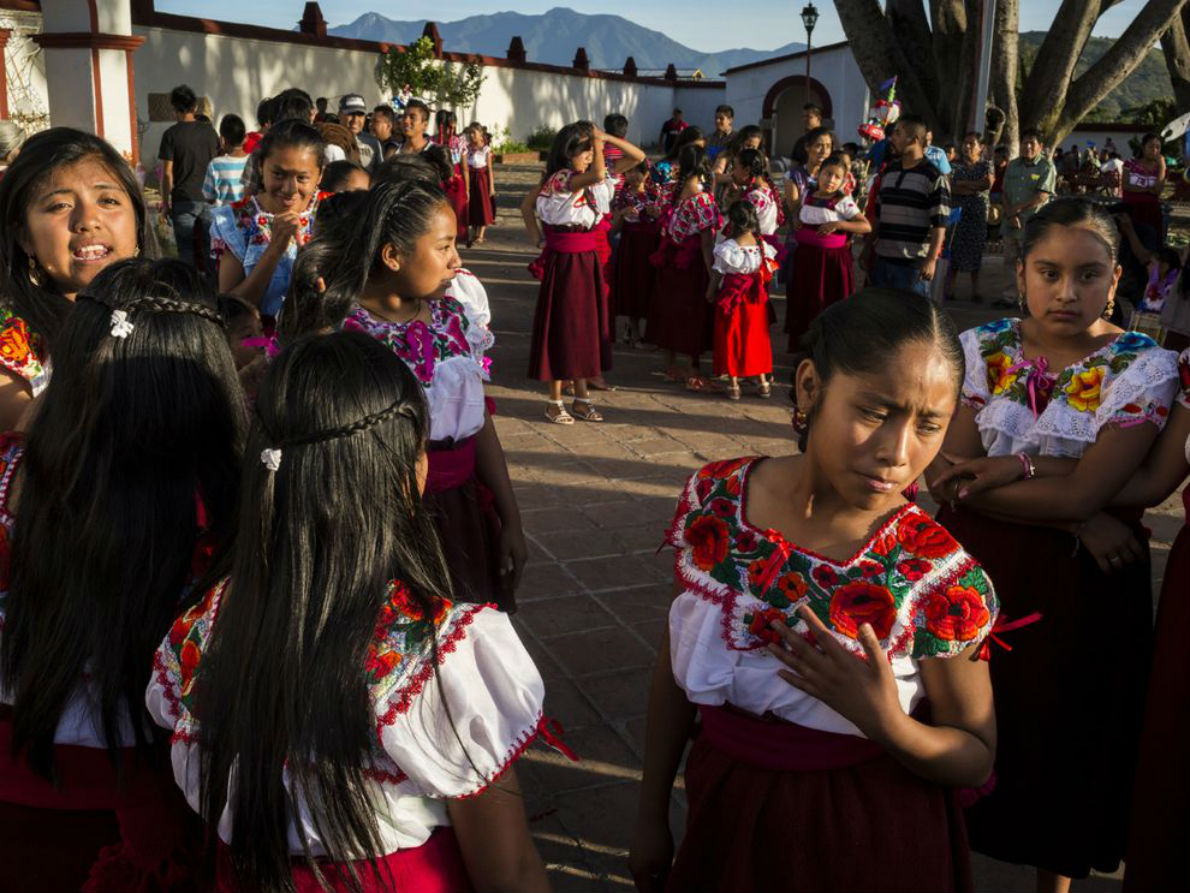

Supplement: Supplementary file 2 [file Data_Sheet_1.zip › Raw Images for Experiment 1/All/ppl32.jpg]

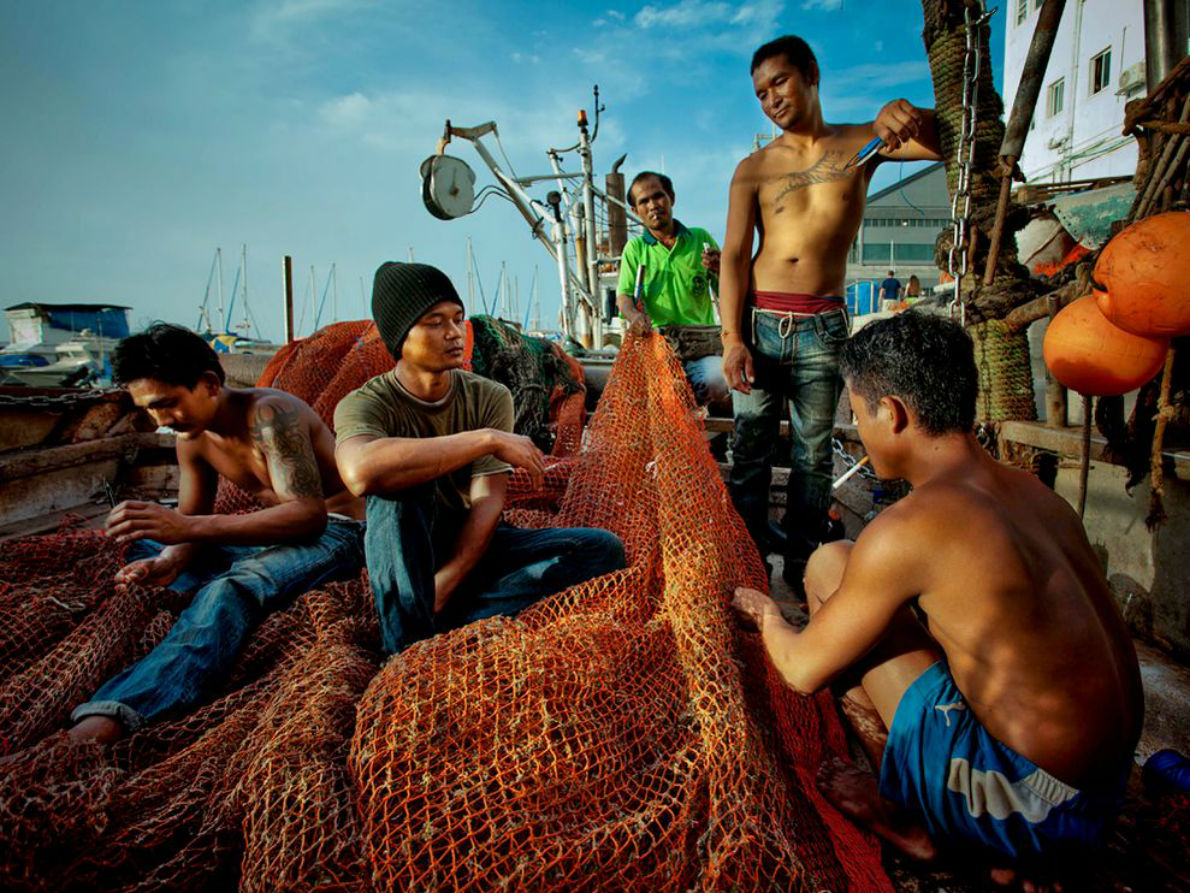

Supplement: Supplementary file 2 [file Data_Sheet_1.zip › Raw Images for Experiment 1/All/ppl33.jpg]

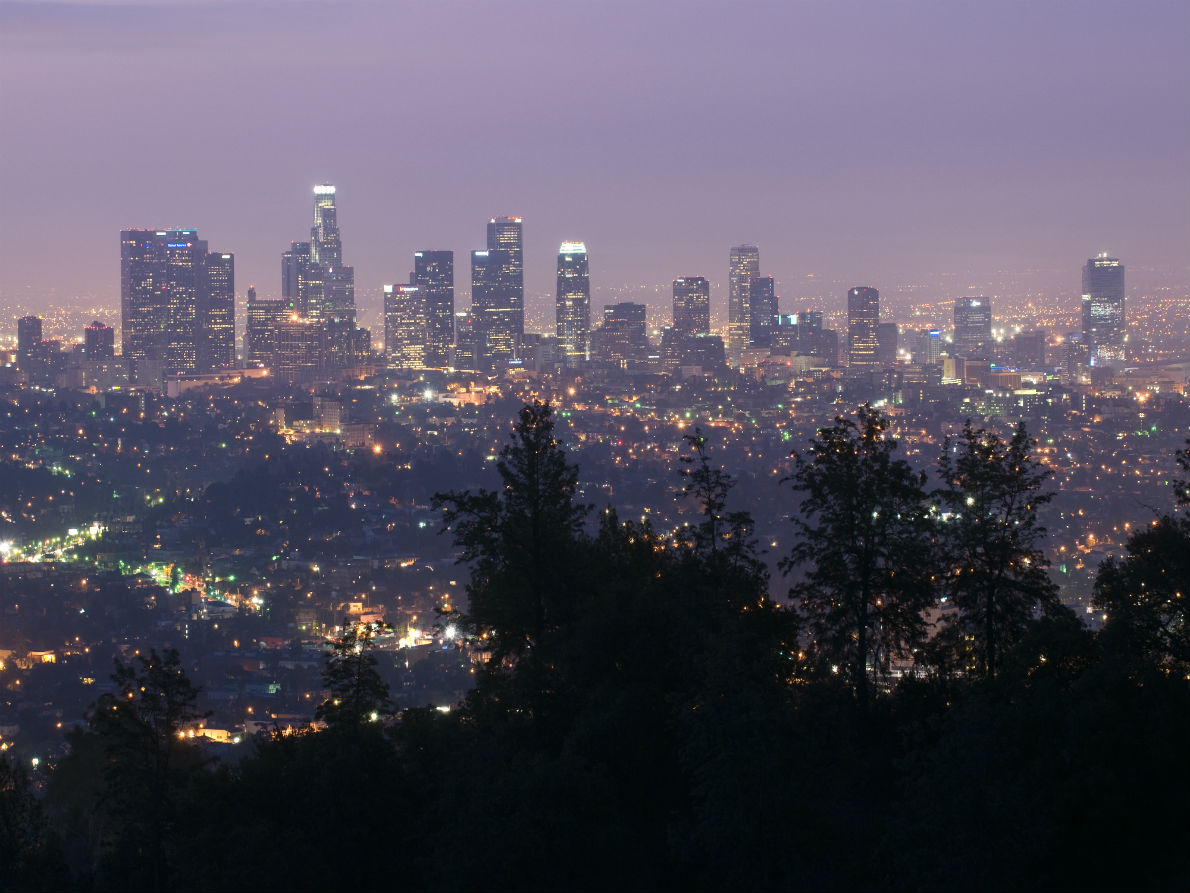

Supplement: Supplementary file 2 [file Data_Sheet_1.zip › Raw Images for Experiment 1/All/sky12.jpg]

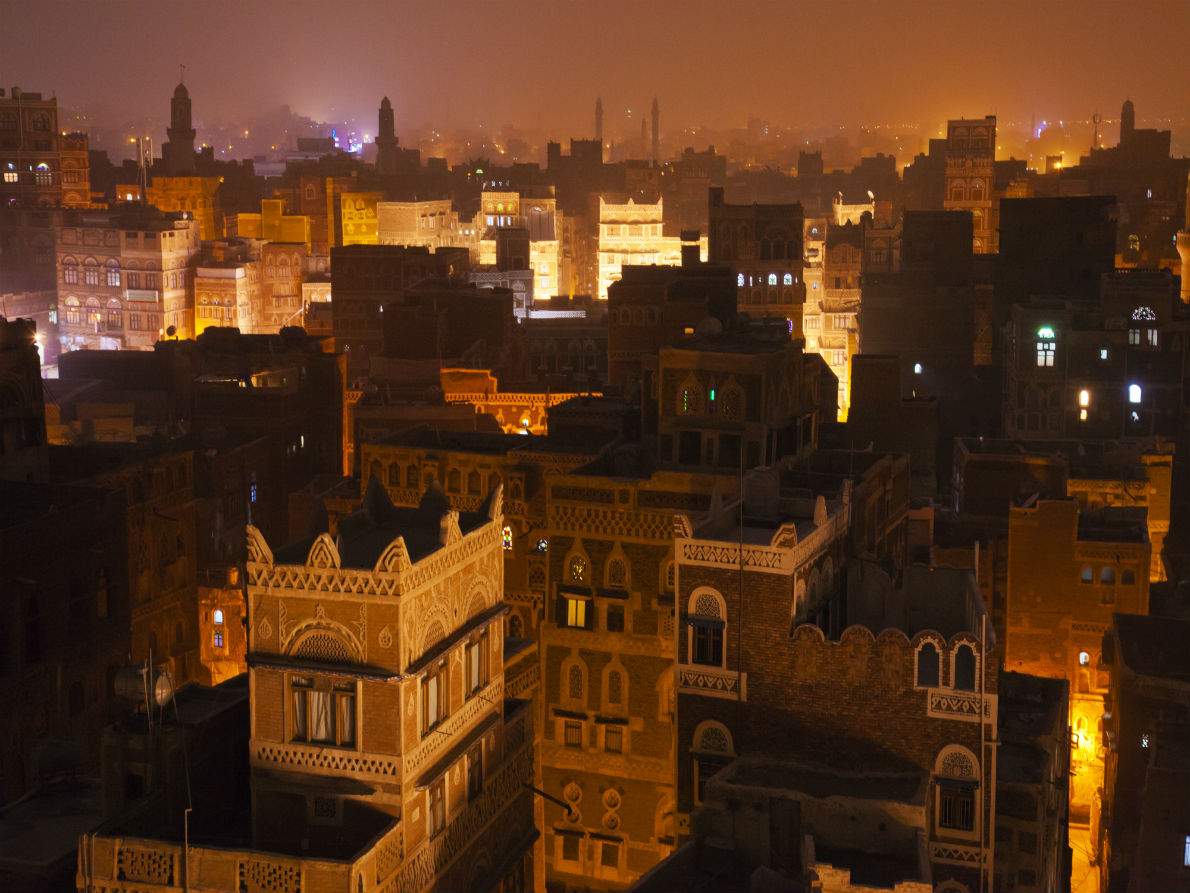

Supplement: Supplementary file 2 [file Data_Sheet_1.zip › Raw Images for Experiment 1/All/sky14.jpg]

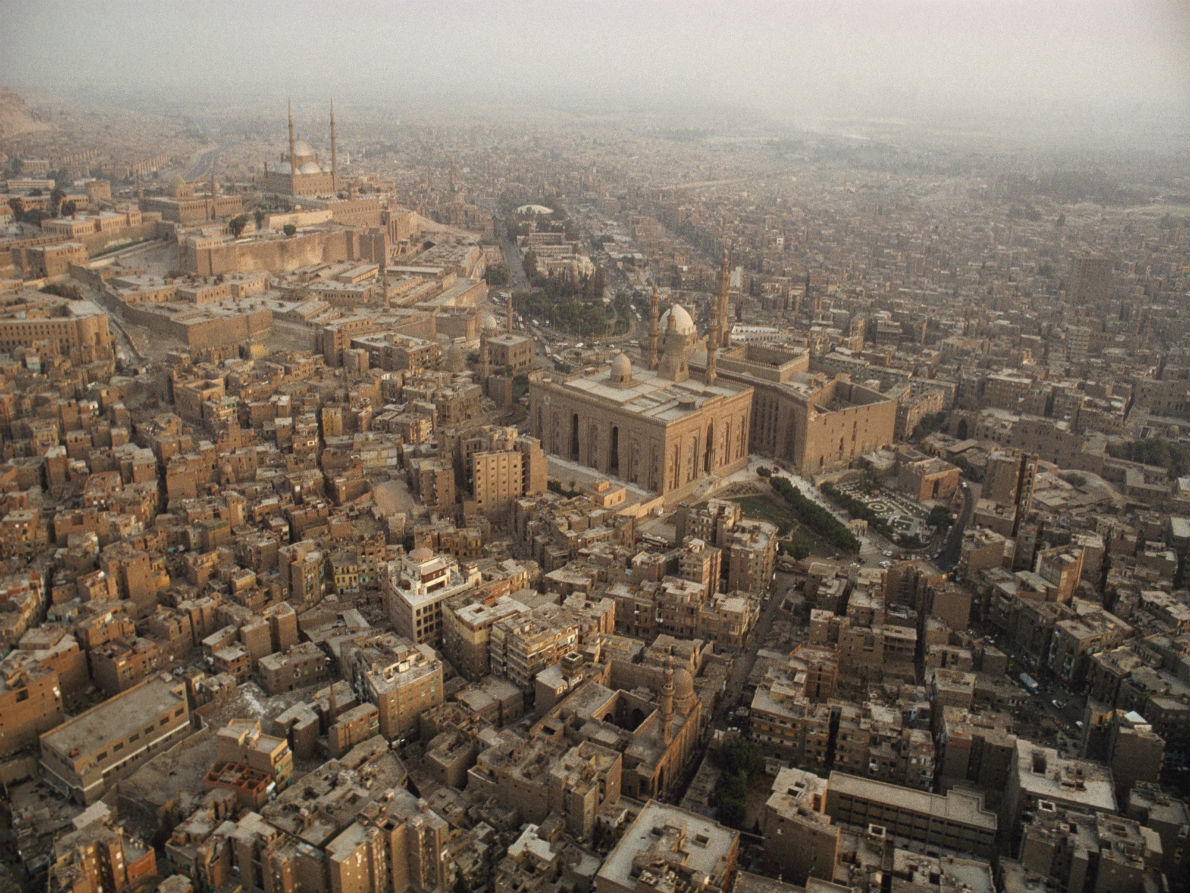

Supplement: Supplementary file 2 [file Data_Sheet_1.zip › Raw Images for Experiment 1/All/sky2.jpg]

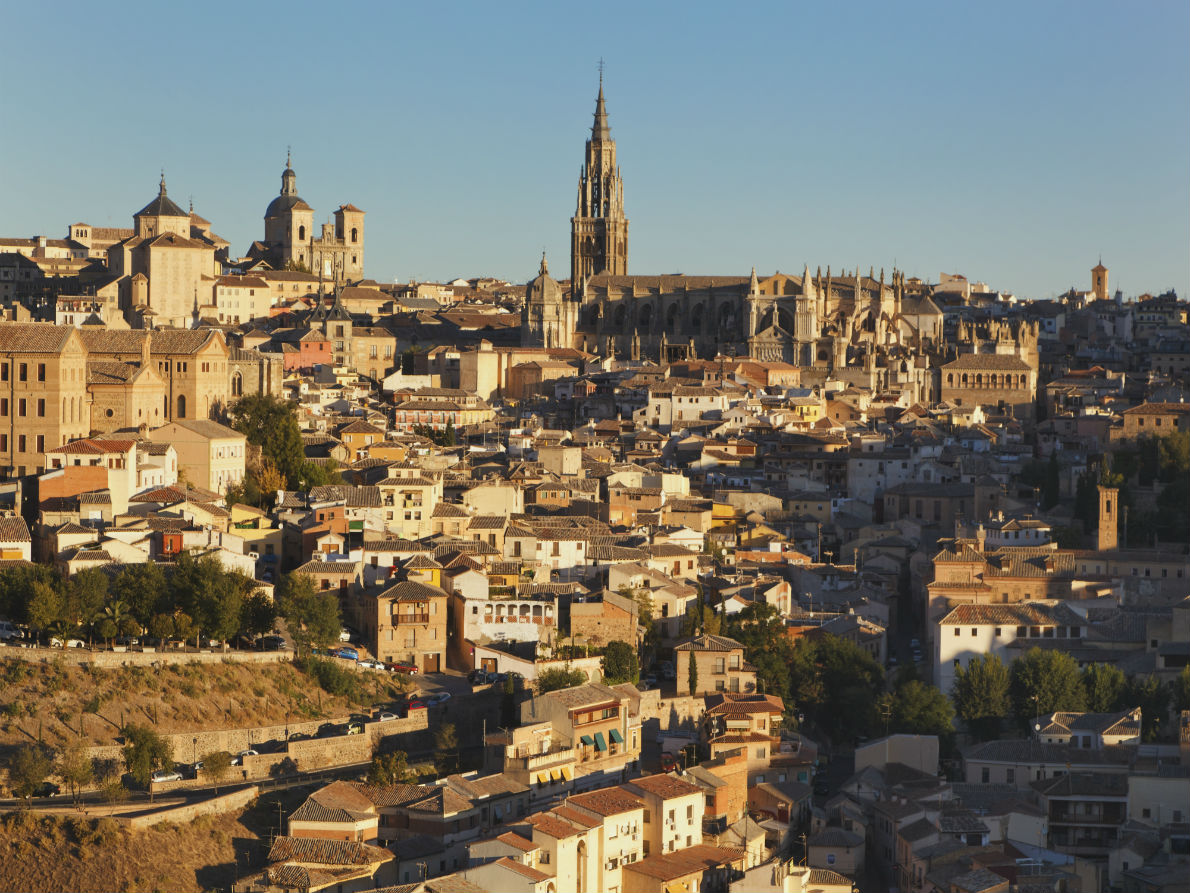

Supplement: Supplementary file 2 [file Data_Sheet_1.zip › Raw Images for Experiment 1/All/sky25.jpg]

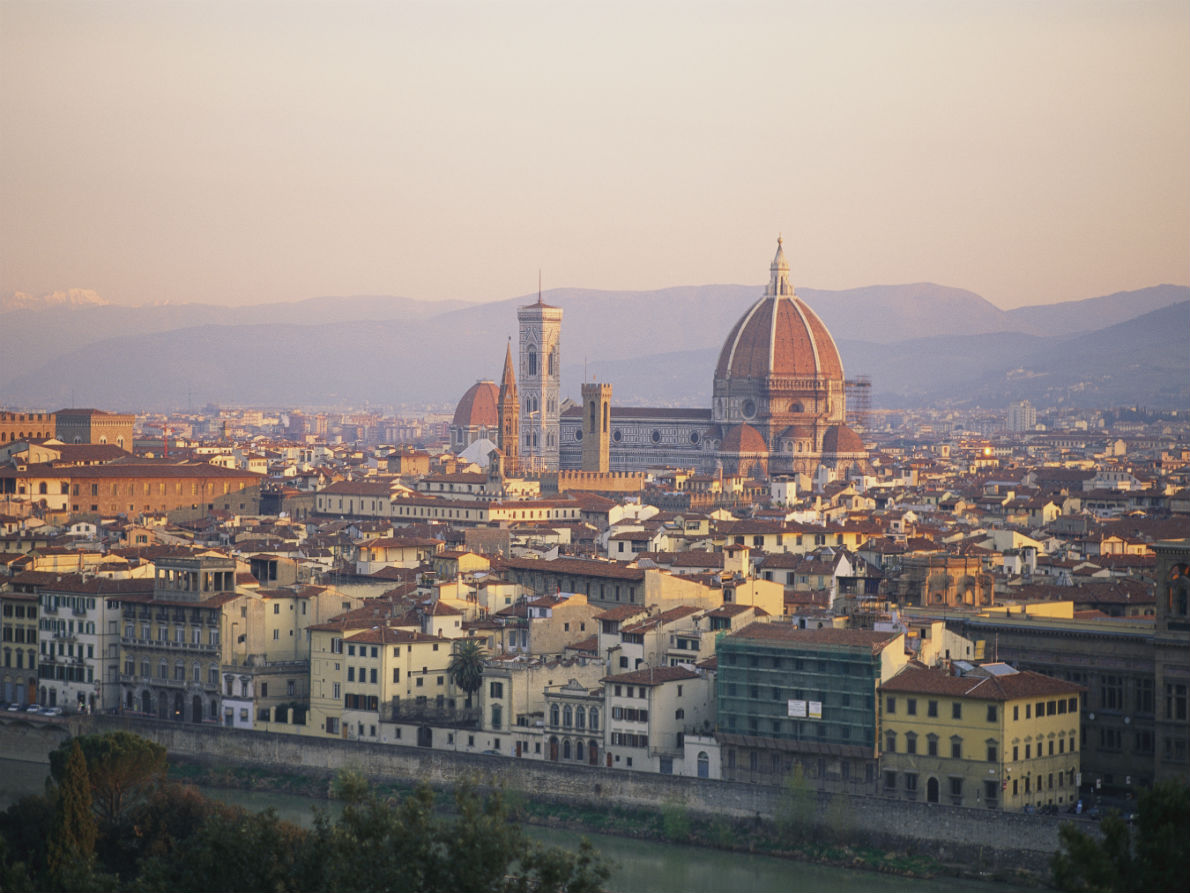

Supplement: Supplementary file 2 [file Data_Sheet_1.zip › Raw Images for Experiment 1/All/sky6.jpg]

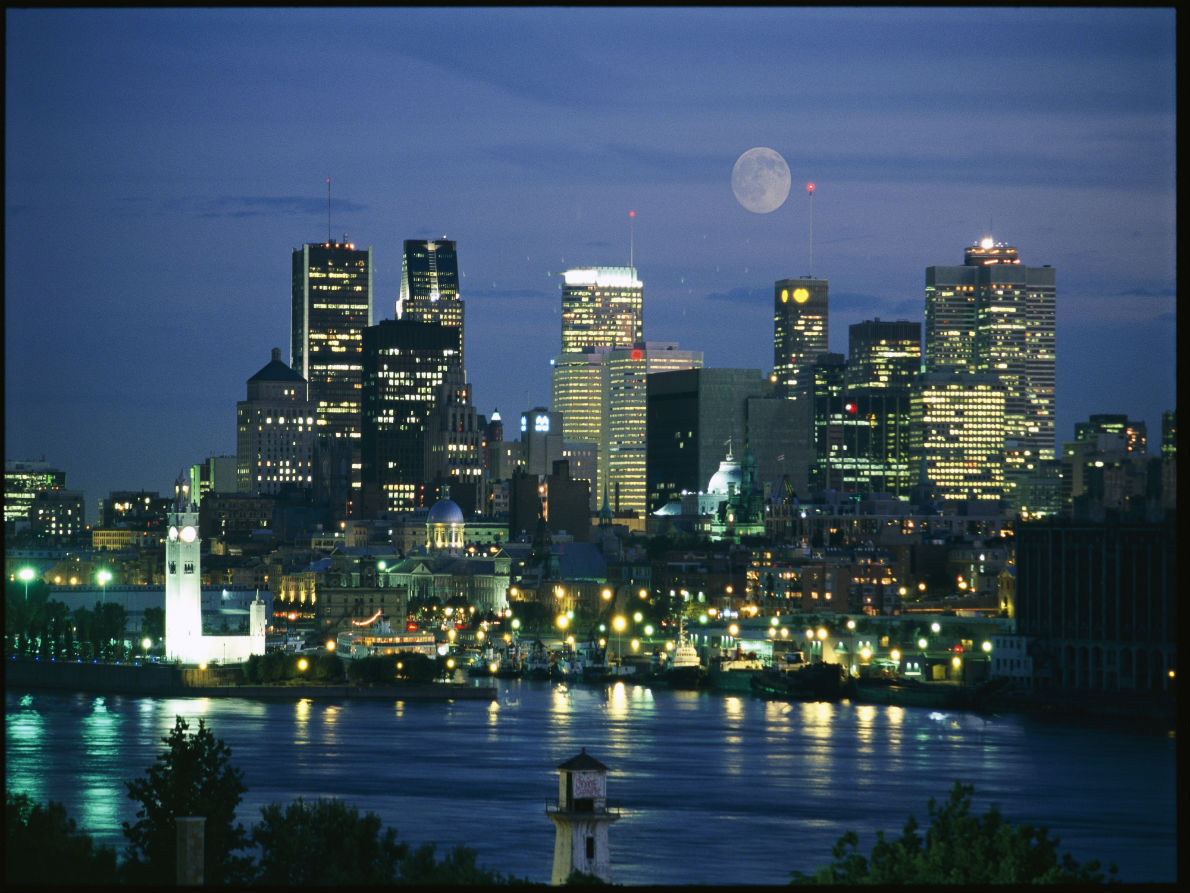

Supplement: Supplementary file 2 [file Data_Sheet_1.zip › Raw Images for Experiment 1/Cityscapes/city1.jpg]

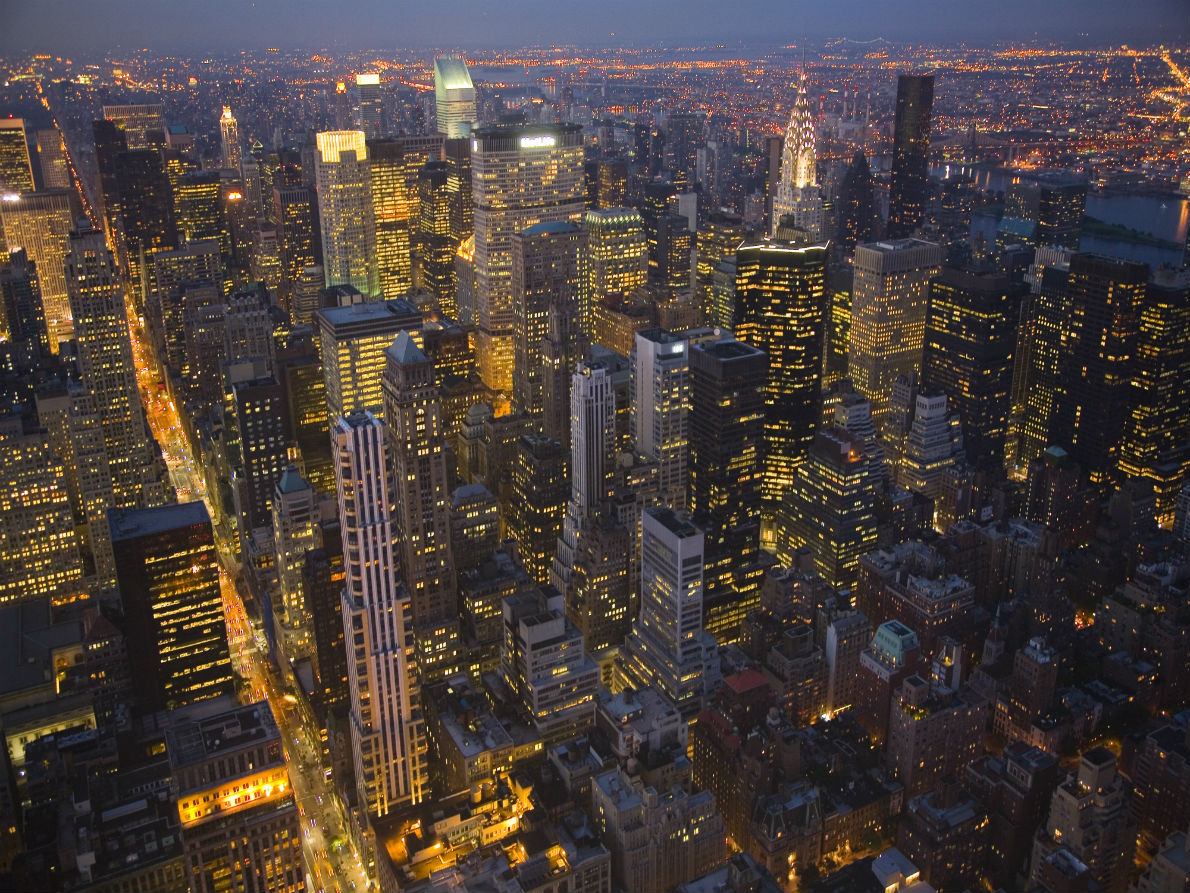

Supplement: Supplementary file 2 [file Data_Sheet_1.zip › Raw Images for Experiment 1/Cityscapes/city11.jpg]

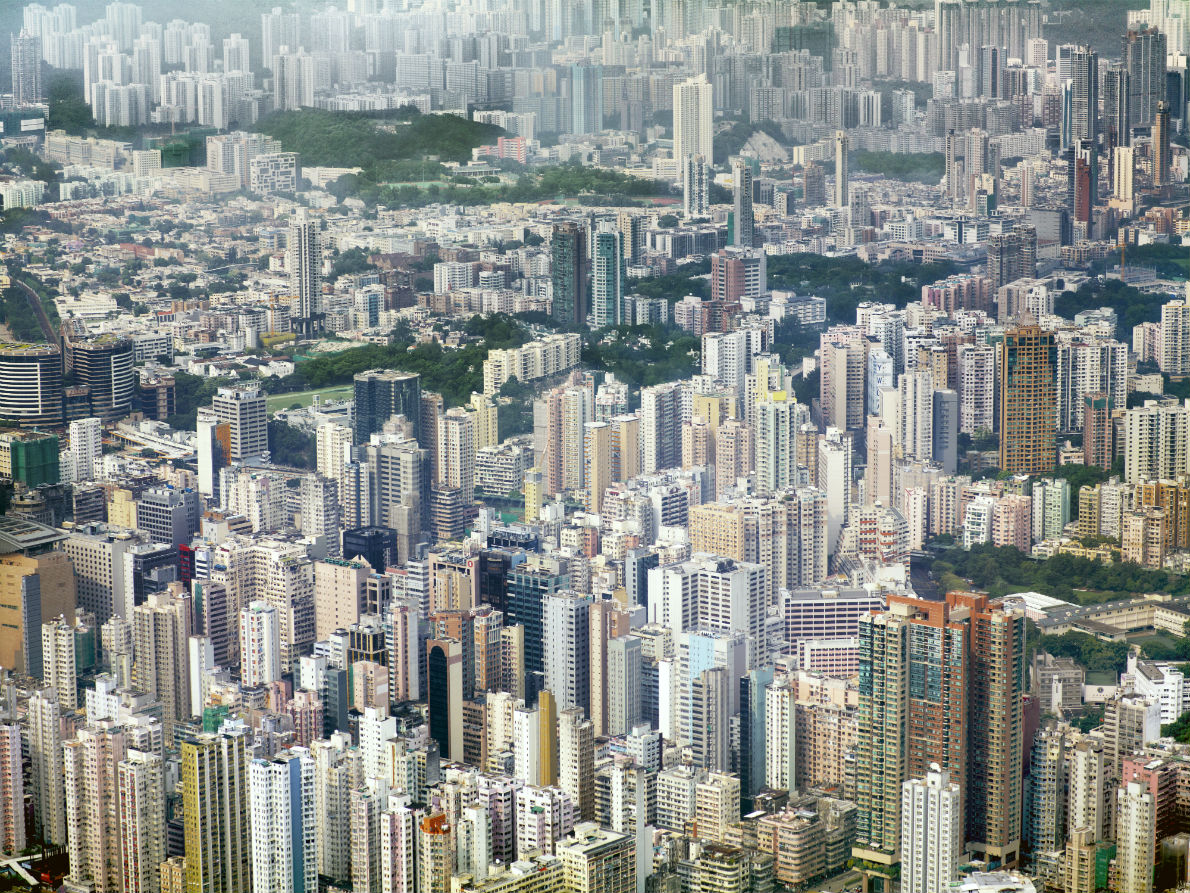

Supplement: Supplementary file 2 [file Data_Sheet_1.zip › Raw Images for Experiment 1/Cityscapes/city13.jpg]

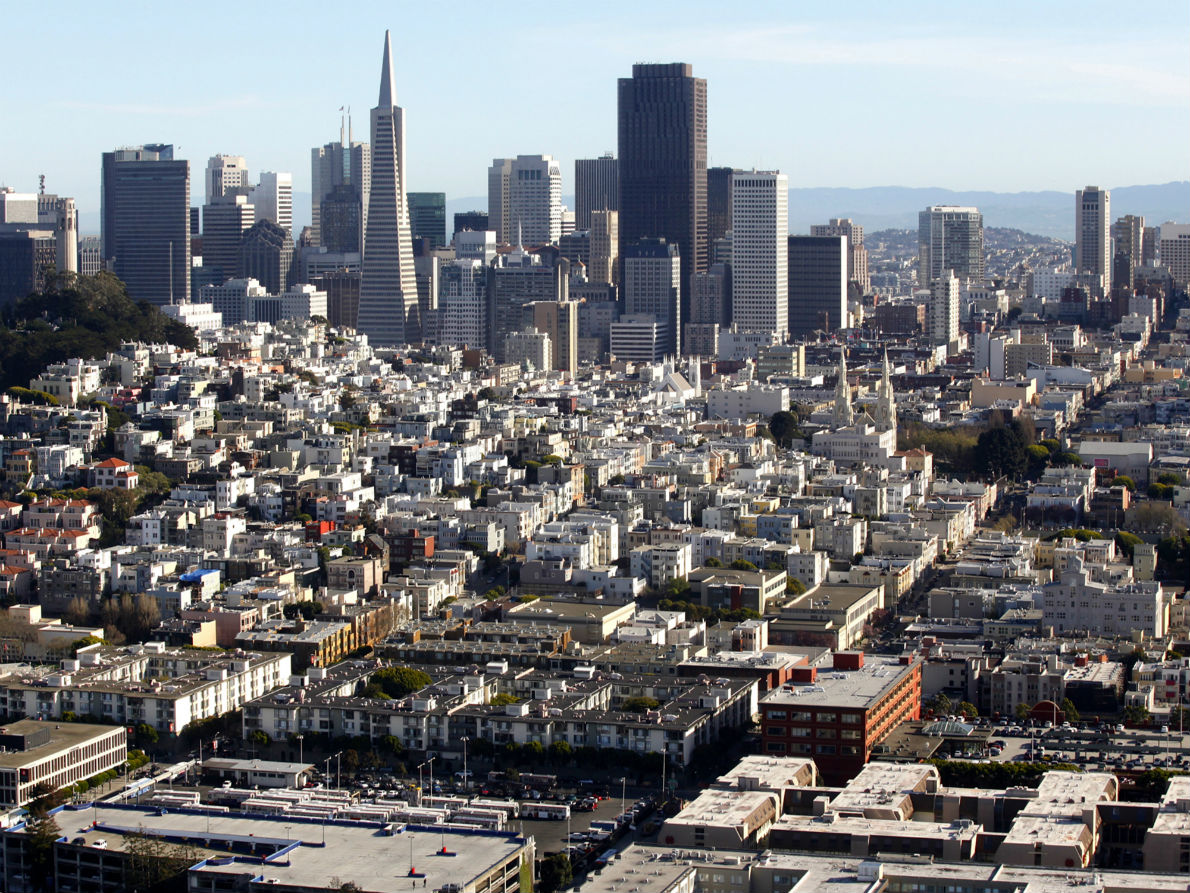

Supplement: Supplementary file 2 [file Data_Sheet_1.zip › Raw Images for Experiment 1/Cityscapes/city15.jpg]

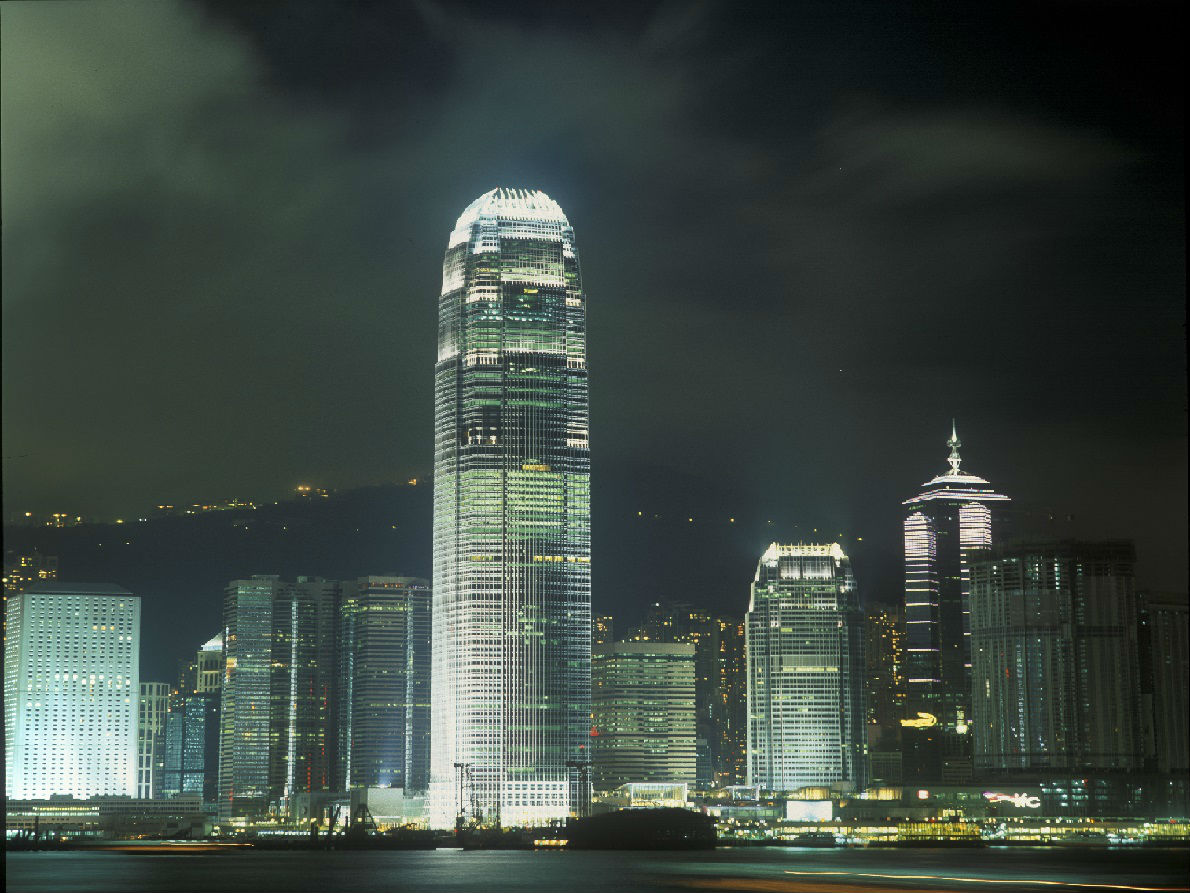

Supplement: Supplementary file 2 [file Data_Sheet_1.zip › Raw Images for Experiment 1/Cityscapes/city16.jpg]

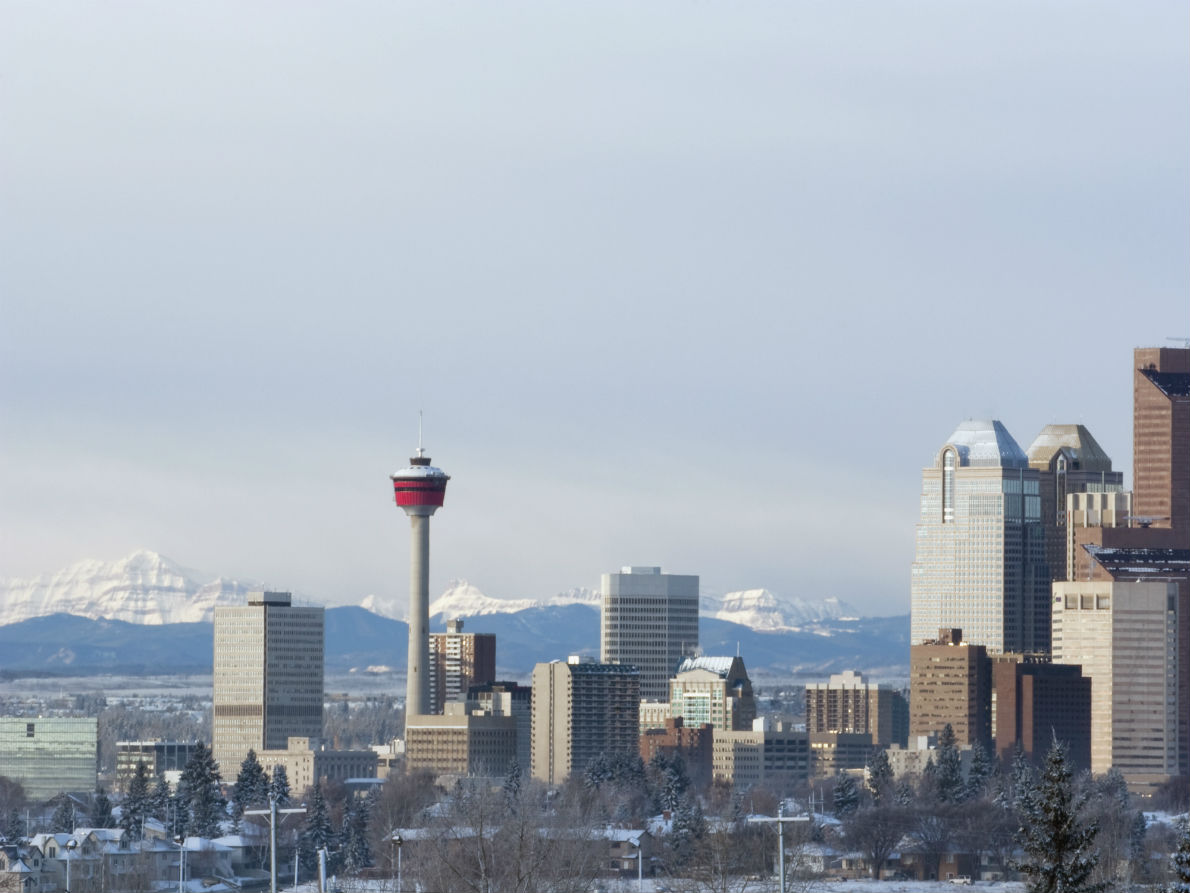

Supplement: Supplementary file 2 [file Data_Sheet_1.zip › Raw Images for Experiment 1/Cityscapes/city17.jpg]

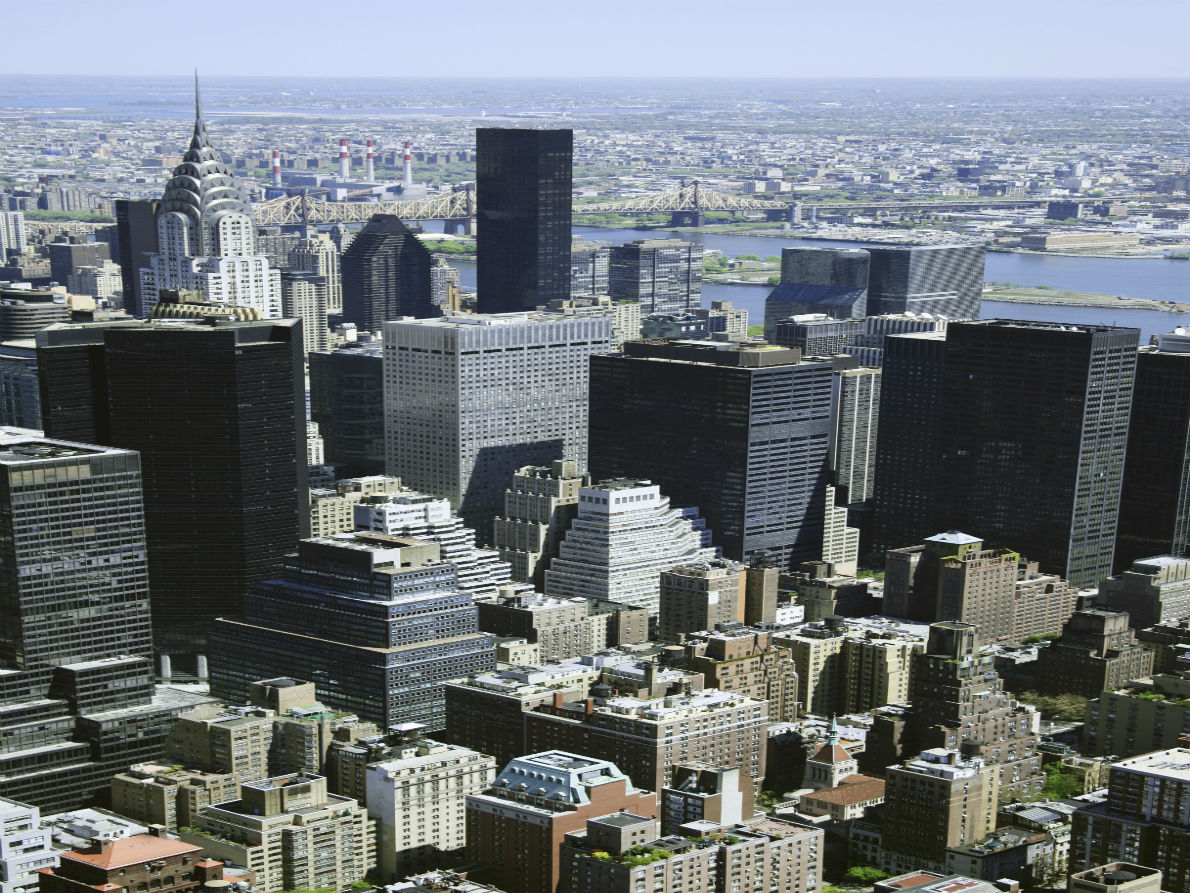

Supplement: Supplementary file 2 [file Data_Sheet_1.zip › Raw Images for Experiment 1/Cityscapes/city20.jpg]

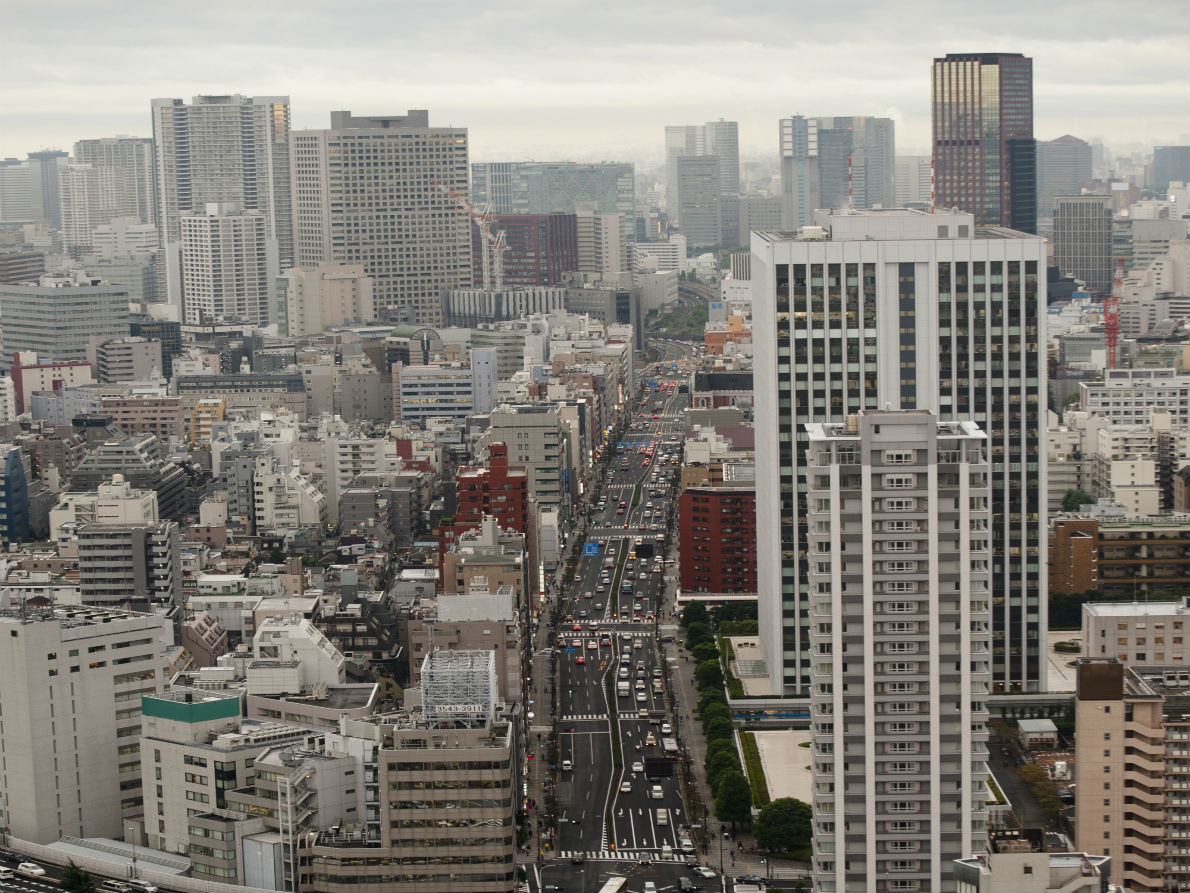

Supplement: Supplementary file 2 [file Data_Sheet_1.zip › Raw Images for Experiment 1/Cityscapes/city21.jpg]

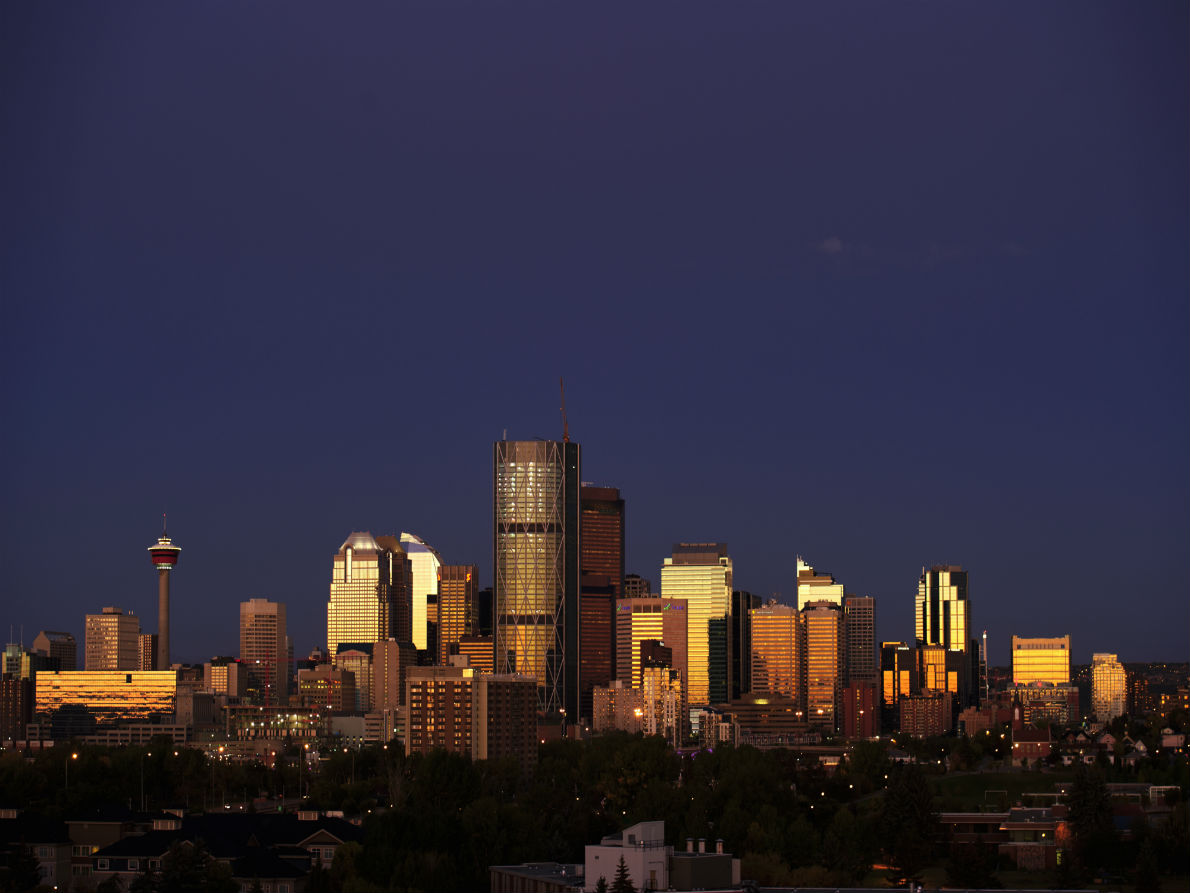

Supplement: Supplementary file 2 [file Data_Sheet_1.zip › Raw Images for Experiment 1/Cityscapes/city28.jpg]

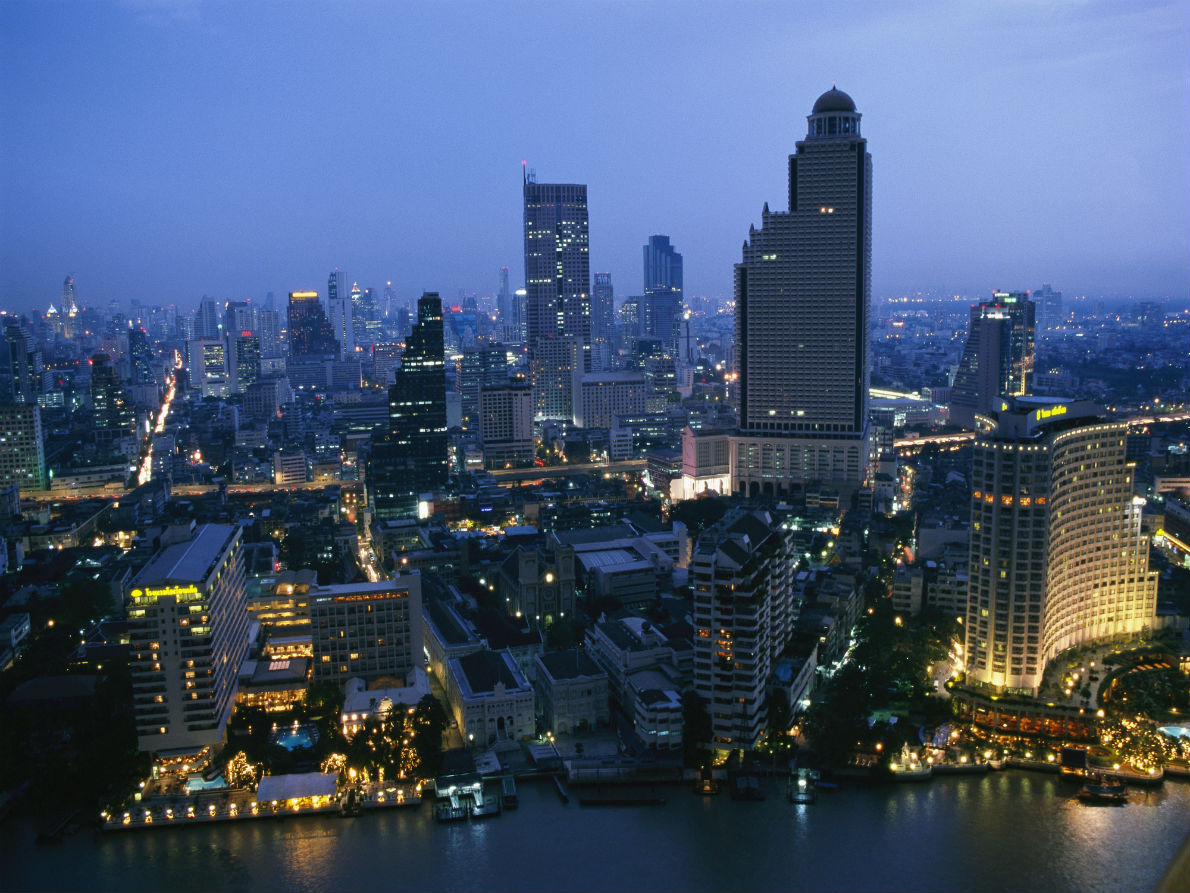

Supplement: Supplementary file 2 [file Data_Sheet_1.zip › Raw Images for Experiment 1/Cityscapes/city3.jpg]

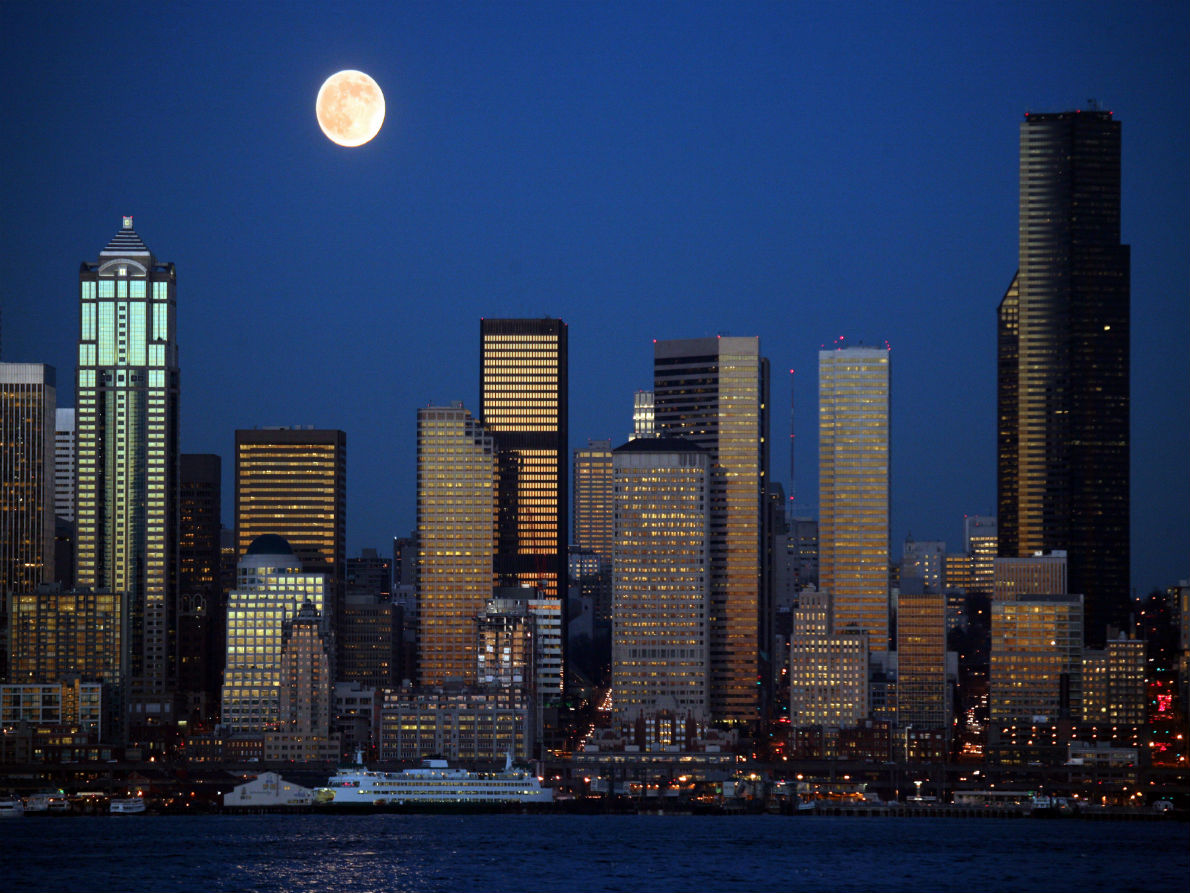

Supplement: Supplementary file 2 [file Data_Sheet_1.zip › Raw Images for Experiment 1/Cityscapes/city31.jpg]

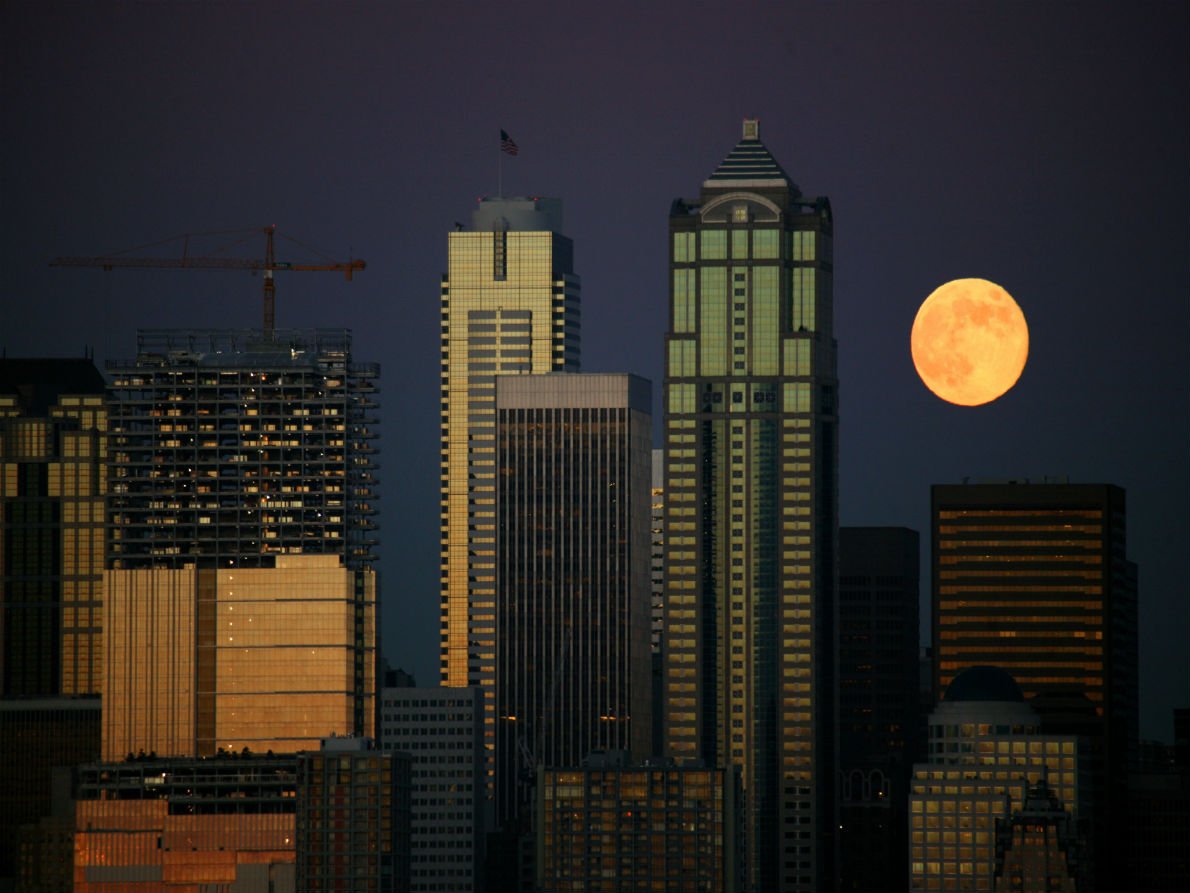

Supplement: Supplementary file 2 [file Data_Sheet_1.zip › Raw Images for Experiment 1/Cityscapes/city32.jpg]

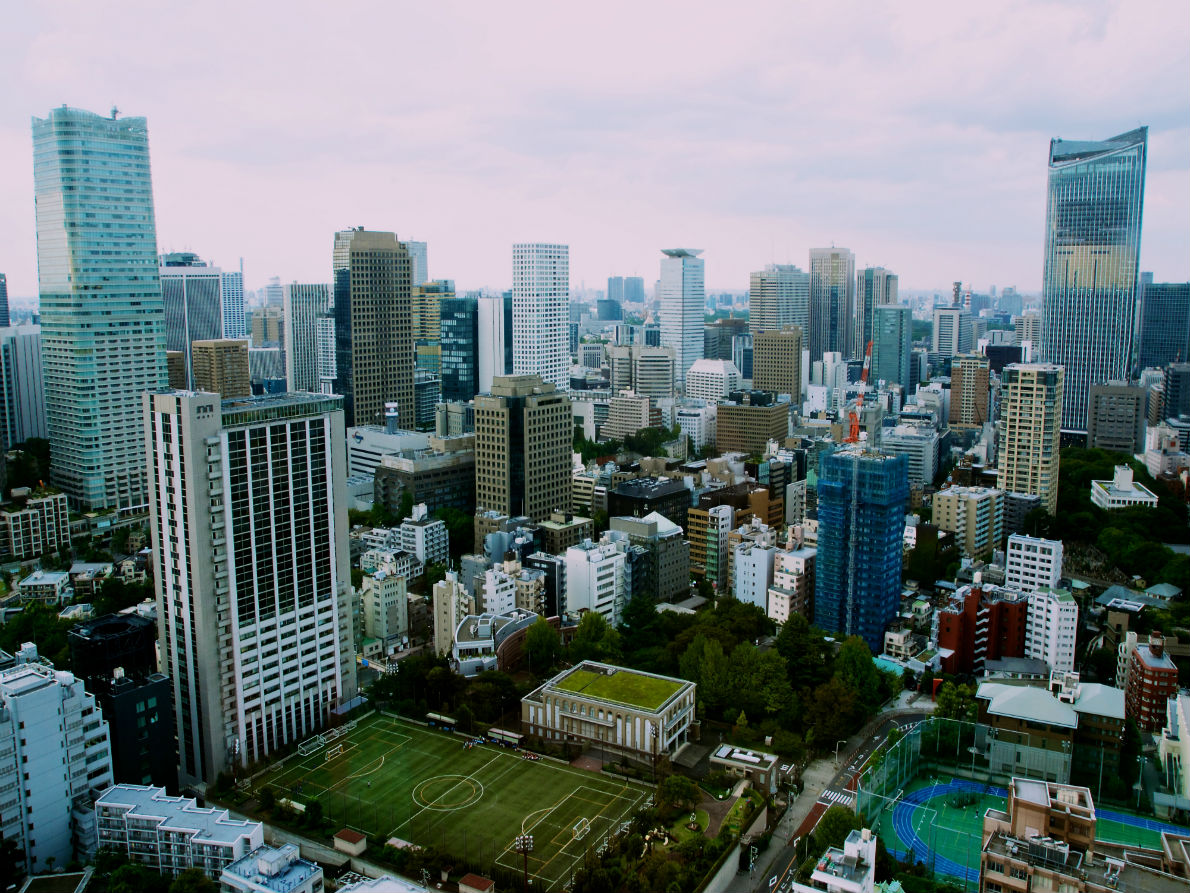

Supplement: Supplementary file 2 [file Data_Sheet_1.zip › Raw Images for Experiment 1/Cityscapes/city33.jpg]

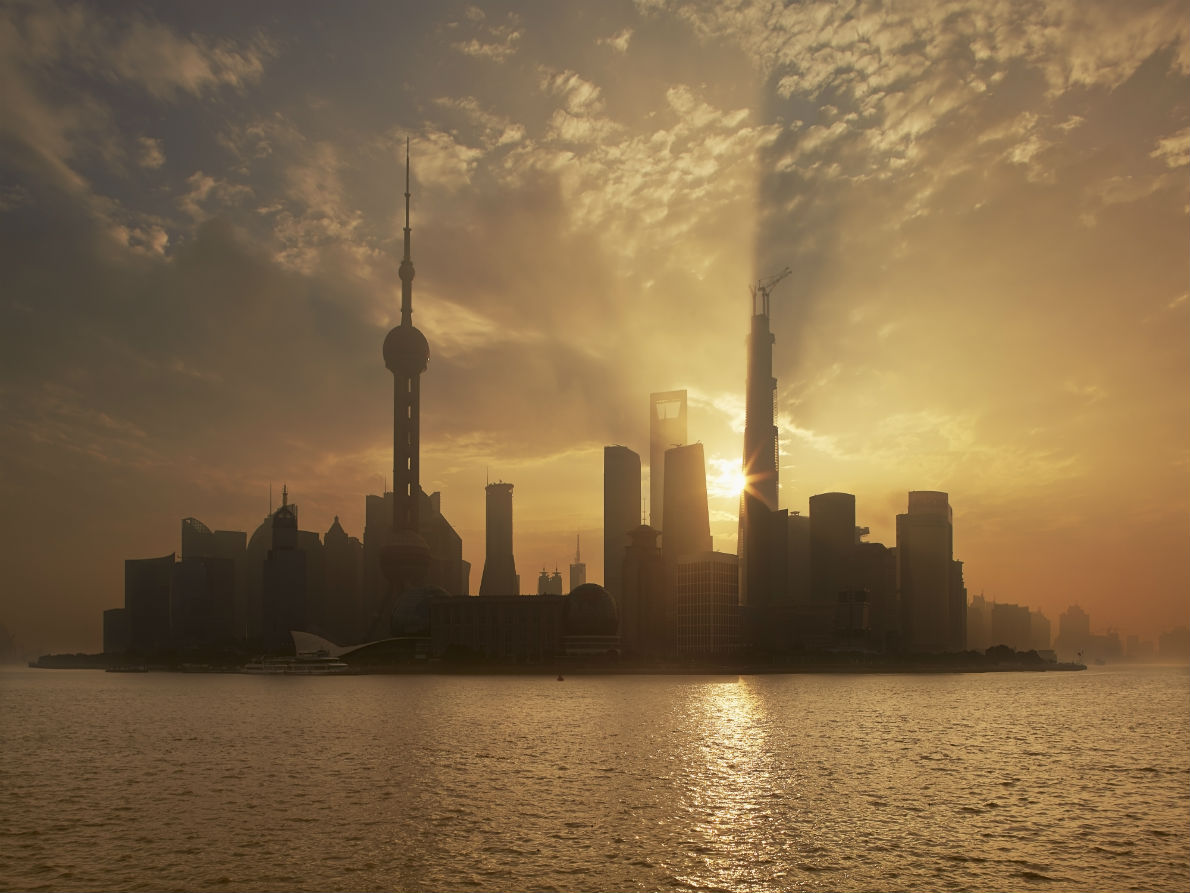

Supplement: Supplementary file 2 [file Data_Sheet_1.zip › Raw Images for Experiment 1/Cityscapes/city35.jpg]

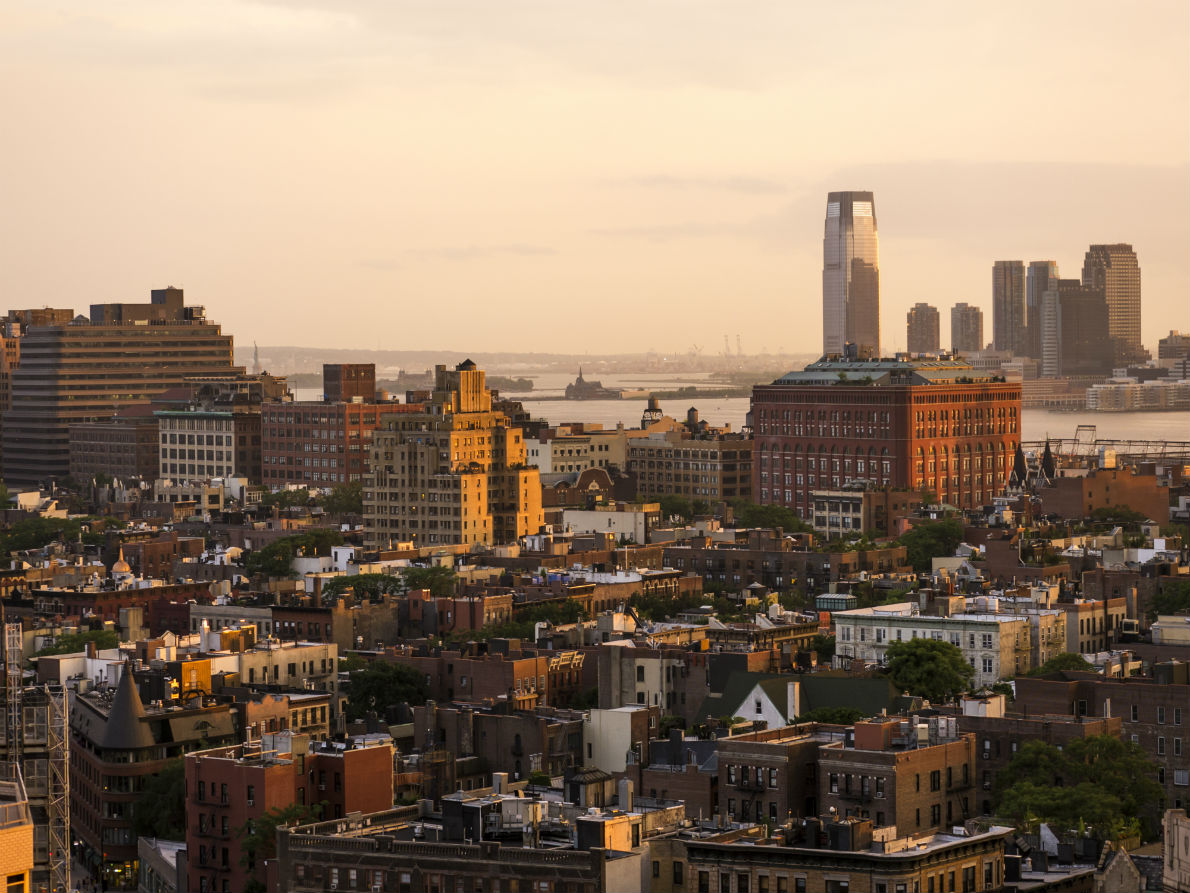

Supplement: Supplementary file 2 [file Data_Sheet_1.zip › Raw Images for Experiment 1/Cityscapes/city37.jpg]

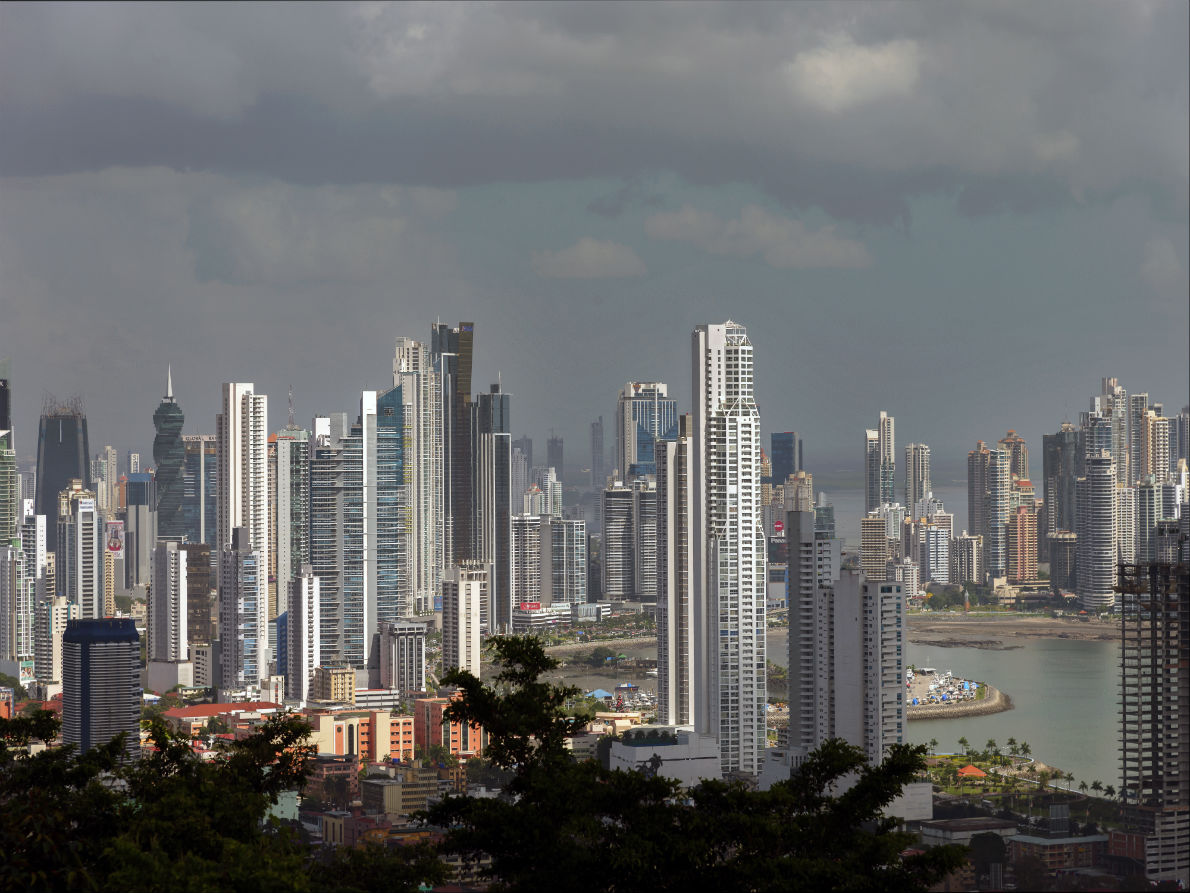

Supplement: Supplementary file 2 [file Data_Sheet_1.zip › Raw Images for Experiment 1/Cityscapes/city38.jpg]

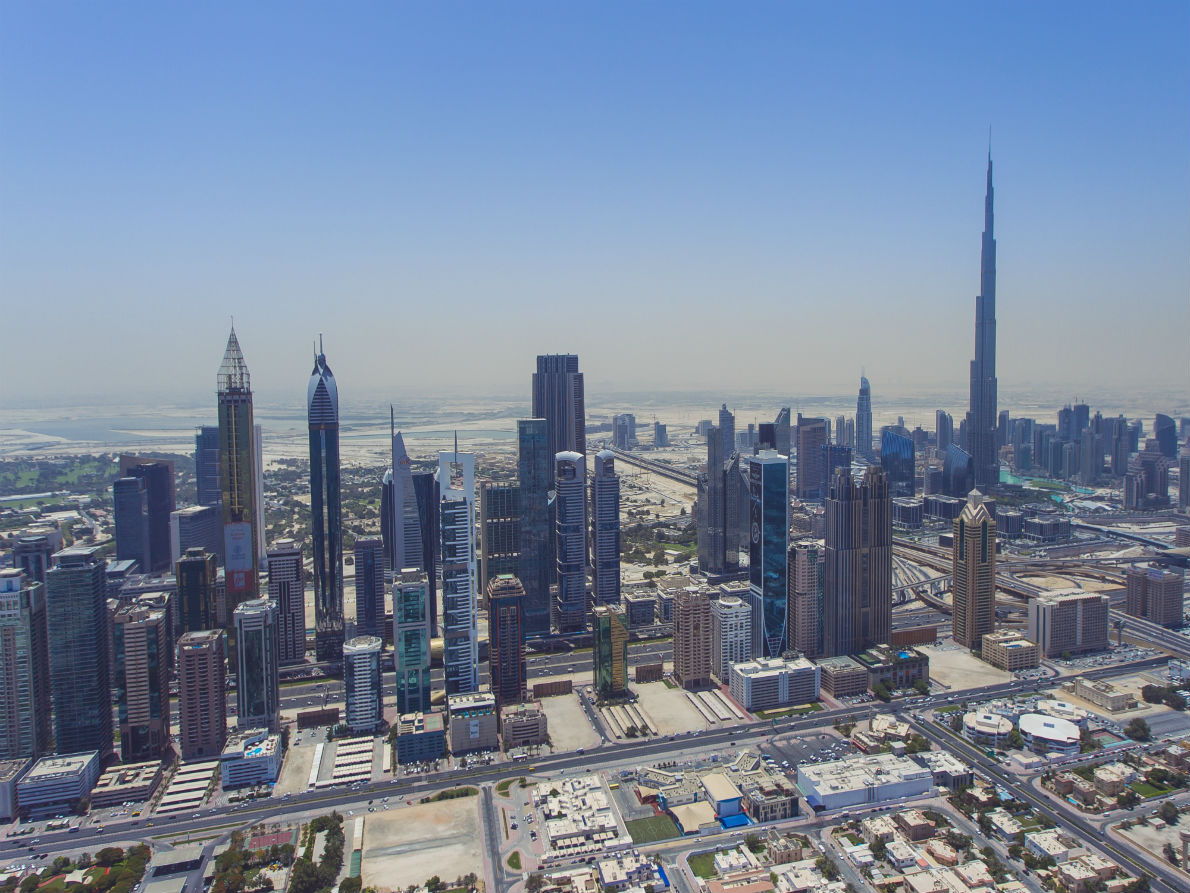

Supplement: Supplementary file 2 [file Data_Sheet_1.zip › Raw Images for Experiment 1/Cityscapes/city44.jpg]

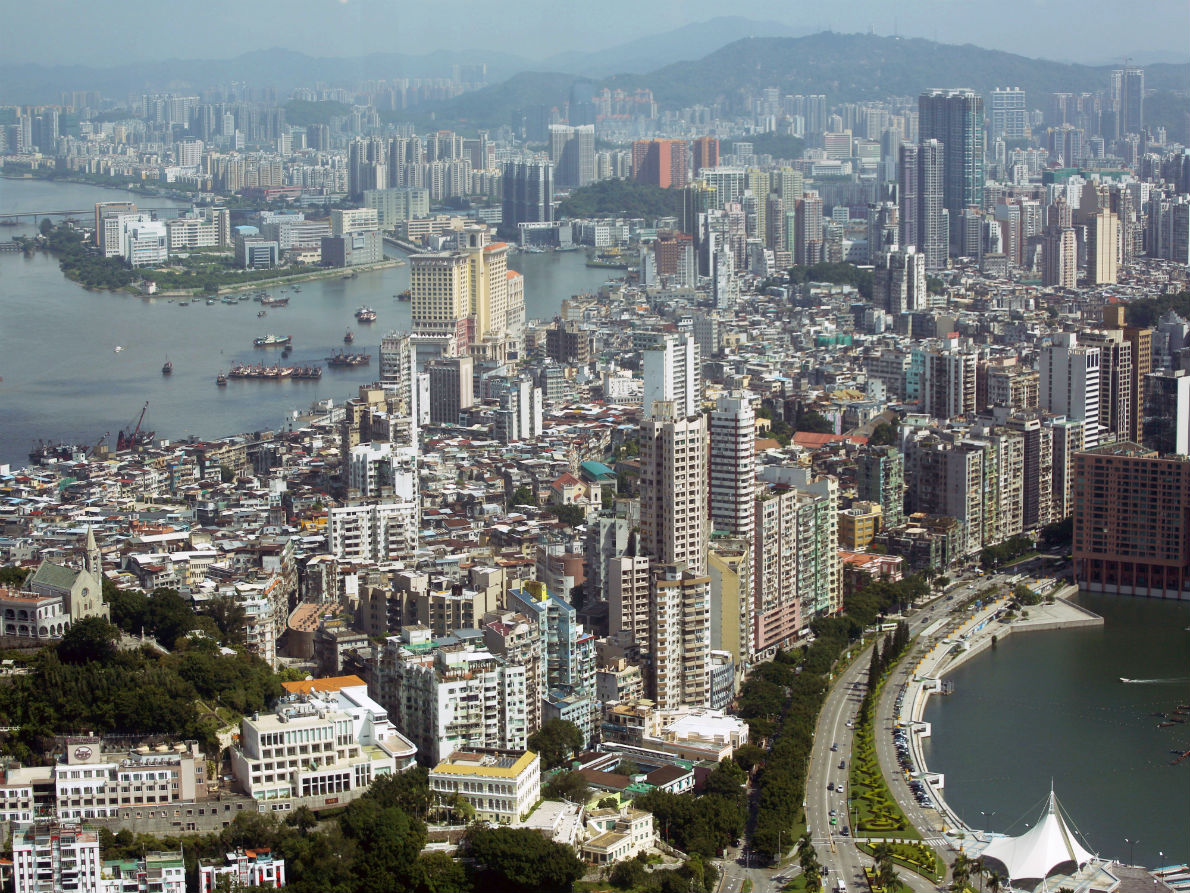

Supplement: Supplementary file 2 [file Data_Sheet_1.zip › Raw Images for Experiment 1/Cityscapes/city47.jpg]

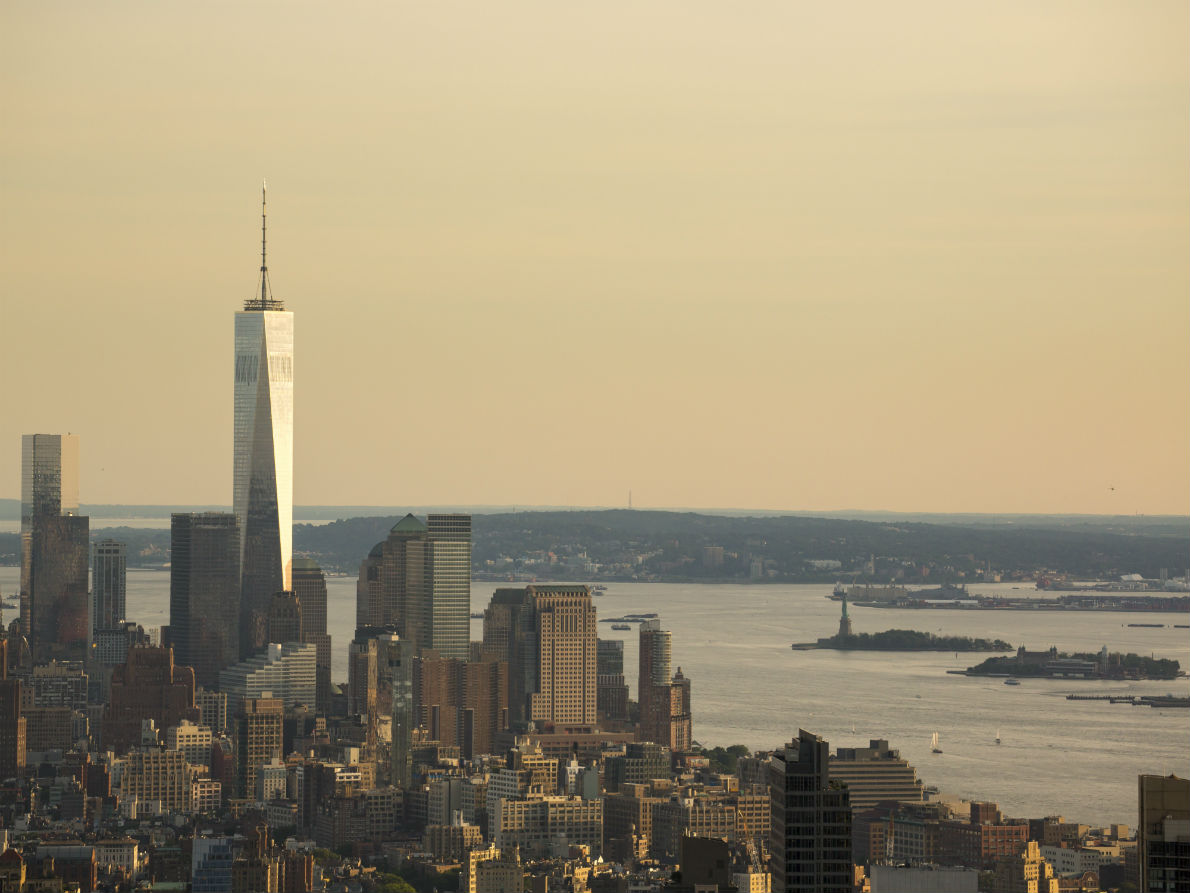

Supplement: Supplementary file 2 [file Data_Sheet_1.zip › Raw Images for Experiment 1/Cityscapes/city48.jpg]

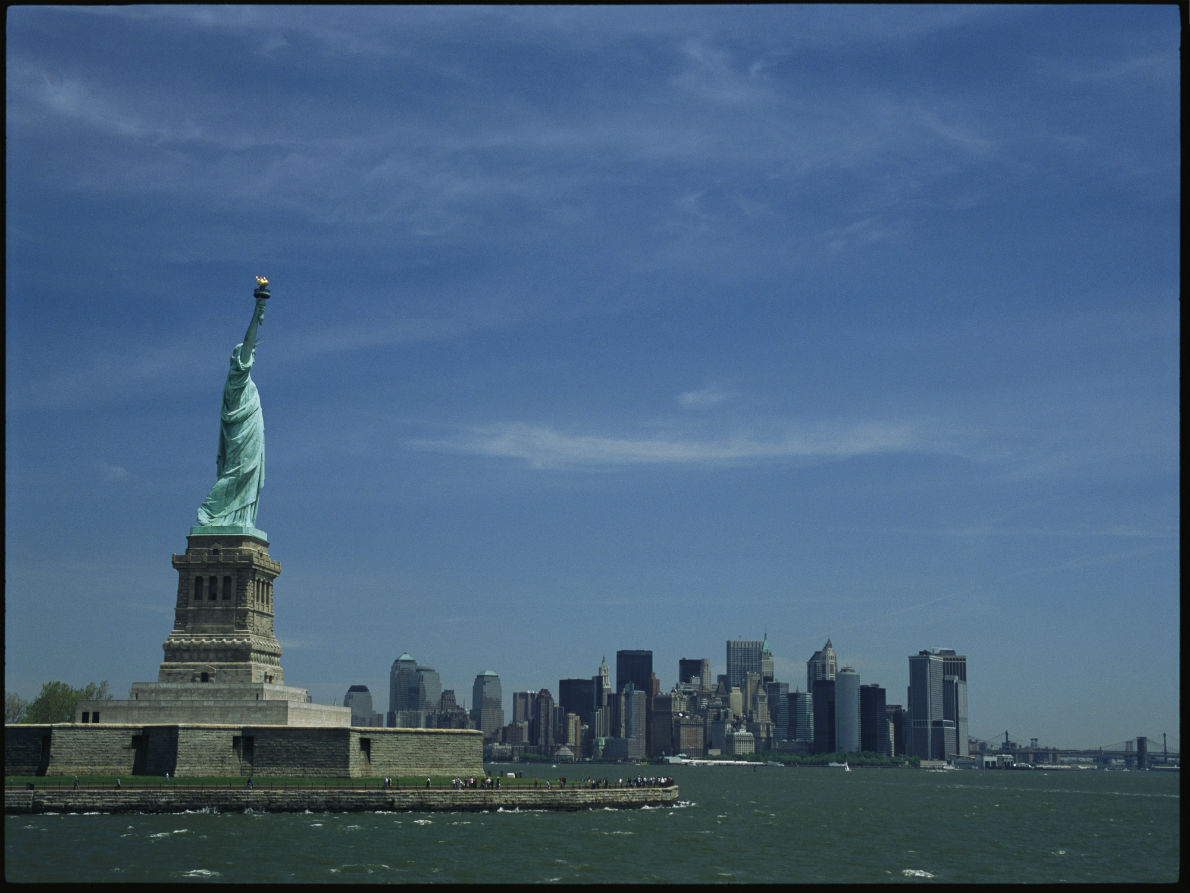

Supplement: Supplementary file 2 [file Data_Sheet_1.zip › Raw Images for Experiment 1/Cityscapes/city5.jpg]

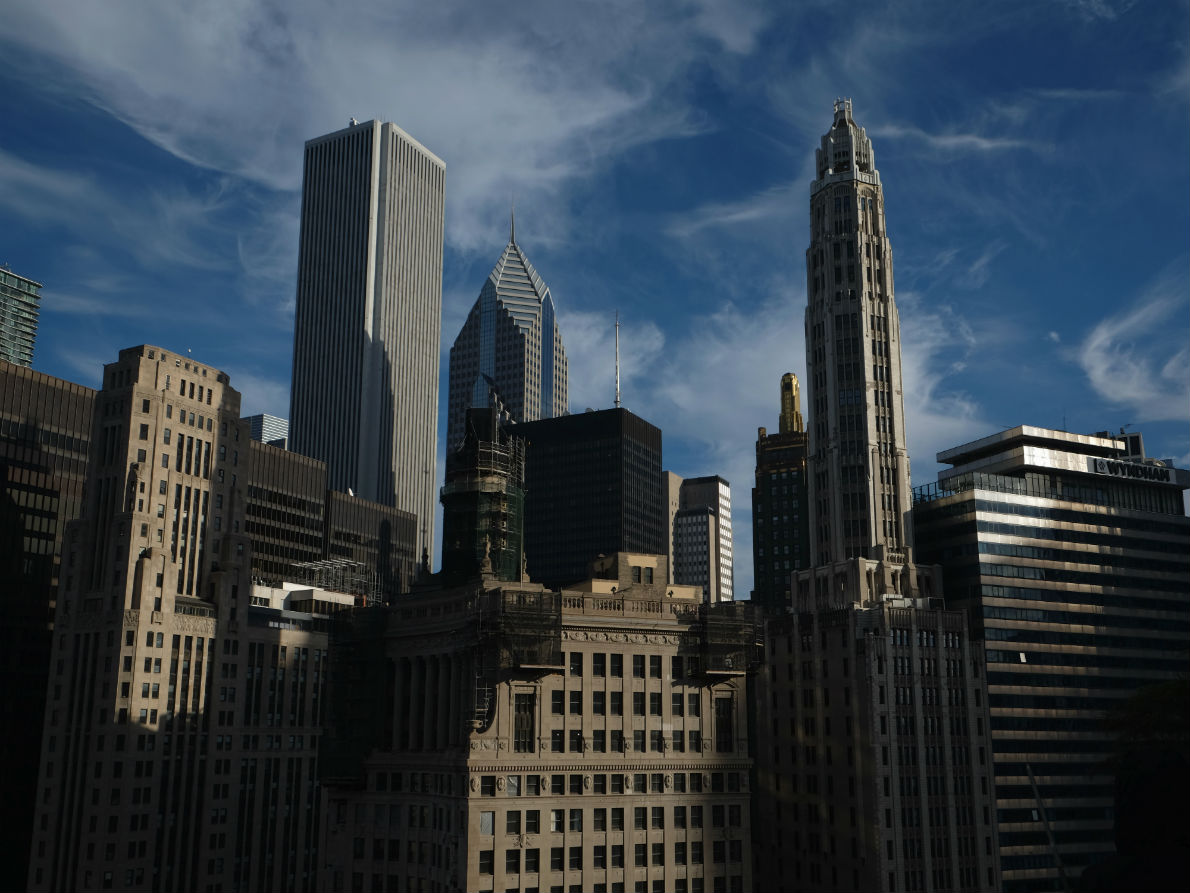

Supplement: Supplementary file 2 [file Data_Sheet_1.zip › Raw Images for Experiment 1/Cityscapes/city50.jpg]

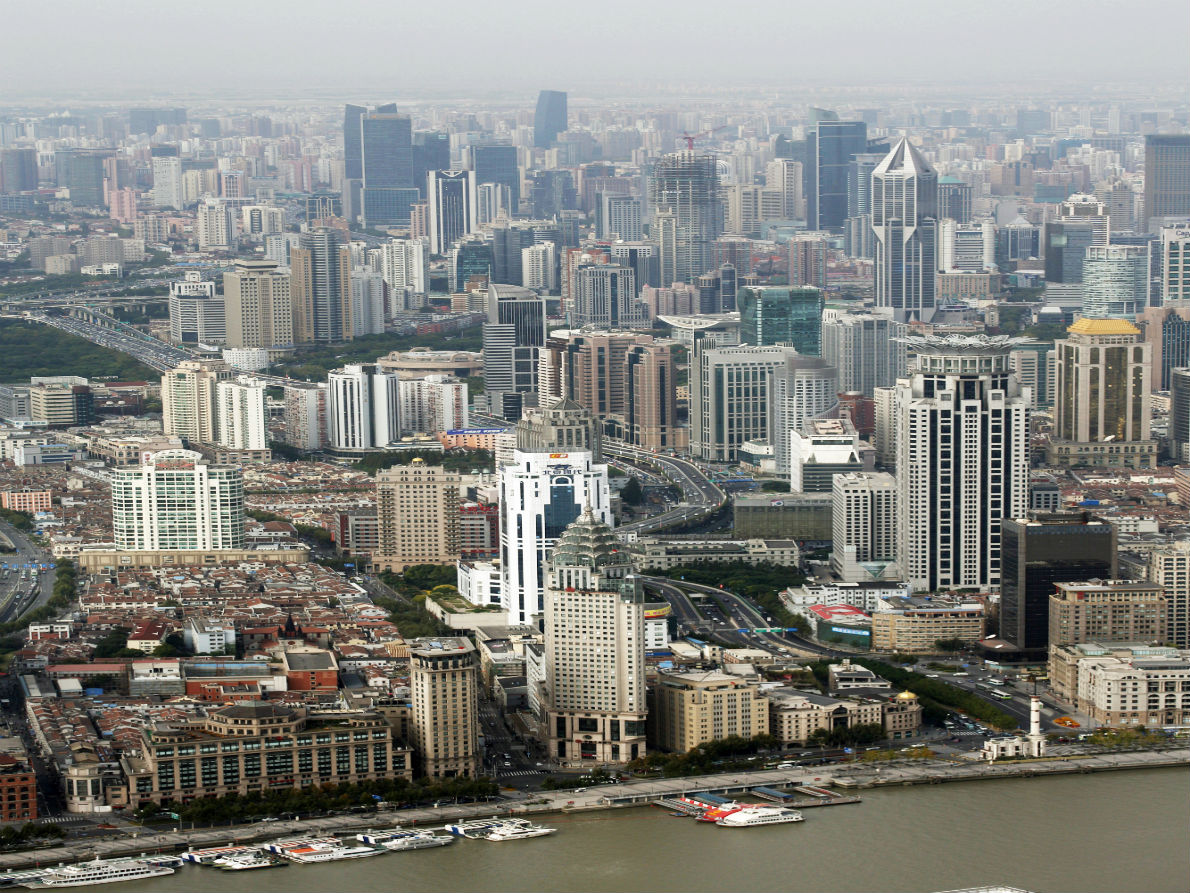

Supplement: Supplementary file 2 [file Data_Sheet_1.zip › Raw Images for Experiment 1/Cityscapes/city52.jpg]

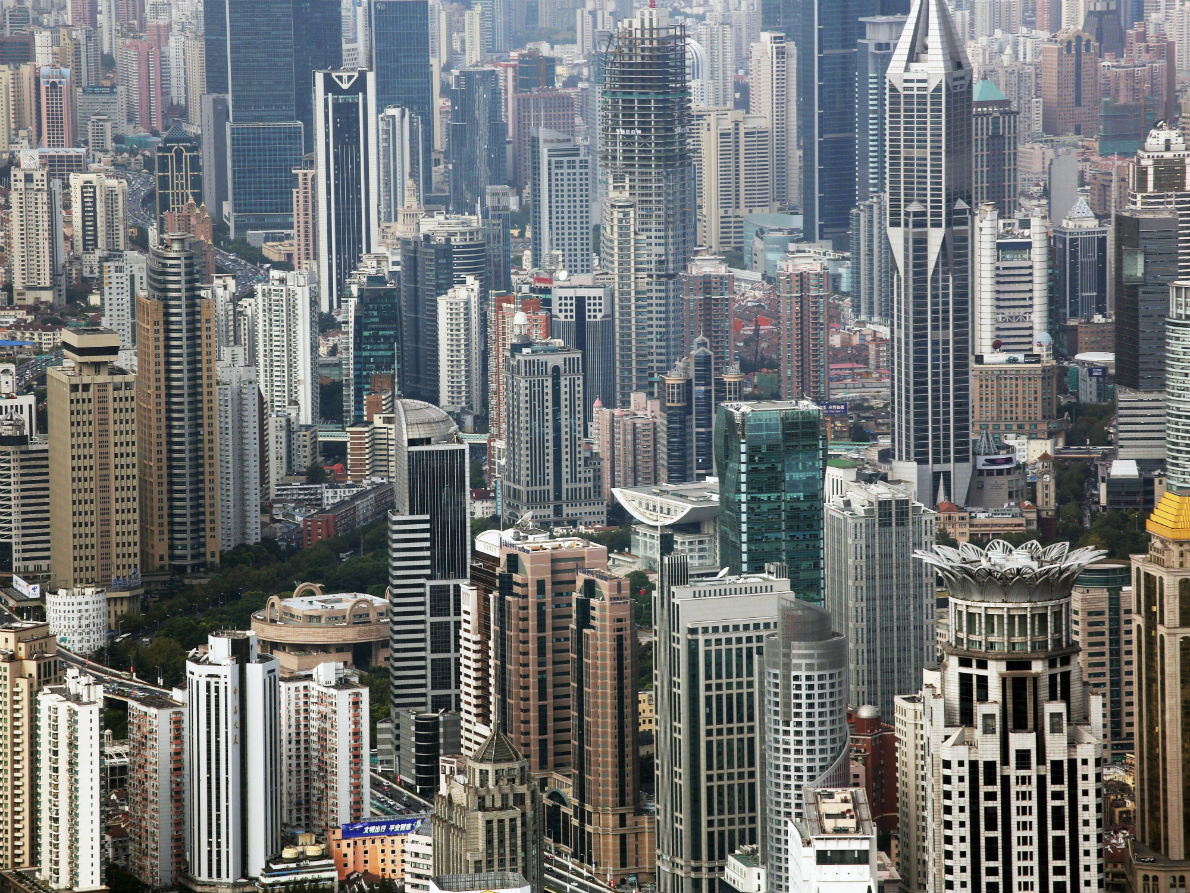

Supplement: Supplementary file 2 [file Data_Sheet_1.zip › Raw Images for Experiment 1/Cityscapes/city53.jpg]

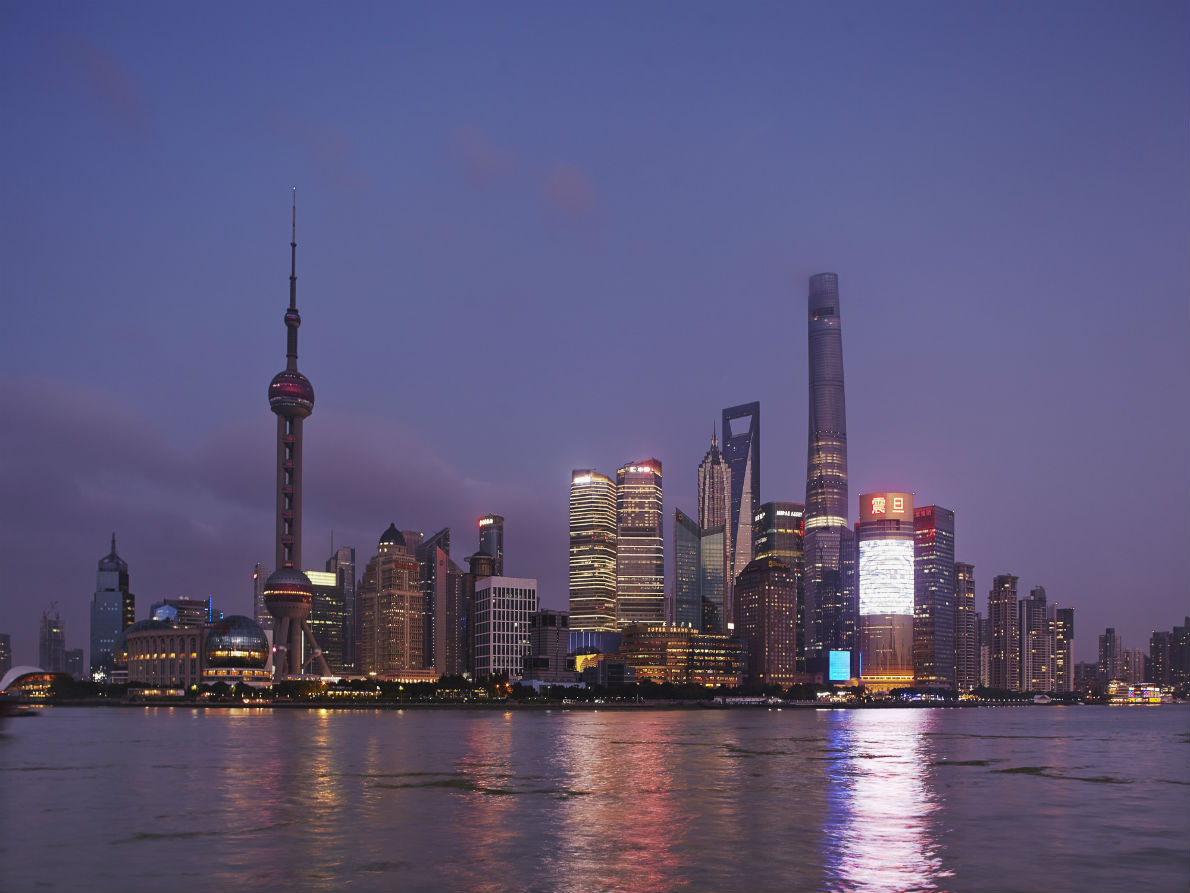

Supplement: Supplementary file 2 [file Data_Sheet_1.zip › Raw Images for Experiment 1/Cityscapes/city56.jpg]

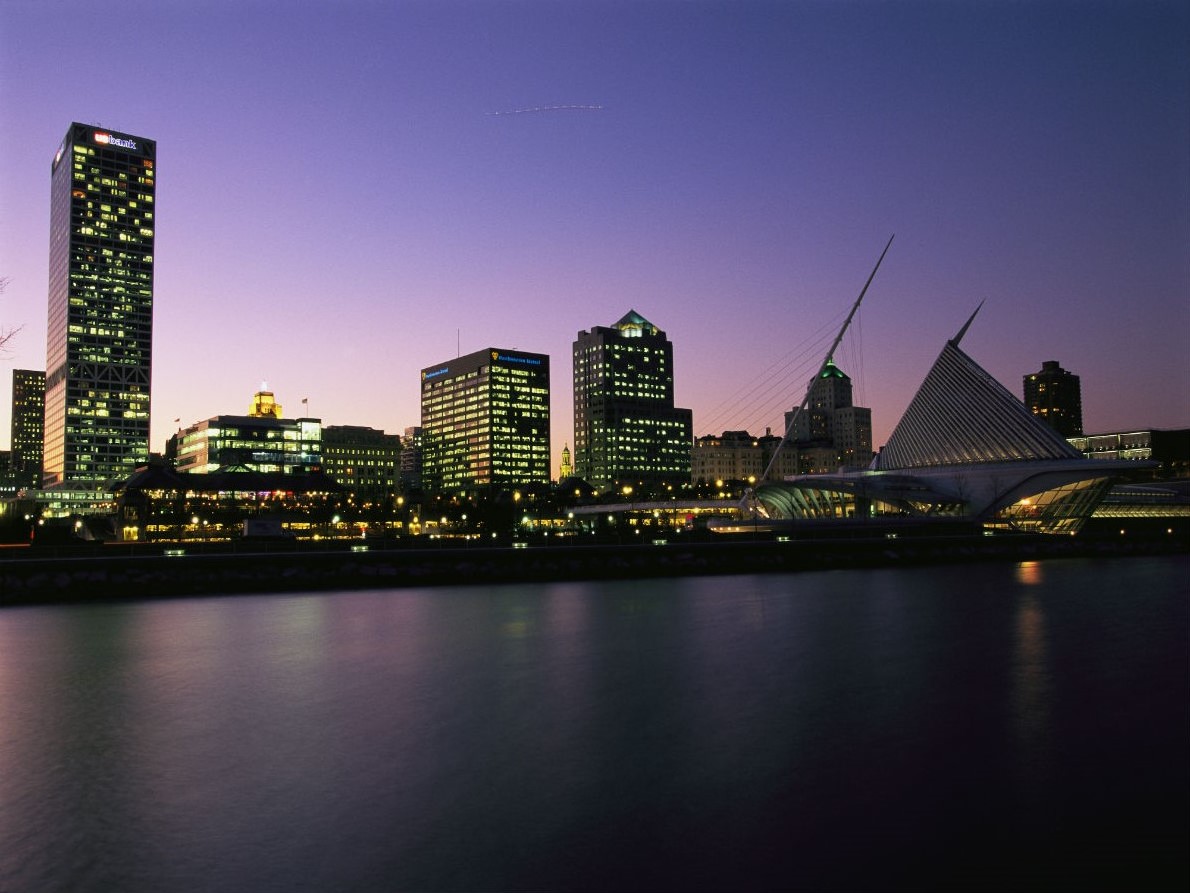

Supplement: Supplementary file 2 [file Data_Sheet_1.zip › Raw Images for Experiment 1/Cityscapes/city7.jpg]

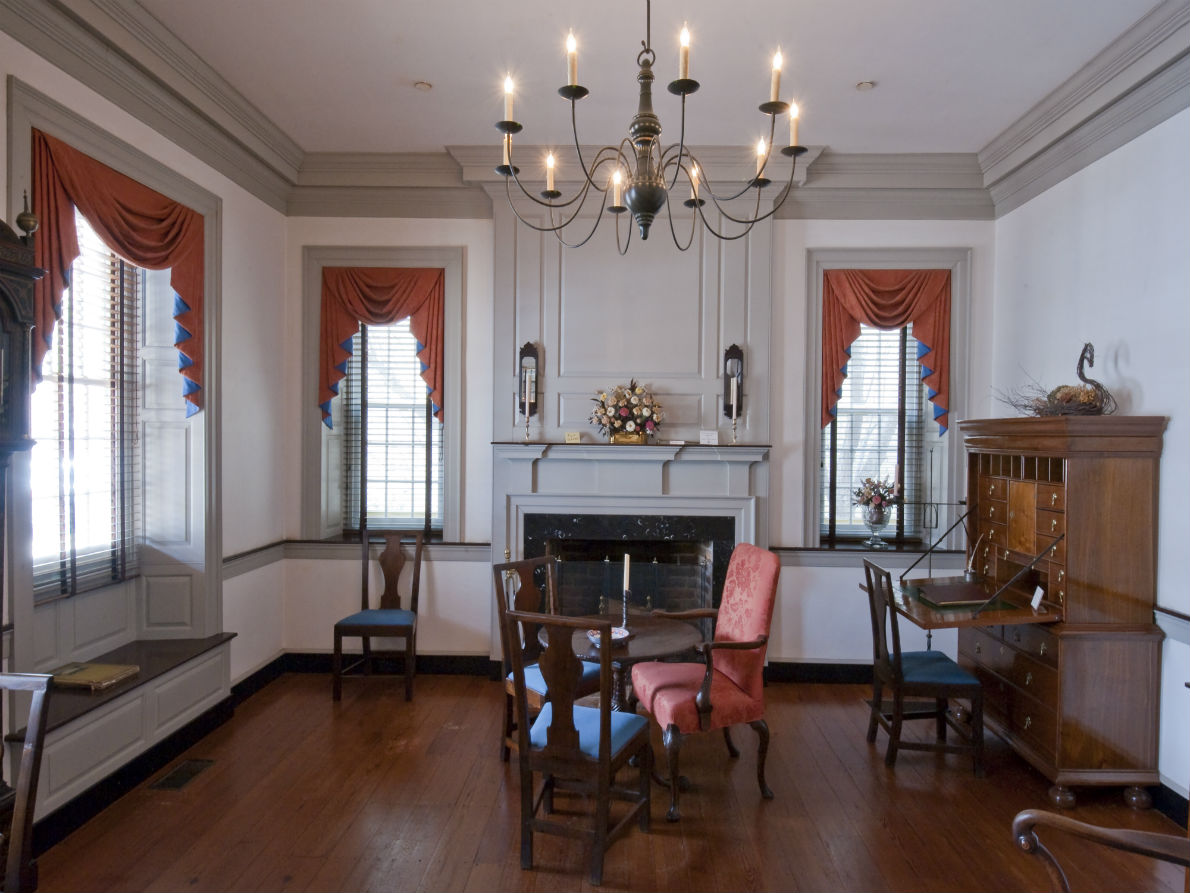

Supplement: Supplementary file 2 [file Data_Sheet_1.zip › Raw Images for Experiment 1/Indoors/id10.jpg]

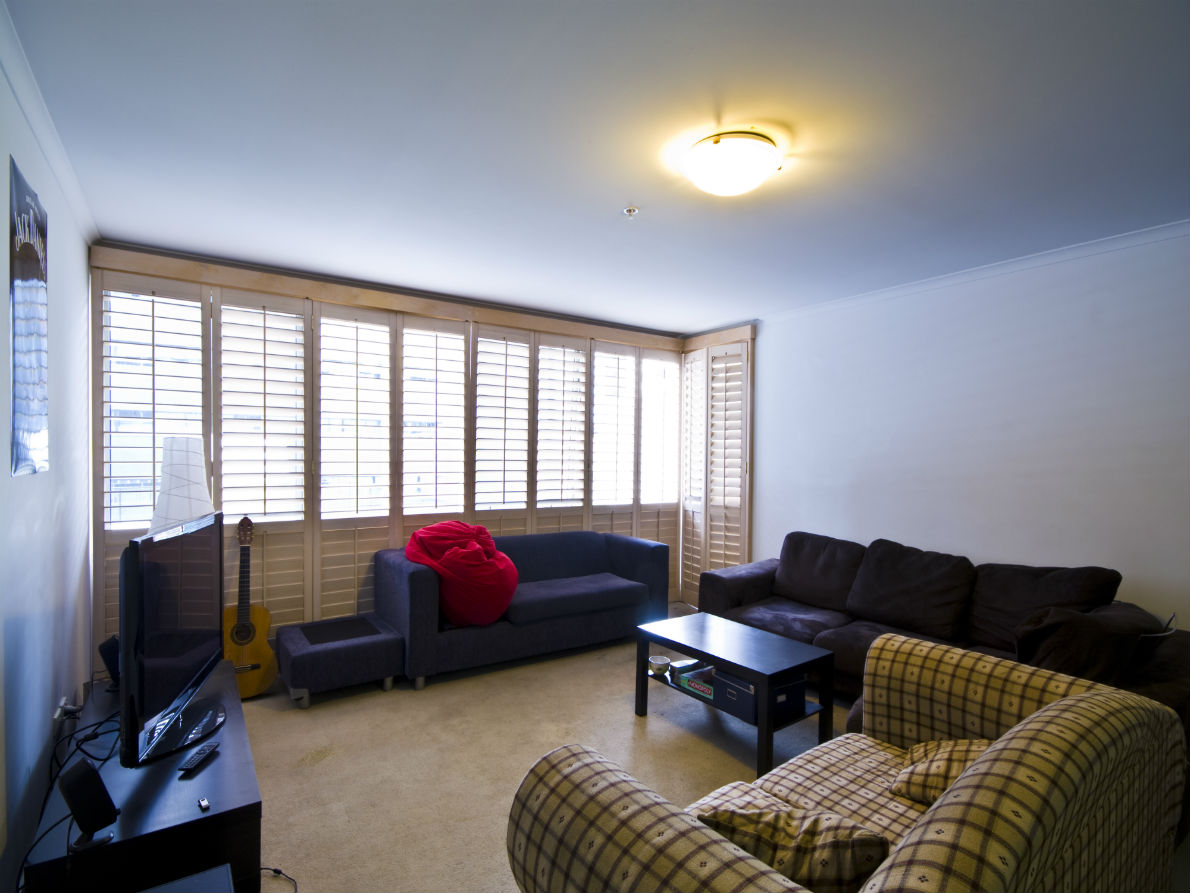

Supplement: Supplementary file 2 [file Data_Sheet_1.zip › Raw Images for Experiment 1/Indoors/id11.jpg]

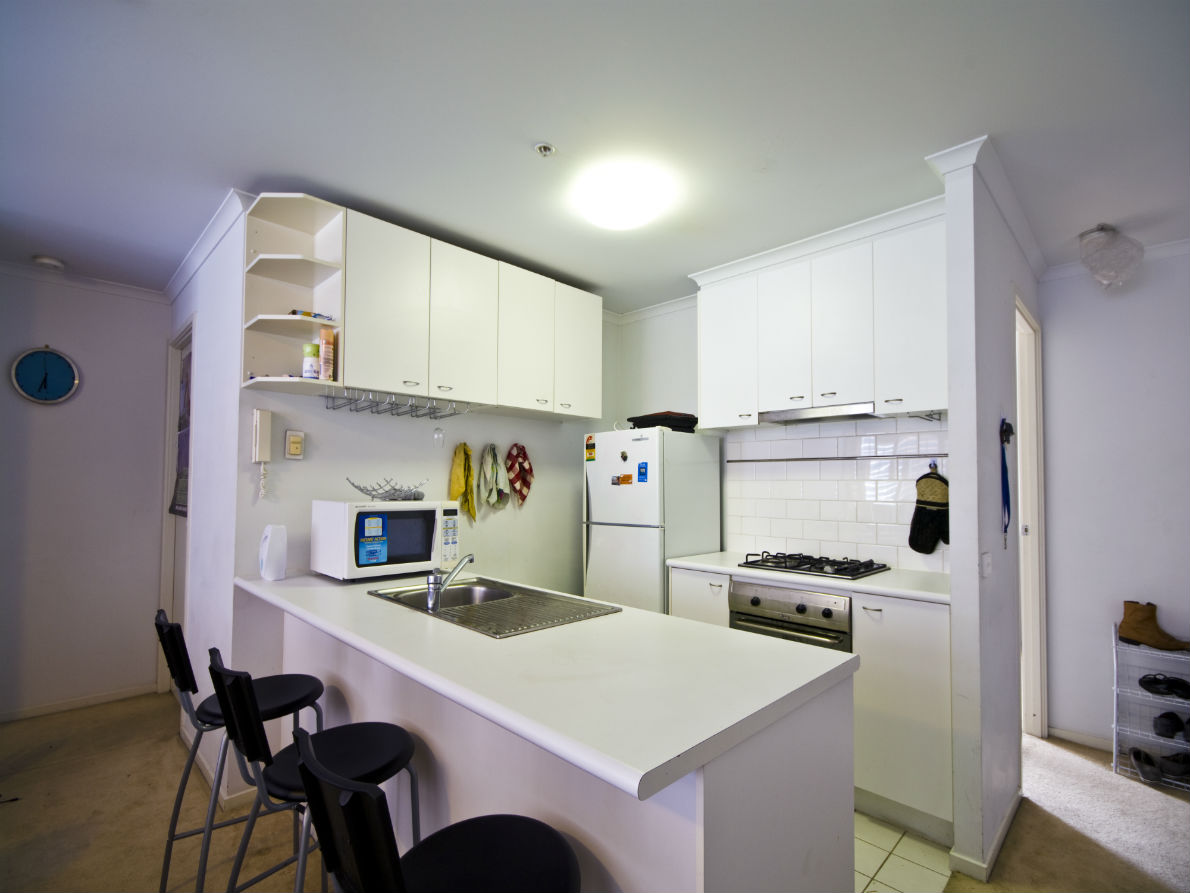

Supplement: Supplementary file 2 [file Data_Sheet_1.zip › Raw Images for Experiment 1/Indoors/id12.jpg]

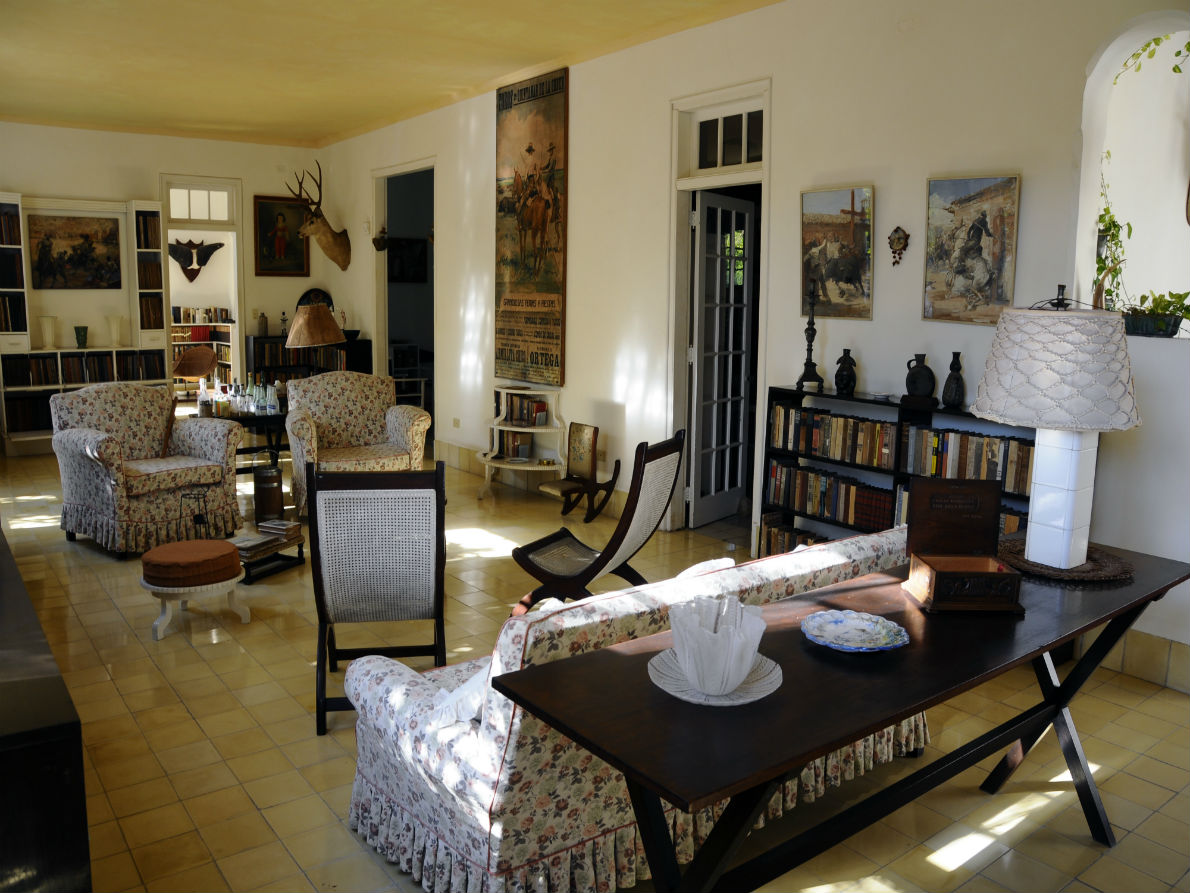

Supplement: Supplementary file 2 [file Data_Sheet_1.zip › Raw Images for Experiment 1/Indoors/id14.jpg]

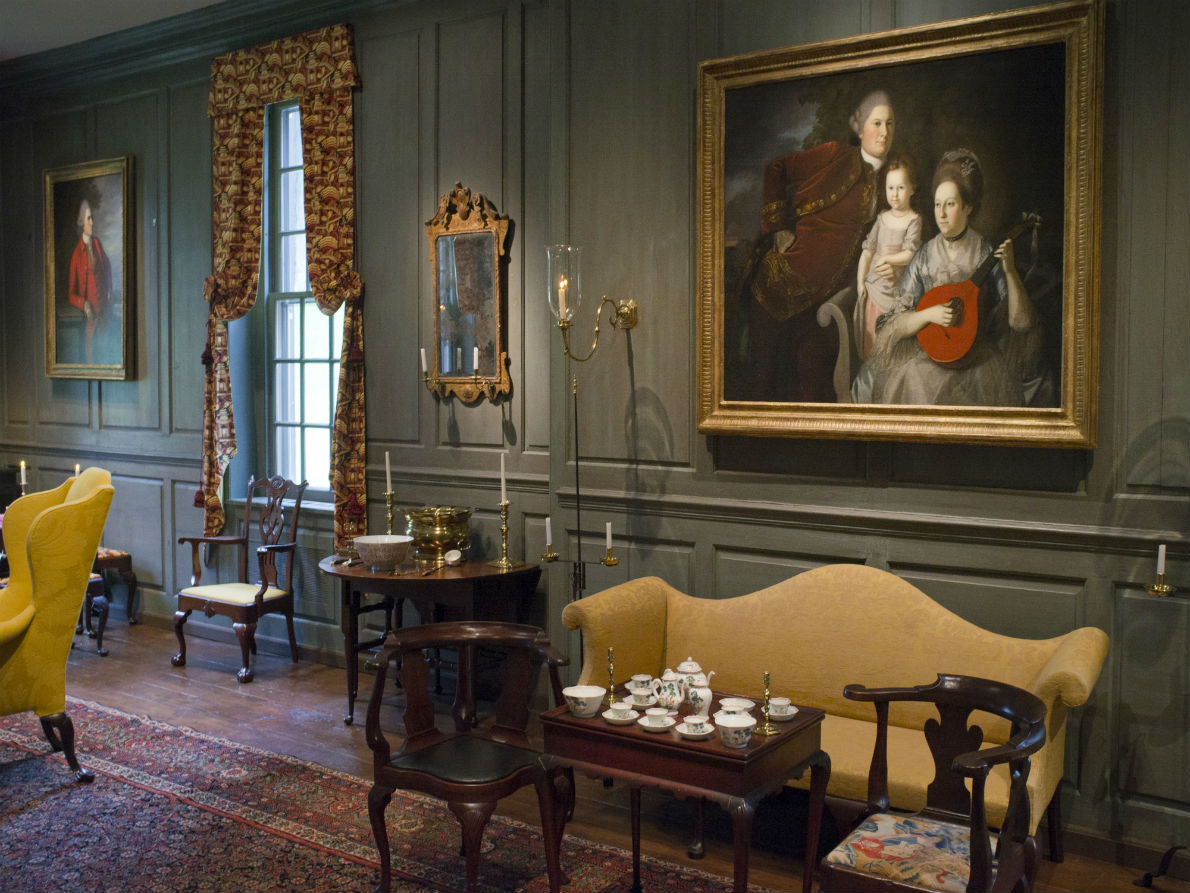

Supplement: Supplementary file 2 [file Data_Sheet_1.zip › Raw Images for Experiment 1/Indoors/id15.jpg]

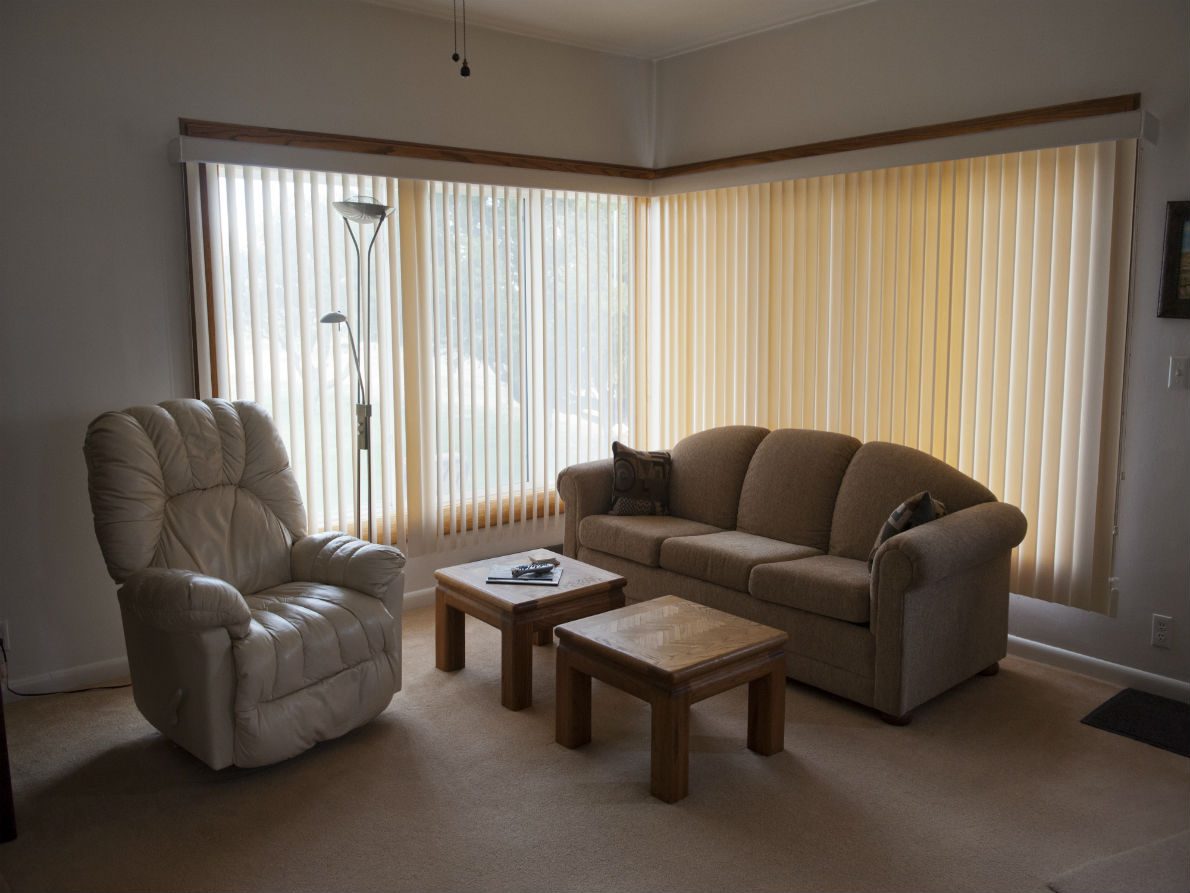

Supplement: Supplementary file 2 [file Data_Sheet_1.zip › Raw Images for Experiment 1/Indoors/id16.jpg]

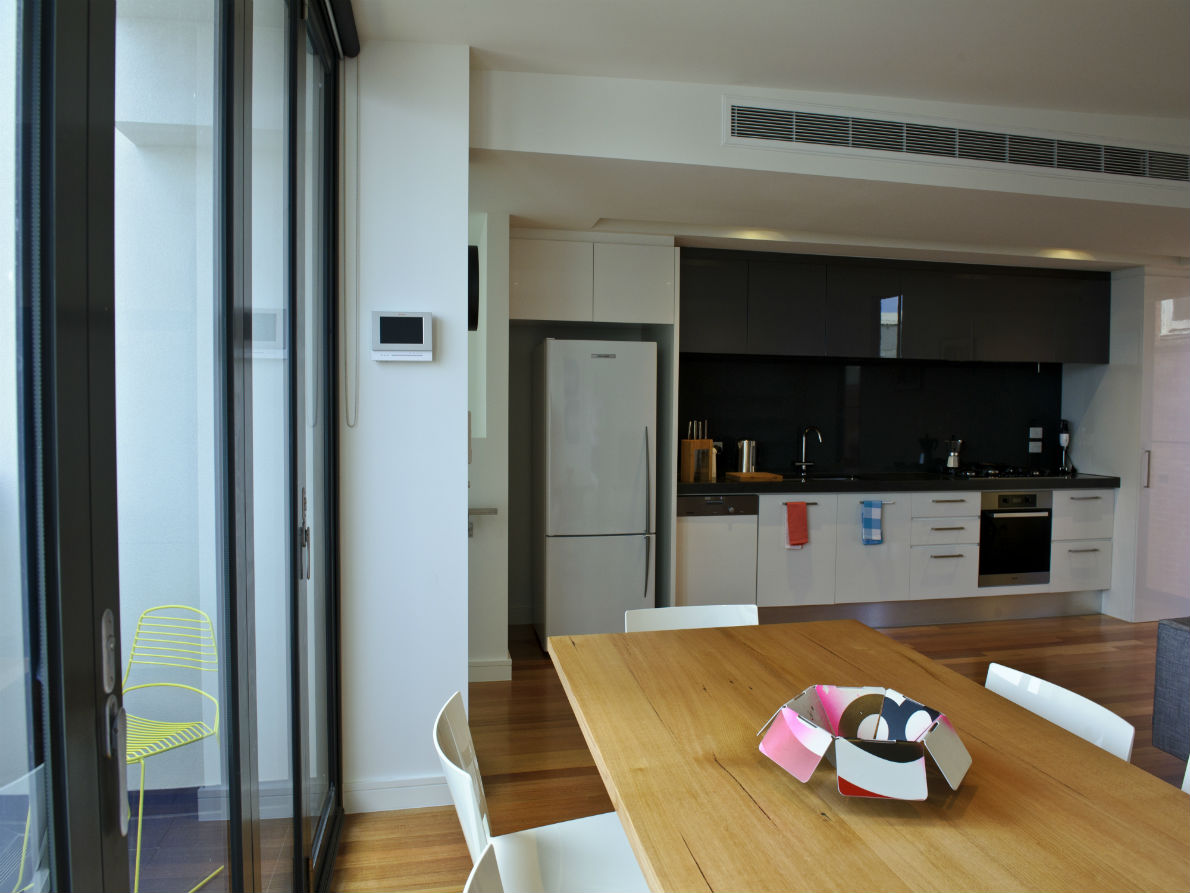

Supplement: Supplementary file 2 [file Data_Sheet_1.zip › Raw Images for Experiment 1/Indoors/id18.jpg]

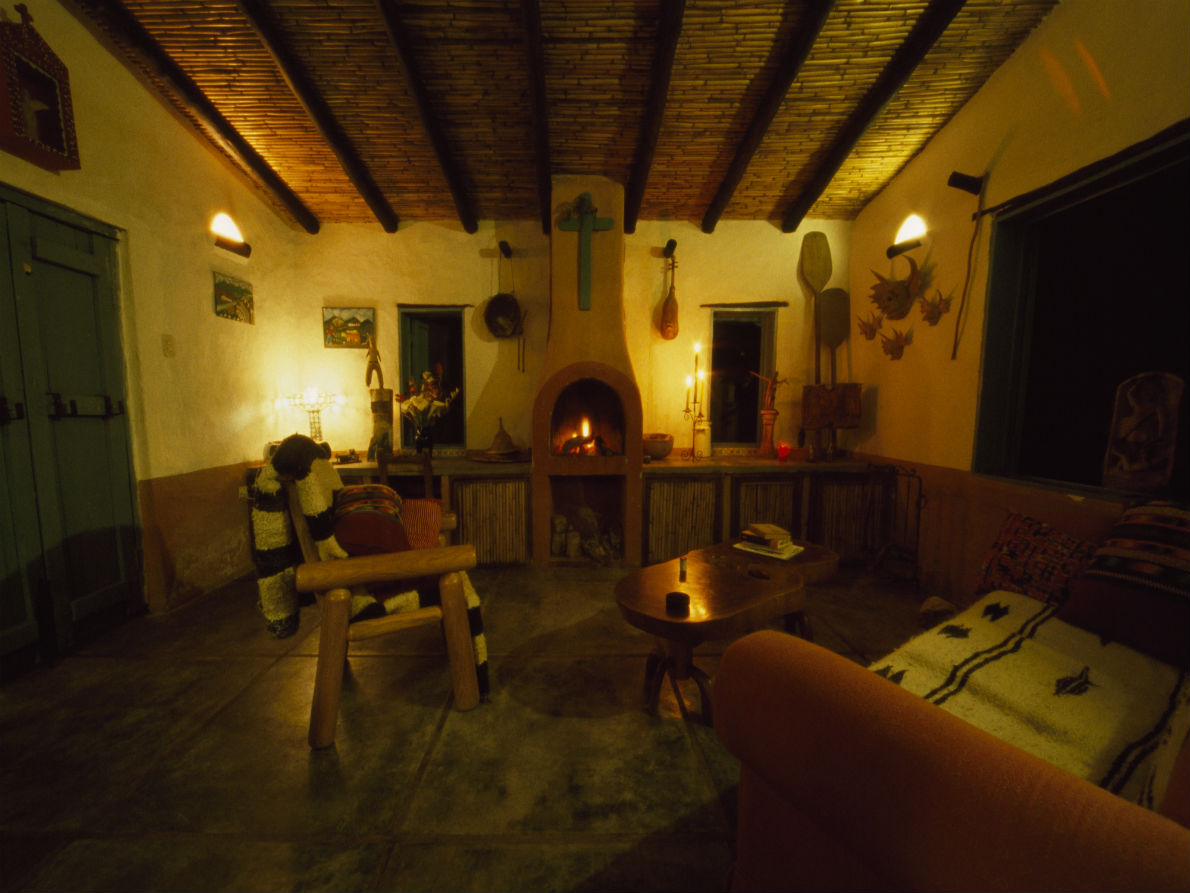

Supplement: Supplementary file 2 [file Data_Sheet_1.zip › Raw Images for Experiment 1/Indoors/id2.jpg]

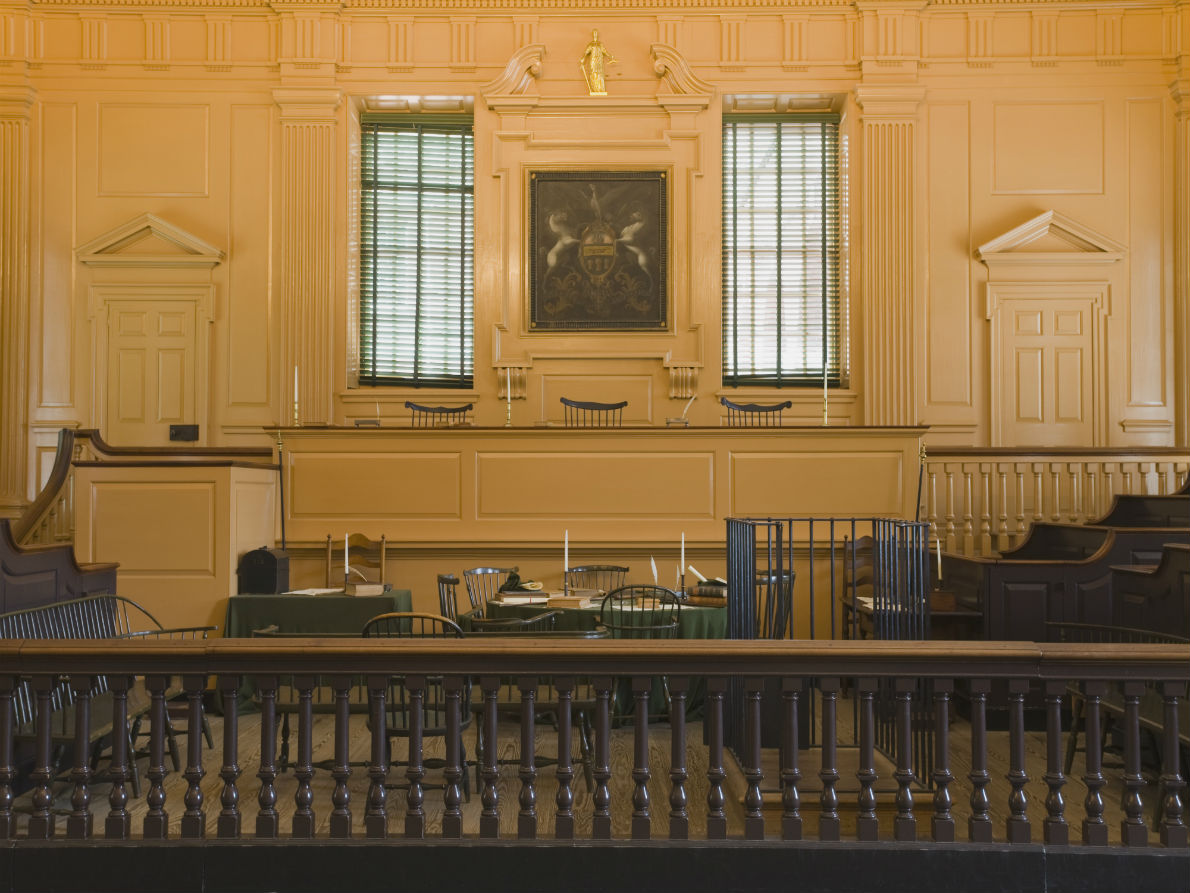

Supplement: Supplementary file 2 [file Data_Sheet_1.zip › Raw Images for Experiment 1/Indoors/id21.jpg]

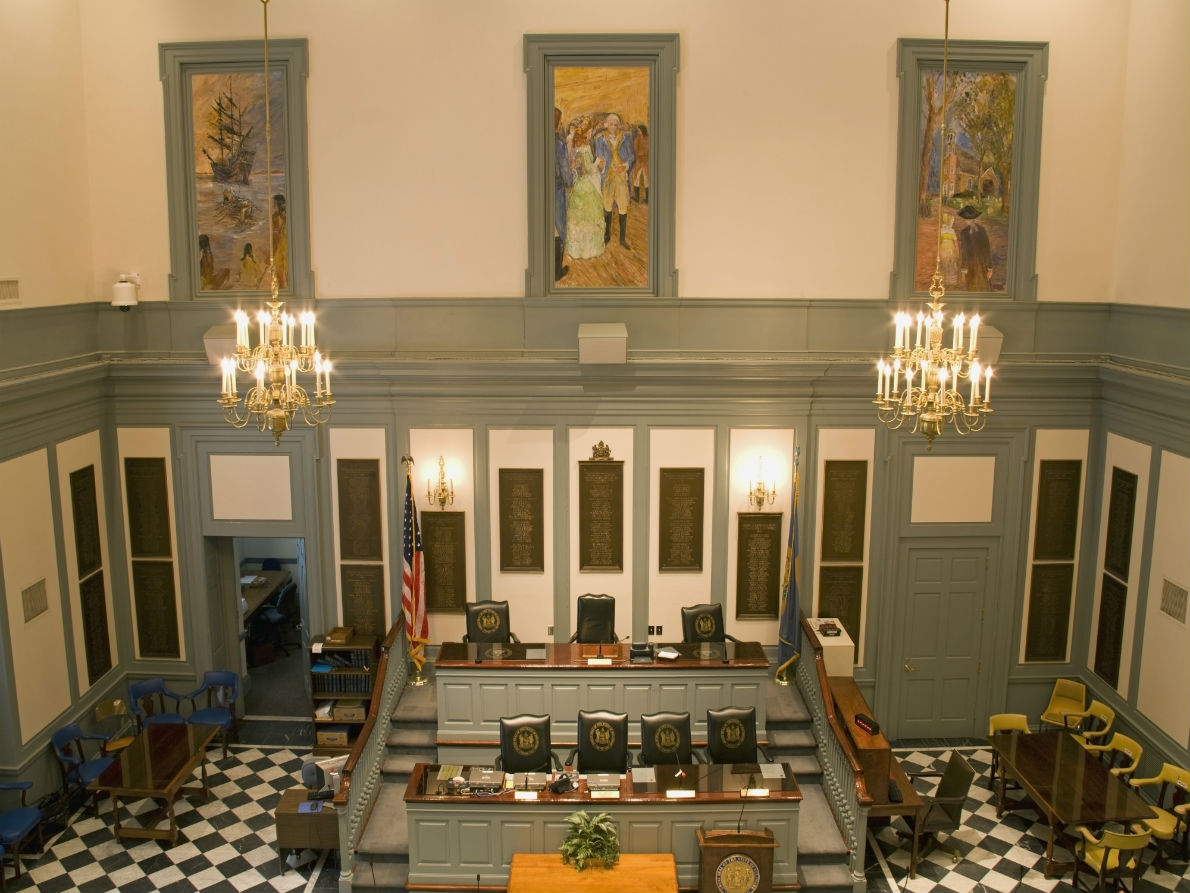

Supplement: Supplementary file 2 [file Data_Sheet_1.zip › Raw Images for Experiment 1/Indoors/id22.jpg]

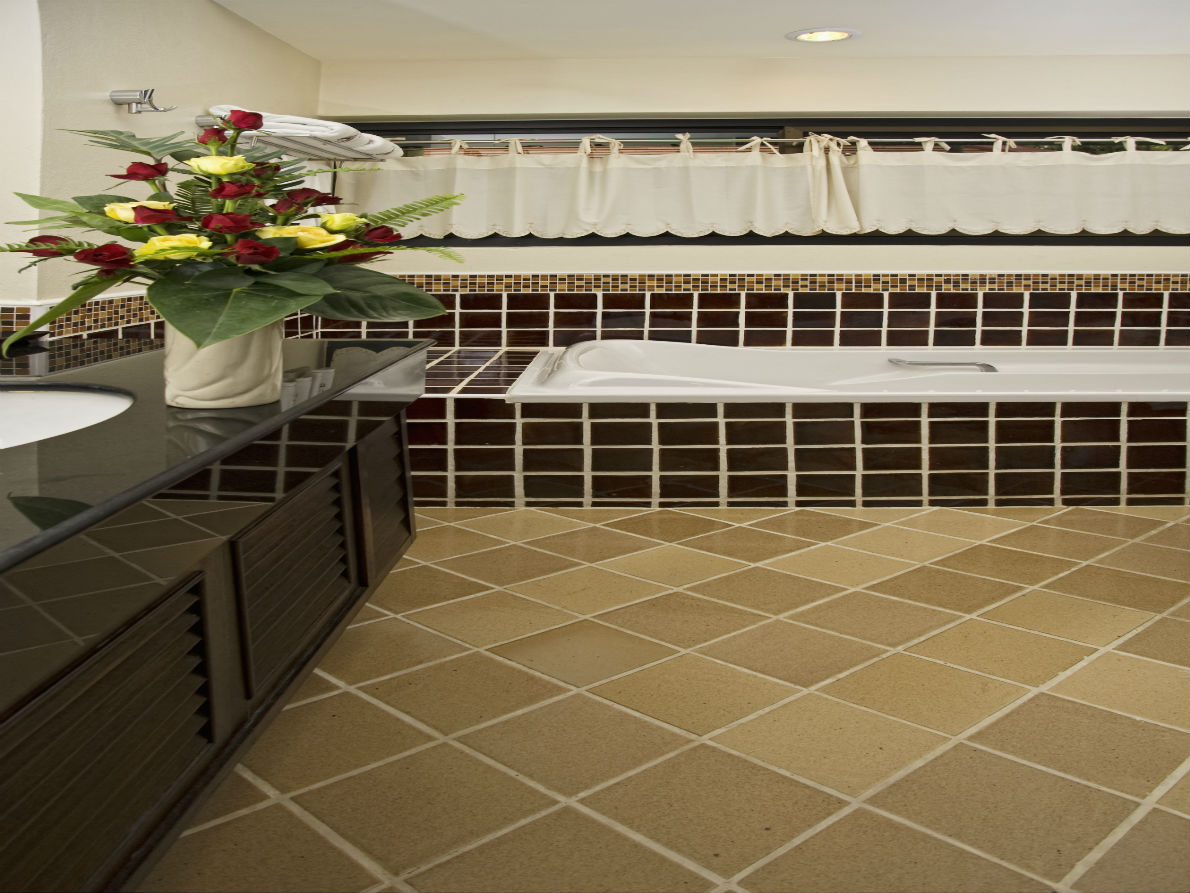

Supplement: Supplementary file 2 [file Data_Sheet_1.zip › Raw Images for Experiment 1/Indoors/id23.jpg]

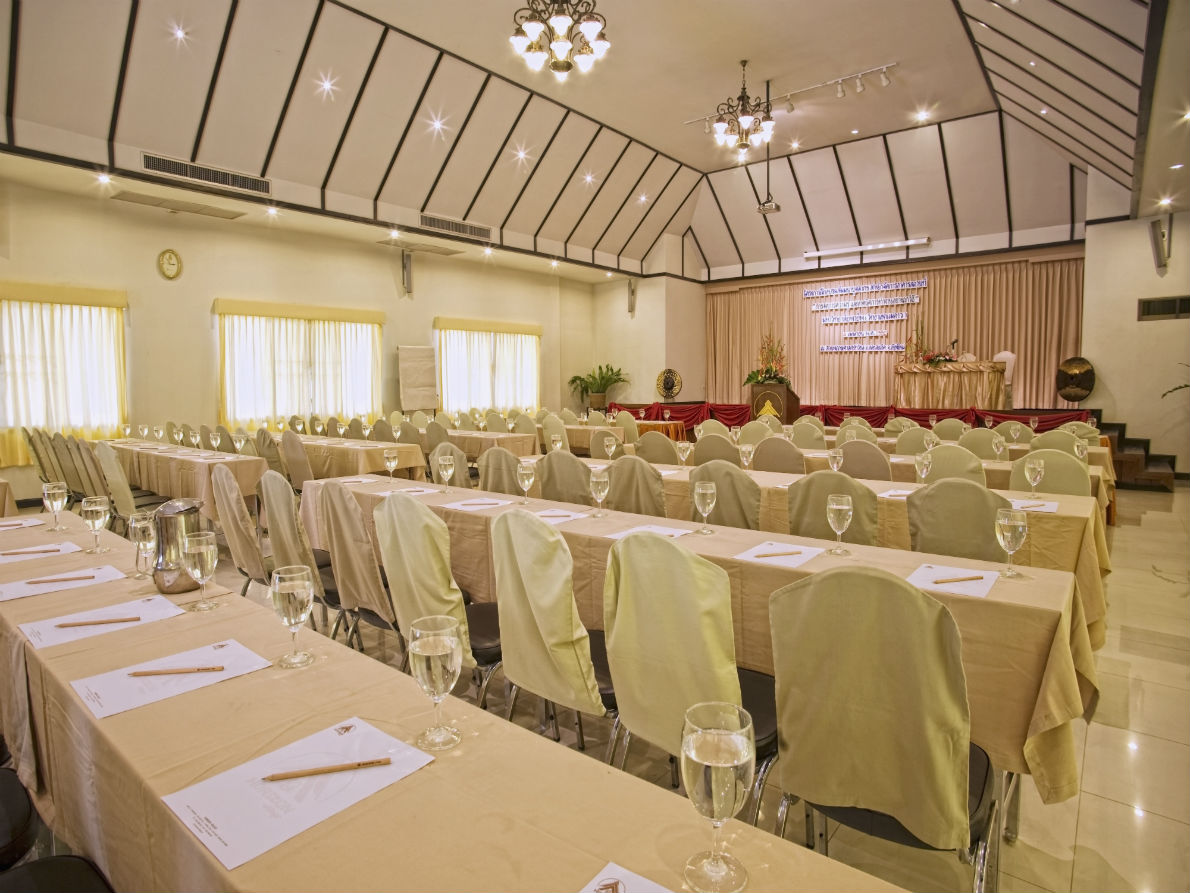

Supplement: Supplementary file 2 [file Data_Sheet_1.zip › Raw Images for Experiment 1/Indoors/id24.jpg]

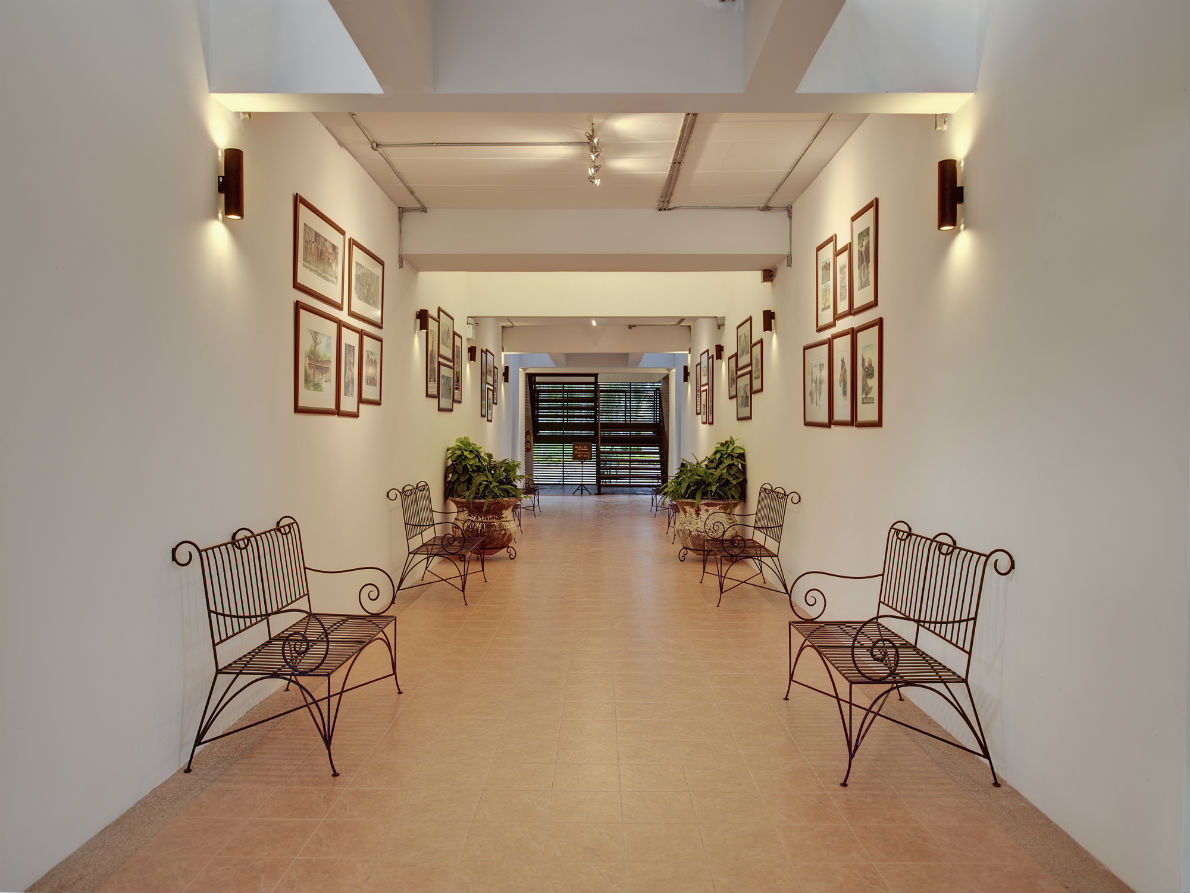

Supplement: Supplementary file 2 [file Data_Sheet_1.zip › Raw Images for Experiment 1/Indoors/id25.jpg]

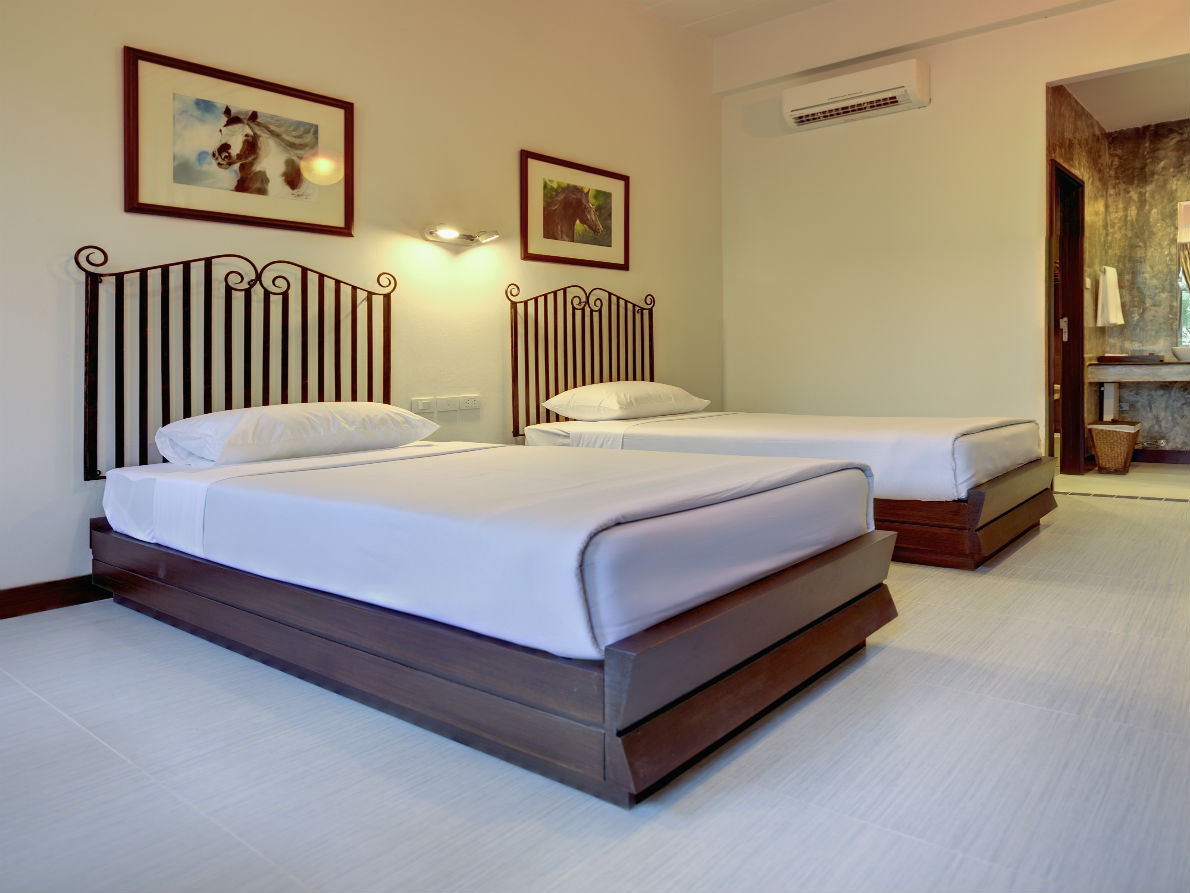

Supplement: Supplementary file 2 [file Data_Sheet_1.zip › Raw Images for Experiment 1/Indoors/id26.jpg]

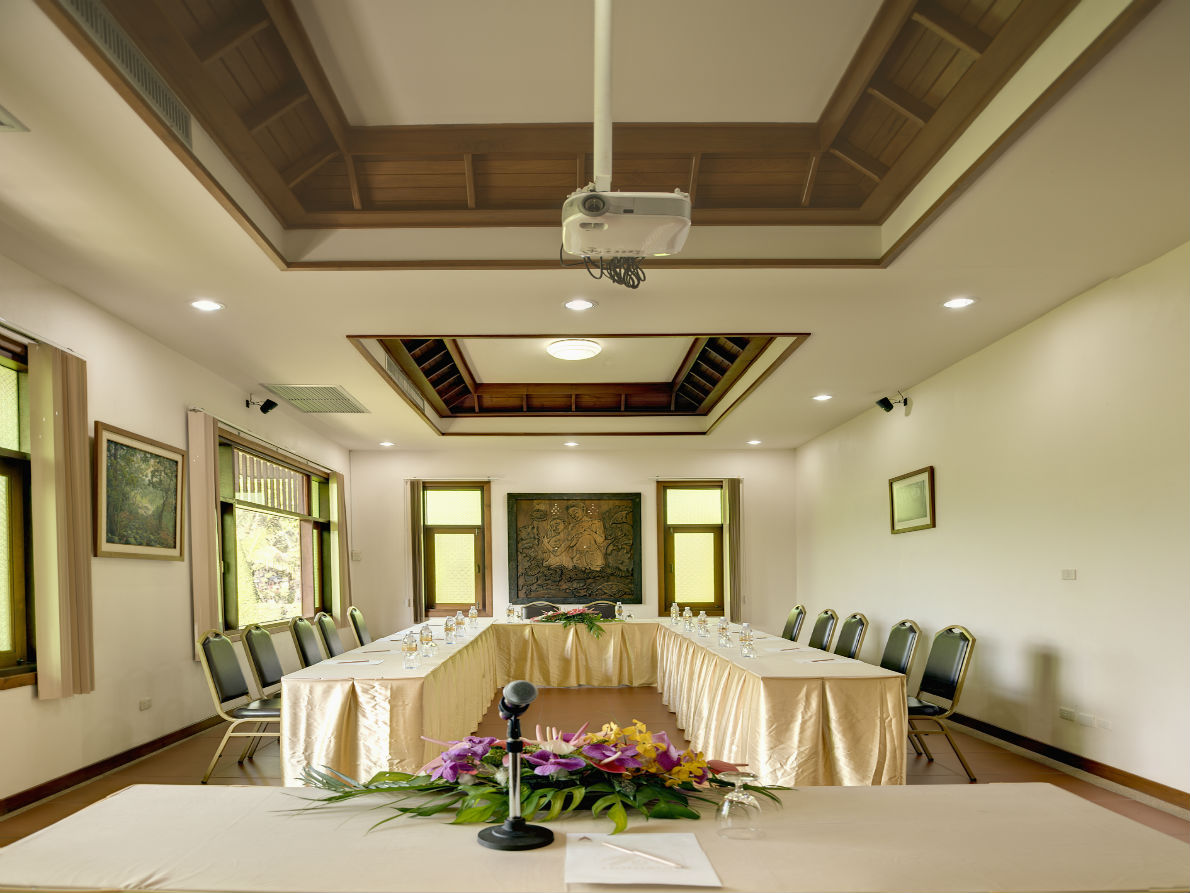

Supplement: Supplementary file 2 [file Data_Sheet_1.zip › Raw Images for Experiment 1/Indoors/id27.jpg]

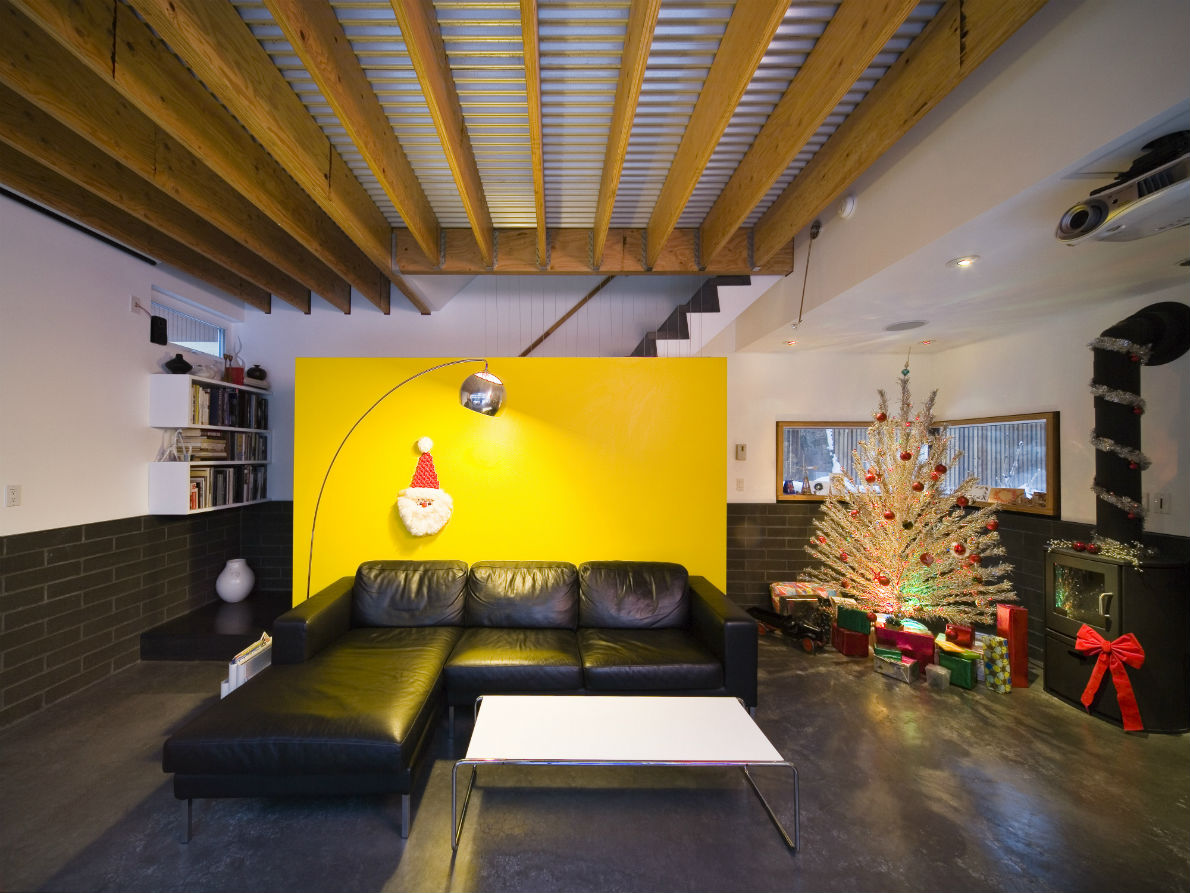

Supplement: Supplementary file 2 [file Data_Sheet_1.zip › Raw Images for Experiment 1/Indoors/id3.jpg]

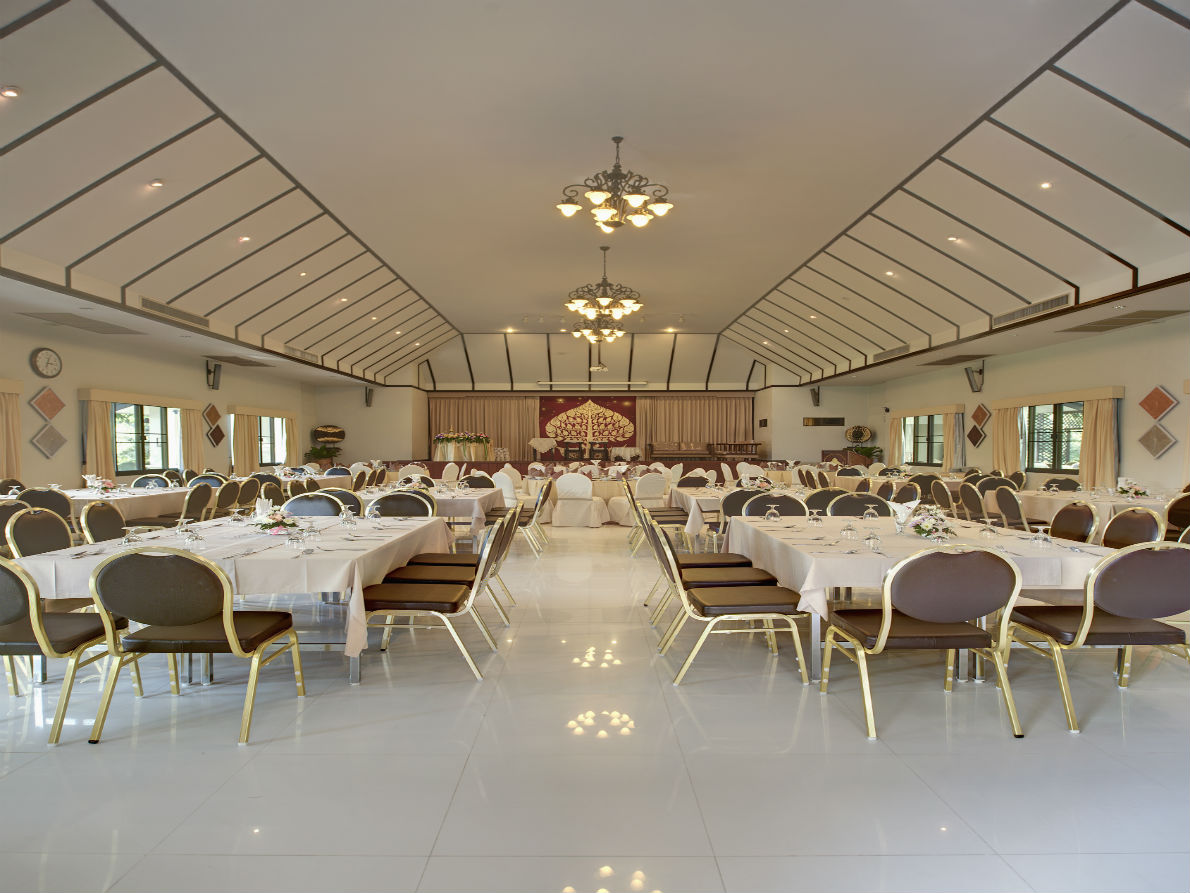

Supplement: Supplementary file 2 [file Data_Sheet_1.zip › Raw Images for Experiment 1/Indoors/id31.jpg]

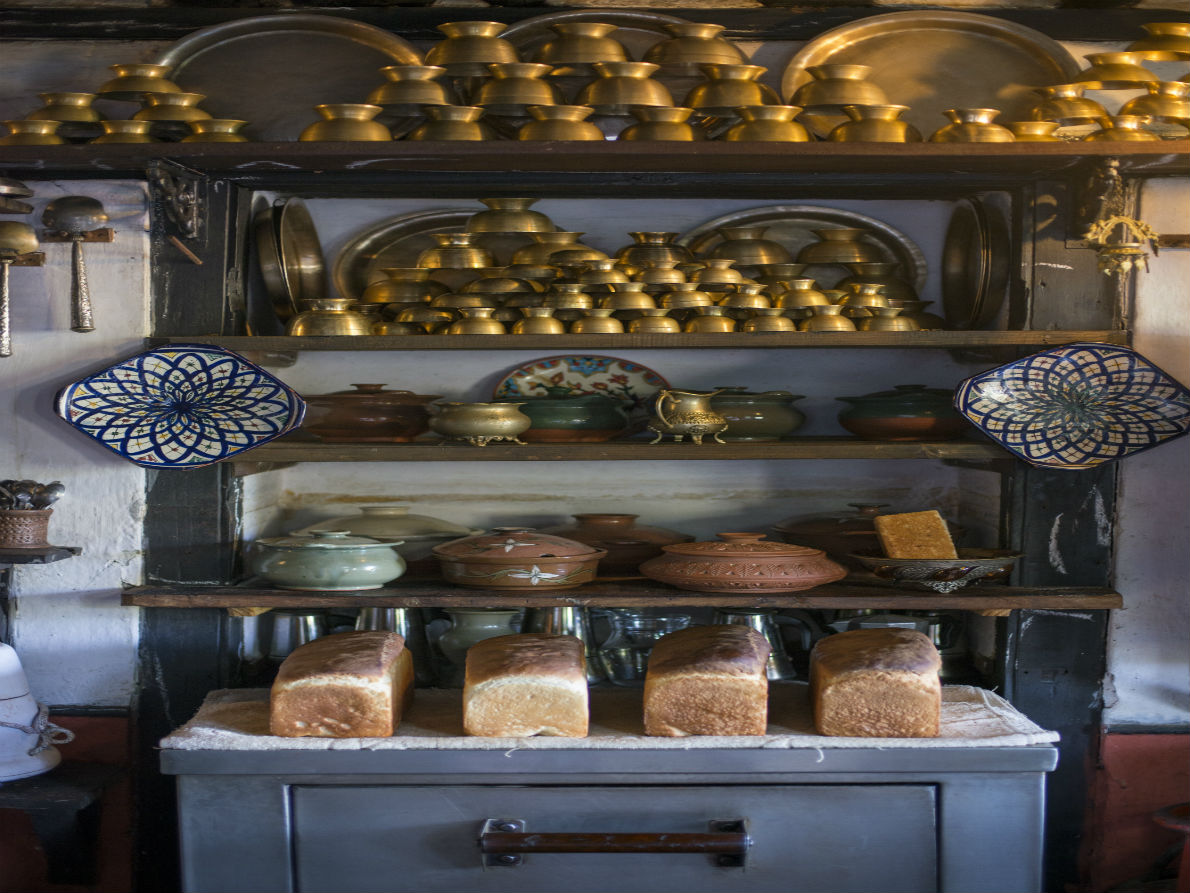

Supplement: Supplementary file 2 [file Data_Sheet_1.zip › Raw Images for Experiment 1/Indoors/id32.jpg]

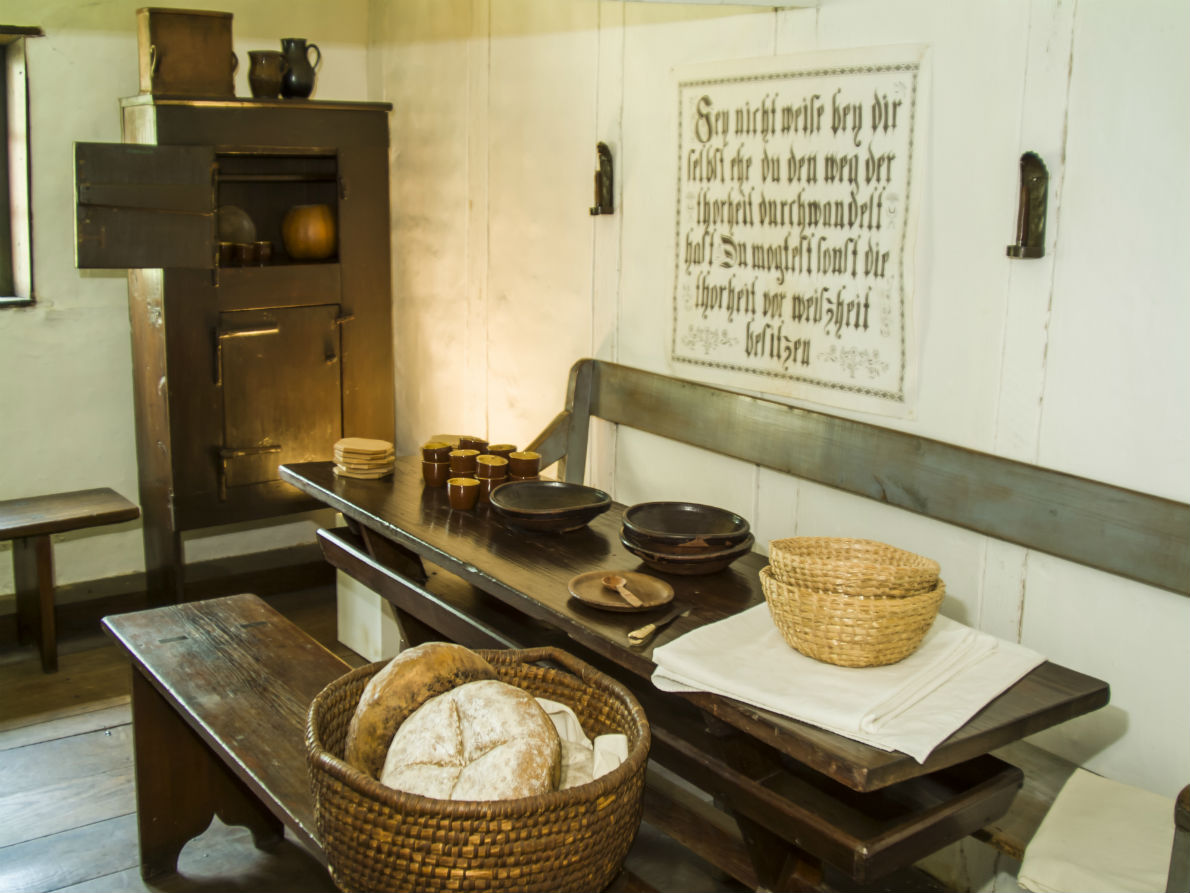

Supplement: Supplementary file 2 [file Data_Sheet_1.zip › Raw Images for Experiment 1/Indoors/id33.jpg]

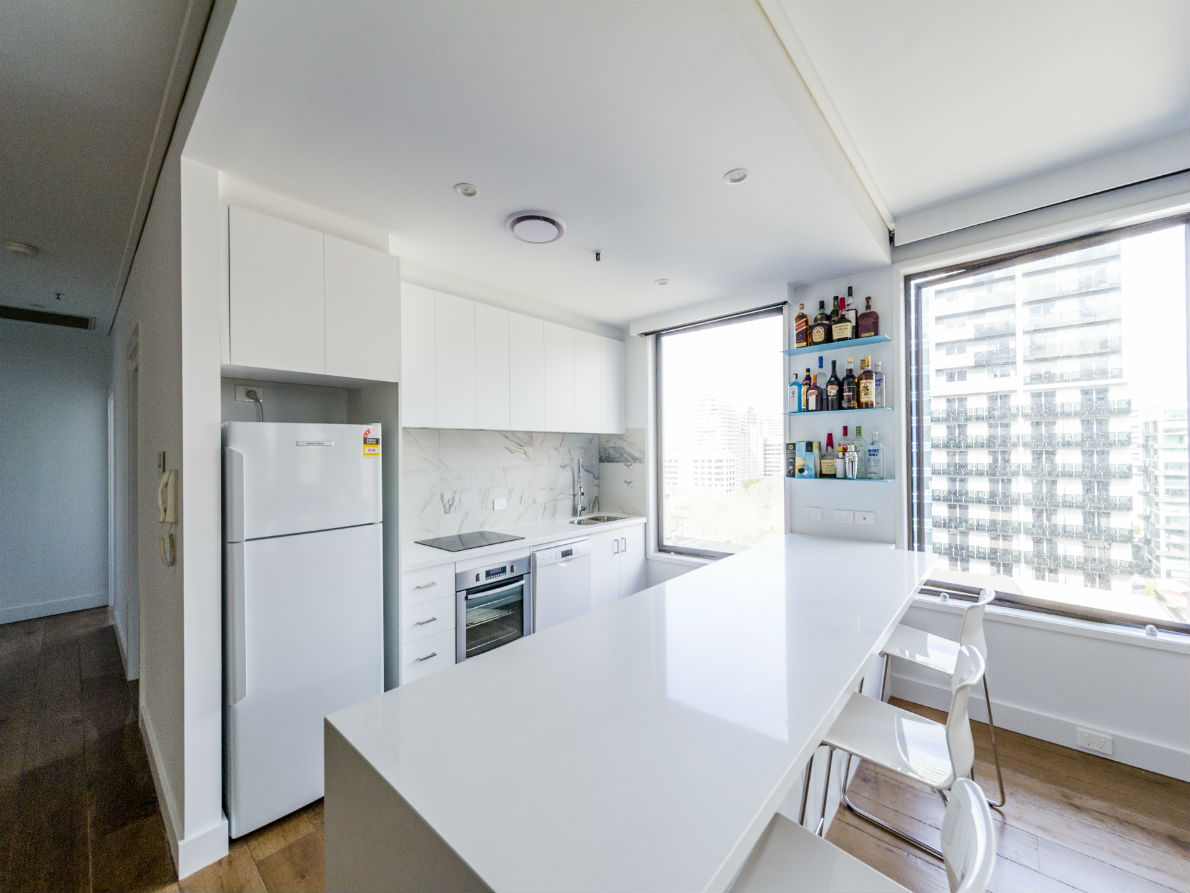

Supplement: Supplementary file 2 [file Data_Sheet_1.zip › Raw Images for Experiment 1/Indoors/id36.jpg]

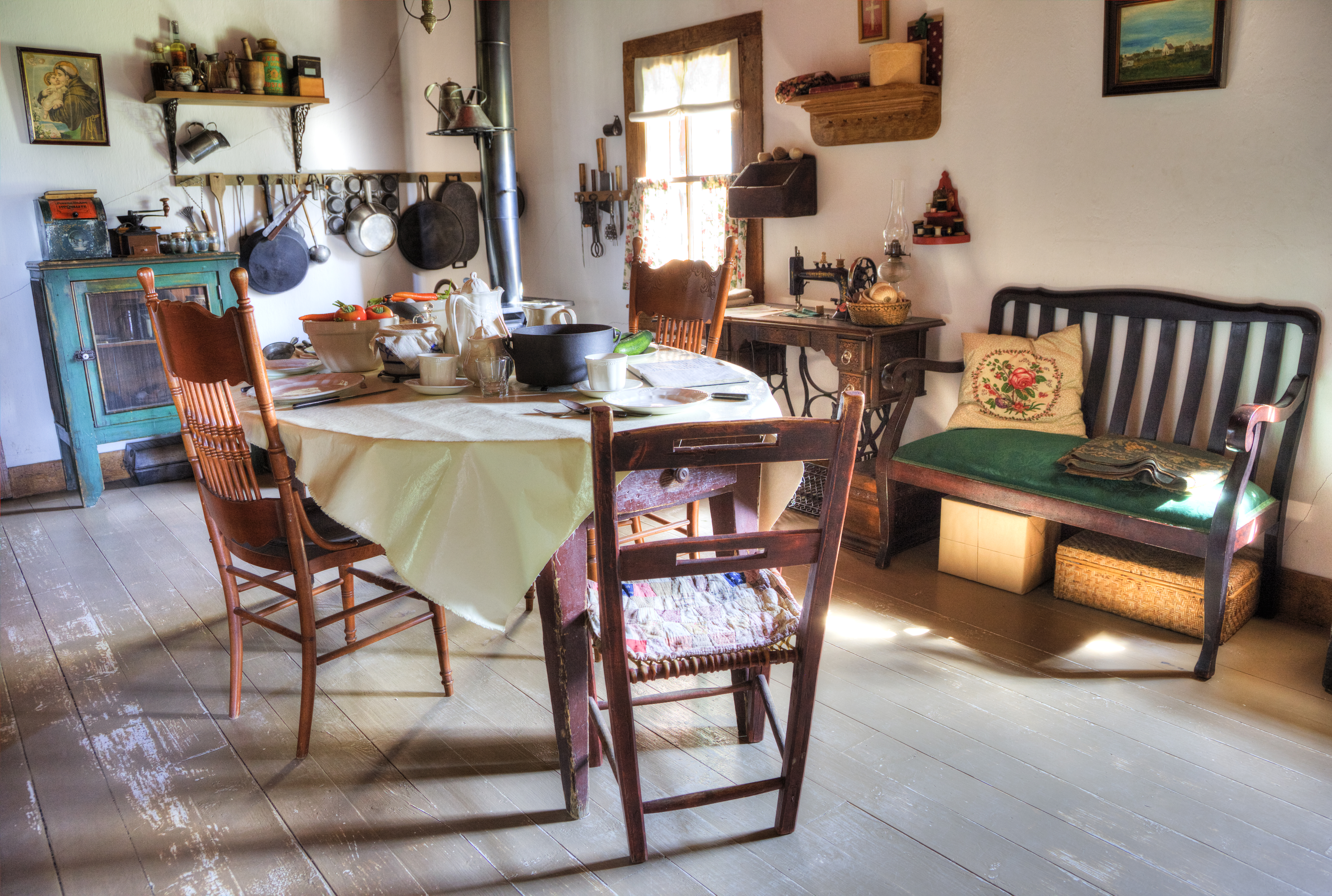

Supplement: Supplementary file 2 [file Data_Sheet_1.zip › Raw Images for Experiment 1/Indoors/id37.jpg]

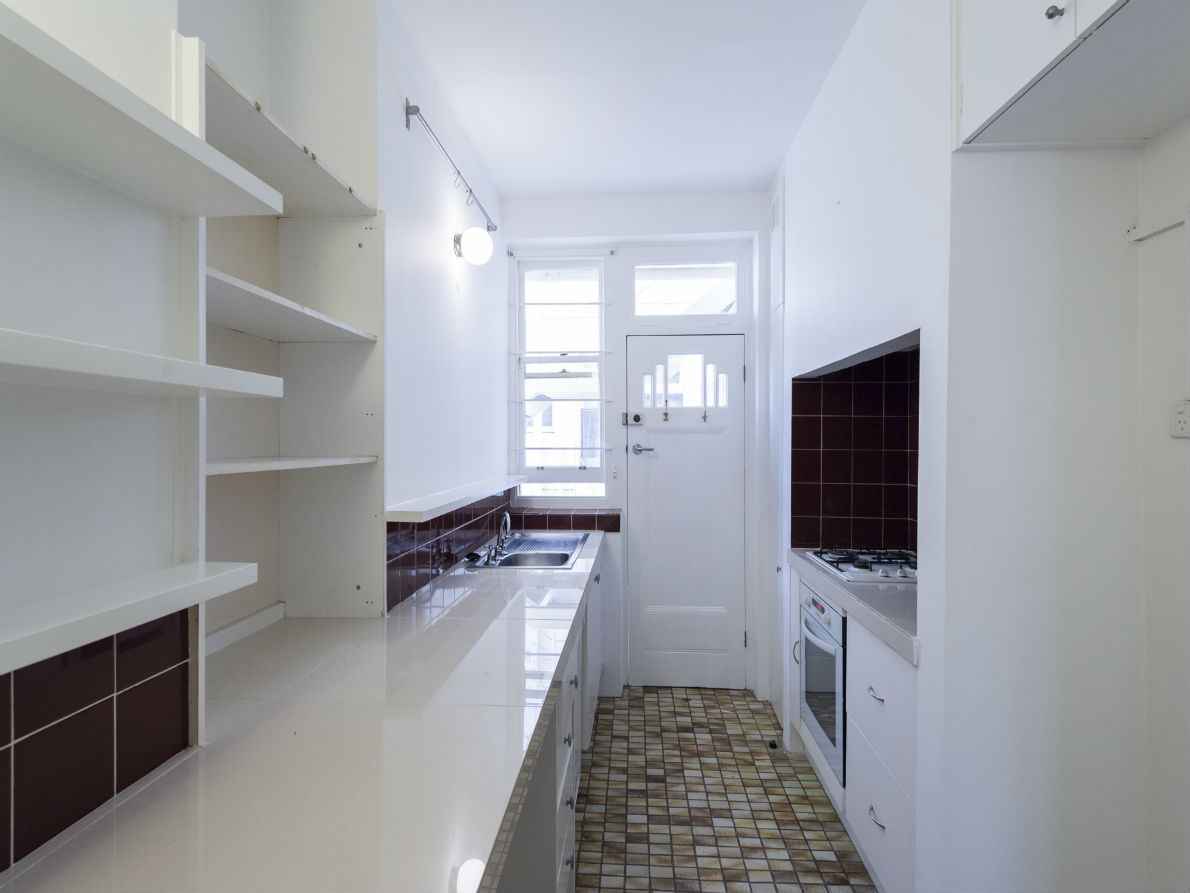

Supplement: Supplementary file 2 [file Data_Sheet_1.zip › Raw Images for Experiment 1/Indoors/id39.jpg]

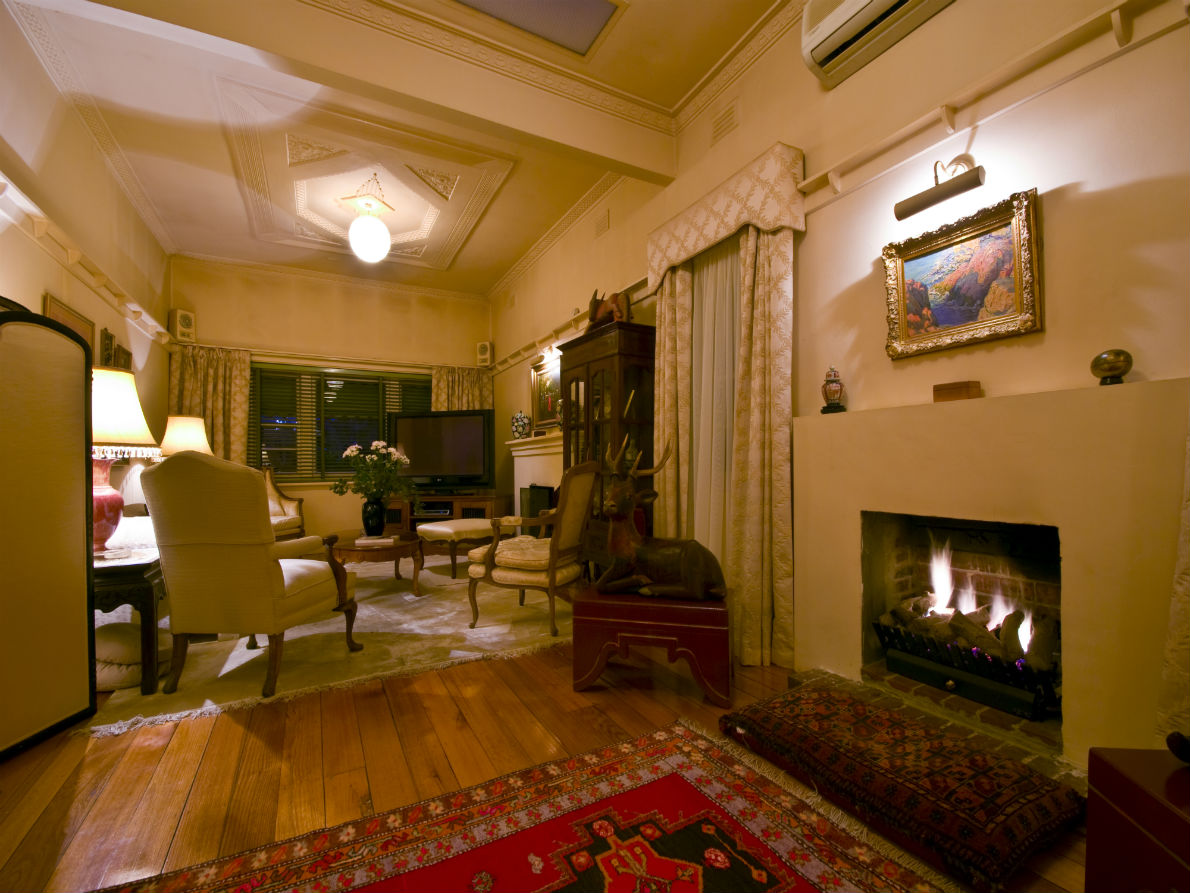

Supplement: Supplementary file 2 [file Data_Sheet_1.zip › Raw Images for Experiment 1/Indoors/id6.jpg]

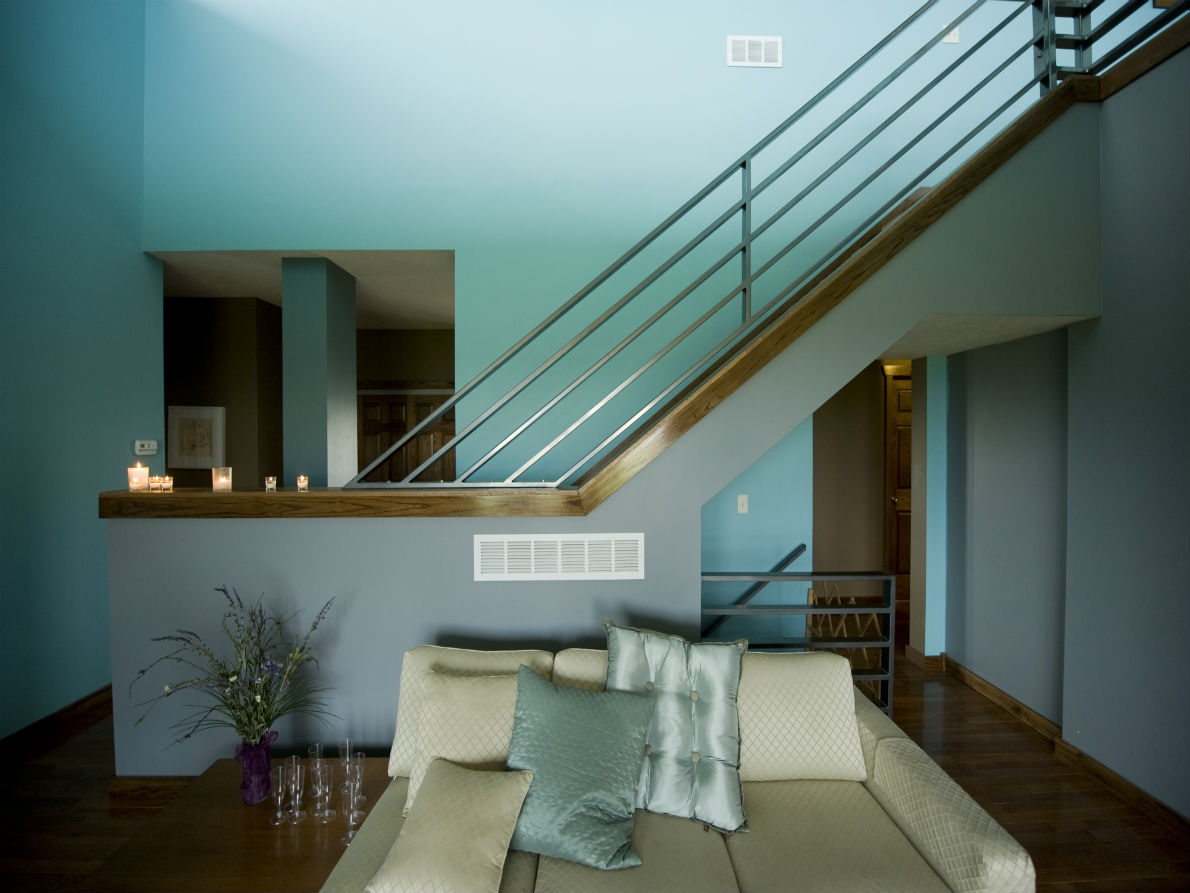

Supplement: Supplementary file 2 [file Data_Sheet_1.zip › Raw Images for Experiment 1/Indoors/id8.jpg]

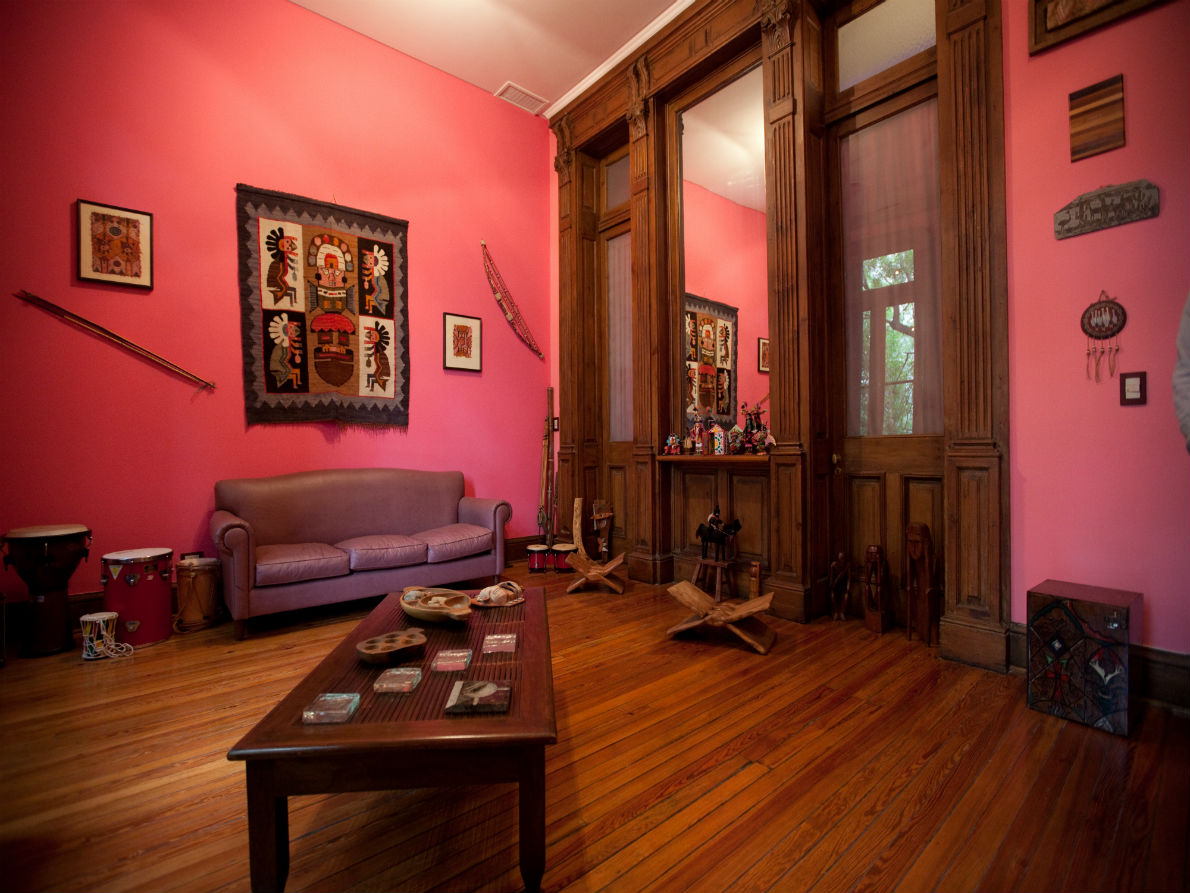

Supplement: Supplementary file 2 [file Data_Sheet_1.zip › Raw Images for Experiment 1/Indoors/id9.jpg]
