# Supplementary figures and images for: What Makes an Image Interesting and How Can We Explain It (part 3 of 3)
Source: Front Psychol. 2021 Sep 1;12:668651. doi: 10.3389/fpsyg.2021.668651 (PMC8440840; doi:10.3389/fpsyg.2021.668651)

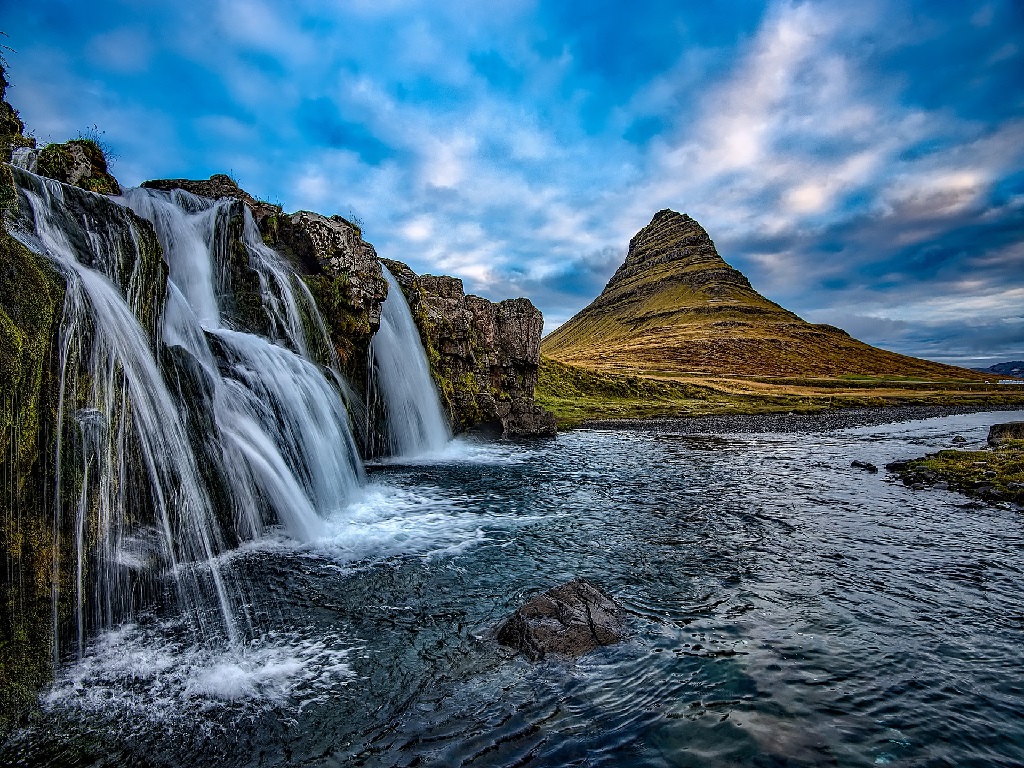

Supplement: Supplementary file 3 [file Data_Sheet_2.zip › Raw Images for Experiment 2/Land/land06.jpg]

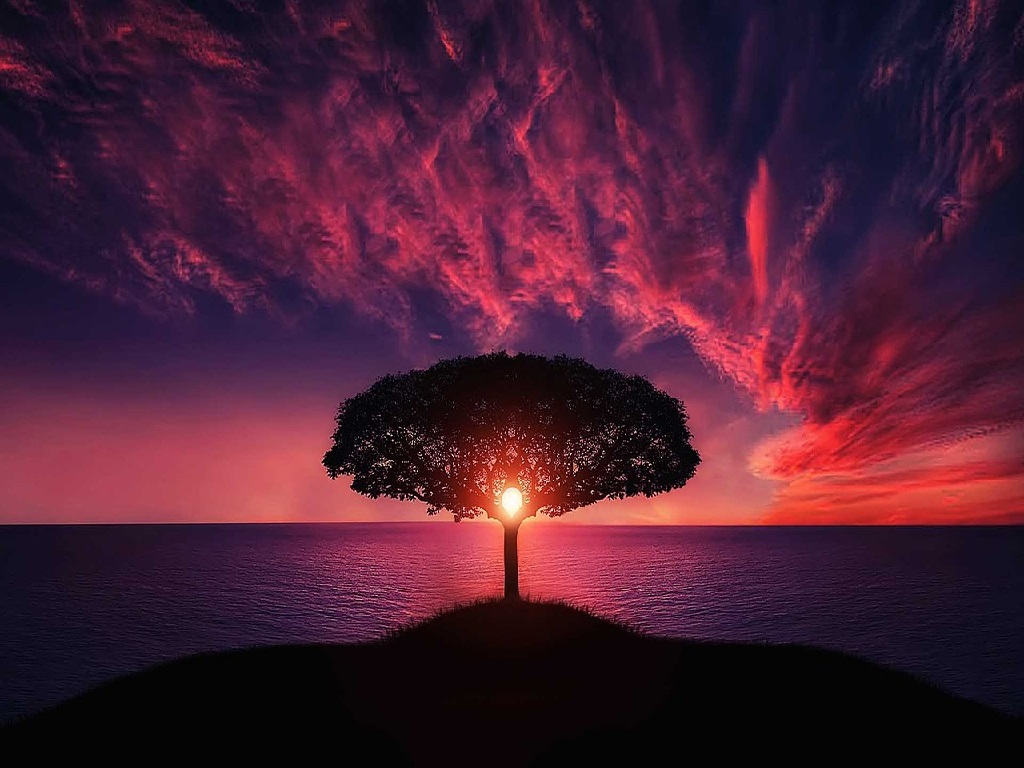

Supplement: Supplementary file 3 [file Data_Sheet_2.zip › Raw Images for Experiment 2/Land/land09.jpg]

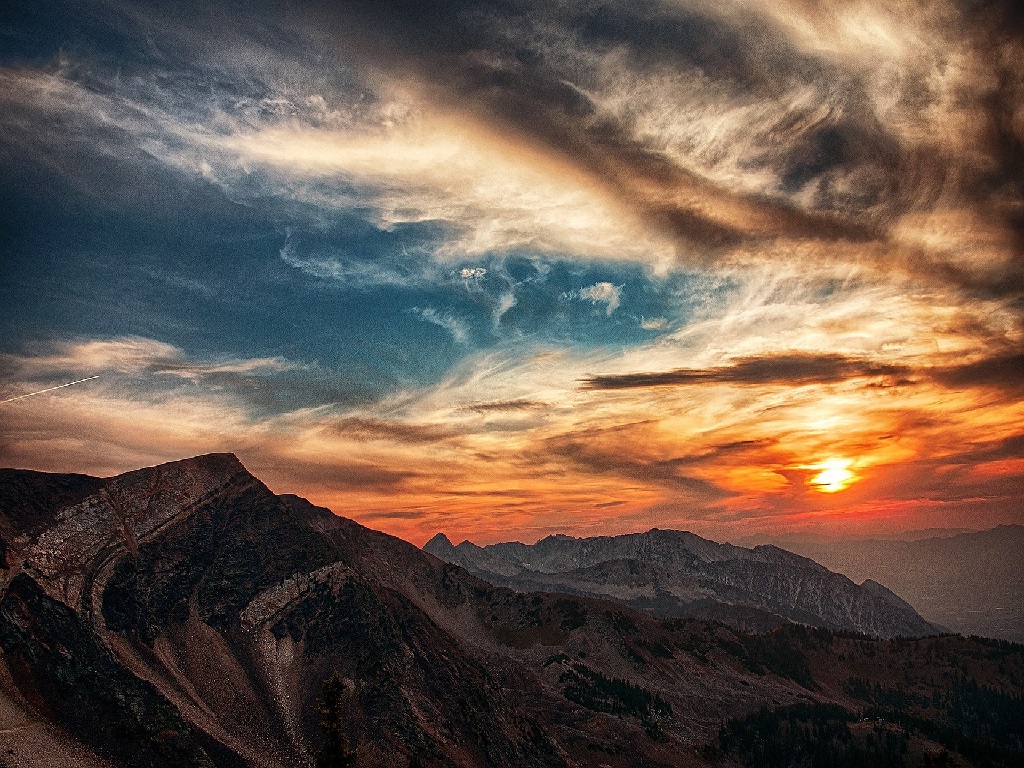

Supplement: Supplementary file 3 [file Data_Sheet_2.zip › Raw Images for Experiment 2/Land/land12.jpg]

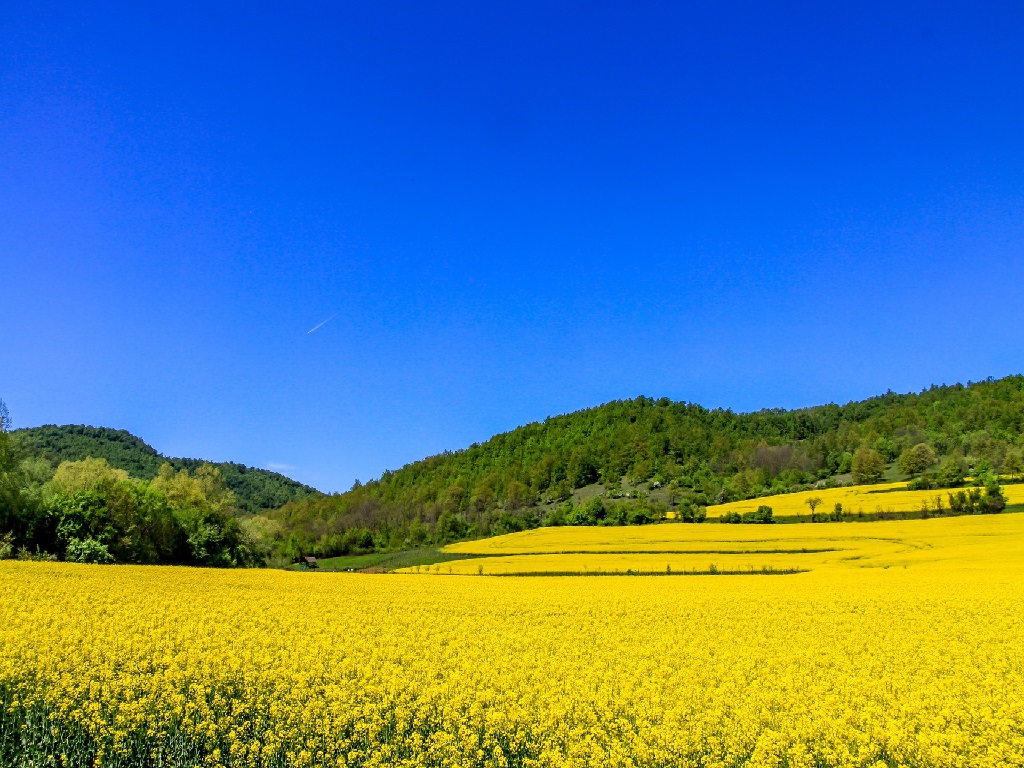

Supplement: Supplementary file 3 [file Data_Sheet_2.zip › Raw Images for Experiment 2/Land/land14.jpg]

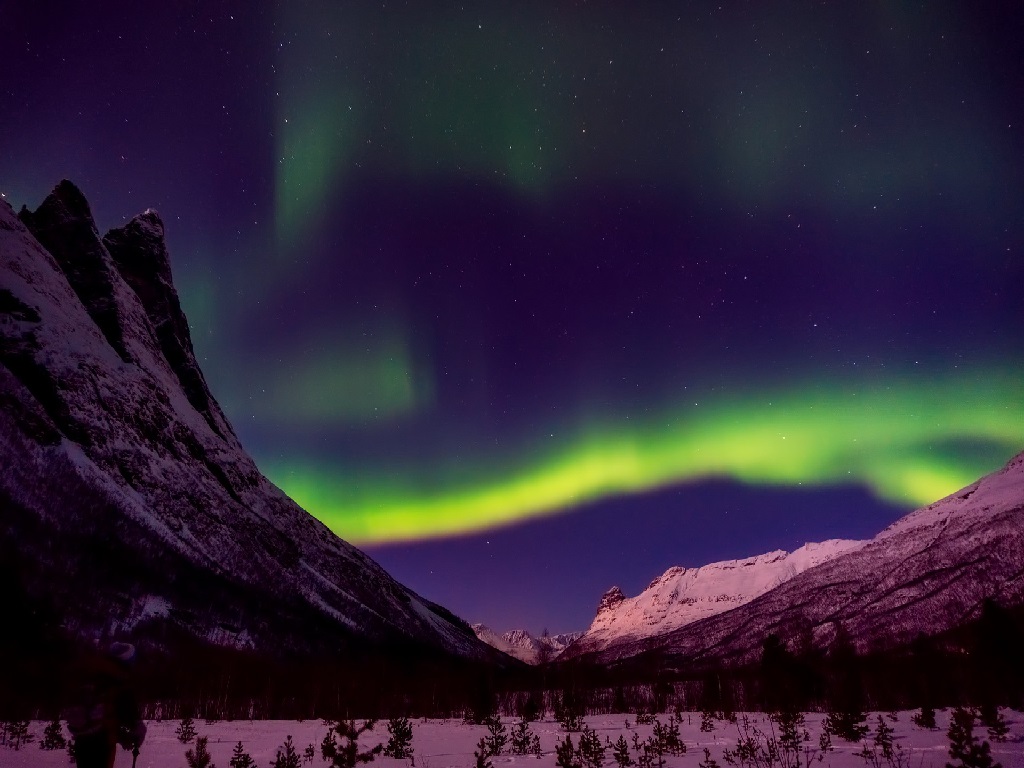

Supplement: Supplementary file 3 [file Data_Sheet_2.zip › Raw Images for Experiment 2/Land/land16.jpg]

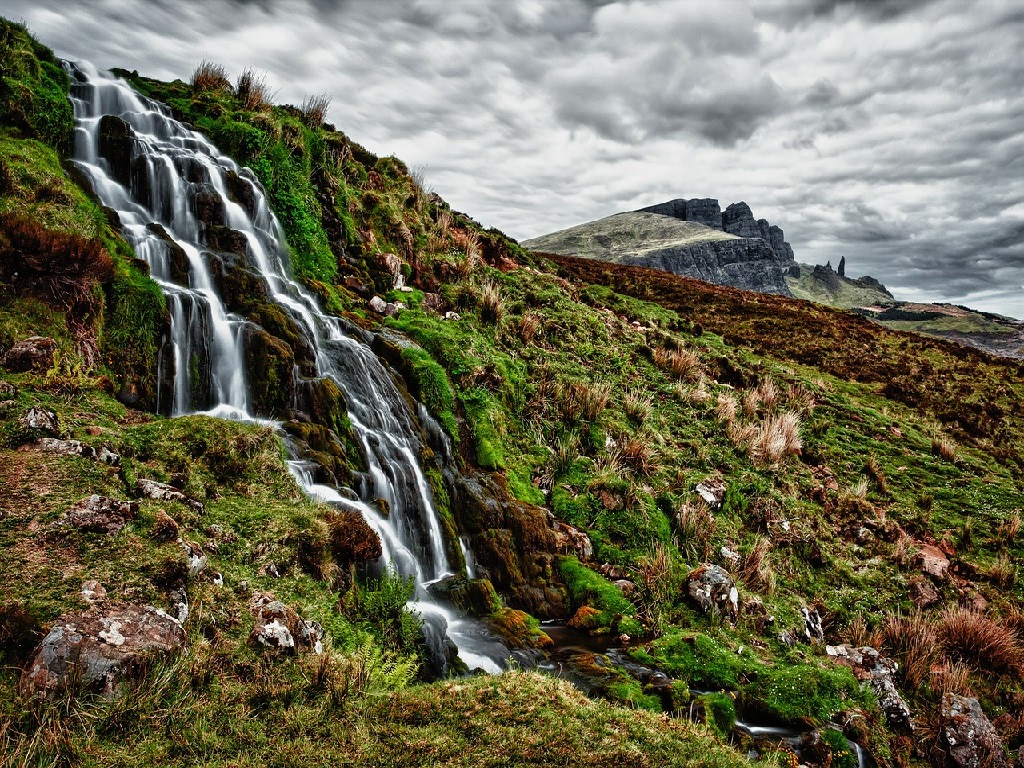

Supplement: Supplementary file 3 [file Data_Sheet_2.zip › Raw Images for Experiment 2/Land/land17.jpg]

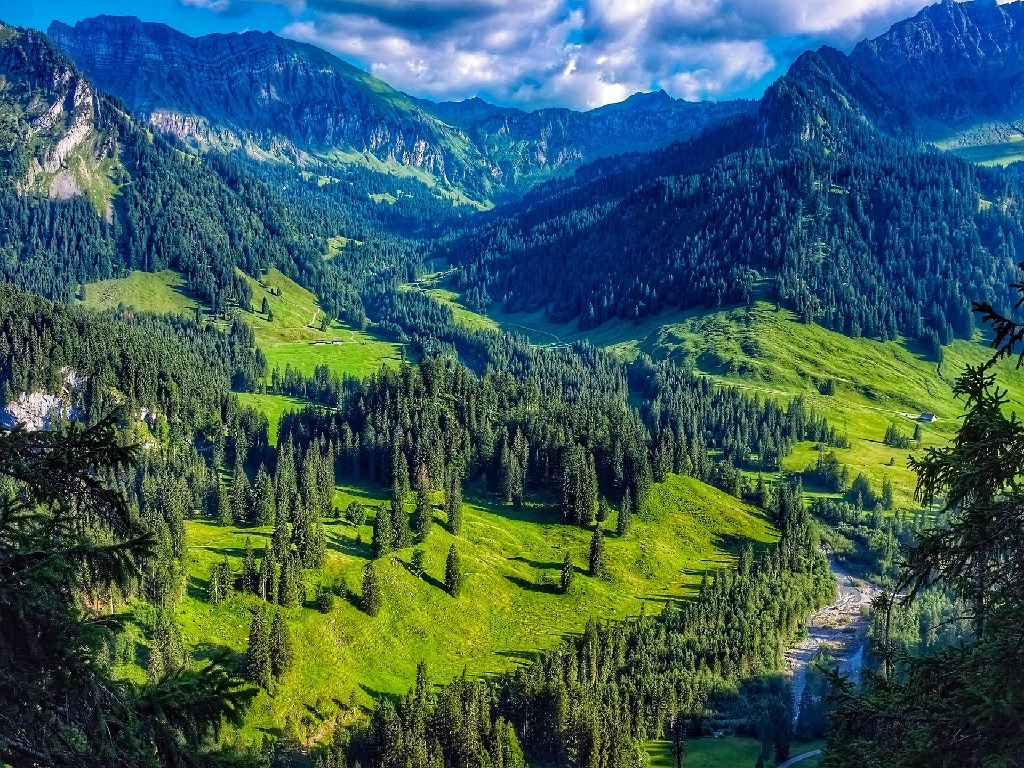

Supplement: Supplementary file 3 [file Data_Sheet_2.zip › Raw Images for Experiment 2/Land/land18.jpg]

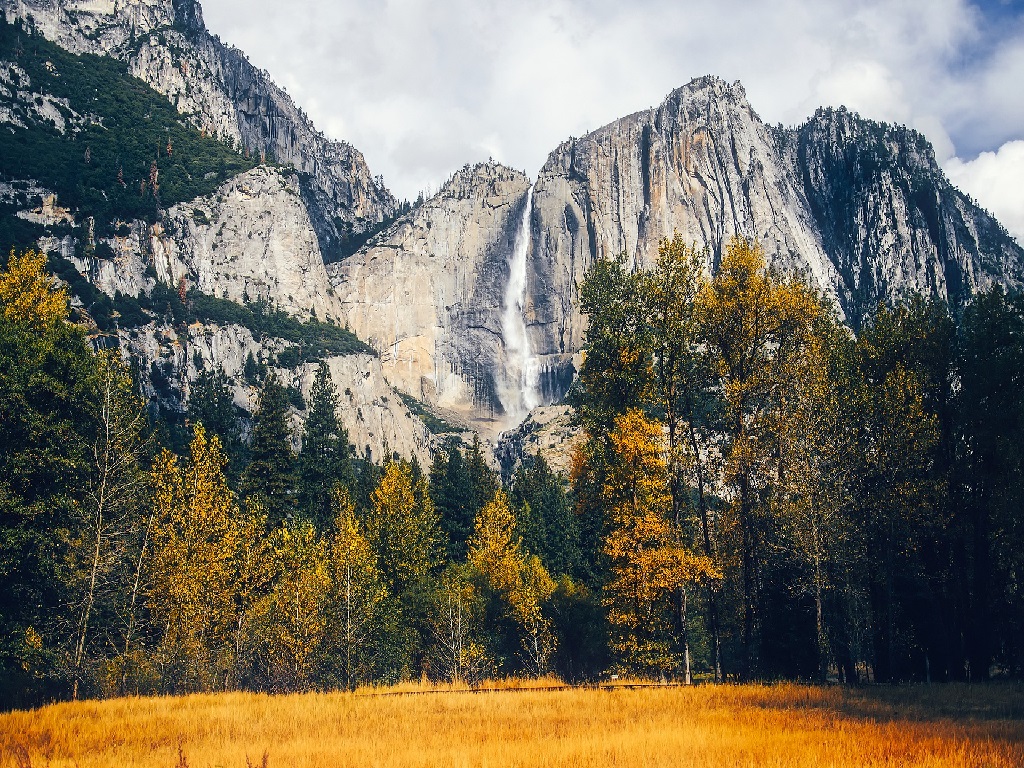

Supplement: Supplementary file 3 [file Data_Sheet_2.zip › Raw Images for Experiment 2/Land/land21.jpg]

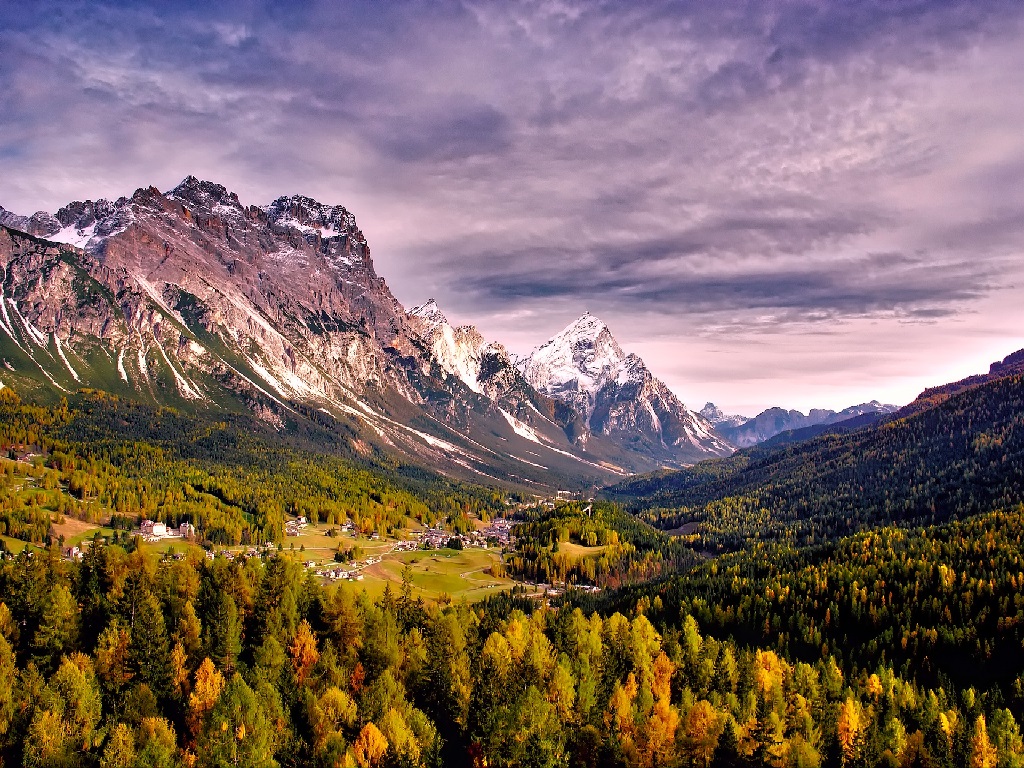

Supplement: Supplementary file 3 [file Data_Sheet_2.zip › Raw Images for Experiment 2/Land/land23.jpg]

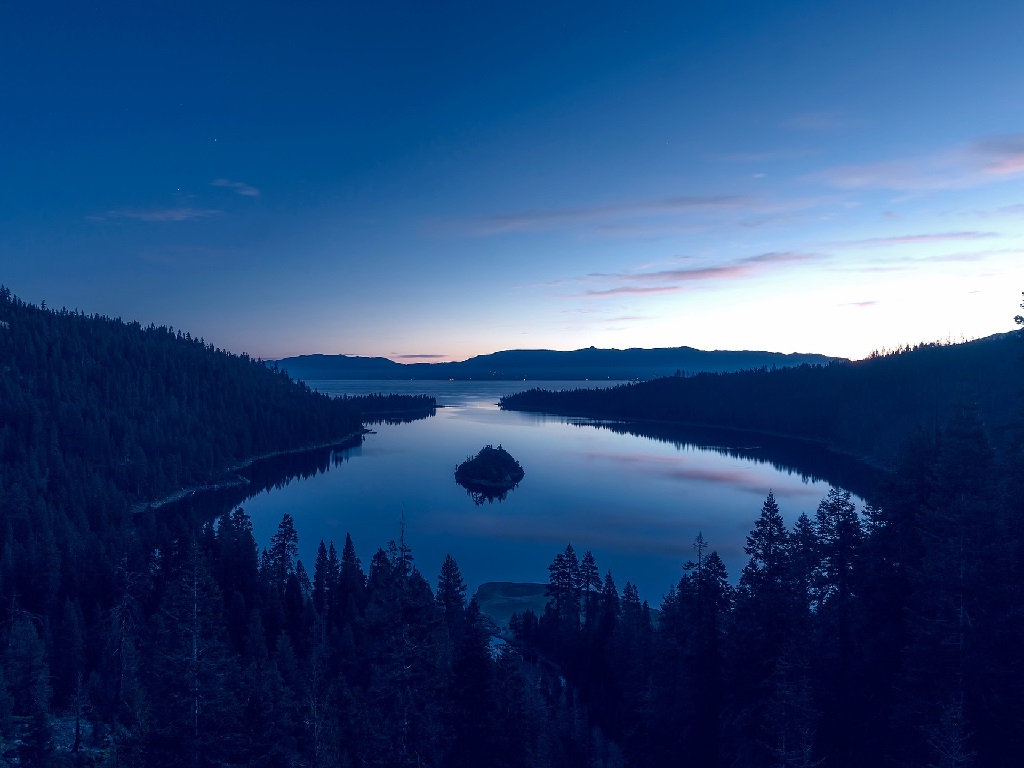

Supplement: Supplementary file 3 [file Data_Sheet_2.zip › Raw Images for Experiment 2/Land/land27.jpg]

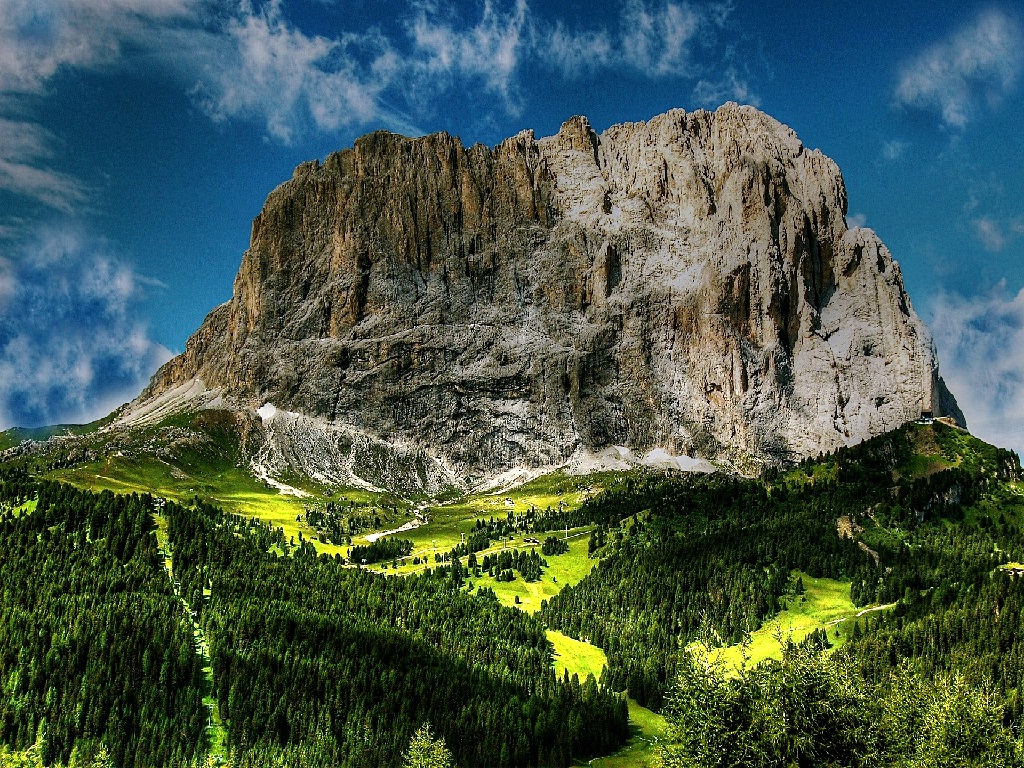

Supplement: Supplementary file 3 [file Data_Sheet_2.zip › Raw Images for Experiment 2/Land/land28.jpg]

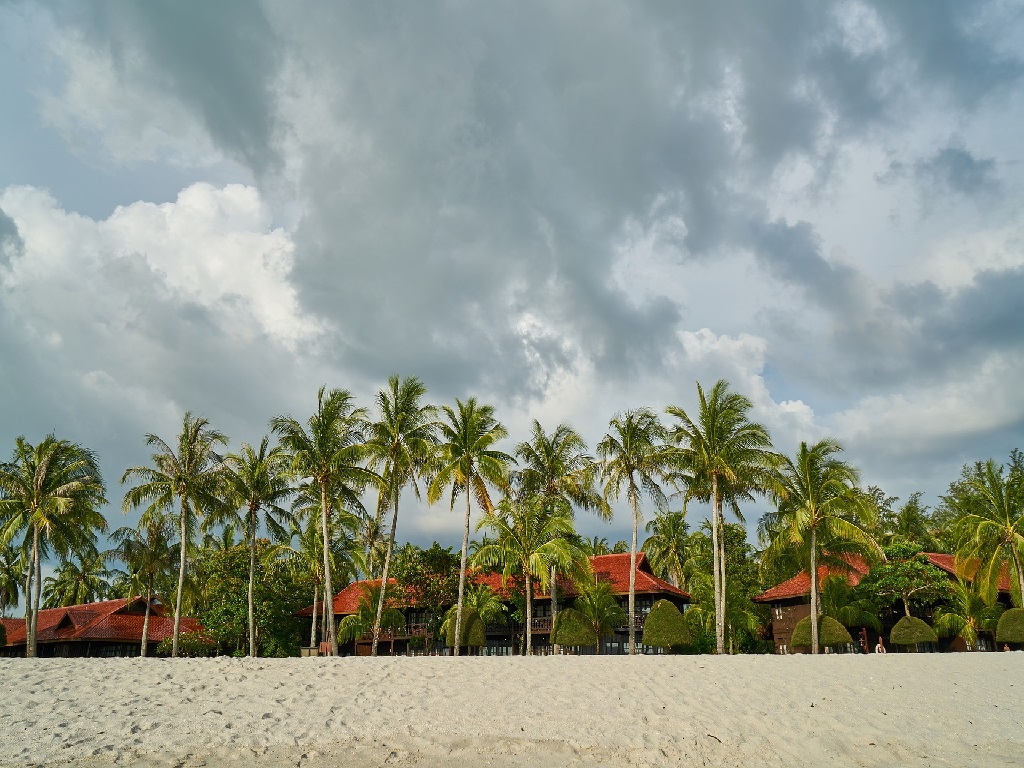

Supplement: Supplementary file 3 [file Data_Sheet_2.zip › Raw Images for Experiment 2/Land/land30.jpg]

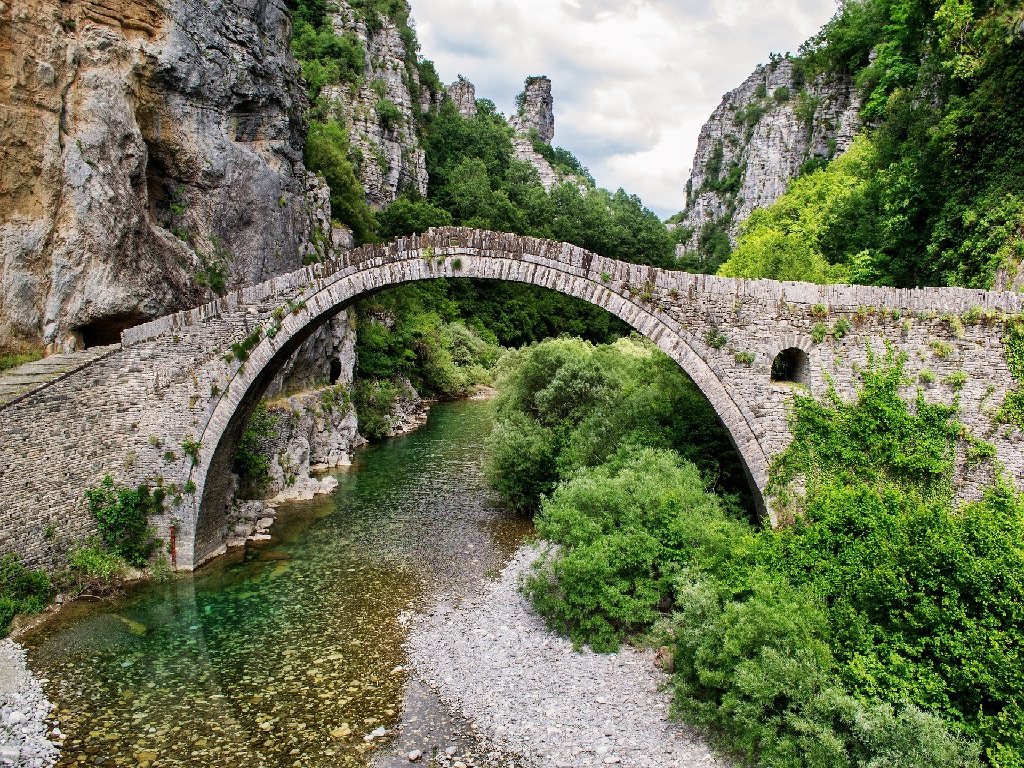

Supplement: Supplementary file 3 [file Data_Sheet_2.zip › Raw Images for Experiment 2/Land/land31.jpg]

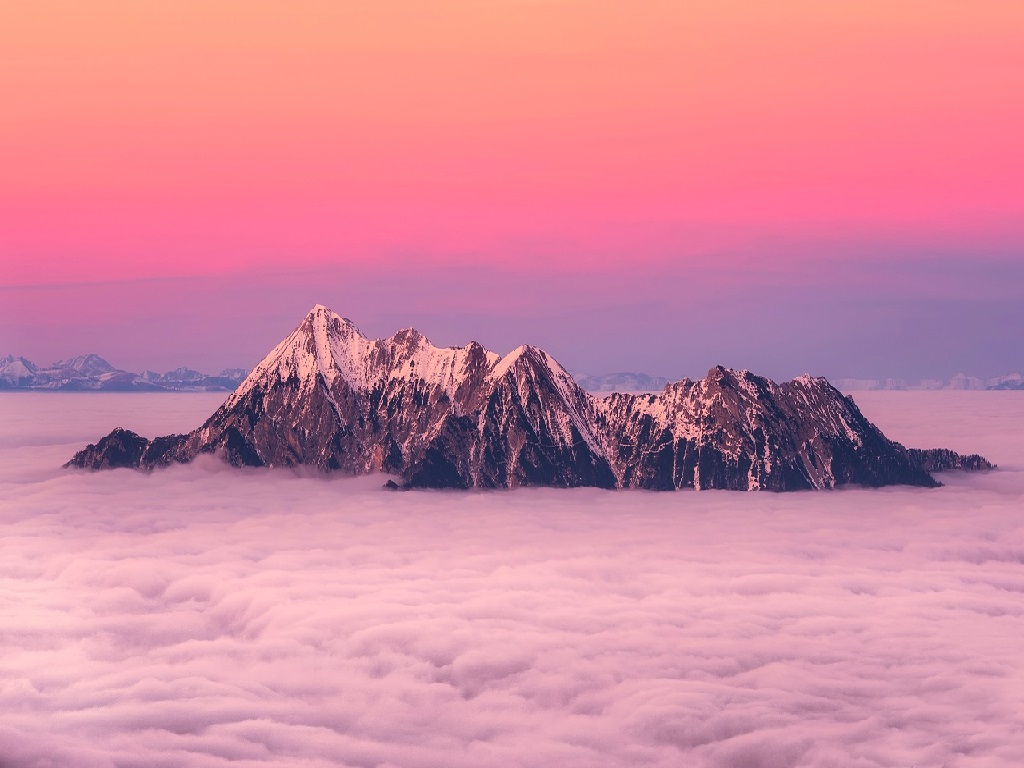

Supplement: Supplementary file 3 [file Data_Sheet_2.zip › Raw Images for Experiment 2/Land/land32.jpg]

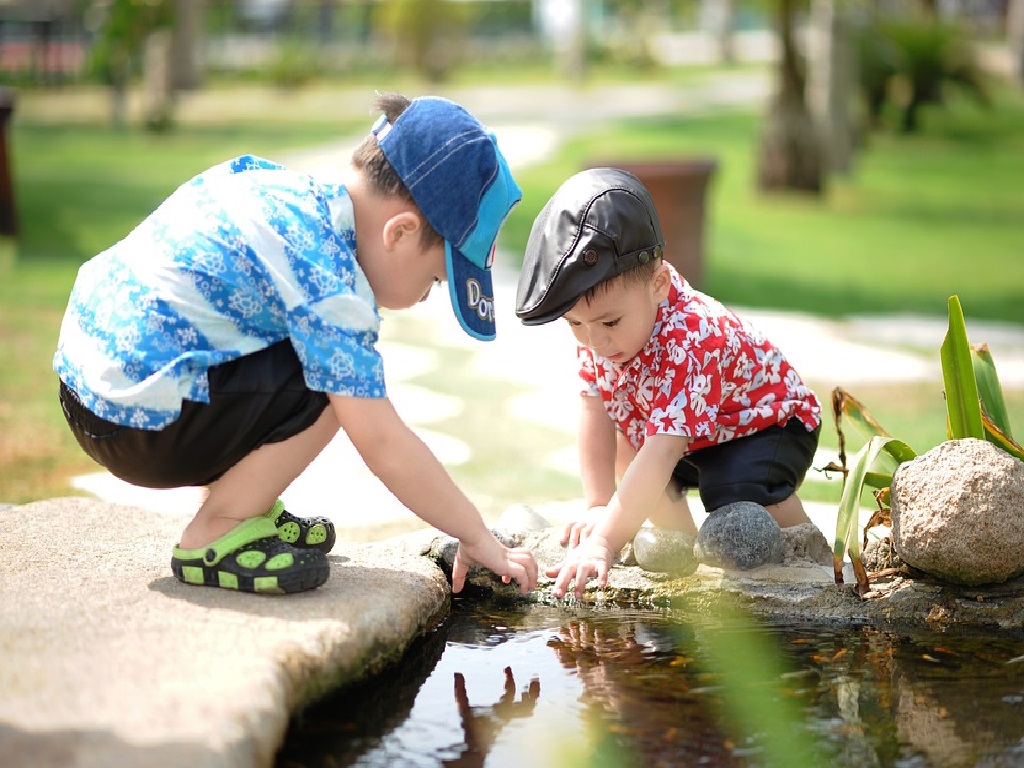

Supplement: Supplementary file 3 [file Data_Sheet_2.zip › Raw Images for Experiment 2/People/people01.jpg]

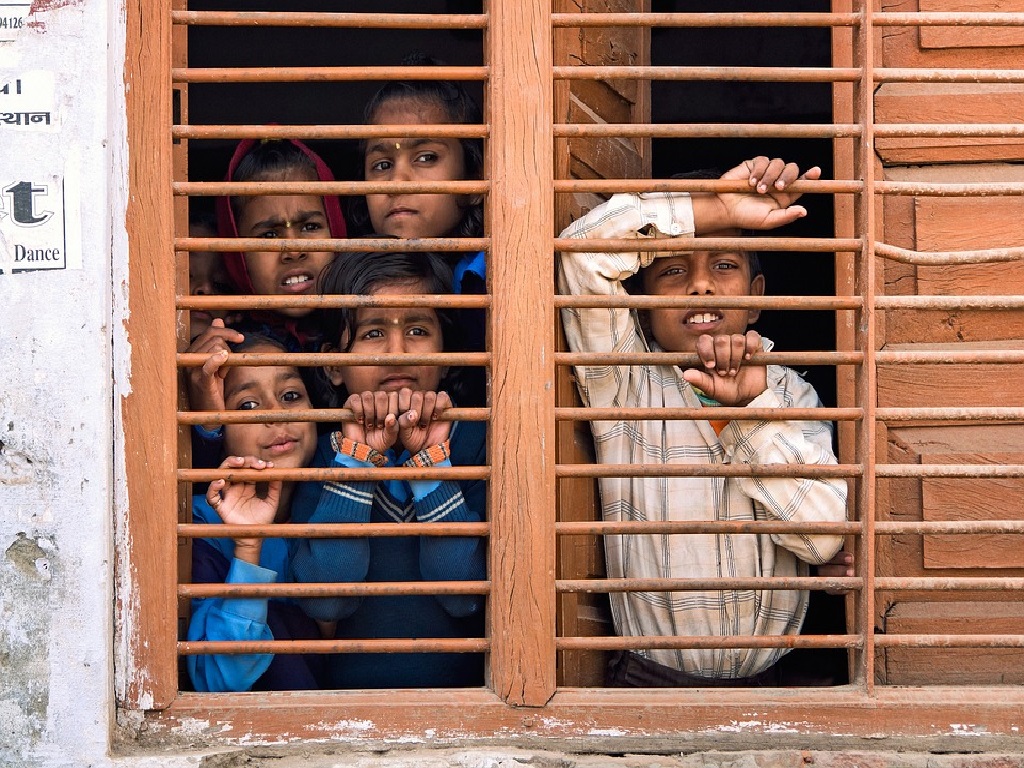

Supplement: Supplementary file 3 [file Data_Sheet_2.zip › Raw Images for Experiment 2/People/people04.jpg]

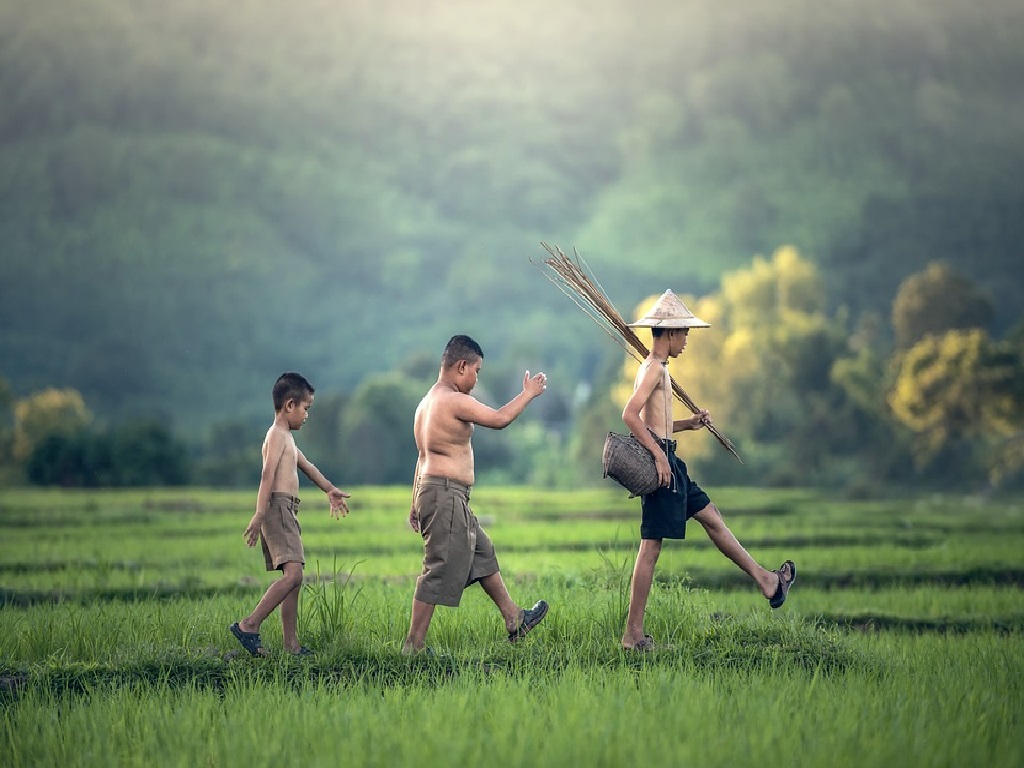

Supplement: Supplementary file 3 [file Data_Sheet_2.zip › Raw Images for Experiment 2/People/people05.jpg]

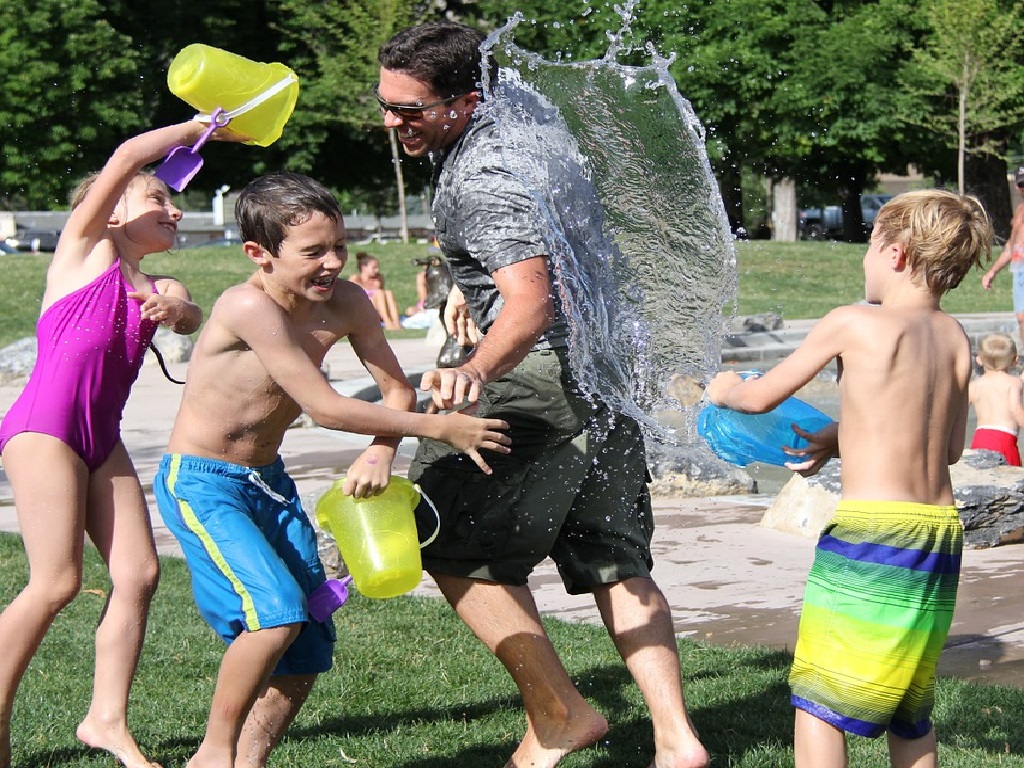

Supplement: Supplementary file 3 [file Data_Sheet_2.zip › Raw Images for Experiment 2/People/people09.jpg]

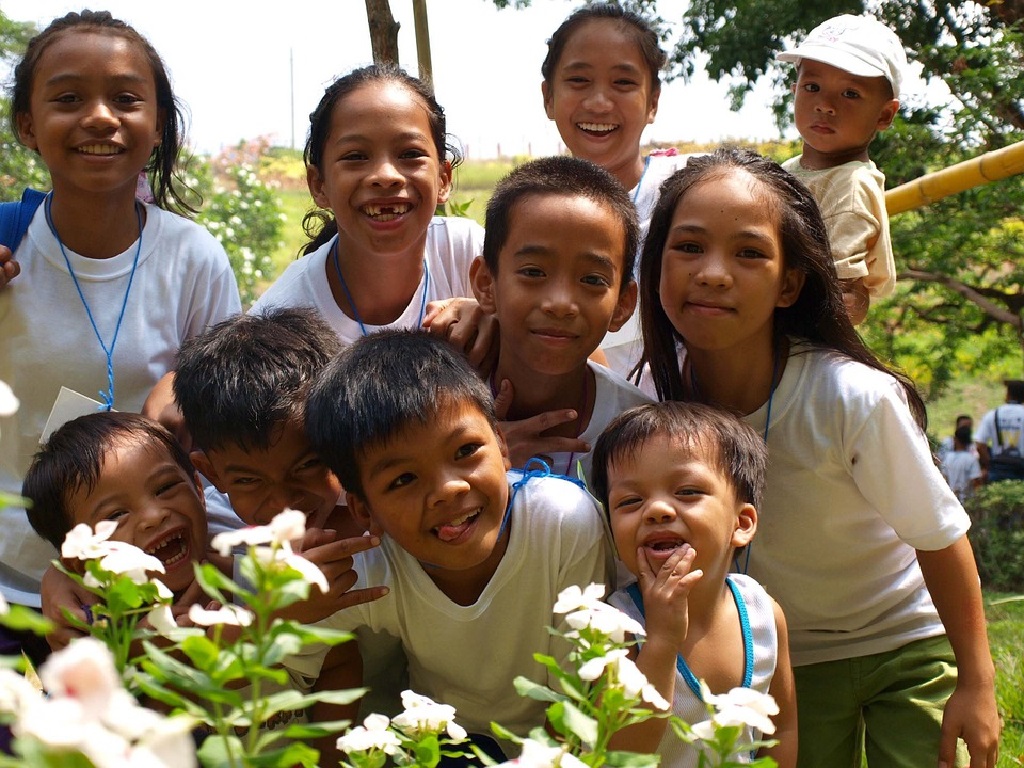

Supplement: Supplementary file 3 [file Data_Sheet_2.zip › Raw Images for Experiment 2/People/people10.jpg]

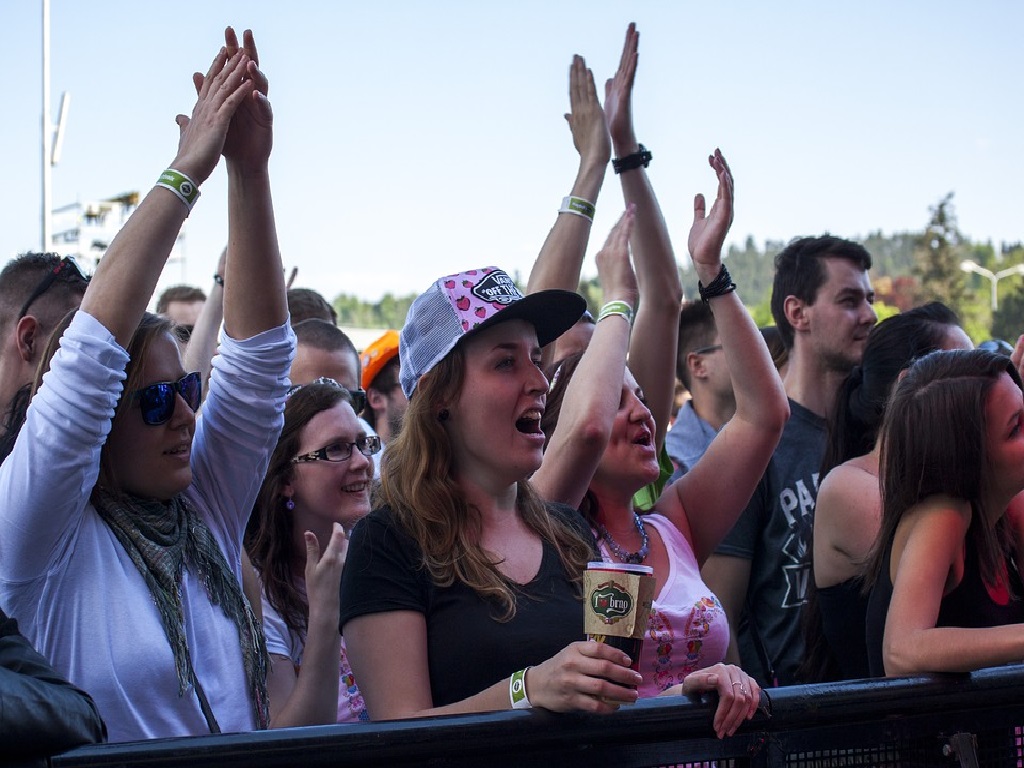

Supplement: Supplementary file 3 [file Data_Sheet_2.zip › Raw Images for Experiment 2/People/people11.jpg]

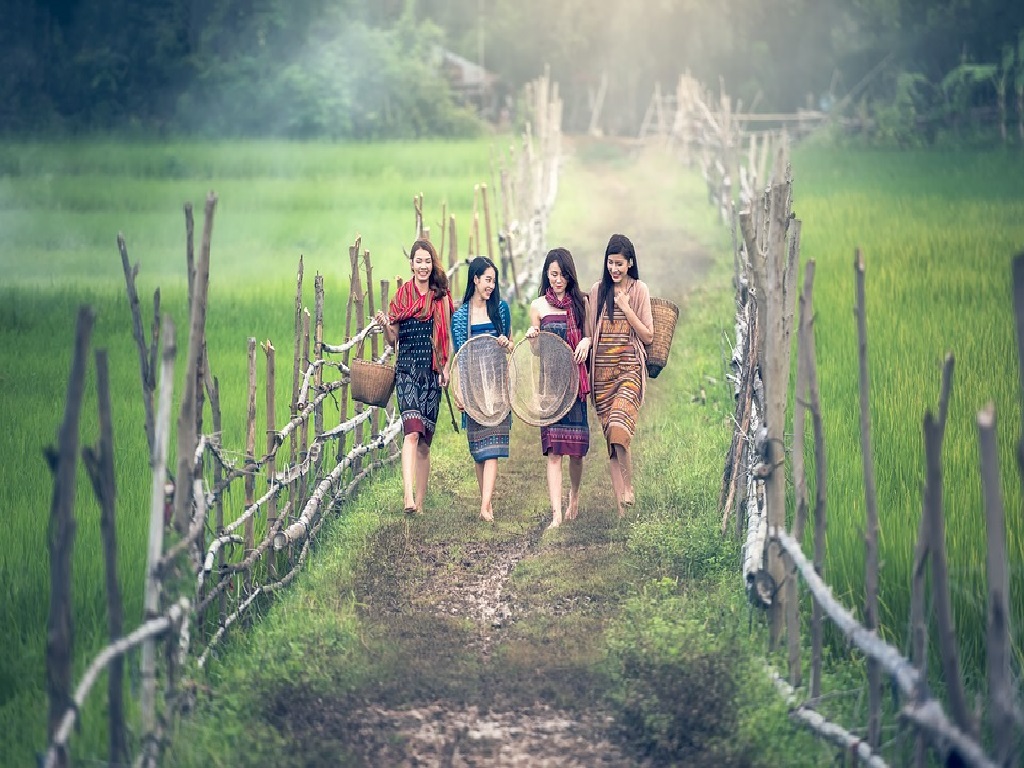

Supplement: Supplementary file 3 [file Data_Sheet_2.zip › Raw Images for Experiment 2/People/people12.jpg]

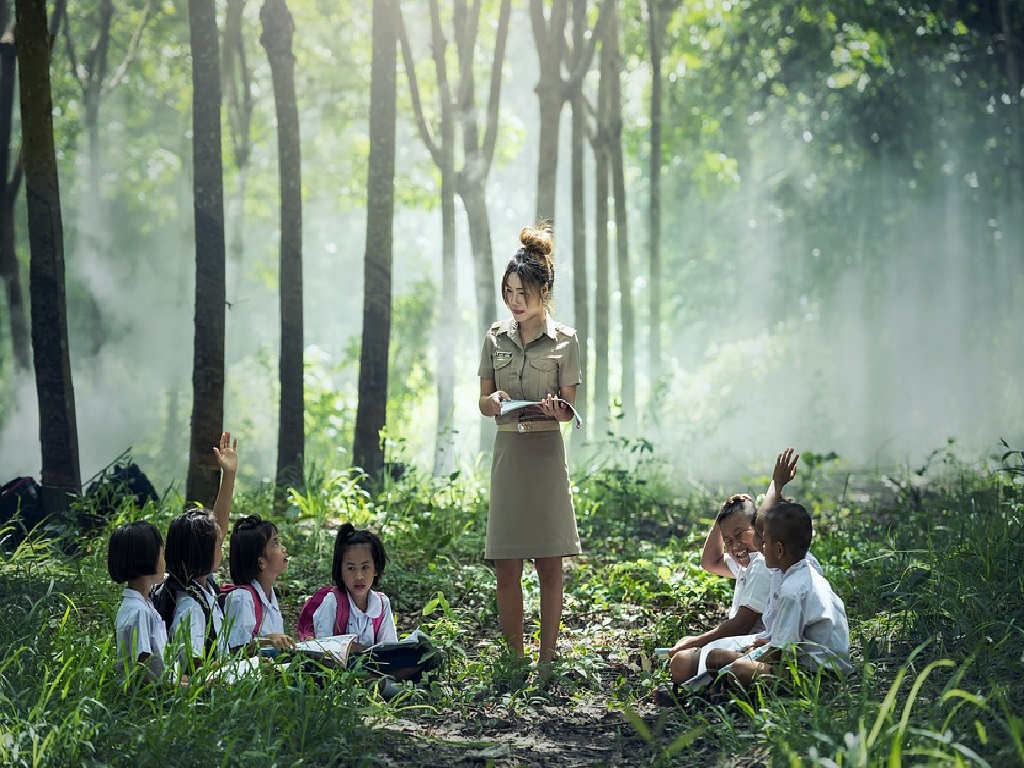

Supplement: Supplementary file 3 [file Data_Sheet_2.zip › Raw Images for Experiment 2/People/people14.jpg]

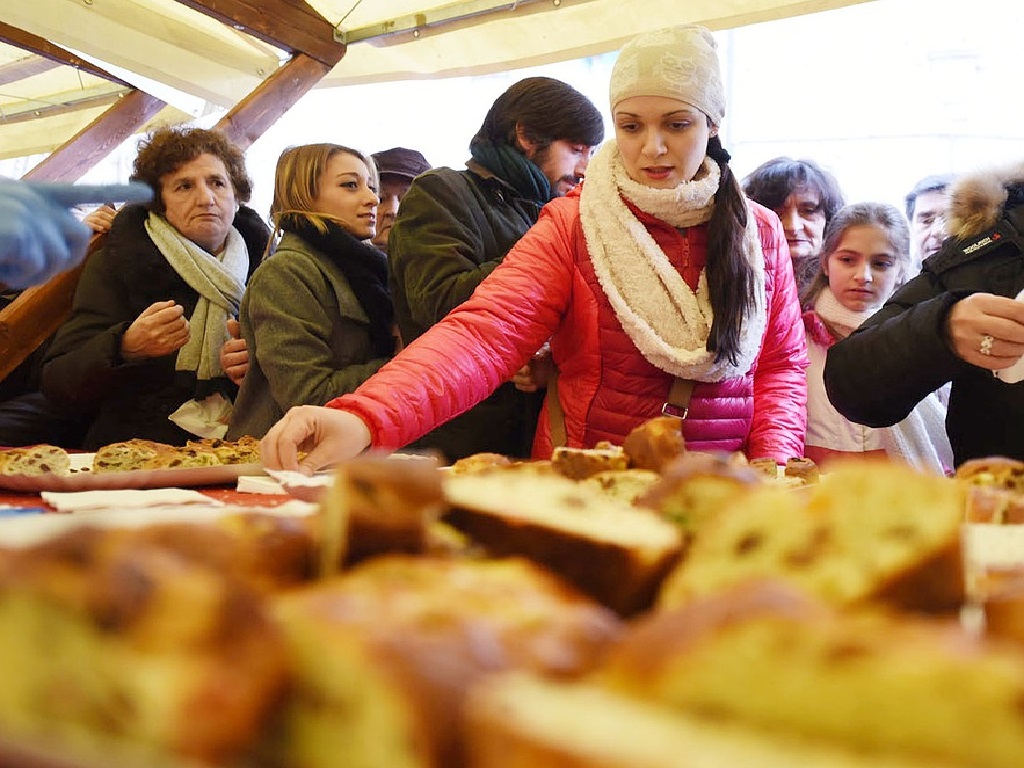

Supplement: Supplementary file 3 [file Data_Sheet_2.zip › Raw Images for Experiment 2/People/people15.jpg]

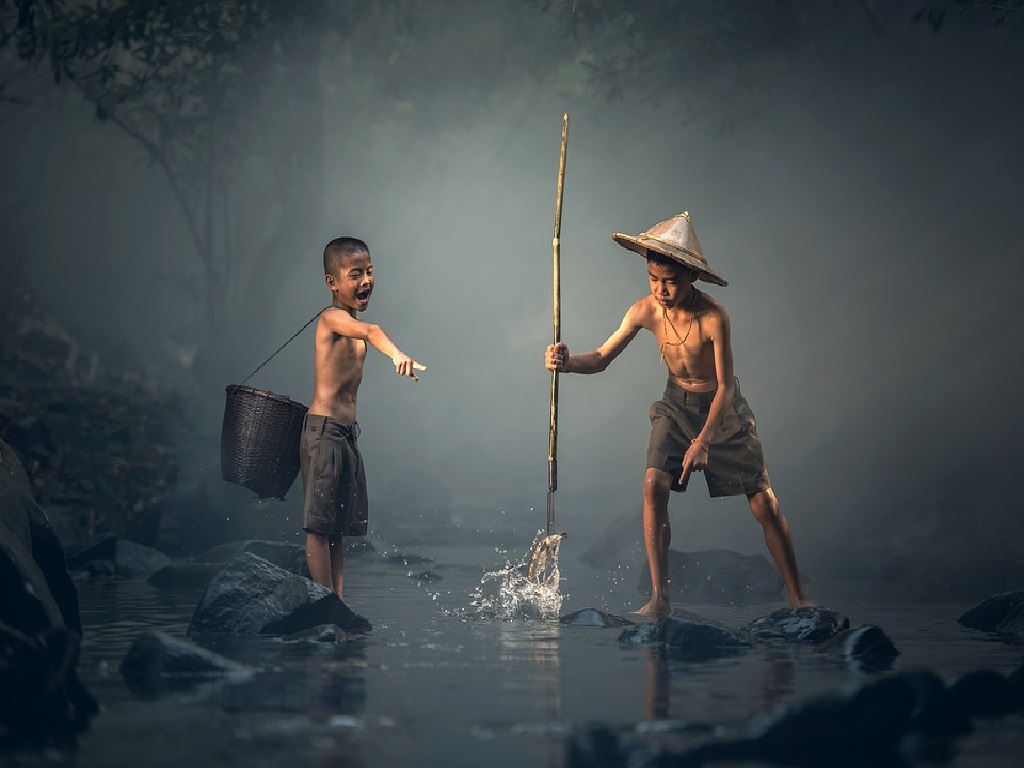

Supplement: Supplementary file 3 [file Data_Sheet_2.zip › Raw Images for Experiment 2/People/people16.jpg]

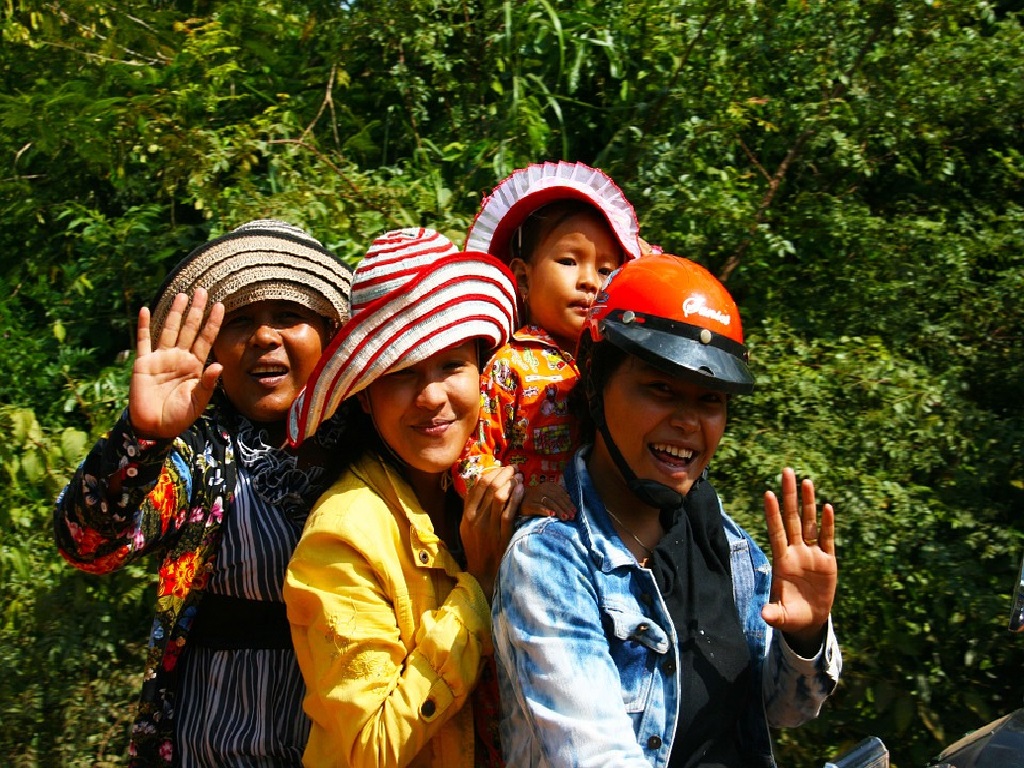

Supplement: Supplementary file 3 [file Data_Sheet_2.zip › Raw Images for Experiment 2/People/people18.jpg]

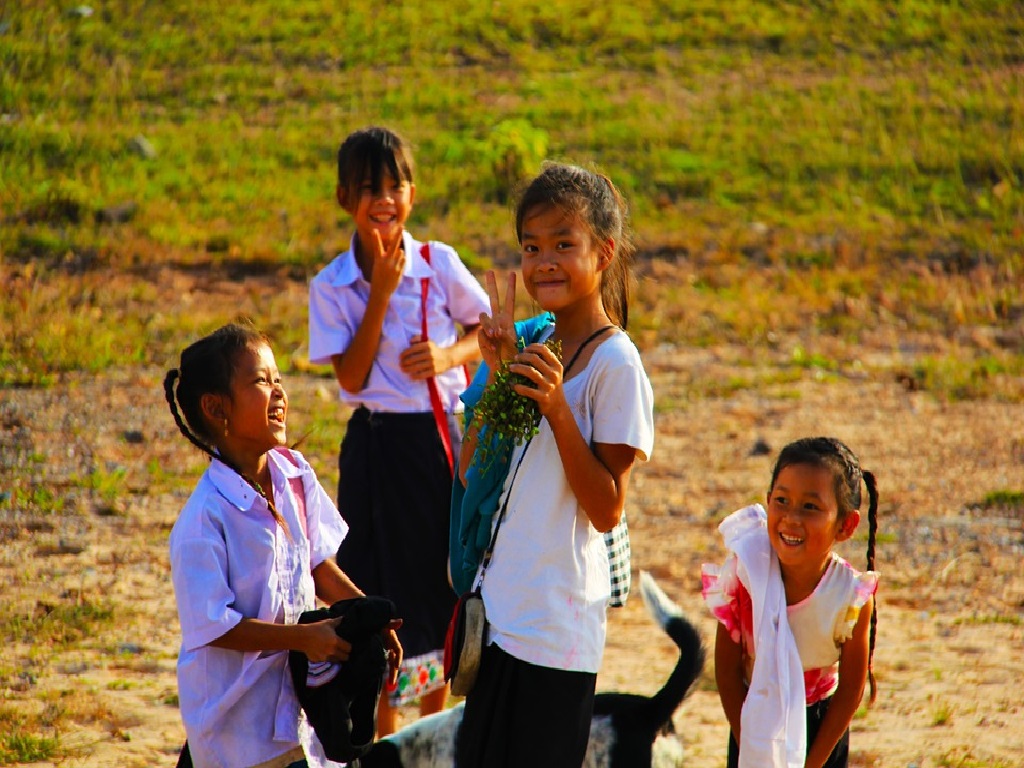

Supplement: Supplementary file 3 [file Data_Sheet_2.zip › Raw Images for Experiment 2/People/people19.jpg]

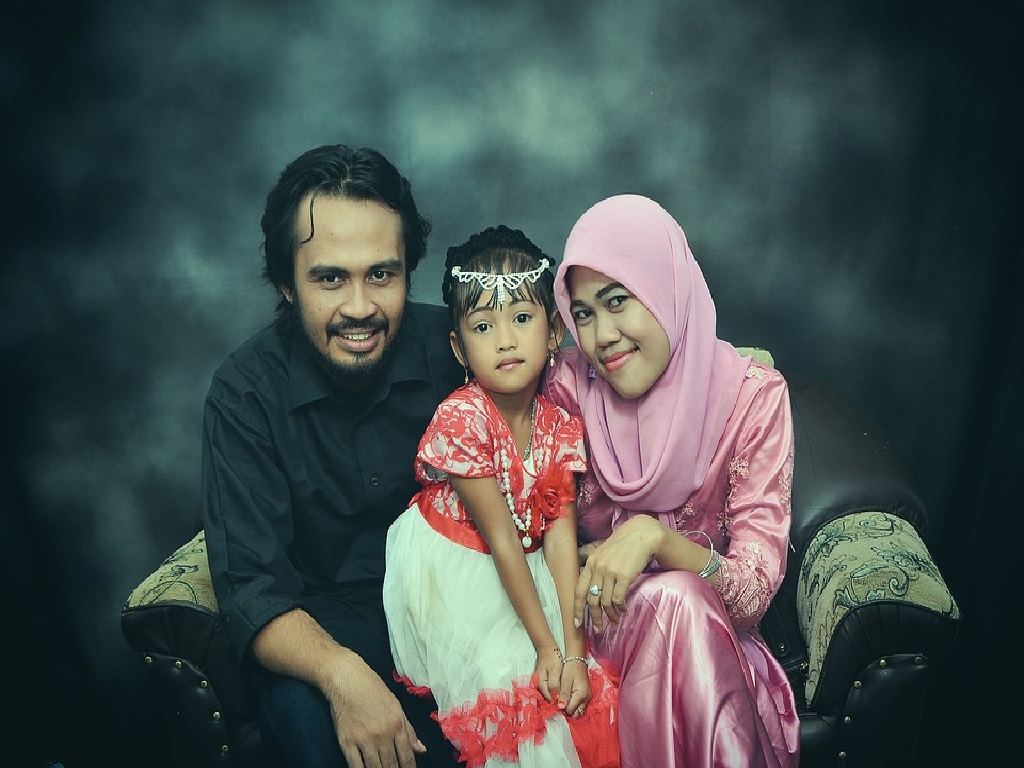

Supplement: Supplementary file 3 [file Data_Sheet_2.zip › Raw Images for Experiment 2/People/people20.jpg]

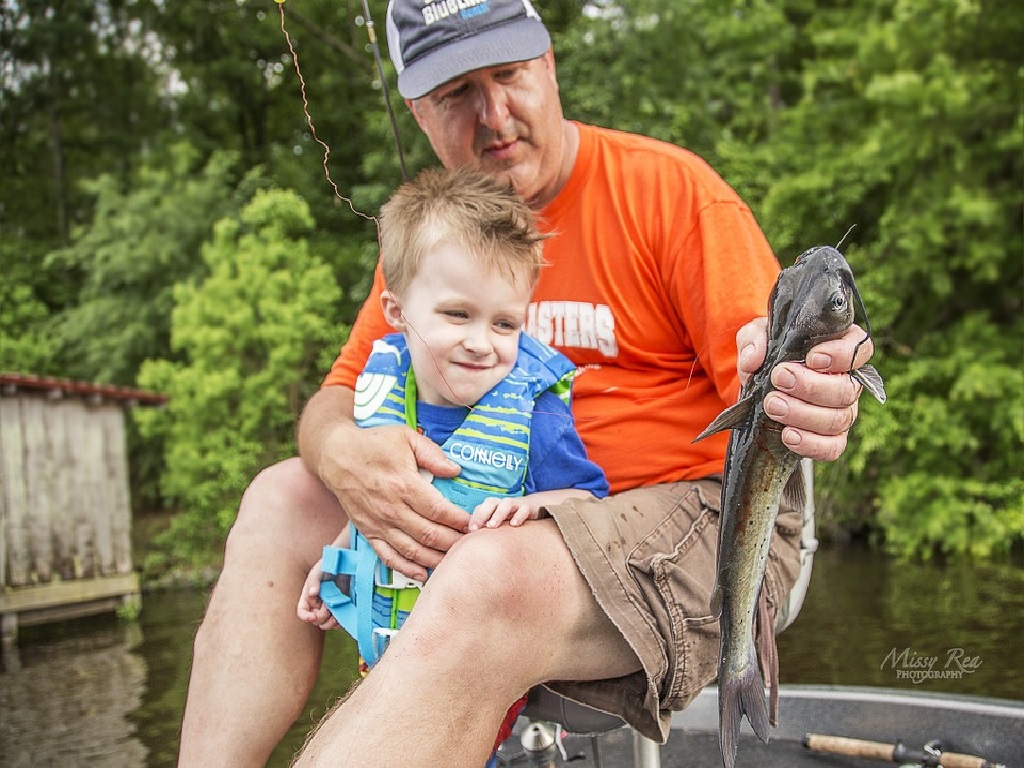

Supplement: Supplementary file 3 [file Data_Sheet_2.zip › Raw Images for Experiment 2/People/people24.jpg]

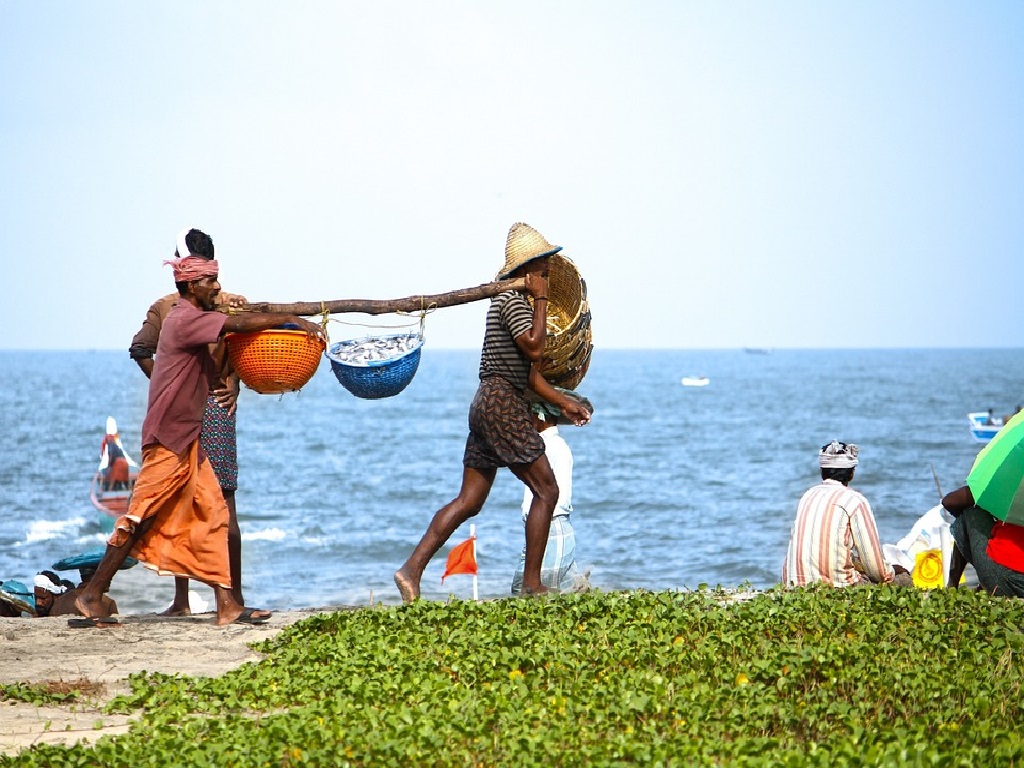

Supplement: Supplementary file 3 [file Data_Sheet_2.zip › Raw Images for Experiment 2/People/people26.jpg]

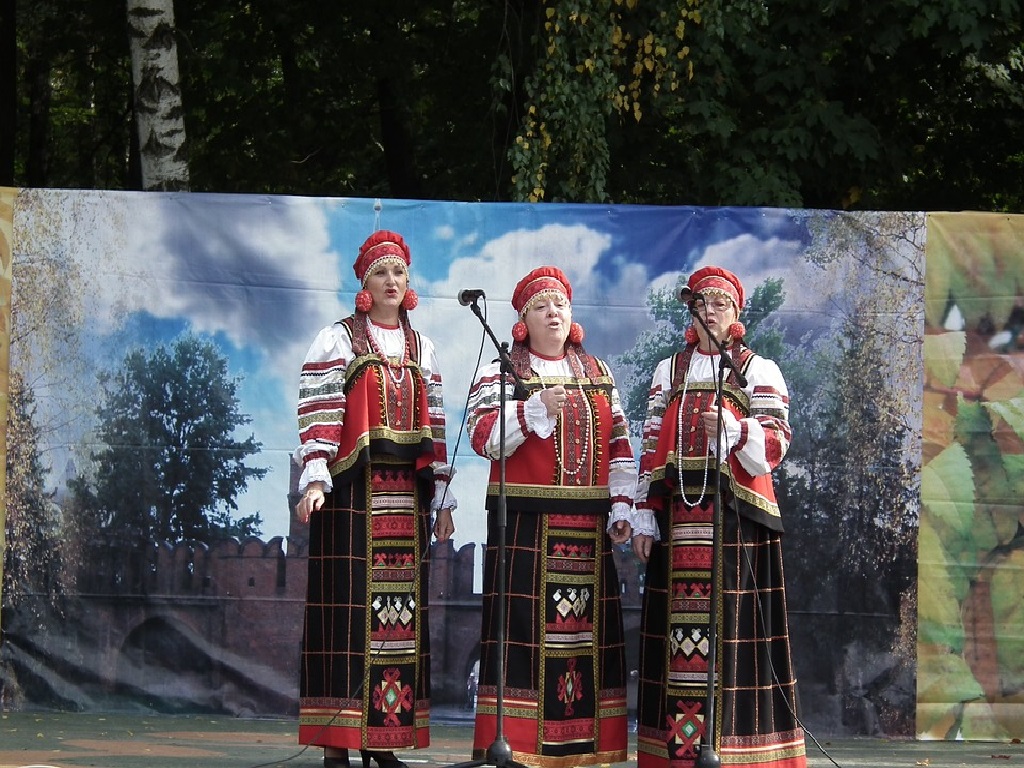

Supplement: Supplementary file 3 [file Data_Sheet_2.zip › Raw Images for Experiment 2/People/people28.jpg]
